# Selectivity, Speciation, and Substrate Control in the Gold-Catalyzed Coupling of Indoles and Alkynes

**DOI:** 10.1021/acs.organomet.2c00035

**Published:** 2022-02-10

**Authors:** Ryan G. Epton, William P. Unsworth, Jason M. Lynam

**Affiliations:** Department of Chemistry, University of York, Heslington, York, YO10 5DD, U.K.

## Abstract

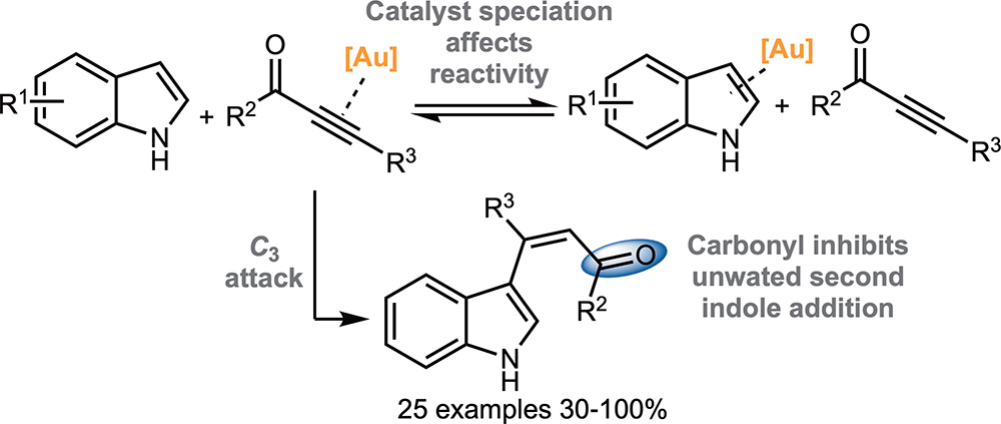

A convenient and mild protocol for the gold-catalyzed intermolecular coupling of substituted indoles with carbonyl-functionalized alkynes to give vinyl indoles is reported. This reaction affords 3-substituted indoles in high yield, and in contrast to the analogous reactions with simple alkynes which give *bis*indolemethanes, only a single indole is added to the alkyne. The protocol is robust and tolerates substitution at a range of positions of the indole and the use of ester-, amide-, and ketone-substituted alkynes. The use of 3-substituted indoles as substrates results in the introduction of the vinyl substituent at the 2-position of the ring. A combined experimental and computational mechanistic study has revealed that the gold catalyst has a greater affinity to the indole than the alkyne, despite the carbon–carbon bond formation step proceeding through an η^2^(π)-alkyne complex, which helps to explain the stark differences between the intra- and intermolecular variants of the reaction. This study also demonstrated that the addition of a second indole to the carbonyl-containing vinyl indole products is both kinetically and thermodynamically less favored than in the case of more simple alkynes, providing an explanation for the observed selectivity. Finally, a highly unusual gold-promoted alkyne dimerization reaction to form a substituted gold pyrylium salt has been identified and studied in detail.

## Introduction

Gold-catalyzed carbon–carbon and carbon–heteroatom bond formation reactions are powerful and synthetically versatile transformations. Coordination of an unsaturated substrate, such as an alkene or alkyne to an electrophilic Au(I) or Au(III) center, results in activation toward nucleophilic attack, and this has been exploited in a wide range of intra- and intermolecular coupling reactions.^1,2^

**Scheme 1:**
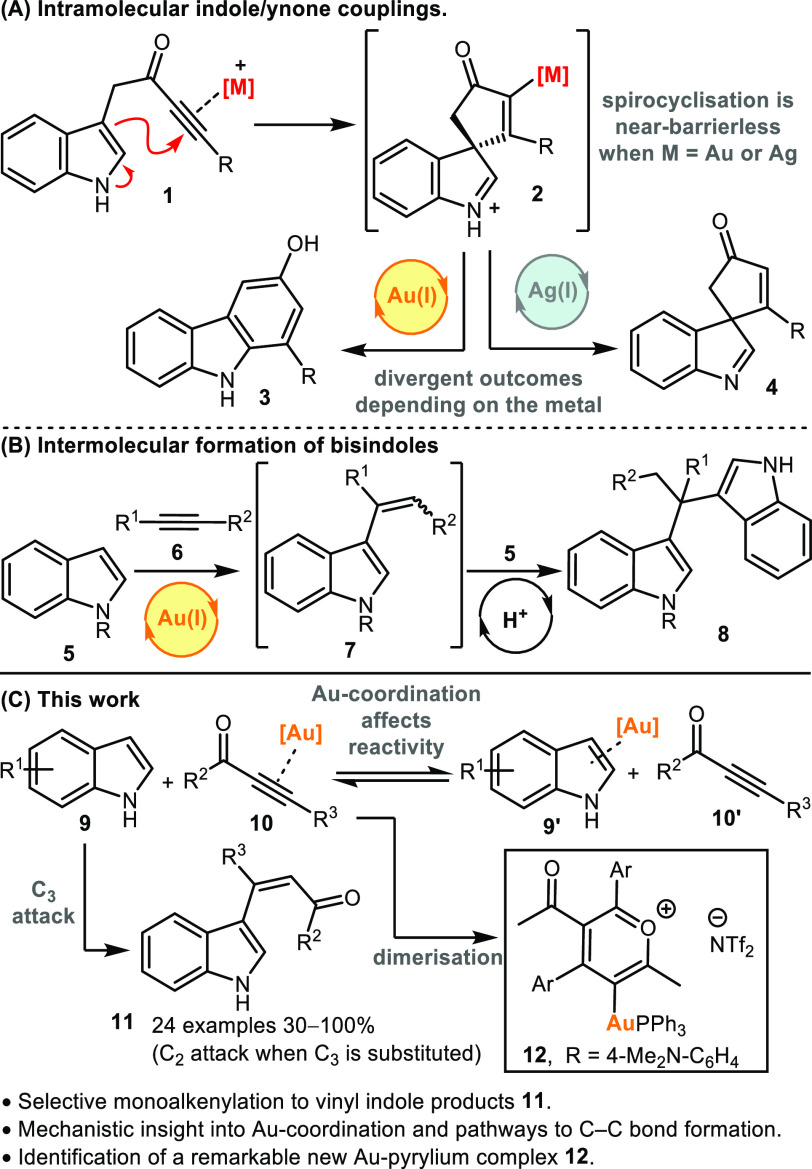
Intra- and Intermolecular Reactions of Indoles with Alkynes Catalyzed by Au(I) and Ag(I)

We have recently demonstrated how gold(I) catalysts promote intramolecular C–C bond formation in ynone-tethered indoles **1** to afford carbazoles **3** (Scheme 1A).^3^ Alternative catalysts such as AgOTf result in the formation of spirocycles **4**.^4−7^ DFT calculations suggest that carbon–carbon bond formation proceeds by nucleophilic attack onto a gold- or silver-bound alkyne (**1** → **2**).^8,9^ The calculations indicate that the spirocycle **4** is a kinetic product, formed though indole *C*_*3*_-attack onto the activated alkyne, and carbazole **3** is the thermodynamic product, formed through the corresponding *C*_*2*_-addition when the spirocyclization step is reversible. In all cases the calculated transition states for carbon–carbon bond formation are located at low energy (<41 kJ mol^–1^ with respect to the reference state) and the C–C bond formation (**1** → **2**) step was calculated to be almost barrierless. These results, and others,^10−17^ demonstrate the synthetic versatility of **1** as a framework to access a range of important structural motifs.

The ease with which ynone-tethered indoles **1** can be converted into scaffolds **3** and **4** provided encouragement that hitherto unknown intermolecular variants could be developed to enable the facile C–H alkenylation of simple, unfunctionalized indoles.^18−21^ 3-Vinyl indoles (e.g., **7**, Scheme 1B) are highly important molecules, both as synthetic building blocks for biologically significant indole-based drugs and natural products, and in their own right, with various biologically active 3-vinyl indoles and methods to synthesize them known.^22−27^ However, making 3-vinyl indoles via the gold-catalyzed coupling of indoles and alkynes is challenging; while the required coupling reaction (**6** → **7**, Scheme 1B) can be promoted using gold catalysis, the vinyl indole products **7** undergo rapid reaction with a second molecule of indole to form *bis*indolemethanes **8**.^28−30^ It is possible to inhibit the second indole addition by designing systems in which the monofunctionalized intermediate is trapped in situ (e.g., in a cyclization with a tethered nucleophile),^28,31^ but this approach does not allow access to vinyl indoles like **7**. Recently, Lee and co-workers have shown that vinyl indoles can be prepared in excellent yield from the reaction of 2-substituted indoles with an excess of alkyne at low gold catalyst loadings, although with indole itself, *bis*indolemethanes were still generated.^32^ While this could be circumvented through the use of a 2-boryl-substituted indole and subsequent deprotection, the selective monoalkenylation of unsubstituted indole remains an unsolved challenge.

The formation of the *bis*indolemethanes follows an interesting mechanistic pathway. Both experimental^33^ and computational^34^ data with indole and pyrrole nucleophiles indicate that the initial coupling with the alkyne is gold-catalyzed, whereas the subsequent addition of the second heterocycle to vinyl **7** is Brønsted acid-catalyzed. Protonation occurs at the alkene group of the vinyl indole to give a carbocation which is then attacked by another molecule of indole; even in cases where no Brønsted acidic reagents are used, trace acid formed in situ is usually sufficient to promote this transformation.^5,35^ Avoiding *bis*indolemethane formation is therefore a significant challenge, but one we were confident could be overcome by harnessing the unique reactivity of ynones.^3,5,8,9,11,13^ In our previous work, we have shown that the electron-withdrawing carbonyl group of the ynone moiety can significantly enhance the reactivity of the alkyne when treated with a Au(I) catalyst. This enables ynones to be coupled with indoles under very mild conditions. Furthermore, the same carbonyl group has been shown to suppress Brønsted acid-catalyzed migration reactions in the resulting products—both features were postulated to promote the selective formation of the desired vinylindoles **11** in this study (Scheme 1C).

The successful realization of this strategy is reported herein. A simple method for the synthesis of a range of vinyl indoles **11** has been established, using a cationic gold(I) catalyst to promote the coupling of indoles and ynones, as well as other electron deficient ester- and amide-based alkyne derivatives (Scheme 1C). Our theory that the carbonyl group can enhance the first vinylation reaction but suppress subsequent reactions appears to be valid, given that reactions proceed under mild conditions and *bis*indolemethane formation is completely avoided. A series of mechanistic and computational experiments have also been performed that enable a deeper understanding of the nature of the states involved in C–C bond formation, and help to explain how the site of Au-coordination both influences the regioselectivity of the vinylindole formation and accounts for the stark difference in reactivity between the intermolecular and intramolecular variants. An investigation into the speciation of the gold catalyst is also presented, which enabled the identification of a novel gold pyrylium complex **12**, arising from the dimerization of two ynones.

## Results and Discussion

### Catalyst Optimization and Synthetic Scope

Our initial experiments focused on assessing the intermolecular addition of indole **5** to ynone **13**, as a like-for-like comparison with the intramolecular cyclization of **1**: the results are summarized in Table 1. First, AgOTf, Cu(OTf)_2_ and SnCl_2_.2H_2_O were tested, as these reagents were found to be excellent catalysts for the intramolecular spirocyclization of indoles **1** into **4** in our previous work (Scheme 1A). However, all were ineffective in this reaction; no consumption of either **5** or **13** was observed when analyzed using ^1^H NMR spectroscopy. A more reactive catalyst was therefore sought, and the Gagosz catalyst [Au(NTf_2_)(PPh_3_)]_2_·Tol (**Au-1**) was chosen for its well-known activity and ready availability.^36^ Pleasingly, **Au-1** effectively catalyzed the coupling, promoting 90% conversion into vinyl indole **14a** at room temperature (entry 4) at 5 mol % catalyst loading, with further optimization enabling complete conversion into **14a** at 40 °C over 2 h (entry 5; for additional optimization experiments see the Supporting Information). The vinyl indole product **14** was formed as a mixture of *E-* and *Z-* isomers, which are believed to equilibrate in solution. The formal electrophilic addition occurred at the *C*_*3*_-position of the indole as expected, and pleasingly, no evidence for the formation *bis*indolemethane product **16a** was obtained. This contrasts starkly to the reaction outcome when indole **5** was reacted with phenyl acetylene **15** under the same conditions; in this case the only product observed was *bis*indolemethane **16b**.

**Table 1:**
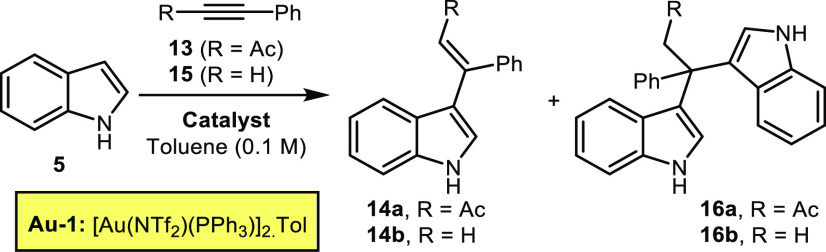
Intermolecular Reaction of Indole **5** with Ynone **13** and Alkyne **15**

entry	alkyne^a^	catalyst	temp (°C)	time (h)	product^b^ (*E*/*Z*)
1	**13**	AgOTf (10 mol %)	RT	24	–
2	**13**	Cu(OTf)_2_ (10 mol %)	RT	24	–
3	**13**	SnCl_2_·2H_2_O (10 mol %)	RT	24	–
4	**13**	**Au-1** (5 mol %)	RT	24	**14a**, 90% (73:27)
5	**13**^c^	**Au-1** (5 mol %)	40	2	**14a**, 100% (71:29)
6	**15**^d^	**Au-1** (5 mol %)	40	2	**16b**, 81%
7	**15**^d^	**Au-1** (5 mol %) C_2_CO_3_ (10 mol %)	40	16	**16b** trace
8	**15**^d^	**Au-1** (5 mol %) NEt_3_ (10 mol %)	40	16	–

a 1.0 equiv unless stated.

b Conversion determined by the ratio of remaining indole to both geometrical isomers of **14** by ^1^H NMR spectroscopy.

c 1.5 equiv of **13** was used.

d 1.5 equiv of **15** was used.

Attempts to suppress the formation of **16b** by adding basic additives to quench trace Brønsted acid formation were unsuccessful with the basic additives inhibiting the reaction (entries 7–8).

**Scheme 2:**
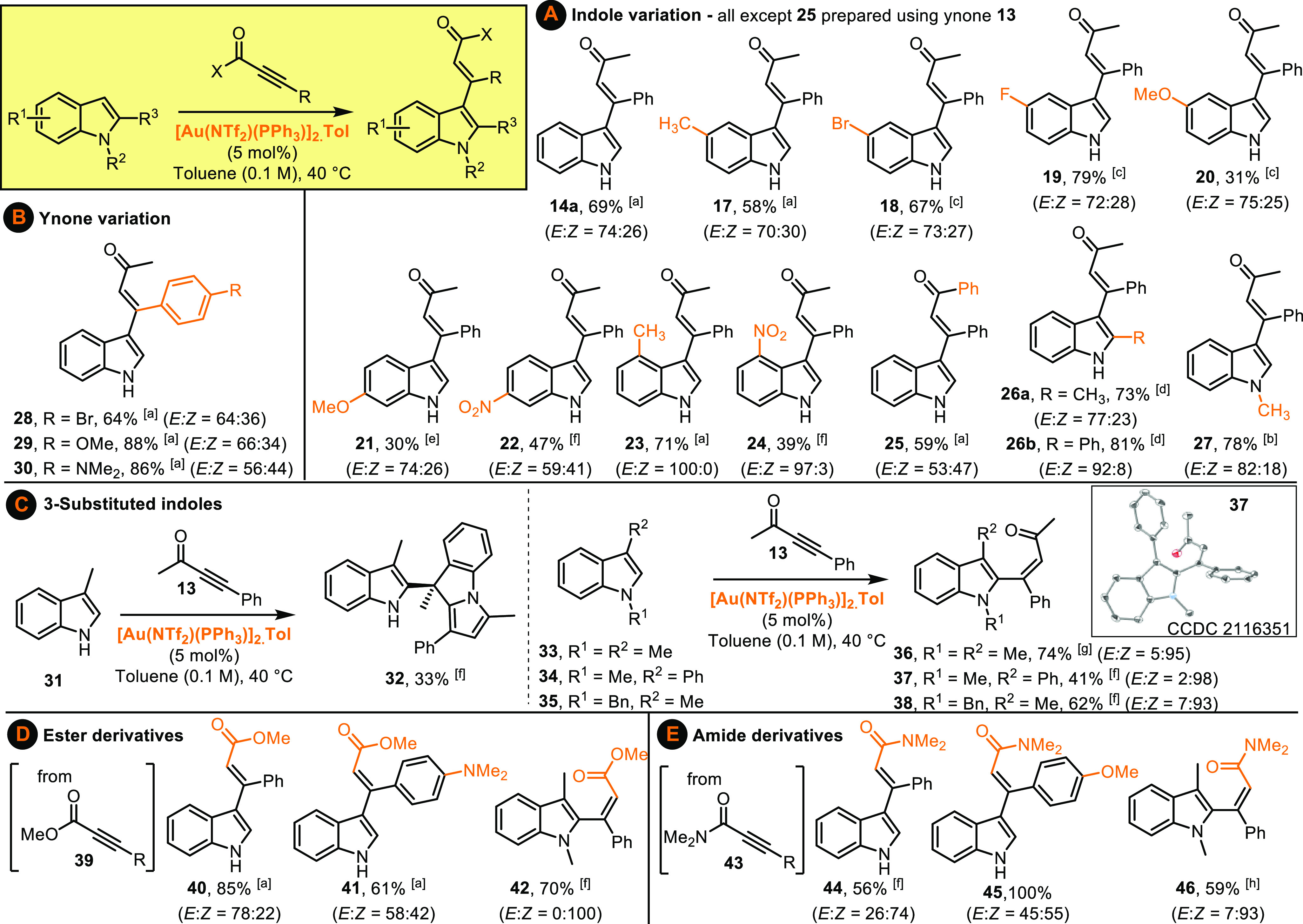
Selective Gold-Catalyzed Monovinylation of Indoles with Electron Deficient Alkynes 2 h reaction time. 3 h reaction time. 18 h reaction time. 19 h reaction time. 21 h reaction time at room temperature. 24 h reaction time. 27 h reaction time. 48 h reaction time.

With conditions for intermolecular indole-ynone coupling established, attention next moved to exploring the scope of the reaction. Variation of the indole coupling partner was first examined, with a range of indoles bearing electronically diverse substituents around the benzenoid portion tested, and all performed well under the standard conditions (**14**–**25**Scheme 2A). Pleasingly, functionalization on the pyrrole ring of the indole is also tolerated, with indoles bearing *C*_*2*_*-* and *N*_*1*_-substituents formed in good yields (**26a**, **26b**, **27**, Scheme 2A). The ynone coupling partner can also be varied, which is noteworthy given that the electronic properties of the ynone can have a major influence on reaction efficiencies in related processes (**28**–**30**, Scheme 2B).

We were also keen to examine the reactivity of an indole substrate in which the more reactive *C*_*3*_-position is blocked, and therefore ynone **13** was reacted with skatole **31** (Scheme 2C). Either *C*_2_-vinylation or dearomative *C*_3_-difunctionalisation were considered to be the two most likely outcomes in this case, but neither of these products were isolated; instead, the dominant component of the reaction mixture was the three-component reaction product **32**. This product presumably formed via an initial *C*_*2*_-vinylation, followed by a cascade process, analogous to that previously observed by Tian and co-workers for a related system treated under Brønsted acid-catalyzed conditions.^37^ The formation of **32** was encouraging nonetheless, as it demonstrated that *C*_*2*_-addition to skatole was occurring, but a subsequent condensation reaction did not allow isolation of the desired vinylindole product. Pleasingly, the introduction of an *N*-substituent prevented the three-component coupling, with indoles **33**–**35** all being converted into vinylindoles **36**–**38** in good yields, with selective vinylation at the indole *C*_*2*_-position. Finally, we tested whether other electron deficient alkynes may react similarly to ynones, and pleasingly, ester- (**40**–**42**, Scheme 2D) and amide-based (**44**–**46**, Scheme 2E) products were formed in good to excellent yields in the same way. These reactions are practically very simple to perform, and across all reaction series, the only change needed to the standard method was to vary the reaction time (based on TLC analysis). Most products were isolated as mixtures of geometrical isomers, with the observed *E*/*Z* ratios believed to be thermodynamic outcomes, resulting from facile alkene isomerism enabled by conjugation of the electron-rich indole into the carbonyl. For products formed via vinylation at the indole *C*_*3*_-position, the *E* isomer tends to predominate, based on chemical shift trends, nOe studies, and comparisons to literature NMR data (see Supporting Information).^38^ In *C*_2_-vinylation examples (**36**–**38**, **42**, **46**) the *Z* isomer is formed as the major geometrical isomer, with the assignment of product **37** based on X-ray crystallographic data^39^ and the others by analogy.

### Experimental and Computational Mechanistic Studies

The results from the synthetic studies raised a number of mechanistic questions about the pathways underpinning the formation of the substituted indole compounds. These were as follows:

- What is the origin of the selectively for a 1:1 coupling in these reactions, compared to the more conventional addition of two molecules of indole to the alkyne to give a *bis*indolemethane?
- What factors influence the stark difference in the relative ease of the intra- and intermolecular variants of the coupling between an indole and ynone?
- What controls the *C*_*2*_ versus *C*_*3*_ regioselectivity addition to the indole, especially when the *C*_3_ position is substituted?

A combined experimental and computational mechanistic study was undertaken to address these questions. In the first instance, the interactions between the gold catalyst [Au(NTf_2_)(PPh_3_)]_2_·Tol and the individual alkyne and heterocyclic substrates were investigated; the idea here was that a better understanding of gold speciation with respect to both reaction components would shed light on the observed reactivity, both in the intra- and intermolecular variants. ^31^P{^1^H} NMR spectroscopy was therefore used to study the speciation, and these experimental data were compared to calculations using density functional theory (DFT). Full details of the computational methods are provided in the Supporting Information, and all energies quoted are Gibbs energies in kJ mol^–1^ at 298.15 K. In the calculations the gold catalyst was treated as [Au(PPh_3_)]^+^. Experimentally, there is evidence of solvent-dependent ion-pairing in these systems,^40−42^ and we have investigated the potential effects computationally (see Supporting Information).

**Figure 1:**
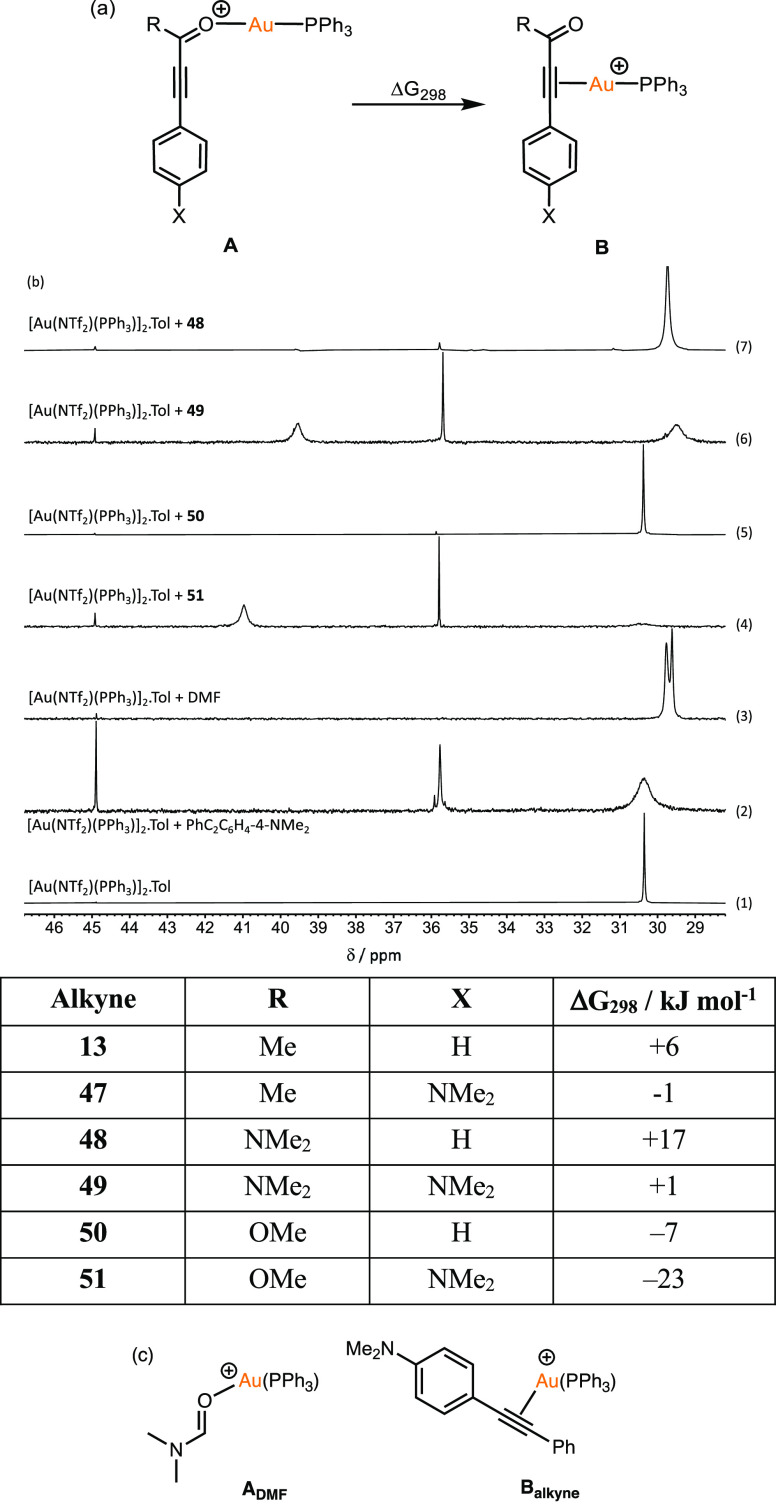
(a) Isodesmic reaction used to compare η^1^(O) and η^2^(π)-bound forms of substituted alkynes. Energies are Gibbs energies at 298.15 K at the D3(BJ)-PBE0/def2-TZVPP//BP86/SV(P) level with COSMO solvation in CH_2_Cl_2_. (b) ^31^P{^1^H} NMR spectra in CD_2_Cl_2_ solution showing the interaction between [Au(NTf_2_)(PPh_3_)]_2_·Tol and different substrates performed at a 1:2 gold:substrate (c) proposed η^1^(O) and η^2^(π) binding for DMF and PhC_2_C_6_H_4_-4-NMe_2_.

The interaction between a range of substituted alkynes and [Au(PPh_3_)]^+^ was studied first. As shown in Figure 1a, the gold has the potential to exhibit either η^1^(O)-binding, **A**, or η^2^(π) binding, **B**, to the alkynes. It was reasoned that the energy balance between these different binding modes would be influenced by changes to the substituents on the phenyl-ring of the alkyne, and whether a ketone, amide, or ester substituent was present.

The energy balance between states **A** and **B** was evaluated by DFT for a range of substituted alkynes (Figure 1a). Two important trends were evident in the data. First, using an amide-substituted alkyne (see **48** and **49**) is predicted to increase the relative stability of the *O*-bound form, **A**. Second, the introduction of an electron-donating NMe_2_-group into the 4-position of the alkyne should have the opposite effect and increase the affinity of the η^2^(π)-bound form, **C** (see **47**, **49**, and **51**).

These predictions were supported by experimental data which used ^31^P{^1^H} NMR spectroscopy to probe the speciation of the gold complex in solution. Experiments performed with DMF and PhC_2_C_6_H_4_-4-NMe_2_ provided reference spectra for η^1^(O) (**A**_**DMF**_) and η^2^(π) binding (**B**_**alkyne**_), respectively (Figure 1b, spectra (2) and (3), Figure 1c). Spectrum (4), obtained after treatment of a CH_2_Cl_2_ solution of [Au(NTf_2_)(PPh_3_)]_2_·Tol with **51**, exhibited a sharp resonance at δ 35.8, consistent with the η^2^(π)-alkyne coordination mode, **B**, being the dominant form. A resonance at δ_p_ 41.0 was assigned to the formation of a product arising from alkyne dimerization which will be discussed in detail later. An analogous reaction with **50**, which lacks the NMe_2_-group on the aryl ring, did not show any change when compared with [Au(NTf_2_)(PPh_3_)]_2_·Tol, spectrum (5). However, spectrum (7), obtained from a reaction with amide **48**, was dominated by a sharp single resonance at δ_p_ 29.7, consistent with binding mode **A**. Using amide **49**, which also possessed a NMe_2_ substituent on the aryl group, showed evidence for both η^1^(O) and η^2^(π) binding, spectrum (6). Several spectra exhibited a resonance at δ_p_ 45.5, which is likely due to [Au(PPh_3_)_2_]^+^ on the basis of a comparison with an authentic sample.

**Figure 2:**
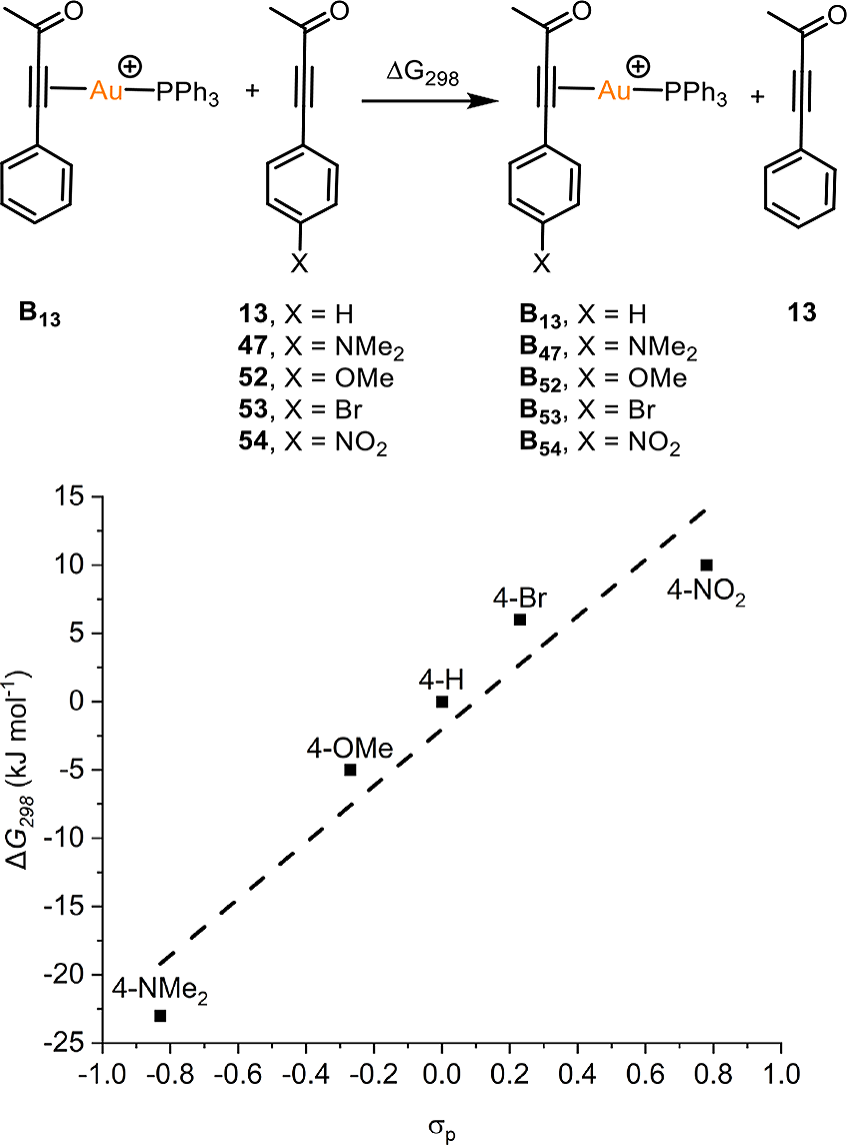
Isodesmic reaction used to calculate affinity of alkynes for gold. Energies are Gibbs energies at 298.15 K at the D3(BJ)-PBE0/def2-TZVPP//BP86/SV(P) level with COSMO solvation in CH_2_Cl_2_ (top). Linear free energy relationship between the calculated change in free energy against Hammett parameter σ_p_ (bottom). Dashed line shows fit to a least mean squares linear regression (*R*^2^ = 0.92).

Next, the relative affinity of the gold cation toward the triple bonds of a range of alkynes was evaluated through a series of calculated isodesmic reactions (Figure 2). The change in free energy for alkyne substitution of the parent complex **B**_**13**_ by alkynes with various substituents in the 4-position of the phenyl ring was calculated using DFT. These data demonstrate the presence of a linear free energy relationship between the relative energy change on binding to the gold and the Hammett (σ_p_) parameter of the aryl substituent. The positive slope indicates that electron-donating groups favor η^2^(π)-alkyne coordination to the gold cation. This is consistent with alkyne binding being a net donor to the gold with π-backdonation to the vacant π*-orbitals on the ligand (a key factor affecting the stability of midtransition metal alkyne complexes) not being a dominant effect.^43,44^

**Figure 3:**
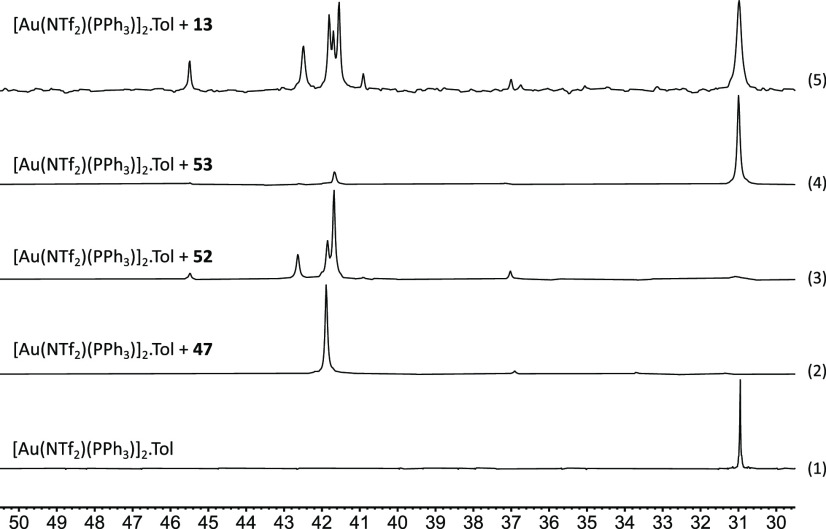
^31^P{^1^H} NMR spectra of a mixture of [Au(NTf_2_)(PPh_3_)]_2_·Tol and ynones in a 1:10 gold:substrate ratio in CD_2_Cl_2_ solution.

The predicted enhanced affinity for the gold cation by electron-rich alkynes was supported by ^31^P{^1^H} NMR spectroscopy. Reaction of [Au(NTf_2_)(PPh_3_)]_2_·Tol with 10 equiv of bromine-substituted alkyne **53** resulted in little change to the ^31^P{^1^H} NMR spectrum (Figure 3, spectrum (4)) with only a small amount of starting material consumed.^45^ The corresponding reaction with **13** resulted in a complex series of resonances in the region between δ 40 and 45: the resonance for [Au(NTf_2_)(PPh_3_)]_2_·Tol was still present (spectrum (5)). In contrast, in spectrum (3) when the 4-OMe-substitued alkyne, **52**, was used almost all of the starting material was consumed and again a series of new resonances between δ 40 and 45 were observed. A resonance at δ 37.0 was also present which, by analogy of the results in Figure 2, may represent a complex with an η^2^(π)-bound alkyne. Reaction between [Au(NTf_2_)(PPh_3_)]_2_·Tol and 10 equiv of NMe_2_-substituted **47** resulted in a single new resonance at δ_p_ 41.9. The chemical shift of this resonance is markedly different from those assigned to the η^1^(Ο) and η^2^(π)alkyne (Figure 2), which typically appear at δ_p_ 29 and 35, respectively. The species responsible for the resonance at δ_p_ 41.9 was identified as a gold-substituted pyrylium salt and is discussed later.

These results demonstrate that the gold cation may readily coordinate to the alkyne and that electronic effects have a significant influence on the binding mode (i.e., η^1^(Ο) versus η^2^(π)alkyne), with electron-donating substituents on the aryl substituent favoring the required η^2^(π)alkyne binding.

**Figure 4:**
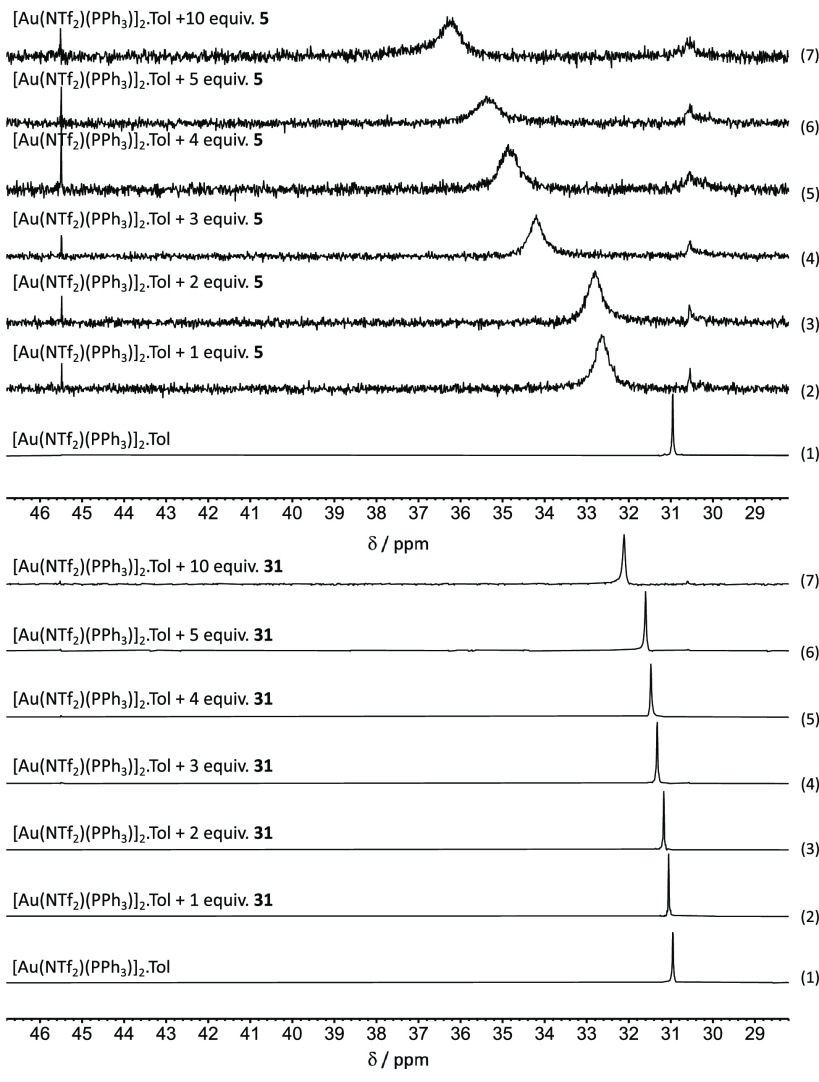
^31^P{^1^H} NMR spectra of [Au(NTf_2_)(PPh_3_)]_2_·Tol with different ratios of **5** (top) and **31** (bottom) in CD_2_Cl_2_.

Next, potential gold coordination to the indole component was examined. Although the carbon–carbon bond formation step in the vinylation reaction was expected to occur through nucleophilic attack of the indole onto a gold-coordinated alkyne, we reasoned that the indoles could also be suitable ligands for the Au(I) cations themselves, and thus compete for the catalyst with the ynone. Experimental evidence for this interaction between the gold and heterocycle was obtained through a series of titrations between [Au(NTf_2_)(PPh_3_)]_2_·Tol and indole, **5**, or skatole, **31**, in CD_2_Cl_2_ solution, monitored by ^31^P{^1^H} NMR spectroscopy. In both cases, the appearance of the spectra was concentration-dependent (Figure 4). For indole, a broad resonance was observed at all [Au(NTf_2_)(PPh_3_)]_2_·Tol:**5** ratios, with a shift to lower field of ca. 4.3 ppm when moving from a 1:1 to a 1:10 ratio. For skatole, a single sharp resonance was observed, which exhibited a smaller concentration-dependent change in chemical shift.

**Scheme 3:**
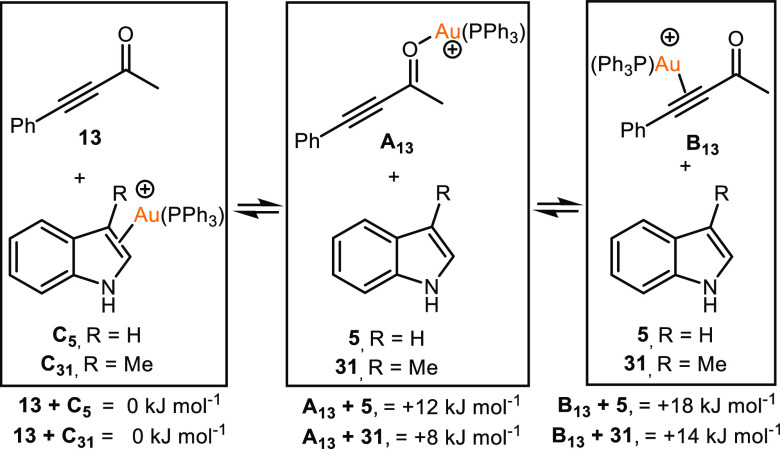
Relative Changes in Free Energy on Coordination of Indole, **5**, Skatole, **31**, and **13** to [Au(PPh_3_)]^+^ Energies are Gibbs energies at 298.15 K at the D3(BJ)-PBE0/def2-TZVPP//BP86/SV(P) level with COSMO solvation in toluene.

These ^31^P{^1^H} NMR studies support the suggestion that the heterocycles may indeed bind to the gold cation. DFT calculations allowed for the relative binding affinity of [Au(PPh_3_)]^+^ toward indole **5** and skatole **31** when compared to the alkyne **13** to be evaluated (Scheme 3). Binding of the [Au(PPh_3_)]^+^ to the five-membered ring of **5** and **31** was successfully modeled as states **C**_**5**_ and **C**_**31**_ respectively. These were taken as the reference states for the calculations with the addition of one molecule of ynone **13**. Coordination of the [Au(PPh_3_)]^+^ to the carbonyl group of the ynone is endergonic (+12 and +8 kJ mol^–1^ for indole and skatole, respectively), whereas η^2^(π)-coordination of the alkyne lies at +18 and +14 kJ mol^–1^ for indole and skatole, respectively. This is a remarkable prediction as the prevailing mechanistic view of gold-catalyzed reactions of this type is that the metal activates the alkyne toward nucleophilic addition via η^2^(π)-coordination of the alkyne.

**Scheme 4:**
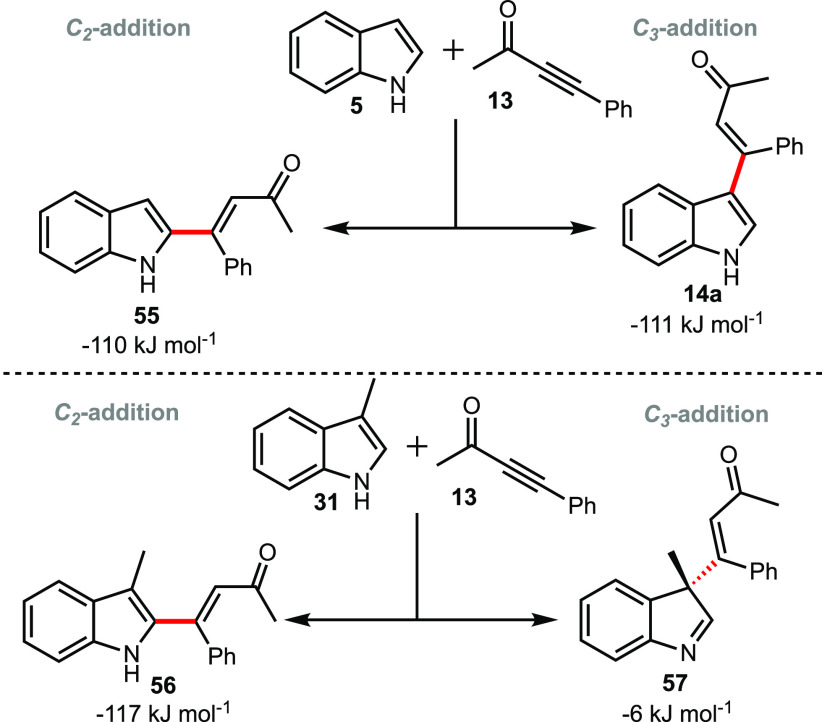
DFT-Calculated Changes in Energy for Products Arising from *C*_*2*_- and *C*_*3*_-Addition to Indole and Skatole Energies are Gibbs energies at 298.15 K at the D3(BJ)-PBE0/def2-TZVPP//BP86/SV(P) level with COSMO solvation in toluene.

The pathways controlling the formation of the vinyl indoles were investigated next. In the first instance, the thermodynamic preference for *C*_*2*_- and *C*_*3*_-addition of the alkyne to indole and skatole were calculated. (Scheme 4). For indole, the experimentally observed *C*_*3*_-addition to produce **14** is exergonic by −111 kJ mol^–1^: the thermodynamic driving force for *C*_*2*_-addition to produce **55** is essentially identical (−110 kJ mol^–1^). The *C*_*3*_-regioselectivy of the reaction between **5** and **13** is therefore predicted to be kinetic in nature. In the skatole case, *C*_*3*_-addition will produce **57**, which is the intermolecular analogue of the spirocycle **4** (Scheme 1). As a consequence of the methyl group at the site of substitution, **57** is not able to rearomatize, which is reflected in the fact it lies at only −6 kJ mol^–1^ with respect to **31** and **13**. The *C*_*2*_-addition product, **56**, which has rearomatized following a formal proton migration, is exergonic by −117 kJ mol^–1^, broadly in line with the addition to indole **5**.

With these data strongly indicating that the *C*_*3*_-selectivity for the reaction is kinetic in nature, transition states for C–C bond formation were sought to support this using DFT. States for the addition of the indole to a gold-coordinated alkyne, **B**, could be readily located; however, the corresponding pathways associated with the addition of the ynone to a coordinated indole (**C**_**5**_) could not be found. A pathway for the addition of indole to the *O*-coordinated alkyne, **A**, was located, but at much higher energy than the pathway via **B** (see Supporting Information). These data support the supposition that the reaction proceeds via addition of indole to an η^2^(π) gold-coordinated ynone, despite the heterocycle being a better ligand for the metal.

**Figure 5:**
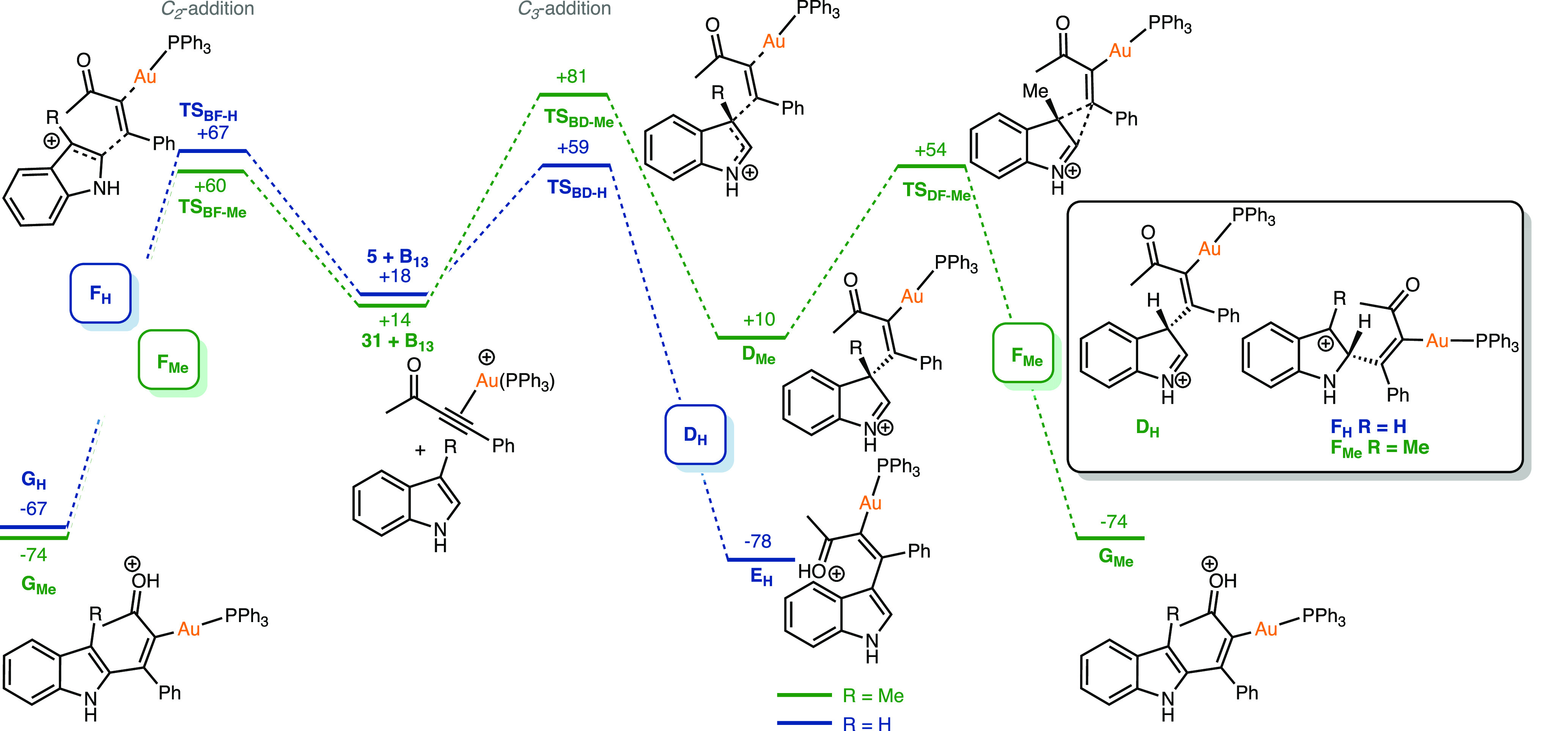
DFT-calculated pathways for the addition of **5** or **31** to gold-coordinated alkyne complex **B**_**13**_. Energies are Gibbs energies in kJ mol^–1^ at 298.15 K at the D3(BJ)-PBE0/def2-TZVPP//BP86/SV(P) level with COSMO solvation in toluene.

Transition states for *C*_*3*_- and *C*_*2*_-addition of **5** to **B** were located (**TS**_**BD-H**_ and **TS**_**BF-H**_) at +59 and +67 kJ mol^–1^, respectively (Figure 5). Although the difference in energy is small, *C*_*3*_-addition through **TS**_**CD-H**_ was found to be the lower energy pathway at all levels of theory employed (see Supporting Information). A Dynamic Reaction Coordinate (DRC) analysis indicated that **TS**_**BD-H**_ connected state **B**_**H**_ with Wheland-type intermediate **D**_**H**_. Attempts to optimize the geometry of **D**_**H**_ resulted in **E**_**H**_ in which a hydrogen atom had migrated to the oxygen of the ynone, resulting in rearomatization of the indole. This may indicate that **D**_**H**_ sits in a shallow minimum and, similarly, attempts to optimize **F**_**H**_ (which DRC predicts connects to **B** via **TS**_**BF-H**_) resulted in **G**_**H**_.

For skatole **31** the kinetic preference for the site of attack was reversed. Here, *C*_*2*_-addition via **TS**_**BF-Me**_ lies at +60 kJ mol^–1^, whereas **TS**_**BE-Me**_ was located at +81 kJ mol^–1^. Again, attempts to optimize **F**_**Me**_ resulted in **G**_**Me**_. However, in the case of *C*_*3*_-addition via **TS**_**BD-Me**_ it was possible to optimize the corresponding states **D**_**Me**_ (+10 kJ mol^–1^), presumably as hydrogen migration is not possible in this Wheland intermediate. Instead, a transition state for vinyl migration was located (**TS**_**DF-Me**_) at +54 kJ mol^–1^. This leads to **G**_**Me**_, again presumably via **F**_**Me**_. A transition state for methyl migration was also obtained, but this was at higher energy (see Supporting Information). These data therefore indicate that **both***C*_*2*_- and *C*_*3*_- addition pathways for addition to skatole will lead to **32**, although the former route is kinetically preferred.

The data also provide an explanation for the difference in reactivity between the intra- and intermolecular systems. There is clearly a greater entropic penalty in the case of the intermolecular coupling reaction; however, the stronger binding of the gold cation to the indole when compared to the alkyne will also inhibit access to the product-forming pathways. Binding to the indole is still possible in the case of the intramolecular pathway; however, the preorganized structure of the substrate may enable low energy π-slippage events^46−48^ leading to alkyne coordination without the necessity for loss of the metal. In the intermolecular case, this is not possible and decoordination of the gold followed by re-coordination in the thermodynamically less preferred binding mode is required.

**Scheme 5:**
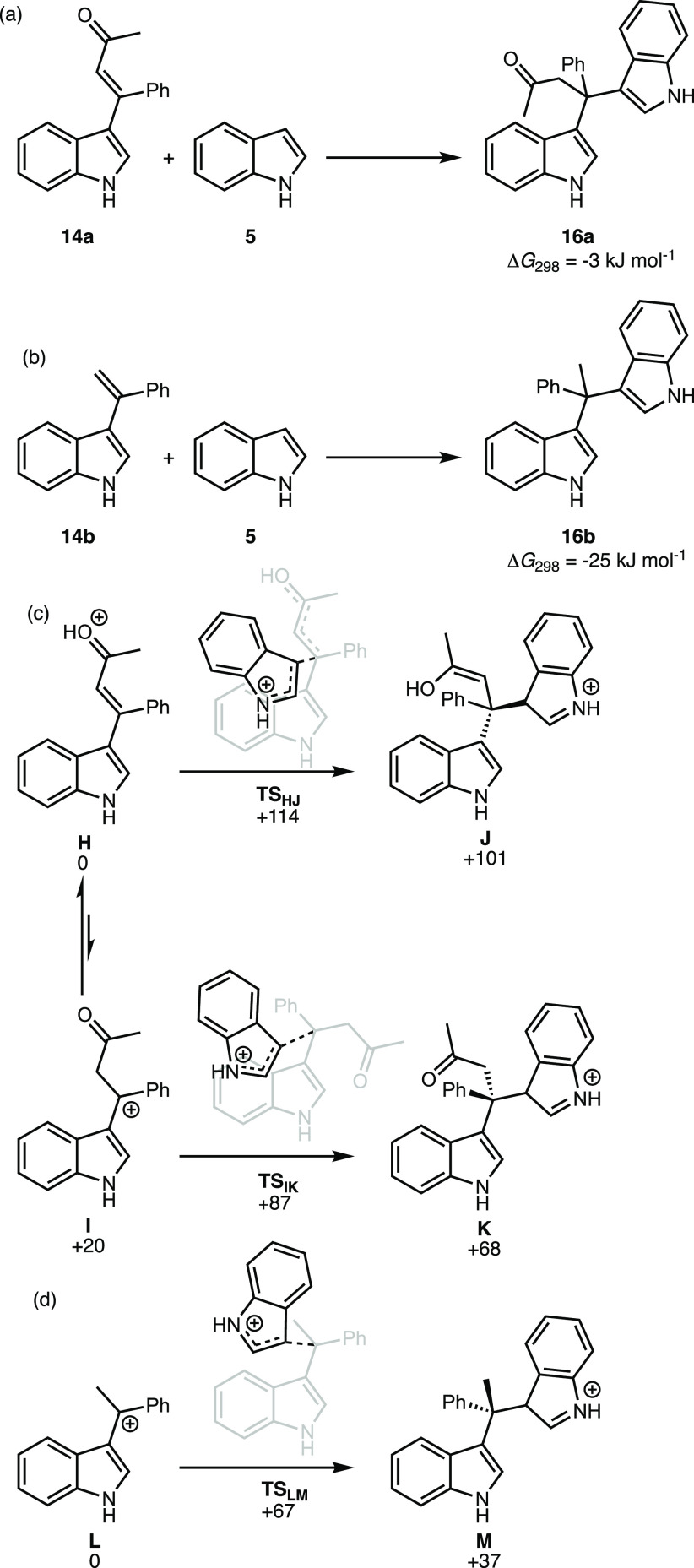
DFT-Calculated Pathways for the Acid Catalyst Addition to Vinylindole **14** (a) and the Corresponding Species Derived from Phenylacetylene (b) Energies are Gibbs energies at 298.15 K (in kJ mol^−1^ for (c) and (d)) at the D3(BJ)-PBE0/def2-TZVPP//BP86/SV(P) level with COSMO solvation in CH_2_Cl_2_.

With the origin of the site-selectivity of the reaction established, the factors controlling single versus double addition of the indole to alkynes were then investigated. In the case of the system derived from the ynone substrate, the relative thermodynamic free energy change for the formation of **16a** from **14a** and **5** (Scheme 5a) was calculated to be −3 kJ mol^–1^. In the case of the corresponding system based on phenylacetylene, the formation of **16b** from **15b** and **5** was found to have much greater change in free energy (Δ*G*_298_ = −25 kJ mol^–1^, Scheme 5b). These data indicate that in the case of the ynone system there is, at best, only a very small thermodynamic driving force for the addition of a second indole to **14a**.

The kinetic factors controlling this difference in reactivity between the two systems were also investigated. As previous experimental and computational work had established that the addition of a second indole molecule to vinyl indoles was acid-catalyzed,^33,34^ analogous processes were calculated for the addition of a second molecule of indole to vinyl indole **14** (Scheme 5c). Protonation of **14** could be envisaged to occur at either the carbonyl groups to give **H** or the alkene to give **I**. Cation **H**, which was taken as the reference state for this series of calculations, was found to be 20 kJ mol^–1^ more stable than **I**. Transition states for the addition of indole to both **H** and **I** (**TS**_**HJ**_ and **TS**_**IK**_) were located at +114 and +87 kJ mol^–1^ respectively. The transition state for the analogous phenylacetylene-derived product (**TS**_**LM,**_Scheme 5d) lies at +67 kJ mol^–1^ with respect to the cation precursor and the coupled product **M** lies at lower energy than those derived from **14** (+101 and +68 kJ mol^–1^ for **J** and **K**, respectively)

The precise putative pathway for the addition of the second indole in the ynone system will depend on the relative rate of proton transfer between **H** and **I** (if this is rapid and an equilibrium concentration of **I** is present, then the energic span will be 87 kJ mol^–1^, otherwise it will be 114 kJ mol^–1^). However, it is evident the addition of a second indole to either cation of the ynone-derived system has a higher barrier than for the phenyl-acetylene derivative. In the ynone case, the resulting cationic intermediates **J** and **K** lie at a significantly higher relative energy than in the phenylacetylene case, **M**.

At the start of this study, we postulated that the introduction of the carbonyl group on the alkynes might reduce the proclivity of the vinylindole product to undergo additional reactions with indole, and this notion was borne out in the synthetic reactions featured in Scheme 2. Further, it is supported by the DFT data, with the carbonyl-based system facing significantly higher kinetic barriers for the addition of a second indole compared to the analogous simple alkyne system, and there is a negligible thermodynamic driving force for this process.

### Identification of a Novel Gold-Coordinated Pyrylium Salt from Alkyne Coupling

**Scheme 6:**
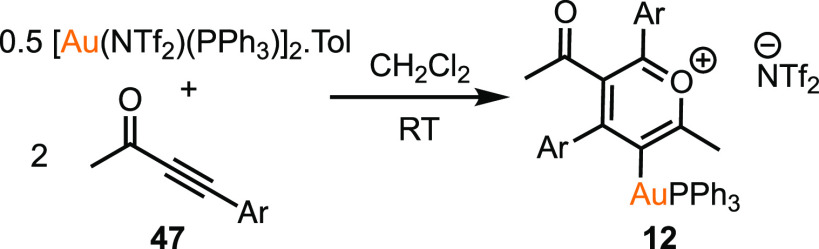
Formation of Pyrylium Salt **12** Ar = C_6_H_4_-4-NMe_2_.

The NMR studies designed to investigate the interaction of substituted ynones with [Au(NTf_2_)(PPh_3_)]_2_·Tol had demonstrated that a selective reaction occurred when **47** was employed. Specifically, the addition of 2 equiv of **47** to a CD_2_Cl_2_ solution of Au^I^ complex [Au(NTf_2_)(PPh_3_)]_2_·Tol resulted in an immediate change in color to deep red and the formation of **12**, as shown by the resonance in the ^31^P{^1^H} NMR spectrum at δ_p_ 41.9. Although all attempts to isolate **12** from the reaction mixture were unsuccessful, a combination of spectroscopic methods demonstrated that the product was a gold-substituted pyrylium salt,^49^ arising from the dimerization of two ynones (Scheme 6).

**Figure 6:**
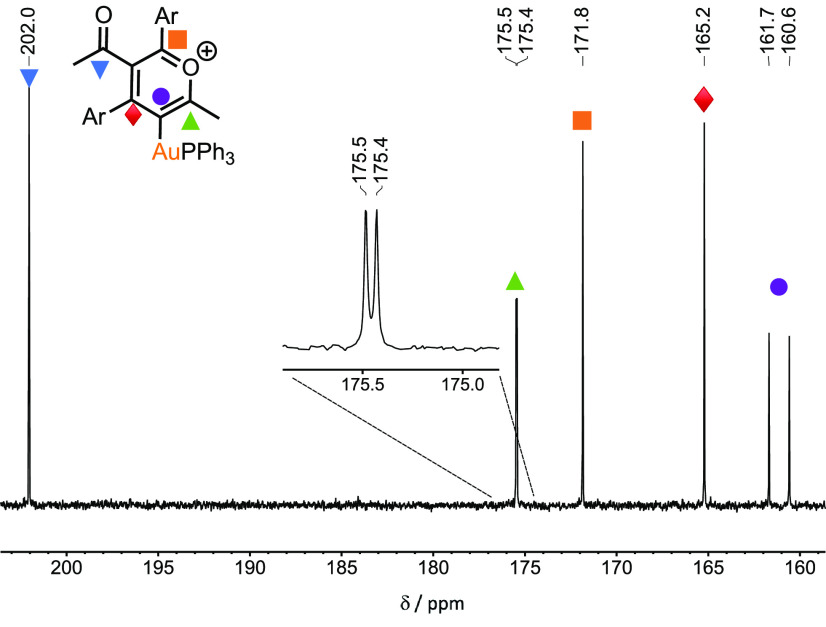
Expansion of a ^13^C{^1^H} NMR spectrum of **12** in CD_2_Cl_2_ solution. Ar = C_6_H_4_-4-NMe_2_.

The ESI mass spectrum of the solution exhibited a peak at *m*/*z* = 833.2579 consistent with **12** having the composition [Au(**47**)_2_(PPh_3_)]^+^; that is, 2 equiv of the alkyne had been incorporated into the coordination sphere of the metal. The ^13^C{^1^H} NMR spectrum (Figure 6) exhibited a series of resonances consistent with the formulation of **12** as a pyrylium complex. For example, a doublet resonance at δ 161.4 (^2^*J*_PC_ = 111.2 Hz) is consistent with a gold-bound carbon atom and compares favorably with the corresponding resonance in [Au(C_6_H_2_-2,4,6-Me_3_)(PPh_3_)] (δ 169.8, ^2^*J*_PC_ = 111.2 Hz).^50^ A doublet resonance at δ 175.5 (^3^*J*_PC_ = 5.4 Hz) and singlets at δ 171.8 and 165.2 were observed whose chemical shifts are characteristic of carbon atoms in the 2, 4, and 6 positions of a pyrylium salt. Two resonances for the NMe_2_ groups were observed, indicating that two ynones had been incorporated into **12** but in different environments. Moreover, only a single resonance was observed in the carbonyl region at δ 202.0, indicating that one acyl group had been modified significantly during the reaction, which is again consistent with pyrylium formation.

The gold-mediated intramolecular coupling of alkynes is a common reaction pathway. However, corresponding intermolecular routes are scarce.^51−53^ Establishing the mechanistic pathways that lead to **12** is therefore important in understanding this unusual product formation. The initial dimerization step was anticipated to occur through nucleophilic attack at an η^2^(π)-coordinated complex of ynone **47**, by either the carbonyl (*O*-attack) or C≡C group (*C*-attack) of another molecule of ynone **47**.

**Figure 7:**
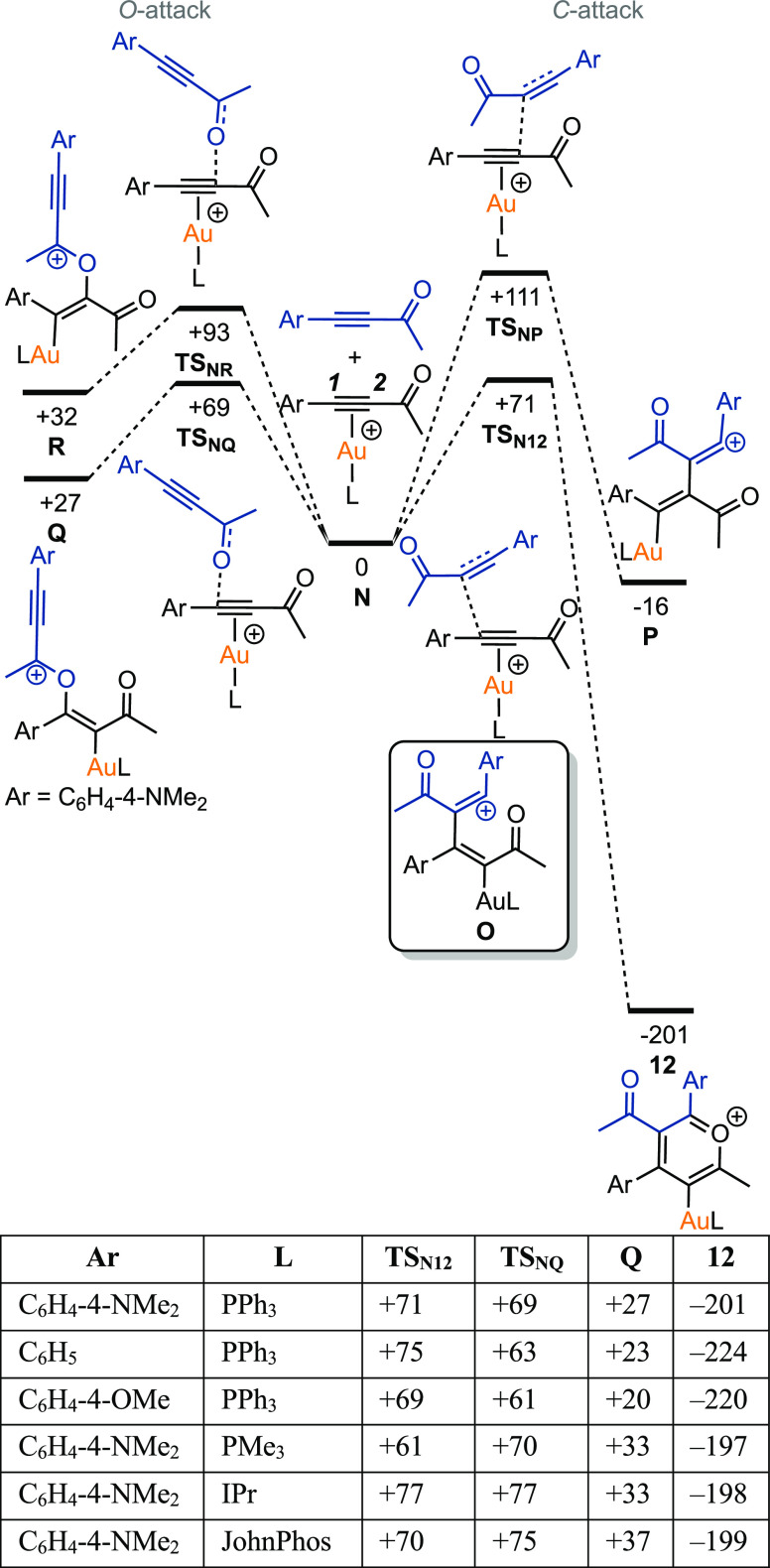
DFT-calculated pathways for the gold-mediated dimerization of alkynes. All energies are Gibbs energies at 298.15 K in kJ mol^−1^ at the D3(BJ)-PBE0/def2-TZVPP//BP86/SV(P) level with COSMO solvation in CH_2_Cl_2_.

DFT calculations were used to distinguish between these possibilities and the results are shown in Figure 7. The alkyne complex **B**_**47**_ and a molecule of ynone **47** was taken as the reference state for the calculations, collectively referred to as **N**. Considering first *C*-attack, transition state **TS**_**N12**_ (located at +71 kJ mol^–1^) involves addition of the carbonyl-substituted carbon of **47** onto the aryl-substituted carbon (***C1***) of the gold-coordinated alkyne. Attempts to optimize structure **O** (the expected state arising from **TS**_**N12**_) were unsuccessful: in all cases, pyrylium complex **12** was obtained. This may indicate that **O** either sits in a very shallow minimum or that **TS**_**N12**_ is a bifurcated transition state proceeding directly to **12**. The transition state for *C*-attack at ***C2*** proceeds through **TS**_**NP**_, which is at much higher energy (+111 kJ mol^–1^) than **TS**_**N12**_ so is uncompetitive.

The alternative *O*-attack by **47** onto ***C1*** of **B**_**47**_ proceeds to give alkenyl complex **Q** through **TS**_**NQ**_ (+69 kJ mol^–1^), which lies at a similar energy to **TS**_**N12**_ (+71 kJ mol^–1^). As with the *C*-attack pathway, addition to ***C2*** lies at considerably higher energy in the *O*-attack case (**TS**_**NR**_ + 93 kJ mol^–1^) and therefore can be discounted. On the basis of the energies of **TS**_**NQ**_ and **TS**_**N12**_ it is likely that both *O-* and *C*-attack pathways operate, but it appears that pyrylium complex formation can only take place via *C*-attack, as all attempts to find a pathway by which **Q** might itself rearrange into **12** were unsuccessful. In this case, it is likely that **Q** is an off-cycle complex in equilibrium with the starting materials **N**, and because **Q** lies at higher energy than the reference state (and **12** much lower), the irreversible formation of **12** through **TS**_**N12**_ predominates.

We were also interested to learn why in the synthetic NMR experiments described earlier (Figure 3), selective pyrylium formation was only observed when using electron rich ynone **47**. Therefore, the calculations were repeated for the two lowest energy *O*- and *C*- attack pathways to evaluate the effects of different substituents on the ynone and the ligand on gold (Figure 7). Overall, the ligand effects are small, but the most notable observation is that in the case of the C_6_H_5_-substituted ynone **13**, the corresponding transition state leading to the pyrylium complex **TS**_**N12**_, lies at 12 kJ mol^–1^ higher in energy than the *O*-attack pathway. This need not rule out selective pyrylium formation if the reaction remains under thermodynamic control, but side reactions resulting from **Q** are also possible. For example, **Q** could potentially be converted into to a cyclic complex, via a mechanism related to that observed in the gold-catalyzed 1,3-*O*-transposition of ynones (see Supporting Information for details).^54^ Thus, the experimental observation that the reaction of C_6_H_5_-substituted ynone **13** is much less selective than it is for the C_6_H_4_-4-NMe_2_-substituted case is likely a consequence of an increased kinetic preference for **Q** resulting in more side-reactions.

## Conclusions

The use of carbonyl-substituted alkynes as coupling partners in the gold-catalyzed coupling with indoles has unlocked a well-controlled pathway enabling selective monovinylation to give vinyl indoles. DFT calculations reveal that there is both a kinetic and thermodynamic component to the observed selectivity, with the carbonyl group destabilizing the intermediate carbocations and related transition states for C–C addition when compared to more conventional substrates. It is clear that the outcome of the reaction is controlled by the nature of the alkyne substrate, with control reactions with phenylacetylene giving the expected *bis*indolemethane.

To account for the difference between the intra-^3,5,7,8^ and intermolecular variants of the coupling reaction, there is presumably an entropic component, but also important is the somewhat surprising observation that the gold catalyst shows a thermodynamic preference for binding to the indole, rather than the alkyne. This may also explain the lack of activity of other Lewis acid catalysts in the intermolecular reactions as it is not unreasonable to expect that the most potent nucleophile in the reaction is also the best ligand for the metal.

The observation of a product arising from the coupling of two alkynes within the coordination sphere of gold is remarkable. Although related pyrylium salts have been frequently proposed in the gold-catalyzed intramolecular coupling of alkynes with tethered carbonyl groups,^55,56^ and may be isolated,^57^ the observation of **12** is a unusual example of an intermolecular addition and may offer the potential for future synthetic findings.

## Supporting Information Available

Structure data (XYZ)
Experimental details and spectroscopic data for the compounds prepared in this work; Details of the computational methodology employed, an evaluation of anion effects, and collated energies and coordinates (PDF)

## Supplementary Material

om2c00035_si_001.xyz

## Accession Codes

CCDC 2116351 contains the supplementary crystallographic data for this paper. These data can be obtained free of charge via www.ccdc.cam.ac.uk/data_request/cif, or by emailing data_request@ccdc.cam.ac.uk, or by contacting The Cambridge Crystallographic Data Centre, 12 Union Road, Cambridge CB2 1EZ, UK; fax: +44 1223 336033.
