# Supplementary material for: Selectivity, Speciation, and Substrate Control in the Gold-Catalyzed Coupling of Indoles and Alkynes
Source: Organometallics. 2022 Feb 10;41(4):497–507. doi: 10.1021/acs.organomet.2c00035 (PMC9007570; doi:10.1021/acs.organomet.2c00035)
Supplement: Supplementary file 2 — om2c00035_si_002.pdf [file om2c00035_si_002.pdf]

Electronic Supporting Information for

Selectivity, speciation and substrate control in the gold-catalyzed coupling of indoles and alkynes

Ryan G. Epton, William P. Unsworth* and Jason M. Lynam*

Department of Chemistry, University of York, Heslington, York, UK

e-mail: william.unsworth@york.ac.uk; jason.lynam@york.ac.uk

Table of Contents

Reaction Optimisation S3

Stereochemistry Assignment S5

NOESY of 4-(4-(dimethylamino)phenyl)-4-(1H-indol-3-yl)but-3-en-2-one (30) S6

NOESY of 3-(1*H*-indol-3-yl)-3-(4-methoxyphenyl)-*N*,*N*-dimethylacrylamide (45) S7

XRD Crystal Structure of 4-(1-methyl-3-phenyl-indol-2-yl)-4-phenylbut-3-en-2-one (37) S8

Experimental Data S10

General Information S10

General Procedures S11

Starting Material Synthesis S12

Vinylated-indole Products S15

Gold-pyrylium Complex S42

NMR Spectra S44

DFT Calculations S76

Computational Method S76

Energies of C2 and C3 Indole Addition at Different Levels of Theory S77

Interaction of the Triflimide Anion with Cationic Gold Complexes. S78

Energies S80

Indoles S80

Alkynes S81

Vinylated Indole Products S82

O-coordinated Gold Complexes S83

Alkyne-coordinated Gold Complexes S85

Indole-coordinated Gold Complexes S87

Indole-vinylation Pathway S88

Indole-vinylation via *O*-coordinated alkyne S89

Skatole-vinylation Pathway S90

Indole Addition to Vinylated-indoles S92

Pyrylium Complex Formation S95

Triflimide-containing structures S106

Gold-catalysed 1,3-*O*-transposition of Ynones S110

References S111

Reaction Optimisation

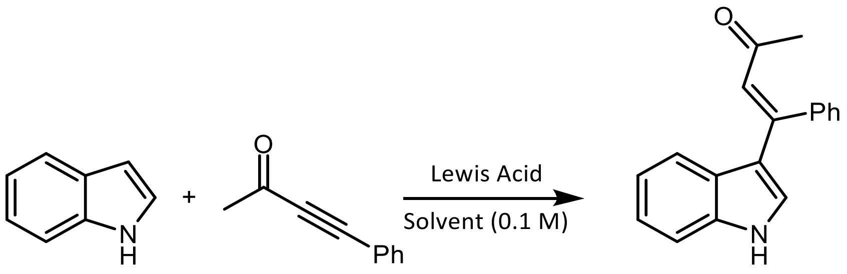

5 **13** **14a**

Indole (12 mg, 0.1 mmol) was dissolved in the desired solvent (1 mL, 0.1 M), and the desired quantity of 4-phenyl-3-butyne-2-one was added, followed by the chosen Lewis acid catalyst. The reaction was stirred at the chosen temperature for the given amount of time before the solvent was concentrated *in vacuo*. ^{1}H NMR spectra were then recorded to determine the conversion of indole to the desired product (Table S1).

Table S1 - Optimisation of the intermolecular reaction of indole **5** with ynone **13**. [a] Conversion determined by ratio of remaining indole to both isomers of **14a** by ^{1}H NMR spectroscopy. [b] 10 mol%. [c] 5 mol%.

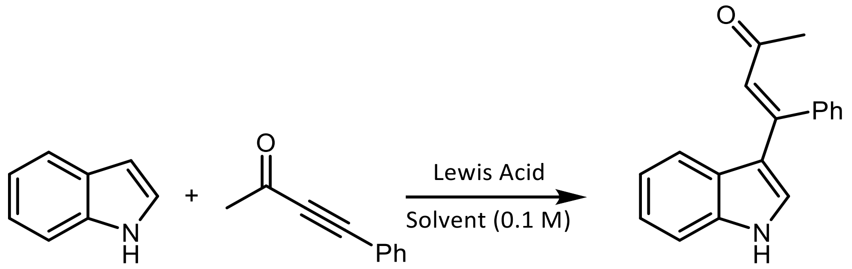

5 **13** **14**

Entry	Catalyst	Solvent	Temp / °C	Time / hr	Equiv. of 13	Conversion[a] (*E*/*Z*) / %
1	AgOTf [b]	Toluene	RT	24	1	0
2	AgOTf [b]	Toluene	40	24	1	0
3	$Cu(OTf)_2$ [b]	Toluene	RT	24	1	0
4	$Cu(OTf)_2$ [b]	Toluene	40	24	1	0
5	$SnCl_2.2H_2O$ [b]	Toluene	RT	24	1	0
6	$SnCl_2.2H_2O$ [b]	Toluene	40	24	1	0
7	$[Au(NTf_2)(PPh_3)]_2$.Tol [c]	Toluene	RT	24	1	90 (73:27)
8	$[Au(NTf_2)(PPh_3)]_2$.Tol [c]	Toluene	RT	4	1	55 (72:28)
9	$[Au(NTf_2)(PPh_3)]_2$.Tol [c]	DCM	RT	4	1	57 (73:27)
10	$[Au(NTf_2)(PPh_3)]_2$.Tol [c]	MeCN	RT	4	1	45 (72:28)
11	$[Au(NTf_2)(PPh_3)]_2$.Tol [c]	EtOH	RT	4	1	17 (73:27)
12	$[Au(NTf_2)(PPh_3)]_2$.Tol [c]	Toluene	RT	4	1	55 (72:28)
13	$[Au(NTf_2)(PPh_3)]_2$.Tol [c]	Toluene	RT	24	1	90 (73:27)
14	$[Au(NTf_2)(PPh_3)]_2$.Tol [c]	Toluene	40	4	1	82 (73:27)
15	$[Au(NTf_2)(PPh_3)]_2$.Tol [c]	Toluene	40	24	1	88 (72:28)
16	$[Au(NTf_2)(PPh_3)]_2$.Tol [c]	Toluene	40	2	1	91 (73:27)
17	$[Au(NTf_2)(PPh_3)]_2$.Tol [c]	Toluene	40	2	1.2	94 (72:28)
**18**	**$[Au(NTf_2)(PPh_3)]_2$.Tol** [c]	**Toluene**	**40**	**2**	**1.5**	**100 (71:29)**
19	$[Au(NTf_2)(PPh_3)]_2$.Tol [c]	Toluene	40	2	2	100 (71:29)

Stereochemistry Assignment

In order to assign the *E* and *Z* isomers, 2D NOESY spectra were recorded using a Bruker AVIIIHD 500 MHz spectrometer. Godoi *et al.* previously used a method to assign the stereochemistry of methyl 3-(1*H*-indol-3-yl)-3-phenylacrylate **40**,[1] in which the key interaction between the C-4 proton on indole, and the vinyl proton (Figure S1), was assigned to the *E* isomer depicted. This interaction was therefore used primarily in our assignment of 4-(4-(dimethylamino)phenyl)-4-(1*H*-indol-3-yl)but-3-en-2-one **30** (Figure S2) and 3-(1*H*-indol-3-yl)-3-(4-methoxyphenyl)-*N*,*N*-dimethylacrylamide **45** (Figure S3).

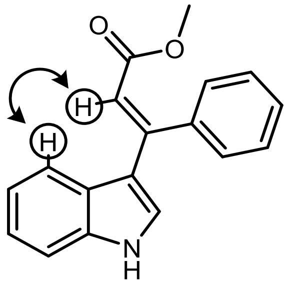

(*E*)-40

Figure S1 - Structure of **(*E*)-40**, with key stereochemical assignment, as reported by Godoi *et al.*

NOESY of 4-(4-(dimethylamino)phenyl)-4-(1H-indol-3-yl)but-3-en-2-one (30)

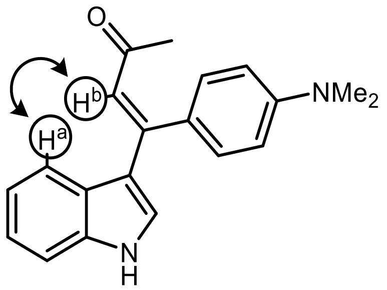

(*E*)-30

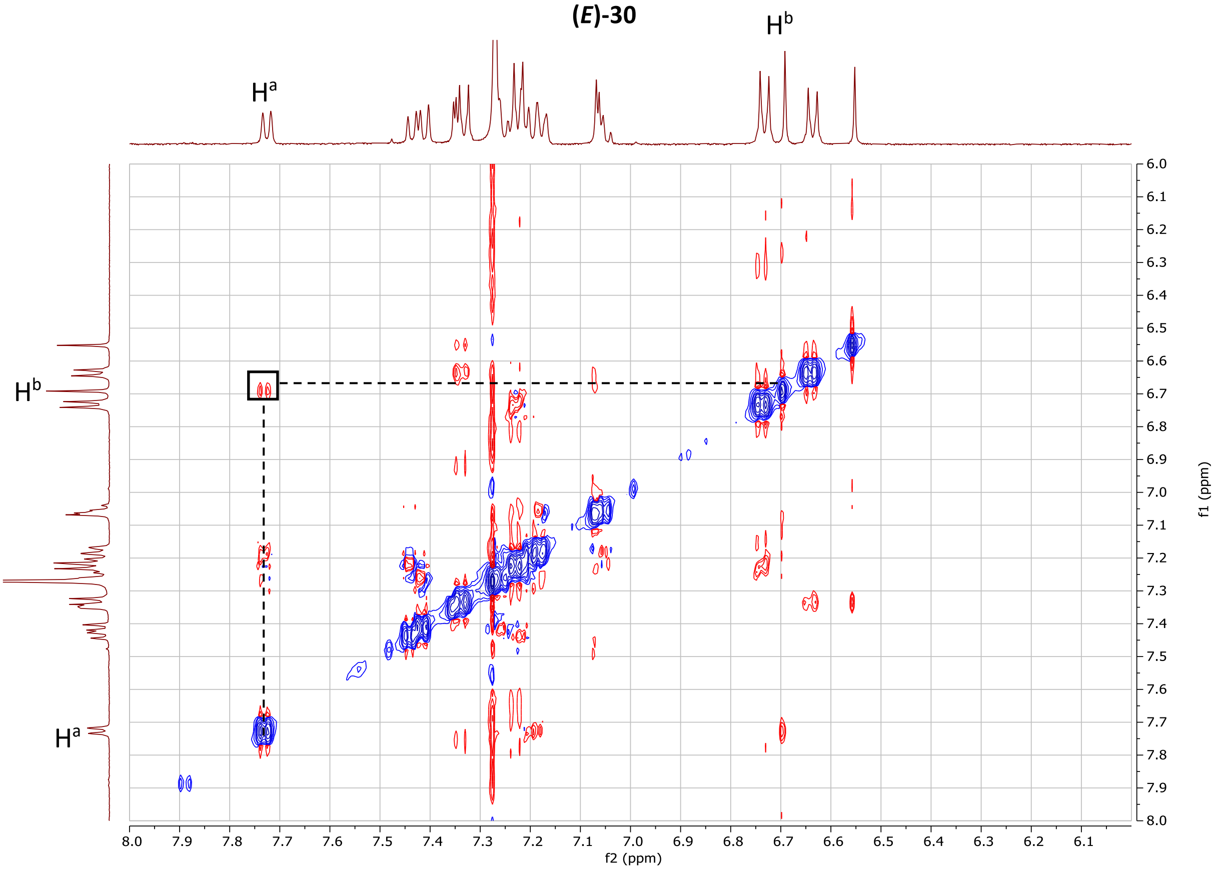

Figure S2 - NOESY spectrum of **30** recorded at 500 MHz in $CDCl_3$. Parameters - D1 = 2 s, D8 = 0.5 s.

NOESY of 3-(1*H*-indol-3-yl)-3-(4-methoxyphenyl)-*N*,*N*-dimethylacrylamide (45)

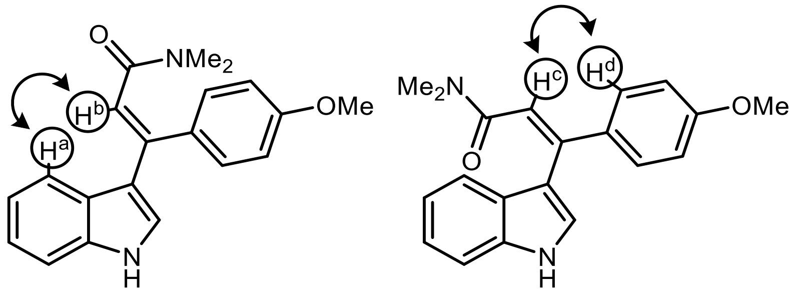

(*E*)-45 **(*Z*)-45**

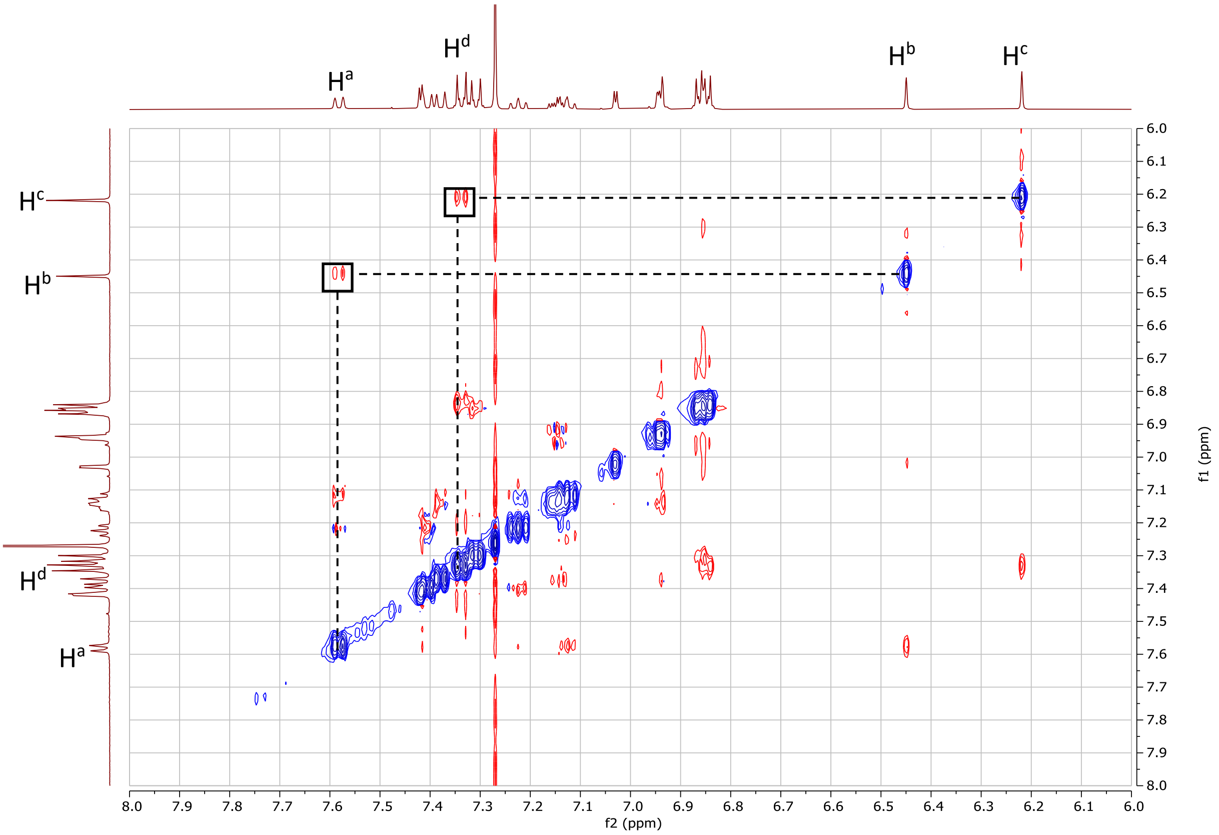

Figure S3 - NOESY spectrum of **45** recorded at 500 MHz in $CDCl_3$. Parameters - D1 = 2 s, D8 = 0.5 s.

XRD Crystal Structure of 4-(1-methyl-3-phenyl-indol-2-yl)-4-phenylbut-3-en-2-one (37)

The structure and stereochemistry of compound **37** were confirmed by X-ray crystallography. Supplementary crystallography data can be downloaded from:

www.ccdc.cam.ac.uk/conts/retrieving.html.

Single crystals of $C_{25}H_{21}NO$, **37**, were crystallised from dichloromethane layered with hexane. A suitable crystal was selected and mounted in oil on a 200 micrometre LithoLoop on a SuperNova, Dual source diffractometer. Cu-K_{α} radiation was used for the data collection. The crystal was kept at 109.95(10) K during data collection. Using Olex2,[2] the structure was solved with the SHELXT[3] structure solution program using Intrinsic Phasing and refined with the SHELXL[4] refinement package using Least Squares minimisation.

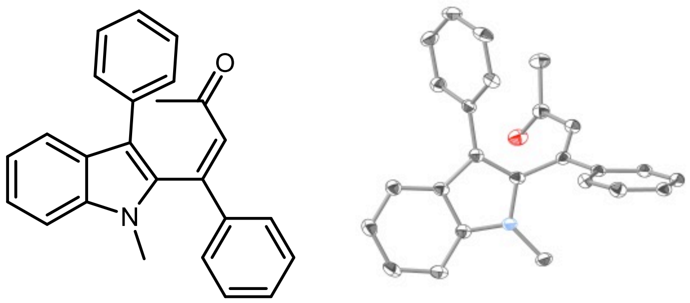

37 CCDC 2116351

Table S2 Crystal data and structure refinement for CCDC 2116351.

Empirical formula	$C_{25}H_{21}NO$
Formula weight	351.43
Temperature/K	109.95(10)
Crystal system	triclinic
Space group	P-1
a/Å	9.7468(7)
b/Å	10.0795(7)
c/Å	10.7670(7)
α/°	115.484(7)
β/°	93.470(6)
γ/°	101.134(6)
Volume/Å^3	924.50(12)
Z	2
ρ_{calc}g/cm^3	1.262
μ/mm^{-1}	0.592
F(000)	372.0
Crystal size/mm^3	0.376 × 0.147 × 0.057
Radiation	Cu Kα (λ = 1.54184)
2Θ range for data collection/°	9.22 to 134.13
Index ranges	$-11 \leq h \leq 11$, $-12 \leq k \leq 9$, $-12 \leq l \leq 12$
Reflections collected	6953
Independent reflections	3293 [R_{int} = 0.0189, R_{sigma} = 0.0258]
Data/restraints/parameters	3293/0/329
Goodness-of-fit on F^2	1.033
Final R indexes [I>=2σ (I)]	R_1 = 0.0332, wR_2 = 0.0812
Final R indexes [all data]	R_1 = 0.0396, wR_2 = 0.0859
Largest diff. peak/hole / e Å^{-3}	0.21/-0.18

Experimental Data
General Information

Except where stated, all reagents were purchased from commercial sources and used without further purification. Anhydrous THF was obtained from an Innovative Technology Inc. PureSolv® solvent purification system. Anhydrous *d*2-DCM was dried over calcium hydride overnight before being distilled via a Schlenk line. ^{1}H NMR, ^{13}C NMR, ^{19}F NMR and ^{31}P NMR spectra were recorded on a JEOL ECX400 or JEOL ECS400 spectrometer, operating at 400 MHz, 100 MHz, 376 MHz and 162 MHz respectively, or on a Bruker AVIIIHD 600 Widebore spectrometer operating at 600 MHz, 150 MHz, 565 MHz and 243 MHz respectively. All spectral data was acquired at 298 K. Chemical shifts (δ) are quoted in parts per million (ppm). The residual solvent peaks; δ_H 7.27 and δ_C 77.16 for $CDCl_3$, δ_H 5.32 and δ_C 53.84 for CD_2Cl_2 and δ_H 2.50 and δ_C 39.52 for *d*6-DMSO, were used as a reference. Coupling constants (J) are reported in Hertz (Hz) to the nearest 0.5 Hz. The multiplicity abbreviations used are: s singlet, d doublet, t triplet, q quartet, m multiplet, br s broad singlet. Signal assignment was achieved by analysis of DEPT, COSY, HMBC and HSQC experiments where required. Infrared (IR) spectra were recorded on a PerkinElmer UATR 2 spectrometer as a thin film dispersed from either CH_2Cl_2 or $CDCl_3$. Mass-spectra (low and high-resolution) were obtained by the University of York Mass Spectrometry Service, using electrospray ionisation (ESI) on a Bruker Daltonics, Micro-tof spectrometer. Melting points were determined using Gallenkamp apparatus. Thin layer chromatography was carried out on Merck silica gel 60F254 pre-coated aluminium foil sheets and were visualised using UV light (254 nm) and stained with either an acidic solution of vanillin in ethanol or a basic potassium permanganate solution. Flash column chromatography was carried out using slurry packed Fluka silica gel (SiO2), 35–70 µm, 60 Å, under a light positive pressure, eluting with the specified solvent system.

General Procedures

General Procedure A: Gold-catalysed alkyne addition to indole

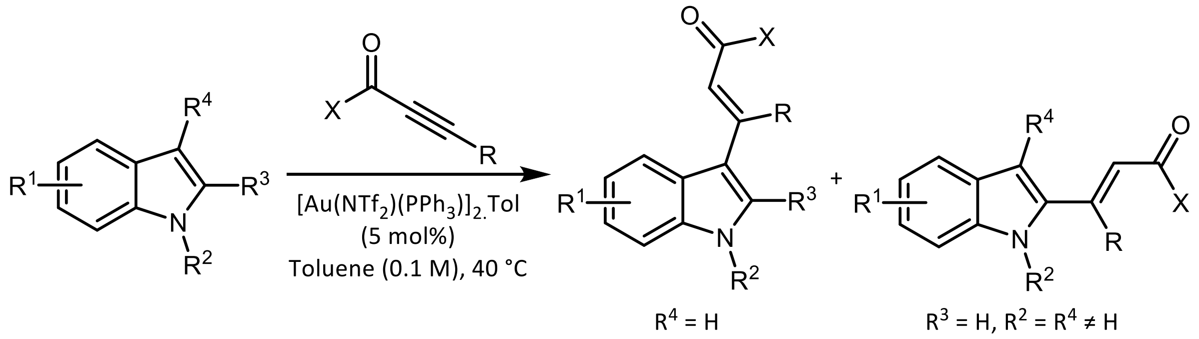

The indole substrate (1 equiv) and alkyne (1.5 equiv) were dissolved in toluene (0.1 M) and [bis(trifluoromethanesulfonyl)imidate](triphenylphosphine)gold(I) (2:1) toluene adduct (5 mol%) was added. The reaction was then stirred at 40 °C for the reported time. The reaction mixture was then concentrated *in vacuo* and the product was purified via flash column chromatography using the reported eluent.

General Procedure B: NMR Studies

To a sample vial, [bis(trifluoromethanesulfonyl)imidate](triphenylphosphine)gold(I) (2:1) toluene adduct (15.7 mg, 0.01 mmol) and the desired amount of substrate were dissolved in *d*2-DCM (0.6 mL). ^{1}H and $^{31}P\{^{1}H\}$ NMR spectra were then recorded using 16 and 128 scans respectively. For the amide and ester alkynes, **48** – **51**, anhydrous *d*2-DCM was used. Due to the dimeric nature of the gold catalyst, 1 equivalent of substrate = 0.02 mmol.

Starting Material Synthesis

The following starting materials have been described previously in the literature and the established published methods were used for their synthesis (Figure S4).[5-10]

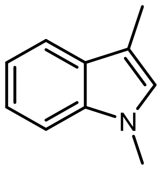

33 [5]

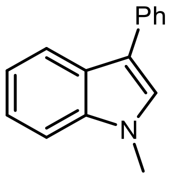

34 [6]

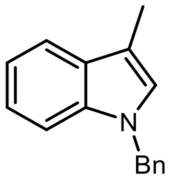

35 [7]

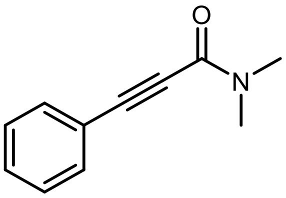

48 [8]

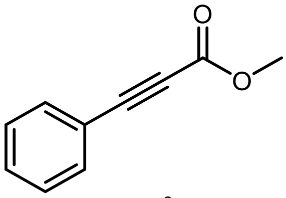

50 [9]

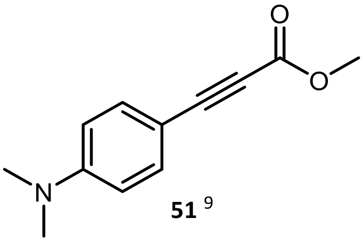

51 [9]

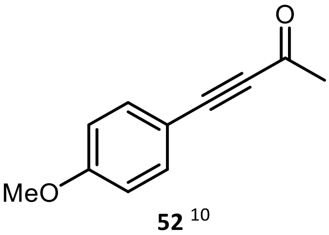

52 [10]

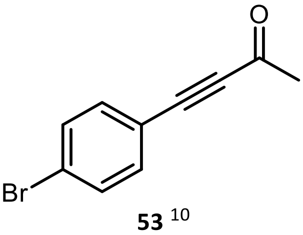

53 [10]

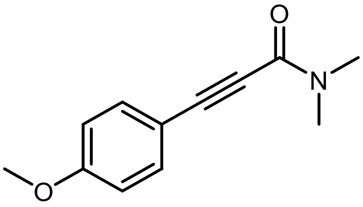

S1 [8]

Figure S4 - Starting materials synthesised using literature methods.

4-(4-(Dimethylamino)phenyl)but-3-yn-2-one (47)

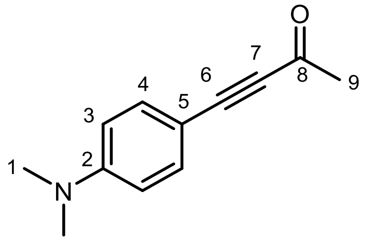

47

The synthesis was based on a literature procedure.[10] To a dry round-bottomed flask, 4-dimethylaminophenyl acetylene (436 mg, 3.00 mmol) was dissolved in THF (50 mL) under an atmosphere of Ar and cooled to 0 °C. With vigorous stirring, *n*-BuLi (1.32 mL, 2.5 M in hexane, 3.3 mmol) was added and stirred for 15 minutes, at which point the solution was cooled to –78 °C and *N*-methyl, *N*-methoxy acetamide (356 mg, 0.36 mL, 3.5 mmol) was added slowly over 3 minutes. The reaction was stirred for 30 minutes, before warming to room temperature and stirring for a further 2 hours. The reaction was quenched with aq. HCl (7.5 mL, 2 M) and extracted with DCM (3 x 50 mL). The organic extracts were combined, dried with $MgSO_4$ and concentrated *in vacuo*. The crude product was purified via flash column chromatography (hexane/ethyl acetate 8:2) to yield the *title product* as a pale, yellow solid (270 mg, 48%).

R_f (hexane/ethyl acetate 8:2) 0.36;

m.p. 97 – 98 °C;

ν_{max} (thin film)/cm^{-1} 2905, 2186, 2140, 1656, 816;

δ_H (400 MHz; $CDCl_3$) 2.41 (3 H, s, H-9), 3.02 (6 H, s, H-1), 6.59 – 6.64 (2 H, m, H-3), 7.42 – 7.46 (2 H, m, H-4);

δ_C (100 MHz; $CDCl_3$) 32.6 (C-9), 40.0 (C-1), 89.2 (C-7), 94.8 (C-6), 105.3 (C-5), 111.6 (C-3), 135.2 (C-4), 151.8 (C-2), 184.7 (C-8);

HRMS (ESI$^+$) Found: 188.1074; $C_{12}H_{14}NO$ (MH^+) Requires 188.1070 (−2.0 ppm error); Found: 210.0895; $C_{12}H_{13}NNaO$ (M+Na) Requires 210.0889 (−2.6 ppm error).

3-(4-(Dimethylamino)phenyl)-*N*,*N*-dimethylpropiolamide (49)

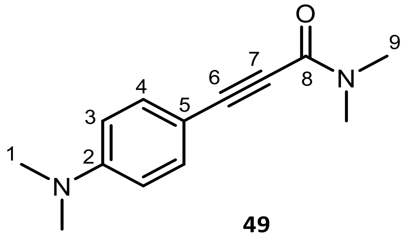

49

The synthesis was based on a literature procedure.[8] To a dry-round bottomed flask 4-ethynyl-*N*,*N*-dimethylaniline (290 mg, 2.0 mmol) was dissolved in dry THF (1.3 mL) under an atmosphere of argon and cooled to −78 °C. *n*-BuLi (1.0 mL, 2.5 mmol, 2.5 M in hexanes) was added and stirred for 15 minutes before dimethylcarbamoyl chloride (194 mg, 0.17 mL, 1.8 mmol) was added. The reaction was stirred for 5 minutes then warmed to room temperature and stirred for a further 1.5 hours. The reaction was quenched with deionised water (15 mL) and extracted with ethyl acetate (3 x 10 mL). The organic extracts were dried with anhydrous magnesium sulphate and concentrated *in vacuo* to yield the crude product. The product was purified via flash column chromatography (hexane/ethyl acetate 1:1) to yield the *title compound* as a pale, brown solid (339 mg, 87%).

R_f (1:1 hexane/ethyl acetate) 0.28;

mp 142 – 143 °C;

ν_{max} (thin film)/cm^{-1} 2908, 2199, 1608, 1526, 1366, 816;

δ_H (400 MHz; $CDCl_3$) 3.00 (6 H, s, H-1), 3.01 (3 H, s, H-9a), 3.28 (3 H, s, H-9b), 6.59 – 6.64 (2 H, m, H-3), 7.39 – 7.44 (2 H, m, H-4);

δ_C (100 MHz; $CDCl_3$) 34.2 (C-9a), 38.5 (C-9b), 40.1 (C-1), 80.8 (C-7), 92.8 (C-6), 106.6 (C-5), 111.6 (C-4), 134.0 (C-3), 151.2 (C-2), 155.5 (C-8);

HRMS (ESI$^+$) Found: 217.1337; $C_{13}H_{17}N_2O$ (MH^+) Requires 217.1335 (−0.9 ppm error); Found: 239.1157; $C_{13}H_{16}N_2NaO$ (M+Na) Requires 239.1155 (−1.0 ppm error).

Vinylated-indole Products

4-(1*H*-Indol-3-yl)-4-phenylbut-3-en-2-one (14a)

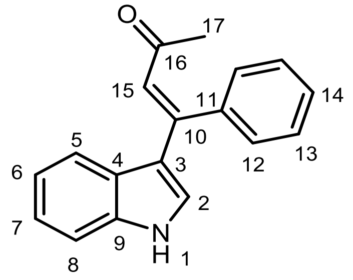

14a

Synthesised using general procedure A from indole (59 mg, 0.50 mmol), 4-phenyl-3-butyne-2-one (0.11 mL, 108 mg, 0.75 mmol) and [bis(trifluoromethanesulfonyl)imidate](triphenylphosphine)gold(I) (2:1) toluene adduct (40 mg, 0.03 mmol) in toluene (5 mL) for 2 hours at 40 °C. Purification by flash column chromatography (1:1 hexane/diethyl ether) afforded the *title product* as a yellow solid, as a mixture of *E* and *Z* stereoisomers (90 mg, 69%, *E*:*Z* = 74:26).

R_f (hexane/diethyl ether 1:1) 0.14;

mp 99–105 °C;

ν_{max} (thin film)/cm^{-1} 3267 (br), 3061, 2928, 1612, 1579, 1554, 1512, 1420, 742;

δ_H (400 MHz; $CDCl_3$) 1.88 (3 H, s, H-17, *E*), 1.99 (3 H, s, H-17, *Z*), 6.50 (1 H, s, H-15, *Z*), 6.79 (1 H, d, *J* = 3.0, H-2, *E*), 6.86 (1 H, s, H-15, *E*), 6.96 – 6.98 (2 H, m, H-Ar, *Z*), 7.11 – 7.24 (4 H, m, H-Ar, *E* and *Z*), 7.26 – 7.32 (5 H, m, H-Ar, *E* and *Z*), 7.32 – 7.40 (7 H, m, H-Ar, *E* and *Z*), 7.74 (1 H, d, *J* = 8.0, H-5, *E*), 8.96 (1 H, br s, H-1, *Z*), 9.15 (1 H, br s, H-1, *E*);

δ_C (100 MHz; $CDCl_3$) 30.0 (C-17, *Z*), 30.6 (C-17, *E*), 111.8 (CH, C-Ar, *Z*), 112.3 (CH, C-Ar, *E*), 114.2 (C-3, *Z*), 118.4 (C-3, *E*), 120.5 (CH, C-Ar, *Z*), 120.6 (CH, C-Ar, *Z*), 120.8 (CH, C-Ar, *E*), 121.4 (CH, C-Ar, *E*), 122.6 (CH, C-Ar, *Z*), 122.8 (C-15, *E*)l, 123.1 (CH, C-Ar, *E*), 125.0 (C-4, *E*), 126.4 (C-15, *Z*), 126.8 (C, C-Ar), 128.3 (CH, C-Ar), 128.4 (CH, C-Ar), 128.4 (CH, C-Ar), 128.5 (CH, C-Ar), 129.0 (CH, C-Ar), 129.4 (CH, C-Ar), 129.5 (CH, C-Ar), 130.6 (C-2, *E*), 136.4 (C, C-Ar), 137.5 (C-9, *E*), 140.3 (C-10, *E*), 141.6 (C-10, *Z*), 148.9 (C-11, *Z*), 151.7 (C-11, *E*), 200.6 (C-16, *E*), 201.1 (C-16, *Z*);

HRMS (ESI$^+$) Found: 262.1228; $C_{18}H_{16}NO$ (M+H$^+$) Requires 262.1226 (−0.8 ppm error); Found: 284.1049; $C_{18}H_{15}NNaO$ (M+Na) Requires 284.1046 (−1.1 ppm error).

3,3'-(1-Phenylethane-1,1-diyl)bis(1*H*-indole) (16)

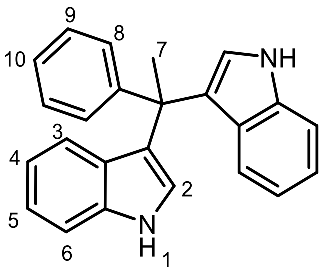

16

Synthesised using general procedure A from indole (23 mg, 0.20 mmol), phenylacetylene (33 µL, 31 mg, 0.30 mmol) and [bis(trifluoromethanesulfonyl)imidate](triphenylphosphine)gold(I) (2:1) toluene adduct (16 mg, 0.01 mmol) in toluene (2 mL) for 2 hours at 40 °C. Purification by flash column chromatography (9:1 to 8:2 hexane/ethyl acetate) afforded the *title product* as a brown solid (27 mg, 81%).

R_f (hexane/ethyl acetate 8:2) 0.32;

δ_H (400 MHz; $CDCl_3$) 2.38 (3 H, s, H-7), 6.66 (2 H, d, *J* = 2.5, H-2), 6.94 (2 H, ddd, *J* = 8.0, 7.0, 1.0, H-4), 7.12 – 7.17 (2 H, m, H-5), 7.18 – 7.23 (1 H, m, H-10), 7.24 – 7.29 (2 H, m, H-Ar), 7.34 (2 H, d, *J* = 8.0, H-6), 7.37 (2 H, d, *J* = 8.0, H-3), 7.39 – 7.43 (2 H, m, H-Ar), 7.90 (2 H, br s, H-1).

Spectroscopic data matches those reported previously.[11]

4-(5-Methyl-1*H*-indol-3-yl)-4-phenylbut-3-en-2-one (17)

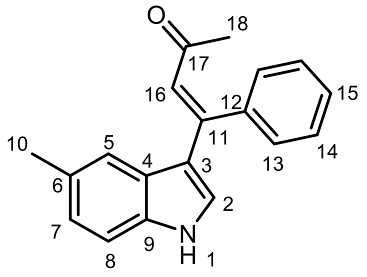

17

Synthesised using general procedure A from 5-methylindole (26 mg, 0.20 mmol), 4-phenyl-3-butyne-2-one (44 µL, 43 mg, 0.30 mmol) and [bis(trifluoromethanesulfonyl)imidate]-(triphenylphosphine)gold(I) (2:1) toluene adduct (16 mg, 0.01 mmol) in toluene (2 mL) for 2 hours at 40 °C. Purification by flash column chromatography (6:4 hexane/diethyl ether) afforded the *title product* as a yellow solid, as a mixture of *E* and *Z* stereoisomers (32 mg, 58%, *E*:*Z* = 70:30).

R_f (hexane/diethyl ether 6:4) 0.17;

mp 133–137 °C;

ν_{max} (thin film)/cm^{-1} 3263 (br), 3054, 2922, 1619, 1555, 1511, 1420, 720, 699;

δ_H (400 MHz; $CDCl_3$) 1.87 (3 H, s, H-18, *E*), 1.99 (3 H, s, H-18, *Z*), 2.31 (3 H, s, H-10, *Z*), 2.48 (3 H, s, H-10, *E*), 6.54 (1 H, s, H-16, *Z*), 6.78 – 6.82 (2 H, m, H-Ar, *E* and *Z*), 6.88 (1 H, s, H-16, *E*), 7.03 (1 H, dd, *J* = 8.5, 1.5, H-7, *Z*), 7.11 (1 H, dd, *J* = 8.5, 1.5, H-7, *E*), 7.29 (1 H, d, *J* = 8.5, H-8, *E*), 7.30 (1 H, d, *J* = 8.5, *Z*), 7.32 – 7.36 (5 H, m, H-Ar, *E* and *Z*), 7.37 – 7.45 (6 H, m, H-Ar, *E* and *Z*), 7.66 (1 H, d, *J* = 1.5, H-5, *E*), 8.60 (1 H, br s, H-1, *Z*), 8.63 (1 H, br s, H-1, *E*);

δ_C (100 MHz; $CDCl_3$) 21.6 (C-10, *Z*), 21.8 (C-10, *E*), 29.9 (C-18, *Z*), 30.5 (C-18, *E*), 111.3 (C-8, *Z*), 111.6 (C-8, *E*), 114.1 (C-3, *Z*), 118.5 (C-3, *E*), 120.3 (C-5, *Z*), 120.8 (C-5, *E*), 123.8 (C-16, *E*), 124.5 (C-7, *Z*), 125.0 (C-7, *E*), 125.3 (C, C-Ar), 127.2 (C-16, *Z*), 127.9 (CH, C-Ar), 128.4 (CH, C-Ar), 128.4 (CH, C-Ar), 128.6 (CH, C-Ar), 128.9 (CH, C-Ar), 129.5 (CH, C-Ar), 129.5 (CH, C-Ar), 130.1 (C-6, *Z*), 130.2 (C-2, *E*), 131.1 (C-6, *E*), 134.7 (C-9, *Z*), 135.6 (C-9, *E*), 140.5 (C-11, *E*), 141.5 (C-11, *Z*), 148.3 (C-12, *Z*), 151.0 (C-12, *E*), 200.5 (C-17, *E*), 201.0 (C-17, *Z*);

HRMS (ESI$^+$) Found: 276.1386; $C_{19}H_{18}NO$ (M+H$^+$) Requires 276.1383 (−1.0 ppm error); Found: 298.1201; $C_{19}H_{17}NNaO$ (M+Na) Requires 298.1202 (0.6 ppm error).

Note that one quaternary carbon resonance could not be found in the ^{13}C NMR spectra, we believe it is overlapping with another resonance.

4-(5-Bromo-1*H*-indol-3-yl)-4-phenylbut-3-en-2-one (18)

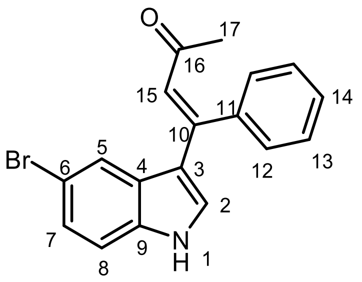

18

Synthesised using general procedure A from 5-bromo-1*H*-indole (39 mg, 0.20 mmol), 4-phenyl-3-butyne-2-one (44 µL, 43 mg, 0.30 mmol) and [bis(trifluoromethanesulfonyl)imidate]-(triphenylphosphine)gold(I) (2:1) toluene adduct (16 mg, 0.01 mmol) in toluene (2 mL) for 18 hours at 40 °C. Purification by flash column chromatography (9:1 toluene/ethyl acetate) afforded the *title product* as a yellow solid, as a mixture of *E* and *Z* stereoisomers (46 mg, 67%, *E*:*Z* = 73:27).

R_f (toluene/ethyl acetate 9:1) 0.18;

mp 138–142 °C;

ν_{max} (thin film)/cm^{-1} 3267 (br), 1666, 1628, 1552, 1515, 1456, 1421, 728;

δ_H (400 MHz; $CDCl_3$) 1.89 (3 H, s, H-17, *E*), 2.05 (3 H, s, H-17, *Z*), 6.57 (1 H, s, H-15, *Z*), 6.77 (1 H, s, H-15, *E*), 6.90 (1 H, d, *J* = 3.0, H-2, *E*), 7.08 (1 H, br s, H-Ar, *Z*), 7.27 – 7.47 (14 H, m, H-Ar, *E* and *Z*), 7.49 (1 H, d, *J* = 2.5, H-Ar, *Z*), 7.89 (1 H, d, *J* = 2.0, H-5, *E*), 8.52 (1 H, br s, H-1, *E*), 8.57 (1 H, br s, H-1, *Z*);

δ_C (100 MHz; $CDCl_3$) 30.5 (C-17, *Z*), 30.7 (C-17, *E*), 113.3 (CH, C-Ar), 113.5 (CH, C-Ar), 113.8 (C-6, *Z*), 113.9 (C-3, *Z*), 114.9 (C-6, *E*), 118.2 (C-3, *E*), 123.1 (C-5, *Z*), 123.4 (C-5, *E*), 123.6 (C-15, *E*), 125.6 (CH, C-Ar), 126.1 (CH, C-Ar), 126.5 (C, C-Ar), 126.7 (C-15, *Z*), 128.5 (CH, C-Ar), 128.6 (CH, C-Ar), 128.8 (CH, C-Ar), 128.8 (CH, C-Ar), 129.2 (CH, C-Ar), 129.4 (CH, C-Ar), 129.8 (CH, C-Ar), 130.8 (C-2, *E*), 135.0 (C-9, *Z*), 136.0 (C-9, *E*), 140.0 (C-10, *E*), 141.2 (C-10, *Z*), 147.9 (C-11, *Z*), 150.5 (C-11, *E*), 200.3 (C-16, *Z*), 200.5 (C-16, *E*);

HRMS (ESI$^+$) Found: 340.0330; $C_{18}H_{15}{}^{79}BrNO$ (M+H$^+$) Requires 340.0332 (0.5 ppm error); Found: 362.0149; $C_{18}H_{14}{}^{79}BrNNaO$ (M+Na) Requires 362.0151 (0.4 ppm error).

Note that one quaternary carbon resonance could not be found in the ^{13}C NMR spectra, we believe it is overlapping with another resonance.

4-(5-Fluoro-1*H*-indol-3-yl)-4-phenylbut-3-en-2-one (19)

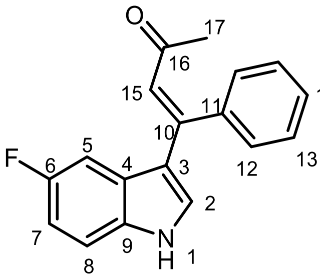

19

Synthesised using general procedure A from 5-fluoro-1*H*-indole (27 mg, 0.20 mmol), 4-phenyl-3-butyne-2-one (44 µL, 43 mg, 0.30 mmol) and [bis(trifluoromethanesulfonyl)imidate]-(triphenylphosphine)gold(I) (2:1) toluene adduct (16 mg, 0.01 mmol) in toluene (2 mL) for 18 hours at 40 °C. Purification by flash column chromatography (9:1 toluene/ethyl acetate) afforded the *title product* as a yellow solid, as a mixture of *E* and *Z* stereoisomers (44 mg, 79%, *E*:*Z* = 72:28).

R_f (toluene/ethyl acetate 9:1) 0.19;

mp 131–134 °C;

ν_{max} (thin film)/cm^{-1} 3267 (br), 2924, 1627, 1585, 1554, 1514, 1483, 721;

δ_H (600 MHz; $CDCl_3$) 1.92 (3 H, s, H-17, *E*), 2.09 (3 H, s, H-17, *Z*), 6.54 (1 H, s, H-15, *Z*), 6.57 (1 H, dd, *J* = 9.5, 2.5, H-5, *Z*), 6.75 (1 H, s, H-15, *E*), 6.92 (1 H, ddd, *J* = 9.5, 9.0, 2.5, H-7, *Z*), 6.94 (1 H, d, *J* = 3.0, H-2, *E*), 7.00 (1 H, ddd, *J* = 9.5, 9.0, 2.5, H-7, *E*), 7.27 – 7.37 (7 H, m, H-Ar, *E* and *Z*), 7.38 – 7.45 (6 H, m, H-Ar, *E* and *Z*), 7.49 (1 H, d, *J* = 2.5, H-2, *Z*), 8.82 (1 H, br s, H-1, *Z*), 8.88 (1 H, br s, H-1, *E*);

δ_C (100 MHz; $CDCl_3$) 30.4 (C-17, *Z*), 30.6 (C-17, *E*), 105.8 (d, *J* = 24.5, C-5, *Z*), 106.3 (d, *J* = 25.0, C-5, *E*), 111.2 (d, *J* = 26.5, C-7, *Z*), 111.6 (d, *J* = 26.5, C-7, *E*), 112.4 (d, *J* = 9.5, C-8), 112.7 (d, *J* = 10.0, C-8), 114.4 (d, *J* = 5.0, C-9, *Z*), 118.9 (d, *J* = 4.5, C-9, *E*), 123.4 (C-15, *E*), 125.6 (d, *J* = 10.0, C-4, *E*), 126.5 (C-15, *Z*), 127.4 (d, *J* = 10.0, C-4), 128.5 (CH, C-Ar), 128.6 (CH, C-Ar), 128.8 (CH, C-Ar), 128.9 (CH, C-Ar), 129.4 (CH, C-Ar), 129.7 (CH, C-Ar), 129.9 (CH, C-Ar), 130.9 (C-2, *E*), 132.8 (C, C-Ar), 133.8 (C, C-Ar), 140.0 (C, C-Ar), 141.3 (C, C-Ar), 147.9 (C, C-Ar), 150.4 (C, C-Ar), 158.3 (d, *J* = 236.0, C-6, *Z*), 158.9 (d, *J* = 236.5, C-6, *E*), 200.1 (C-16, *Z*), 200.2 (C-16, *E*);

δ_F (565 MHz; $CDCl_3$); −122.83 (ddd, *J* = 9.5, 9.5, 4.5, *Z*), −121.77 (ddd, *J* = 9.5, 9.5, 4.5, *E*);

HRMS (ESI$^+$) Found: 280.1132; $C_{18}H_{15}FNO$ (M+H$^+$) Requires 280.1132 (0.2 ppm error); Found: 302.0949; $C_{18}H_{14}FNNaO$ (M+Na) Requires 302.0952 (0.8 ppm error).

4-(5-Methoxy-1*H*-indol-3-yl)-4-phenylbut-3-en-2-one (20)

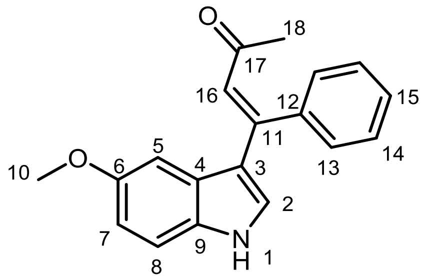

20

Synthesised using general procedure A from 5-methoxy-1*H*-indole (29 mg, 0.20 mmol), 4-phenyl-3-butyne-2-one (44 µL, 43 mg, 0.30 mmol) and [bis(trifluoromethanesulfonyl)imidate]-(triphenylphosphine)gold(I) (2:1) toluene adduct (16 mg, 0.01 mmol) in toluene (2 mL) for 18 hours at 40 °C. Purification by flash column chromatography (9:1 toluene/diethyl ether), followed by recrystallisation (hexane/diethyl) afforded the *title product* as an orange solid, as a mixture of *E* and *Z* stereoisomers (18 mg, 31%, *E*:*Z* = 76:24).

R_f (toluene/diethyl ether 9:1) 0.19;

mp 109–112 °C;

ν_{max} (thin film)/cm^{-1} 3271 (br), 2940, 2831, 1623, 1583, 1554, 1509, 1480, 1212, 720, 699;

δ_H (400 MHz; $CDCl_3$) 1.88 (3 H, s, H-18, *E*), 2.04 (3 H, s, H-18, *Z*), 3.61 (3 H, s, H-10, *Z*), 3.80 (3 H, s, H-10, *E*), 6.38 (1 H, d, *J* = 2.5, H-5, *Z*), 6.51 (1 H, s, H-16, *Z*), 6.79 (1 H, s, H-16, *E*), 6.85 (1 H, dd, *J* = 9.0, 2.5, H-7, *Z*), 6.89 – 6.93 (2 H, m, H-Ar, *E*), 7.08 (1 H, d, *J* = 2.5, H-5, *E*), 7.29 (1 H, d, *J* = 9.0, H-8, *E*), 7.29 (1 H, d, *J* = 9.0, H-8, *Z*), 7.32 – 7.37 (4 H, m, H-Ar, *E* and *Z*), 7.39 – 7.46 (7 H, m, H-Ar, *E* and *Z*), 8.56 (2 H, br s, H-1, *E* and *Z*);

δ_C (400 MHz; $CDCl_3$) 30.0 (C-18, *Z*), 30.5 (C-18, *E*), 55.6 (C-10, *Z*), 56.0 (C-18, *E*), 101.9 (C-5, *Z*), 103.2 (C-5, *E*), 112.3 (C-8, *Z*), 112.6 (C-8, *E*),113.0 (C-7, *Z*), 113.2 (C-7, *E*), 114.2 (C-3, *Z*), 118.6 (C-3, *E*), 119.3 (C, C-Ar), 123.4 (C-16, *E*), 125.8 (C-4, *E*), 126.7 (C-16, *Z*), 128.4 (CH, C-Ar), 128.5 (CH, C-Ar), 128.6 (CH, C-Ar), 128.7 (CH, C-Ar), 129.0 (CH, C-Ar), 129.5 (CH, C-Ar), 129.6 (CH, C-Ar), 129.8 (CH, C-Ar), 131.3 (C-9, *Z*), 132.3 (C-9, *E*), 140.4 (C-11, *E*), 141.5 (C-11, *Z*), 148.2 (C, C-Ar, *Z*), 150.9 (C, C-Ar, *E*), 154.8 (C-6, *Z*), 155.4 (C-6, *E*), 200.3 (C-17, *E*), 200.6 (C-17, *Z*);

HRMS (ESI$^+$) Found: 292.1337; $C_{19}H_{18}NO_2$ (M+H$^+$) Requires 292.1332 (−1.7 ppm error); Found: 314.1156; $C_{19}H_{17}NNaO_2$ (M+Na) Requires 314.1151 (0.8 ppm error).

4-(6-Methoxy-1*H*-indol-3-yl)-4-phenylbut-3-en-2-one (21)

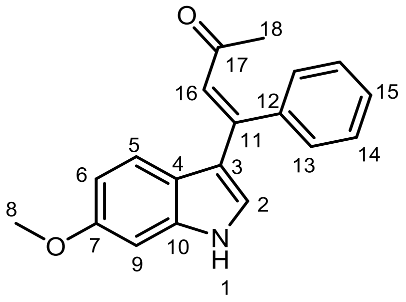

21

Synthesised using general procedure A from 6-methoxy-1*H*-indole (29 mg, 0.20 mmol), 4-phenyl-3-butyne-2-one (44 µL, 43 mg, 0.30 mmol) and [bis(trifluoromethanesulfonyl)imidate]-(triphenylphosphine)gold(I) (2:1) toluene adduct (16 mg, 0.01 mmol) in toluene (2 mL) for 21 hours at room temperature. Purification by flash column chromatography (95:5 to 9:1 toluene/diethyl ether) afforded the *title product* as a yellow solid, as a mixture of *E* and *Z* stereoisomers (18 mg, 30%, *E*:*Z* = 74:26).

R_f (toluene/diethyl ether 95:5) 0.12;

mp 45–50 °C;

ν_{max} (thin film)/cm^{-1} 3282 (br), 2931, 1627, 1557, 1519, 1453, 1415, 1238, 1159, 718;

δ_H (400 MHz; CD_2Cl_2) 1.89 (3 H, s, H-18, *E*), 2.00 (3 H, s, H-18, *Z*), 3.80 (3 H, s, H-8, *Z*), 3.83 (3 H, s, H-8, *E*), 6.48 (1 H, s, H-16), 6.63 (1 H, dd, *J* = 9.0, 2.5, H-6, *Z*), 6.78 (1 H, d, *J* = 3.0, H-2, *E*), 6.79 (1 H, s, H-16, *E*), 6.83 (1 H, dd, *J* = 9.0, 2.5, H-6, *E*), 6.90 (1 H, d, *J* = 2.5, H-9, *E*), 6.91 (1 H, d, *J* = 2.5, H-9, *Z*), 7.29 – 7.36 (5 H, m, H-Ar, *E* and *Z*), 7.37 – 7.44 (7 H, m, H-Ar, *E* and *Z*), 7.62 (1 H, d, *J* = 9.0, H-5, *E*), 8.66 (2 H, br s, H-1, *E* and *Z*);

δ_C (100 MHz; CD_2Cl_2) 30.1 (C-18, *Z*), 30.8 (C-18, *E*), 55.9 (C-8, *E* and *Z*), 95.0 (C-9, *Z*), 95.5 (C-9, *E*), 110.7 (C-6, *Z*), 111.3 (C-6, *E*), 114.6 (C-3, *Z*), 119.0 (C-3, *E*), 119.6 (C, C-Ar), 121.4 (CH, C-Ar), 121.8 (C-5, *E*), 123.2 (C-16, *E*), 127.2 (CH, C-Ar), 127.2 (CH, C-Ar), 128.4 (CH, C-Ar), 128.6 (CH, C-Ar), 129.1 (CH, C-Ar), 129.2 (CH, C-Ar), 129.6 (CH, C-Ar), 129.8 (CH, C-Ar), 137.5 (C, C-Ar), 138.6 (C, C-Ar), 140.7 (C-11, *E*), 142.1 (C-11, *Z*), 147.9 (C, C-Ar), 150.5 (C, C-Ar), 157.1 (C-7, *Z*), 157.3 (C-7, *E*), 199.4 (C-17, *E*), 200.3 (C-17, *Z*);

HRMS (ESI$^+$) Found: 292.1321; $C_{19}H_{18}NO_2$ (M+H$^+$) Requires 292.1332 (3.6 ppm error); Found: 314.1139; $C_{19}H_{17}NNaO_2$ (M+Na) Requires 314.1151 (4.1 ppm error).

Note that one quaternary and one methine carbon resonance could not be found in the ^{13}C NMR spectra, we believe they are overlapping with other resonances.

4-(6-Nitro-1H-indol-3-yl)-4-phenylbut-3-en-2-one (22)

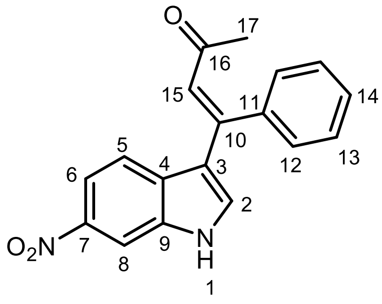

22

Synthesised using general procedure A from 6-nitro-1*H*-indole (32 mg, 0.20 mmol), 4-phenyl-3-butyne-2-one (44 µL, 43 mg, 0.30 mmol) and [bis(trifluoromethanesulfonyl)imidate]-(triphenylphosphine)gold(I) (2:1) toluene adduct (16 mg, 0.01 mmol) in toluene (2 mL) for 24 hours at 40 °C. Purification by flash column chromatography (9:1 to 7:3 toluene/diethyl ether) afforded the *title product* as a yellow solid, as a mixture of *E* and *Z* stereoisomers (29 mg, 47%, *E*:*Z* = 59:41).

R_f (toluene/diethyl ether 7:3) 0.22;

mp 50–54 °C;

ν_{max} (thin film)/cm^{-1} 3297 (br), 2925, 1590, 1507, 1337, 1311, 734;

δ_H (400 MHz; $CDCl_3$) 1.97 (3 H, s, H-17, *E*), 2.27 (3 H, s, H-17, *Z*), 6.68 (1 H, s, H-15, *Z*), 6.80 (1 H, s, H-15, *E*), 6.92 (1 H, d, *J* = 9.0, H-5, *Z*), 7.22 (1 H, d, *J* = 2.5, H-2, *E*), 7.30 – 7.46 (10 H, m, H-Ar, *E* and *Z*), 7.58 (1 H, d, *J* = 9.0, H-5, *E*), 7.69 (1 H, d, *J* = 2.0, H-2, *Z*), 7.81 (1 H, dd, *J* = 9.0, 2.0, H-6, *Z*), 8.00 (1 H, dd, *J* = 9.0, 2.0, H-6, *E*), 8.20 (1 H, d, *J* = 2.0, H-8, *Z*), 8.33 (1 H, d, *J* = 2.0, H-8, *E*), 9.88 (2 H, br s, H-1, *E* and *Z*);

δ_C (100 MHz; $CDCl_3$) 30.7 (C-17, *E*), 31.4 (C-17, *Z*), 108.8 (C-8, *Z*), 109.0 (C-8, *E*), 114.6 (C-3, *Z*), 115.8 (C-6, *Z*), 116.5 (C-6, *E*), 119.2 (C-3, *E*), 120.6 (C-5, *Z*), 120.8 (C-5, *E*), 124.6 (C-15, *E*), 125.9 (C-15, *Z*), 128.7 (CH, C-Ar), 128.9 (CH, C-Ar), 129.2 (CH, C-Ar), 129.4 (CH, C-Ar), 129.8 (C, C-Ar), 130.1 (CH, C-Ar), 131.4 (C, C-Ar), 133.8 (C-2, *Z*), 133.9 (C-2, *E*), 134.9 (C, C-Ar), 136.0 (C, C-Ar), 139.5 (C, C-Ar), 141.3 (C, C-Ar), 143.2 (C-7, *Z*), 143.6 (C-7, *E*), 147.5 (C, C-Ar), 149.6 (C, C-Ar), 199.5 (C-16, *Z*), 200.6 (C-16, *E*);

HRMS (ESI$^+$) Found: 307.1084; $C_{18}H_{15}N_2O_3$ (M+H$^+$) Requires 307.1077 (−2.1 ppm error); Found: 329.0904; $C_{18}H_{14}N_2NaO_3$ (M+Na) Requires 329.0897 (−2.2 ppm error).

Note that one methine carbon resonance could not be found in the ^{13}C NMR spectra, we believe it is overlapping with another resonance.

(*E*)-4-(4-Methyl-1*H*-indol-3-yl)-4-phenylbut-3-en-2-one (23)

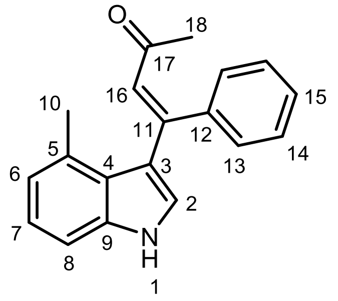

23

Synthesised using general procedure A from 4-methyl-1*H*-indole (26 mg, 0.20 mmol), 4-phenyl-3-butyne-2-one (44 µL, 43 mg, 0.30 mmol) and [bis(trifluoromethanesulfonyl)imidate]-(triphenylphosphine)gold(I) (2:1) toluene adduct (16 mg, 0.01 mmol) in toluene (2 mL) for 2 hours at 40 °C. Purification by flash column chromatography (95:5 toluene/diethyl ether) afforded the *title product* as a yellow solid (39 mg, 71%) as a single geometrical isomer (*E*).

R_f (toluene/diethyl ether 95:5) 0.19;

mp 45–47 °C;

ν_{max} (thin film)/cm^{-1} 3300 (br), 2922, 1630, 1589, 1568, 1409, 751, 731;

δ_H (400 MHz; $CDCl_3$) 1.84 (3 H, s, H-18), 2.07 (3 H, s, H-10), 6.75 (1 H, s, H-16), 6.85 (1 H, d, *J* = 7.0, H-6), 7.14 (1 H, d, *J* = 2.5, H-2), 7.16 (1 H, d, *J* = 7.5, H-8), 7.29 – 7.36 (4 H, m ,H-Ar), 7.38 – 7.42 (2 H, m, H-Ar), 8.66 (1 H, br s, H-1);

δ_C (100 MHz; CDCl3) 19.8 (C-10), 29.8 (C-18), 109.4 (CH, C-Ar), 114.6 (C-3), 122.0 (C-6), 123.3 (CH, C-Ar), 125.7 (C-4), 125.8 (C-2), 128.1 (CH, C-Ar), 128.7 (CH, C-Ar), 129.6 (CH, C-Ar), 129.8 (C-16), 131.3 (C-5), 136.9 (C-9), 141.6 (C-11), 149.4 (C-12), 201.9 (C-17);

HRMS (ESI$^+$) Found: 276.1384; $C_{19}H_{18}NO$ (M+H$^+$) Requires 276.1383 (−0.5 ppm error); Found: 298.1203; $C_{19}H_{17}NNaO$ (M+Na) Requires 298.1202 (−0.2 ppm error).

4-(4-Nitro-1*H*-indol-3-yl)-4-phenylbut-3-en-2-one (24)

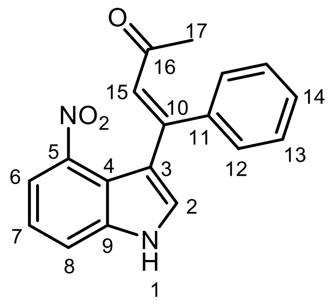

24

Synthesised using general procedure A from 4-nitro-1*H*-indole (32 mg, 0.20 mmol), 4-phenyl-3-butyne-2-one (44 µL, 43 mg, 0.30 mmol) and [bis(trifluoromethanesulfonyl)imidate]-(triphenylphosphine)gold(I) (2:1) toluene adduct (16 mg, 0.01 mmol) in toluene (2 mL) for 24 hours at 40 °C. Purification by flash column chromatography (7:3 diethyl ether/hexane) afforded the *title product* as a yellow solid, as a mixture of *E* and *Z* stereoisomers (24 mg, 39%, *E*:*Z* = 97:3).

^{1}H and ^{13}C NMR data reported for the major *E*-isomer only.

R_f (hexane/diethyl ether 3:7) 0.13;

mp 182–184 °C;

ν_{max} (thin film)/cm^{-1} 3285 (br), 2924, 1661, 1566, 1515, 1447, 1355, 1332, 736;

δ_H (400 MHz; *d*6-DMSO) 1.95 (3 H, s, H-17), 6.66 (1 H, s, H-15), 7.23 – 7.36 (6 H, m, H-Ar), 7.68 (1 H, d, *J* = 2.5, H-2), 7.79 (1 H, d, *J* = 8.0, H-8), 7.91 (1 H, d, *J* = 8.0, H-6), 12.32 (1 H, br s, H-1);

δ_C (100 MHz; *d*6-DMSO) 30.4 (C-17), 111.9 (C-3), 117.2 (C-8), 118.5 (C-4), 118.7 (C-6), 120.9 (CH, C-Ar), 125.8 (C-15), 127.8 (CH, C-Ar), 128.4 (CH, C-Ar), 129.1 (CH, C-Ar), 132.7 (C-2), 138.9 (C-9), 141.7 (C, C-Ar), 141.8 (C, C-Ar), 147.2 (C-10), 198.2 (C-16);

HRMS (ESI$^+$) Found: 307.1077; $C_{18}H_{15}N_2O_3$ (M+H$^+$) Requires 307.1077 (0.2 ppm error); +) Found: 329.0894; $C_{18}H_{14}N_2O_3$ (M+Na) Requires 329.0897 (0.8 ppm error).

Characteristic NMR resonances for the minor *Z*-isomer can be found at: δ_H 6.01 (1 H, s, H-15, *Z*).

3-(1*H*-Indol-3-yl)-1,3-diphenylprop-2-en-1-one (25)

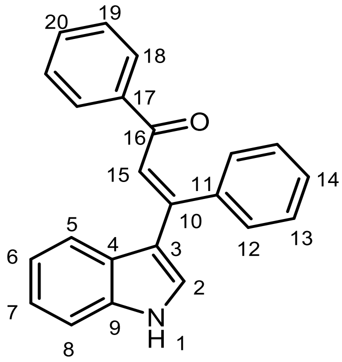

25

Synthesised using general procedure A from indole (23 mg, 0.20 mmol), diphenylpropynone (62 mg, 0.30 mmol) and [bis(trifluoromethanesulfonyl)imidate](triphenylphosphine)gold(I) (2:1) toluene adduct (16 mg, 0.01 mmol) in toluene (2 mL) for 2 hours at 40 °C. Purification by flash column chromatography (9:1 to 7:3 hexane/diethyl ether) afforded the *title product* as a yellow solid, as a mixture of *E* and *Z* stereoisomers (38 mg, 59%, *E*:*Z* = 53:47).

R_f (hexane/diethyl ether 7:3) 0.16;

mp 170–174 °C;

ν_{max} (thin film)/cm^{-1} 3284 (br), 3058, 2926, 1637, 1598, 1580, 1547, 1512, 1422, 744, 698;

δ_H (600 MHz; *d*6-DMSO) 6.67 (1 H, d, *J* = 7.5, H-5, *Z*), 6.81 (1 H, t, *J* = 7.5, H-6, *Z*), 6.99 (1 H, s, H-15, *Z*), 7.02 (1 H, t, *J* = 7.5, H-7, *Z*), 7.09 (1 H, t, *J* = 7.5, H-6, *E*), 7.19 (1 H, t, *J* = 7.5, H-7, *E*), 7.21 – 7.25 (2 H, m, H-Ar, *E*), 7.26 (1 H, d, *J* = 3.0, H-2, *E*), 7.31 – 7.39 (6 H, m, H-Ar, *E* and *Z*), 7.40 – 7.52 (11 H, m, H-Ar, *E* and *Z*), 7.52 (1 H, d, *J* = 2.5, H-2, *Z*), 7.56 (1 H, t, *J* = 7.5, H-Ar, *E*), 7.86 – 7.89 (2 H, m, H-18, *Z*), 7.89 – 7.92 (2 H, m, H-18, *E*), 11.42 (1 H, s, H-1, *Z*), 11.73 (1 H, s, H-1, *E*);

δ_C (150 MHz; *d*6-DMSO) 111.8 (C-8, *Z*), 112.5 (C-8, *E*), 112.9 (C, C-Ar, *Z*), 116.8 (C-15, *E*), 117.1 (C, C-Ar, *E*), 119.3 (C-6, *Z*), 119.9 (C-5, *Z*), 120.2 (C-5, *E*), 120.7 (C-6, *E*), 121.3 (C-7, *Z*), 121.9 (C-15, *Z*), 122.2 (C-7, *E*), 125.0 (C, C-Ar, *E*), 126.4 (C, C-Ar, *Z*), 127.7 (CH, C-Ar), 127.7 (CH, C-Ar), 128.0 (C-18, *E*), 128.1 (C-18, *Z*), 128.3 (CH, C-Ar), 128.4 (CH, C-Ar), 128.5 (CH, C-Ar), 128.6 (CH, C-Ar), 129.0 (CH, C-Ar), 129.2 (CH, C-Ar), 129.5 (C-2, *Z*), 130.4 (C-2, *E*), 132.1 (CH, C-Ar, *E*), 132.2 (CH, C-Ar, *Z*), 136.2 (C-9, *Z*), 137.4 (C-9, *E*), 138.4 (C, C-Ar), 139.2 (C, C-Ar), 140.4 (C, C-Ar, *E*), 141.9 (C, C-Ar, *Z*), 147.9 (C, C-Ar, *Z*), 151.5 (C, C-Ar), 189.9 (C-16, *E*), 191.5 (C-16, *Z*);

HRMS (ESI$^+$) Found: 324.1391; $C_{23}H_{18}NO$ (M+H$^+$) Requires 324.1383 (−2.6 ppm error); Found: 346.1204; $C_{23}H_{17}NNaO$ (M+Na) Requires 346.1202 (−0.4 ppm error).

4-(2-Methyl-1*H*-indol-3-yl)-4-phenylbut-3-en-2-one (26a)

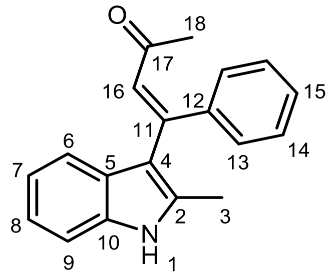

26a

Synthesised using general procedure A from 2-methyl-1*H*-indole (26 mg, 0.20 mmol), 4-phenyl-3-butyne-2-one (44 µL, 43 mg, 0.30 mmol) and [bis(trifluoromethanesulfonyl)imidate]-(triphenylphosphine)gold(I) (2:1) toluene adduct (16 mg, 0.01 mmol) in toluene (2 mL) for 19 hours at 40 °C. Purification by flash column chromatography (1:1 hexane/diethyl ether) afforded the *title product* as an orange oil, as a mixture of *E* and *Z* stereoisomers (40 mg, 73%, *E*:*Z* = 77:23).

R_f (hexane/diethyl ether 1:1) 0.20;

ν_{max} (thin film)/cm^{-1} 3284 (br), 3058, 1631, 1585, 1555, 1459, 1429, 743;

δ_H (400 MHz; $CDCl_3$) 1.89 (3 H, s, H-18, *E*), 1.96 (3 H, s, H-18, *Z*), 2.12 (3 H, s, H-3, *Z*), 2.26 (3 H, s, H-3, *E*), 6.47 (1 H, s, H-16, *Z*), 6.69 (1 H, s, H-16, *E*), 6.97 – 7.05 (3 H, m, H-Ar, *E* and *Z*), 7.12 – 7.17 (2 H, m, H-Ar, *E* and *Z*), 7.20 (1 H, d, *J* = 8.0, H-Ar, *Z*), 7.28 – 7.44 (12 H, m, H-Ar, *E* and *Z*), 8.16 (1 H, br s, H-1, *Z*), 8.23 (1 H, br s, H-1, *E*);

δ_C (100 MHz; $CDCl_3$) 12.7 (C-3, *E*), 13.7 (C-3, *Z*), 29.2 (C-18, *E*), 30.5 (C-18, *Z*), 110.7 (CH, C-Ar), 110.7 (CH,C-Ar), 111.8 (C, C-Ar, *E*), 114.5 (C, C-Ar, *Z*), 119.6 (CH, C-Ar), 119.8 (CH, C-Ar), 120.5 (CH, C-Ar), 120.7 (CH, C-Ar), 121.9 (CH, C-Ar), 122.0 (CH, C-Ar, *Z*), 126.9 (C-16, *Z*), 127.8 (C, C-Ar), 128.3 (CH, C-Ar), 128.5 (CH, C-Ar), 128.5 (CH, C-Ar), 128.6 (C, C-Ar), 128.6 (CH, C-Ar), 129.1 (CH, C-Ar), 129.6 (CH, C-Ar), 130.0 (CH, C-Ar), 135.5 (C, C-Ar), 135.6 (C, C-Ar), 135.9 (C, C-Ar), 137.2 (C, C-Ar), 140.6 (C, C-Ar, *Z*), 141.0 (C, C-Ar), 148.3 (C, C-Ar, *E*), 150.8 (C, C-Ar, *Z*), 200.8 (C-17, *E*), 200.9 (C-17, *Z*);

HRMS (ESI$^+$) Found: 276.1385; $C_{19}H_{18}NO$ (M+H$^+$) Requires 276.1383 (−0.6 ppm error); Found: 298.1202; $C_{19}H_{17}NNaO$ (M+Na) Requires 298.1202 (0.2 ppm error).

4-Phenyl-4-(2-phenyl-1*H*-indol-3-yl)but-3-en-2-one (26b)

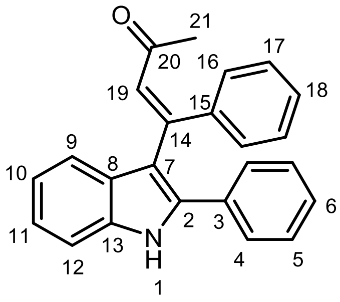

26b

Synthesised using general procedure A from 2-phenyl-1*H*-indole (39 mg, 0.20 mmol), 4-phenyl-3-butyne-2-one (44 µL, 43 mg, 0.30 mmol) and [bis(trifluoromethanesulfonyl)imidate]-(triphenylphosphine)gold(I) (2:1) toluene adduct (16 mg, 0.01 mmol) in toluene (2 mL) for 19 hours at 40 °C. Purification by flash column chromatography (7:3 to 1:1 hexane/diethyl ether) afforded the *title product* as a yellow solid, as a mixture of *E* and *Z* stereoisomers (55 mg, 81%, *E*:*Z* = 92:8).

^{1}H and ^{13}C NMR data reported for the major *E*-isomer only.

R_f (hexane/diethyl ether 7:3) 0.12

mp 153–155 °C;

ν_{max} (thin film)/cm^{-1} 3295 (br), 2924, 1631, 1449, 1432, 759, 743, 730, 694;

δ_H (400 MHz; $CDCl_3$) 1.75 (3 H, s, H-21), 6.75 (1 H, s, H-19), 7.05 – 7.10 (1 H, m, H-Ar), 7.18 – 7.34 (8 H, m, H-Ar), 7.44 (1 H, d, *J* = 8.0, H-9), 7.43 – 7.53 (4 H, m, H-Ar), 8.64 (1 H, br s, H-1);

δ_C (100 MHz; $CDCl_3$) 29.0 (C-21), 111.3 (CH, C-Ar), 111.5 (C, C-Ar), 120.0 (CH, C-Ar), 121.1 (CH, C-Ar), 123.2 (CH, C-Ar), 127.2 (CH, C-Ar), 128.2 (CH, C-Ar), 128.3 (CH, C-Ar), 128.7 (CH, C-Ar), 129.1 (CH, C-Ar), 129.2 (C, C-Ar), 129.7 (CH, C-Ar), 130.4 (C-19), 131.7 (C, C-Ar), 136.1 (C, C-Ar), 136.8 (C, C-Ar), 140.5 (C, C-Ar), 147.8 (C, C-Ar), 200.3 (C-20);

HRMS (ESI$^+$) Found: 338.1540; $C_{24}H_{20}NO$ (M+H$^+$) Requires 338.1539 (−0.1 ppm error); Found: 360.1361; $C_{24}H_{19}NNaO$ (M+Na) Requires 360.1359 (−0.5 ppm error).

Characteristic NMR resonances for the minor *Z*-isomer can be found at: δ_H 1.92 (3 H, s, H-21, *Z*), 6.38 (1 H, s, H-19, *Z*), 8.48 (1 H, br s, H-1, *Z*).

4-(1-Methyl-indol-3-yl)-4-phenylbut-3-en-2-one (27)

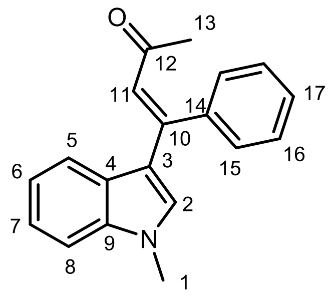

27

Synthesised using general procedure A from 1-methylindole (66 mg, 0.50 mmol), 4-phenyl-3-butyne-2-one (0.11 mL, 108 mg, 0.75 mmol) and [bis(trifluoromethanesulfonyl)imidate]-(triphenylphosphine)gold(I) (2:1) toluene adduct (40 mg, 0.03 mmol) in toluene (5 mL) for 3 hours at 40 °C. Purification by flash column chromatography (7:3 hexane/diethyl ether) afforded the *title product* as an orange oil, as a mixture of *E* and *Z* stereoisomers (108 mg, 78%, *E*:*Z* = 82:18).

R_f (hexane/diethyl ether) 0.17;

ν_{max} (thin film)/cm^{-1} 3053, 2097, 1633, 1579, 1560, 1521, 1374, 1247, 740, 706;

δ_H (600 MHz; $CDCl_3$) 1.89 (3 H, s, H-13, *E*), 2.06 (3 H, s, H-13, *Z*), 3.73 (3 H, s, H-1, *E*), 3.88 (3 H, s, H-1, *Z*), 6.49 (1 H, s, H-11, *Z*), 6.76 (1 H, s, H-2, *E*), 6.88 (1 H, s, H-11, *E*), 6.96 (1 H, ddd, *J* = 8.0, 1.0, 1.0, H-Ar, *Z*), 7.03 (1 H, ddd, *J* = 8.0 Hz, 7.0 Hz, 1.0 Hz, H-Ar, *Z*), 7.23 – 7.28 (2 H, m, H-Ar, *E* + *Z*), 7.31 – 7.39 (7 H, m, H-Ar, *E* + *Z*), 7.40 – 7.42 (1 H, m, H-Ar, *Z*), 7.43 – 7.50 (6 H, m, H-Ar, *E* + *Z*), 7.85 (1 H, d, *J* = 8.0 Hz, H-5, *E*);

δ_C (150 MHz; $CDCl_3$) 30.2 (C-13, *Z*), 30.5 (C-13, *E*), 33.2 (C-1, *E*), 33.3 (C-1, *Z*), 109.7 (CH, C-Ar), 110.1 (C, C-Ar), 112.9 (C, C-Ar), 117.4 (C, C-Ar), 120.4 (CH, C-Ar), 121.0 (CH, C-Ar), 121.2 (CH, C-Ar), 121.4 (CH, C-Ar), 122.3 (CH, C-Ar), 123.0 (CH, C-Ar), 123.3 (C-11, *E*), 125.7 (C, C-Ar), 126.3 (C-11, *Z*), 127.7 (C, C-Ar), 128.3 (CH, C-Ar), 128.4 (CH, C-Ar), 128.5 (CH, C-Ar), 129.0 (CH, C-Ar), 129.3 (CH, C-Ar), 129.5 (CH, C-Ar), 132.8 (C-2, *Z*), 134.1 (C-2, *E*), 137.3 (C-9, *Z*), 138.3 (C-9, *E*), 140.5 (C, C-Ar), 142.1 (C, C-Ar), 148.0 (C-10, *Z*), 150.4 (C-10, *E*), 199.8 (C-12, *E*), 200.1 (C-12, *Z*);

HRMS (ESI$^+$) Found: 276.1373; $C_{19}H_{18}NO$ (M+H$^+$) Requires 276.1383 (3.6 ppm error); Found: 298.1192; $C_{19}H_{17}NNaO$ (M+Na) Requires 298.1202 (3.4 ppm error).

4-(4-Bromophenyl)-4-(1*H*-indol-3-yl)but-3-en-2-one (28)

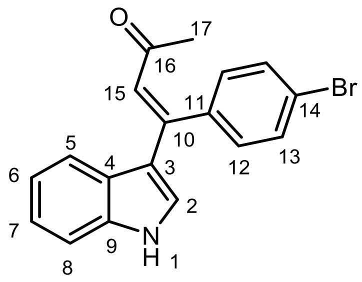

28

Synthesised using general procedure A from indole (29 mg, 0.25 mmol), 4-(4-bromophenyl)but-3-yn-2-one (84 mg, 0.38 mmol) and [bis(trifluoromethanesulfonyl)imidate](triphenylphosphine)gold(I) (2:1) toluene adduct (20 mg, 0.01 mmol) in toluene (2.5 mL) for 2 hours at 40 °C. Purification by flash column chromatography (9:1 to 6:4 hexane/diethyl ether) afforded the *title product* as a yellow solid, as a mixture of *E* and *Z* stereoisomers (55 mg, 64%, *E*:*Z* = 64:36).

R_f (hexane/diethyl ether 1:1) 0.14;

mp 135–138 °C;

ν_{max} (thin film)/cm^{-1} 3302 (br), 3061, 2926, 1666, 1552, 1514, 1486, 742;

δ_H (400 MHz; $CDCl_3$) 2.02 (6 H, s, H-17, *E* and *Z*), 6.50 (1 H, s, H-15, *Z*), 6.87 (1 H, d, *J* = 3.0, H-2, *E*), 6.89 (1 H, s, H-15, *E*), 6.99 (1 H, d, *J* = 8.5, H-Ar, *Z*), 7.01 – 7.07 (1 H, m, H-Ar, *Z*), 7.19 – 7.23 (3 H, m, H-Ar, *E* and *Z*), 7.23 – 7.26 (1 H, m, H-Ar, *E*), 7.26 – 7.31 (3 H, m, H-Ar, *E* and *Z*), 7.39 – 7.43 (3 H, m, H-Ar, *E* and *Z*), 7.45 – 7.49 (2 H, m, H-12, *Z*), 7.53 – 7.57 (2 H, m, H-12, *E*), 7.79 (1 H, d, *J* = 7.5, H-5, *E*), 8.73 (2 H, br s, H-1, *E* and *Z*);

δ_C (100 MHz; $CDCl_3$) 30.1 (C-17, *Z*), 31.1 (C-17, *E*), 111.8 (CH), 112.1 (CH), 113.8 (C-3, *Z*), 118.5 (C-3, *E*), 120.7 (CH), 120.8 (CH), 120.9 (C-5, *E*), 121.7 (CH), 122.8 (C- 14, *E*), 122.9 (C-15, *E*), 123.0 (CH), 123.5 (CH), 123.9 (C-14, *Z*), 125.0 (C), 126.7 (C), 127.0 (C-15, *Z*), 128.1 (CH), 129.8 (C-2, *E*), 130.4 (CH), 131.1 (CH), 131.6 (CH), 131.7 (CH), 136.4 (C-9, *Z*), 137.4 (C-9, *E*), 139.3 (C-10, *E*), 140.5 (C-10, *Z*), 146.8 (C-11, *Z*), 149.3 (C-11, *E*), 199.3 (C-16, *E*), 200.6 (C-16, *Z*);

HRMS (ESI$^+$) Found: 362.0153; $C_{18}H_{14}{}^{79}BrNNaO$ (M+Na) Requires 362.0151 (−0.5 ppm error); Found: 364.0133; $C_{18}H_{14}{}^{81}BrNNaO$ (M+Na) Requires 364.0131 (0.5 ppm error).

4-(1*H*-Indol-3-yl)-4-(4-methoxyphenyl)but-3-en-2-one (29)

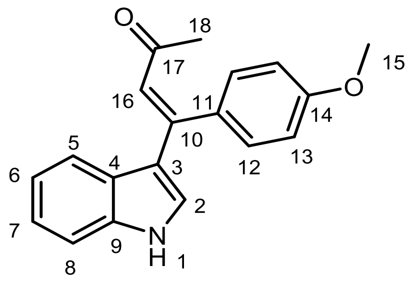

29

Synthesised using general procedure A from indole (29 mg, 0.25 mmol), 4-(4-methoxyphenyl)but-3-yn-2-one (65 mg, 0.38 mmol) and [bis(trifluoromethanesulfonyl)imidate](triphenylphosphine)gold(I) (2:1) toluene adduct (20 mg, 0.01 mmol) in toluene (2.5 mL) for 2 hours at 40 °C. Purification by flash column chromatography (1:1 hexane/diethyl ether) afforded the *title product* as a brown solid, as a mixture of *E* and *Z* stereoisomers (64 mg, 88%, *E*:*Z* = 66:34).

R_f (hexane/diethyl ether 1:1) 0.09;

mp 52–55 °C;

ν_{max} (thin film)/cm^{-1} 3282 (br), 1605, 1581, 1556, 1508, 1422, 1248, 743;

δ_H (600 MHz; $CDCl_3$) 1.91 (3 H, s, H-18, *E*), 1.96 (3 H, s, H-18, *Z*), 3.84 (3 H, s, H-15, *Z*), 3.88 (3 H, s, H-15, *E*), 6.52 (1 H, s, H-16, *Z*), 6.79 (1 H, s, H-16, *E*), 6.84 – 6.88 (2 H, m, H-13, *Z*), 6.94 – 6.97 (2 H, m, H-13, *E*), 6.98 (1 H, d, *J* = 3.0, H-2, *E*), 7.01 – 7.08 (2 H, m, H-Ar, *Z*), 7.18 – 7.23 (2 H, m, H-Ar, *E* and *Z*), 7.25 – 7.30 (3 H, m, H-Ar, *E*), 7.35 – 7.39 (2 H, m, H-12, *Z*), 7.39 – 7.45 (3 H, m, H-Ar, *E* and *Z*) 7.73 (1 H, d, *J* = 8.0, H-5, *E*), 8.53 (1 H, br s, H-1, *E*), 8.57 (1 H, br s, H-1, *Z*).

δ_C (150 MHz; $CDCl_3$) 29.9 (C-18, *Z*), 30.5 (C-18, *E*), 55.5 (C-15, *E*), 55.5 (C-15, *Z*), 111.6 (CH, C-Ar, *Z*), 111.9 (CH, C-Ar, *E*), 113.8 (C-13, *E*), 113.9 (C-13, *Z*), 114.8 (C-3, *Z*), 119.4 (C-3, *E*), 120.7 (CH, C-Ar, *Z*), 120.9 (CH, C-Ar, *Z*), 121.1 (C-5, *E*), 121.5 (CH, C-Ar, *E*), 122.8 (CH, C-Ar, *Z*), 123.3 (CH, C-Ar, *E*), 124.1 (C-16, *E*), 125.4 (C-4, *E*), 126.0 (C-16, *Z*), 127.1 (C, C-Ar, *Z*), 127.7 (CH, C-Ar, *Z*), 129.1 (C-2, *E*), 130.3 (C-12, *Z*), 131.2 (C-12, *E*), 132.5 (C-11, *E*), 133.8 (C-10, *Z*), 136.4 (C-9, *Z*), 137.3 (C-9, *E*), 147.7 (C-10, *Z*), 150.2 (C-10, *E*), 160.3 (C-14, *E*), 161.0 (C-14, *Z*), 200.5 (C-17, *E*), 200.6 (C-17, *Z*);

HRMS (ESI$^+$) Found: 292.1333; $C_{19}H_{18}NO_2$ (M+H$^+$) Requires 292.1332 (−0.4 ppm error); Found: 314.1152; $C_{19}H_{17}NNaO_2$ (M+Na) Requires 314.1151 (−0.3 ppm error).

4-(4-(Dimethylamino)phenyl)-4-(1*H*-indol-3-yl)but-3-en-2-one (30)

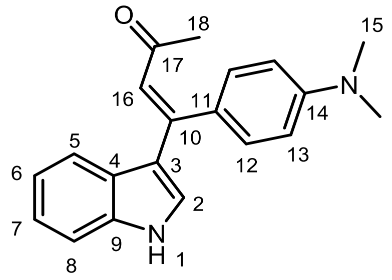

30

Synthesised using general procedure A from indole (29 mg, 0.25 mmol), 4-(4-(dimethylamino)phenyl)but-3-yn-2-one (70 mg, 0.38 mmol) and [bis(trifluoromethanesulfonyl)-imidate](triphenylphosphine)gold(I) (2:1) toluene adduct (20 mg, 0.01 mmol) in toluene (2.5 mL) for 2 hours at 40 °C. Purification by flash column chromatography (1:1 to 3:7 hexane/diethyl ether) afforded the *title product* as a yellow solid, as a mixture of *E* and *Z* stereoisomers (66 mg, 86%, *E*:*Z* = 56:44).

R_f (hexane/diethyl ether 1:1) 0.12;

mp 62–66 °C;

ν_{max} (thin film)/cm^{-1} 3254 (br), 2922, 1604, 1561, 1519, 1428, 742;

δ_H (400 MHz; $CDCl_3$) 1.91 (3 H, s, H-18, *Z*), 1.93 (3 H, s, H-18, *E*), 3.00 (6 H, s, H-15, *Z*), 3.02 (6 H, s, H-15, *E*), 6.56 (1 H, s, H-16, *Z*), 6.61 – 6.62 (2 H, m, H-13, *Z*), 6.69 – 6.74 (2 H, m, H-13, *E*), 6.70 (1 H, s, H-16, *E*), 7.01 – 7.07 (2 H, m, H-Ar, *E* and *Z*), 7.15 – 7.29 (7 H, m, H-Ar, *E* and *Z*), 7.31 – 7.35 (2 H, m, H-12, *Z*), 7.38 – 7.43 (2 H, m, H-Ar, *E* and *Z*), 7.72 (1 H, d, *J* = 8.0, H-5, *E*), 8.83 (1 H, br s, H-1, *Z*), 8.87 (1 H, br s, H-1, *E*);

δ_C (100 MHz; $CDCl_3$) 29.8 (C-18, *Z*), 39.2 (C-18, *E*), 40.3 (C-15, *Z*), 40.4 (C-15, *E*), 111.6 (C-13, *E* and *Z*), 111.9 (CH, C-Ar), 115.1 (C-3, *Z*), 119.5 (C-3, *E*), 120.5 (CH, C-Ar), 120.9 (CH, C-Ar), 121.0 (CH, C-Ar), 121.2 (CH, C-Ar), 122.6 (CH, C-Ar), 123.0 (CH, C-Ar), 123.5 (C-16, *E*), 124.0 (C-16, *Z*), 125.7 (C, C-Ar), 127.3 (C, C-Ar), 127.6 (CH, C-Ar), 128.5 (C, C-Ar), 129.3 (C-2, *E*), 130.3 (C-12, *Z*), 131.4 (C-12, *E*), 136.4 (C-9, *Z*), 137.3 (C-9, *E*), 149.0 (C, C-Ar), 151.1 (C, C-Ar), 151.5 (C, C-Ar), 151.7 (C, C-Ar), 200.7 (C-17, *Z*), 201.3 (C-17, *E*);

HRMS (ESI$^+$) Found: 305.1646; $C_{20}H_{21}N_2O$ (M+H$^+$) Requires 305.1648 (0.9 ppm error); Found: 327.1465; $C_{20}H_{20}N_2NaO$ (M+Na) Requires 327.1468 (1.0 ppm error).

Note that one quaternary and one methine carbon resonance could not be found in the ^{13}C NMR spectra, we believe they are overlapping with other resonances.

3,9-Dimethyl-9-(3-methyl-1*H*-indol-2-yl)-1-phenyl-9*H*-pyrrolo[1,2-α]indole (32)

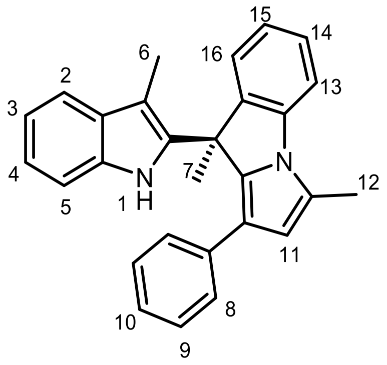

32

Synthesised using general procedure A from 3-methyl-1*H*-indole (26 mg, 0.20 mmol), 4-phenyl-3-butyne-2-one (44 µL, 43 mg, 0.30 mmol) and [bis(trifluoromethanesulfonyl)imidate]-(triphenylphosphine)gold(I) (2:1) toluene adduct (16 mg, 0.01 mmol) in toluene (2 mL) for 24 hours at 40 °C. Purification by flash column chromatography (99:1 to 98:2 hexane/diethyl ether) afforded the *title product* as a brown solid (13 mg, 33%).

R_f (hexane/diethyl ether 98:2) 0.08;

δ_H (400 MHz; $CDCl_3$) 2.01 (3 H, s, H-7), 2.09 (3 H, s, H-6), 2.69 (3 H, s, H-12), 6.35 (1 H, s, H-11), 6.99 – 7.08 (2 H, m, H-Ar), 7.09 – 7.13 (5 H, m, H-Ar), 7.15 (1 H, dd, *J* = 7.0, 1.5, H-Ar), 7.21 – 7.25 (1 H, m, H-Ar), 7.25 – 7.29 (2 H, m, H-Ar), 7.42 (1 H, d, *J* = 8.0, H-Ar), 7.51 (1 H, d, *J* = 8.0, H-Ar), 7.98 (1 H, br s, H-1).

Spectroscopic data matches those reported previously.[12]

4-(1,3-Dimethyl-indol-2-yl)-4-phenylbut-3-en-2-one (36)

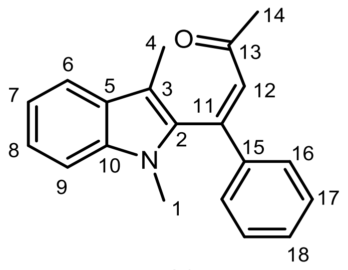

36

Synthesised using general procedure A from 1,3-dimethylindole (73 mg, 0.50 mmol), 4-phenyl-3-butyne-2-one (0.11 mL, 108 mg, 0.75 mmol) and [bis(trifluoromethanesulfonyl)imidate]-(triphenylphosphine)gold(I) (2:1) toluene adduct (40 mg, 0.03 mmol) in toluene (5 mL) for 27 hours at 40 °C. Purification by flash column chromatography (9:1 to 8:2 hexane/diethyl ether) afforded the *title product* as an orange oil, as a mixture of *E* and *Z* stereoisomers (107 mg, 74%, *E*:*Z* = 5:95).

^{1}H and ^{13}C NMR data reported for the major *Z*-isomer only.

R_f (hexane/diethyl ether 8:2) 0.23;

ν_{max} (thin film)/cm^{-1} 3060, 2919, 1657, 1599, 1573, 1467, 1244, 739;

δ_H (400 MHz; $CDCl_3$) 1.89 (3 H, s, H-14), 2.26 (3 H, s, H-4), 3.49 (3 H, s, H-1), 6.95 (1 H, s, H-12), 7.24 (1 H, ddd, *J* = 8.0, 6.5, 1.5, H-Ar), 7.32 – 7.45 (7 H, m, H-Ar), 7.63 (1 H, ddd, *J* = 8.0, 1.0, 1.0, H-Ar);

δ_C (100 MHz; $CDCl_3$) 9.3 (C-4), 28.7 (C-14), 30.6 (C-1), 109.4 (CH, C-Ar), 112.4 (C-3), 119.4 (CH, C-Ar), 119.5 (CH, C-Ar), 122.7 (CH, C-Ar), 127.5 (CH, C-Ar), 128.2 (C-5), 129.0 (CH, C-Ar), 130.0 (CH, C-Ar), 131.8 (C-12), 133.0 (C-2), 137.6 (C-10), 138.7 (C-11), 143.5 (C-15), 199.8 (C-13);

HRMS (ESI$^+$) Found: 290.1530; $C_{20}H_{20}NO$ (M+H$^+$) Requires 290.1539 (3.3 ppm error); Found: 312.1349; $C_{20}H_{19}NNaO$ (M+Na) Requires 312.1359 (3.2 ppm error).

Characteristic NMR resonances for the minor *E*-isomer can be found at: δ_H 2.14 (3 H, s, H-14, *E*), 2.39 (3 H, s, H-4, *E*), 3.34 (3 H, s, H-1, *E*), 6.37 (1 H, s, H-12, *E*).

4-(1-Methyl-3-phenyl-indol-2-yl)-4-phenylbut-3-en-2-one (37)

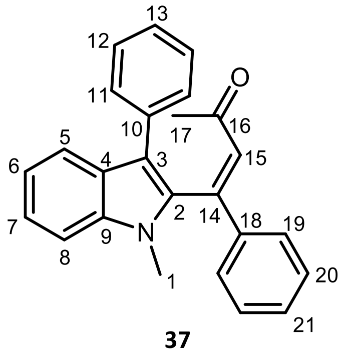

37

Synthesised using general procedure A from 1-methyl-3-phenylindole (42 mg, 0.20 mmol), 4-phenyl-3-butyne-2-one (44 µL, 43 mg, 0.30 mmol) and [bis(trifluoromethanesulfonyl)imidate]-(triphenylphosphine)gold(I) (2:1) toluene adduct (16 mg, 0.01 mmol) in toluene (2 mL) for 24 hours at 40 °C. Purification by flash column chromatography (9:1 to 8:2 hexane/diethyl ether) afforded the *title product* as an orange solid, as a mixture of *E* and *Z* stereoisomers (29 mg, 41%, *E*:*Z* = 2:98).

^{1}H and ^{13}C NMR data reported for the major *Z*-isomer only.

R_f (hexane/diethyl ether 8:2) 0.19;

mp 145–148 °C;

ν_{max} (thin film)/cm^{-1} 3056, 2931, 1690, 1659, 1602, 1465, 772, 760, 747, 699;

δ_H (400 MHz; $CDCl_3$) 1.74 (3 H, s, H-17), 3.53 (3 H, s, H-1), 6.82 (1 H, s, H-15), 7.19 – 7.26 (2 H, m, H-Ar), 7.29 – 7.44 (11 H, m, H-Ar), 7.84 (1 H, d, *J* = 8.0, H-Ar);

δ_C (100 MHz; $CDCl_3$) 28.9 (C-17), 30.7 (C-1), 109.8 (CH), 118.1 (C, C-Ar), 120.3 (C-5), 120.5 (CH, C-Ar), 123.0 (CH, C-Ar), 126.4 (CH, C-Ar), 126.9 (C, C-Ar), 127.6 (CH, C-Ar), 128.7 (CH, C-Ar), 128.9 (CH, C-Ar), 129.2 (CH, C-Ar), 130.2 (CH, C-Ar), 132.4 (C-15), 133.0 (C-2), 134.5 (C, C-Ar), 137.8 (C-9), 138.9 (C, C-Ar), 142.6 (C, C-Ar), 199.0 (C-16);

HRMS (ESI$^+$) Found: 352.1699; $C_{25}H_{22}NO$ (M+H$^+$) Requires 352.1696 (−0.7 ppm error); Found: 374.1517; $C_{25}H_{21}NNaO$ (M+Na) Requires 374.1515 (−0.4 ppm error).

Characteristic NMR resonances for the minor *E*-isomer can be found at: δ_H 1.91 (3H, s, H-17, *E*), 3.36 (3 H, s, H-1, *E*), 6.18 (1 H, s, 15, *E*).

4-(1-Benzyl-3-methyl-indol-2-yl)-4-phenylbut-3-en-2-one (38)

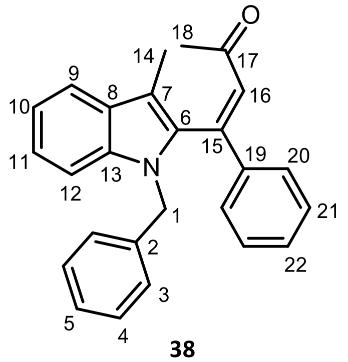

38

Synthesised using general procedure A from 1-benzyl-3-methylindole (40 mg, 0.18 mmol), 4-phenyl-3-butyne-2-one (39 µL, 39 mg, 0.27 mmol) and [bis(trifluoromethanesulfonyl)imidate]-(triphenylphosphine)gold(I) (2:1) toluene adduct (14 mg, 0.01 mmol) in toluene (1.8 mL) for 24 hours at 40 °C. Purification by flash column chromatography (9:1 to 8:2 hexane/diethyl ether) afforded the *title product* as an orange solid, as a mixture of *E* and *Z* stereoisomers (41 mg, 62%, *E*:*Z* = 7:93).

^{1}H and ^{13}C NMR data reported for the major *Z*-isomer only.

R_f (hexane/diethyl ether 8:2) 0.30;

mp 93–95 °C;

ν_{max} (thin film)/cm^{-1} 3057, 2919, 1689, 1657, 1602, 1462, 1448, 742, 696;

δ_H (400 MHz; CDCl$_3$) 1.63 (3 H, s, H-18), 2.23 (3 H, s, H-14), 4.72 (1 H, d, *J* = 16.0, H-1a), 5.12 (1 H, d, *J* = 16.0, H-1b), 6.73 (1 H, s, H-16), 6.97 – 7.02 (2 H, m, H-Ar), 7.13 – 7.39 (11 H, m, H-Ar), 7.67 (1 H, d, *J* = 7.5, H-9);

δ_C (100 MHz; CDCl$_3$) 9.5 (C-14), 28.5 (C-18), 47.9 (C-1), 110.2 (CH, C-Ar), 113.9 (C-7), 119.7 (C-9), 123.1 (CH, C-Ar), 127.2 (CH, C-Ar), 127.6 (CH, C-Ar), 127.8 (CH, C-Ar), 128.4 (C, C-Ar), 128.5 (C, C-Ar), 128.6 (CH, C-Ar), 129.0 (CH, C-Ar), 130.0 (CH, C-Ar), 132.0 (C-16), 132.8 (C-6), 137.7 (C, C-Ar), 137.8 (C-13), 139.0 (C-15), 143.4 (C, C-Ar), 199.7 (C-17);

HRMS (ESI$^+$) Found: 366.1858; $C_{26}H_{24}NO$ (M+H$^+$) Requires 366.1852 (−1.6 ppm error); Found: 388.1677; $C_{26}H_{23}NNaO$ (M+Na) Requires 388.1672 (−1.2 ppm error).

Characteristic NMR resonances for the minor *E*-isomer can be found at: δ_H 1.97 (3 H, s, H-18, *E*), 2.29 (3 H, s, H-14, *E*), 4.99 (2 H, s, H-1, *E*), 6.24 (1 H, s, H-16, *E*).

Methyl 3-(1*H*-indol-3-yl)-3-phenylacrylate (40)

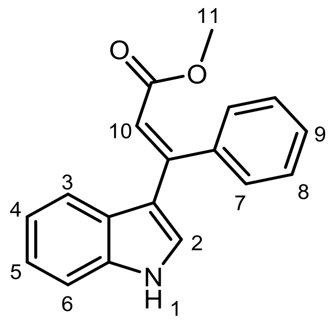

40

Synthesised using general procedure A from indole (23 mg, 0.20 mmol), methyl phenylpropiolate (44 µL, 48 mg, 0.30 mmol) and [bis(trifluoromethanesulfonyl)imidate](triphenylphosphine)gold(I) (2:1) toluene adduct (16 mg, 0.01 mmol) in toluene (2 mL) for 2 hours at 40 °C. Purification by flash column chromatography (7:3 hexane/diethyl ether) afforded the *title product* as a pale, yellow solid, as a mixture of *E* and *Z* stereoisomers (47 mg, 85%, *E*:*Z* = 78:22).

R_f (hexane/diethyl ether 7:3) 0.17;

δ_H (400 MHz; $CDCl_3$) 3.63 (3 H, s, H-11, *E*), 3.65 (3 H, s, H-11, *Z*), 6.26 (1 H, s, H-10, *Z*), 6.59 (1 H, s, H-10, *E*), 6.89 (1 H, d, *J* = 3.0, H-2, *E*), 6.93 (1 H, d, *J* = 8.0, H-Ar, *Z*), 6.99 (1 H, d, *J* = 7.5, H-Ar, *Z*), 7.14 – 7.19 (1 H, m, H-Ar, *Z*), 7.21 – 7.30 (2 H, m, H-Ar, *E*), 7.31 – 7.36 (4 H, m, H-Ar, *E* and *Z*), 7.37 – 7.44 (8 H, m, *E* and *Z*), 7.55 (1 H, d, *J* = 2.5, H-2, *Z*), 7.82 (1 H, d, *J* = 8.5, H-3, *E*), 8.41 (2 H, br s, H-1, *E* and *Z*).

Spectroscopic data matches those reported previously.[1]

Methyl 3-(4-(dimethylamino)phenyl)-3-(1*H*-indol-3-yl)acrylate (41)

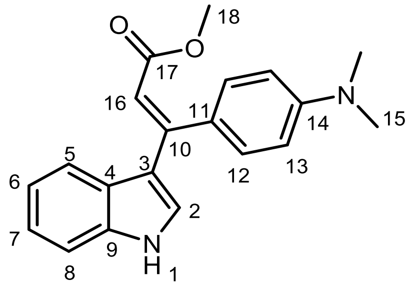

41

Synthesised using general procedure A from indole (23 mg, 0.20 mmol), methyl 3-(4-(dimethylamino)phenyl)propiolate (61 mg, 0.30 mmol) and [bis(trifluoromethanesulfonyl)imidate]-(triphenylphosphine)gold(I) (2:1) toluene adduct (16 mg, 0.01 mmol) in toluene (2 mL) for 2 hours at 40 °C. Purification by flash column chromatography (8:2 to 7:3 hexane/ethyl acetate) afforded the *title product* as a yellow solid, as a mixture of *E* and *Z* stereoisomers (39 mg, 61%, *E*:*Z* = 58:42).

R_f (hexane/ethyl acetate 8:2) 0.15, 0.10;

mp 58-65 °C;

ν_{max} (thin film)/cm^{-1} 3327 (br), 2945, 1691, 1607, 1585, 1520, 1155, 819, 744;

δ_H (600 MHz; $CDCl_3$) 3.00 (6 H, s, H-15, *E*), 3.00 (6 H, s, H-15, *Z*), 3.63 (3 H, s, H-18, *Z*), 3.68 (3 H, s, H-18, *E*), 6.25 (1 H, s, H-16, *Z*), 6.41 (1 H, s, H-16, *E*), 6.61 – 6.65 (2 H, m, H-13, *Z*), 6.69 – 6.75 (2 H, m, H-13, *E*), 6.98 (1 H, d, *J* = 2.5, H-2, *E*), 7.01 (1 H, ddd, *J* = 8.0, 7.0, 1.0, H-Ar, *Z*), 7.12 (1 H, dd, *J* = 8.0, 1.0, H-Ar, *Z*), 7.13 – 7.19 (2 H, m, H-Ar, *E*), 7.21 – 7.25 (1 H, m, H-Ar, *E*), 7.25 – 7.27 (2 H, m, H-12, *Z*), 7.31 – 7.37 (5 H, m, H-Ar, *E* and *Z*), 7.71 (1 H, dd, *J* = 8.0, 1.0, H-5, *E*), 8.55 (2 H, br s, H-1, *E* and *Z*);

δ_C (150 MHz; $CDCl_3$) 40.3 (C-15, *Z*), 40.5 (C-15, *E*), 51.0 (C-18, *E*), 51.1 (C-18, *Z*), 110.6 (C-16, *E*), 111.3 (CH, C-Ar), 111.4 (CH, C-Ar), 111.6 (CH, C-Ar), 111.7 (CH, C-Ar), 111.8 (CH, C-Ar), 114.2 (C-3, *Z*), 119.7 (C-3, *E*), 120.1 (CH, C-Ar), 120.8 (CH, C-Ar), 121.0 (CH, C-Ar), 121.1 (CH, C-Ar), 122.1 (CH, C-Ar), 122.8 (CH, C-Ar), 125.8 (C-4, *E*), 127.3 (C, C-Ar), 127.5 (C, C-Ar), 127.5 (CH, C-Ar), 128.9 (C-2, *E*), 129.4 (C-11, *Z*), 130.2 (C-12, *E*), 130.9 (C-12, *Z*), 136.1 (C-9, *Z*), 137.1 (C-9, *E*), 150.7 (C, C-Ar), 151.3 (C, C-Ar), 151.5 (C, C-Ar), 153.5 (C-11, *E*), 167.6 (C-17, *Z*), 167.8 (C-17, *E*);

HRMS (ESI$^+$) Found: 321.1584; $C_{20}H_{21}N_2O_2$ (M+H$^+$) Requires 321.1598 (4.2 ppm error); Found: 343.1403; $C_{20}H_{20}N_2NaO_2$ (M+Na) Requires 343.1417 (4.0 ppm error); Found: 289.1322; $C_{19}H_{17}N_2O$ (M−OMe) Requires 289.1335 (−4.5 ppm error).

Methyl (*Z*)-3-(1,3-dimethyl-indol-2-yl)-3-phenylacrylate (42)

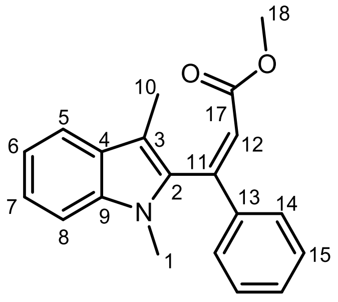

42

Synthesised using general procedure A from 1,3-dimethylindole (29 mg, 0.20 mmol), methyl phenylpropiolate (44 µL, 48 mg, 0.30 mmol) and [bis(trifluoromethanesulfonyl)imidate]-(triphenylphosphine)gold(I) (2:1) toluene adduct (16 mg, 0.01 mmol) in toluene (2 mL) for 24 hours at 40 °C. Purification by flash column chromatography (9:1 hexane/diethyl ether) afforded the *title product* as a yellow oil (43 mg, 70%) as a single geometrical isomer (*Z*).

R_f (hexane/diethyl ether 9:1) 0.17;

ν_{max} (thin film)/cm^{-1} 3061, 2950, 1690, 1728, 1623, 1467,1449, 1260, 741;

δ_H (400 MHz; $CDCl_3$) 2.17 (3 H, s, H-10), 3.44 (3 H, s, H-1), 3.64 (3 H, s, H-18), 6.74 (1 H, s, H-12), 7.15 (1 H, ddd, *J* = 8.0, 6.5, 1.0, H-6), 7.24 – 7.28 (1 H, m, H-Ar), 7.29 – 7.43 (6 H, m, H-Ar), 7.63 (1 H, d, *J* = 8.0, H-5);

δ_C (100 MHz; $CDCl_3$) 9.3 (C-10), 30.7 (C-1), 51.8 (C-18), 109.3 (CH, C-Ar), 110.7 (C-3), 118.9 (C-6), 119.3 (C-5), 121.1 (C-12), 122.0 (CH, C-Ar), 127.6 (CH, C-Ar), 128.3 (C-4), 129.0 (CH, C-Ar), 130.0 (CH, C-Ar), 133.5 (C-2), 137.6 (C-9), 139.0 (C-11), 146.5 (C-13), 166.0 (C-17);

HRMS (ESI$^+$) Found: 306.1489; $C_{20}H_{20}NO_2$ (M+H$^+$) Requires 306.1489 (0.0 ppm error); Found: 328.1311; $C_{20}H_{19}NNaO_2$ (M+Na) Requires 328.1308 (−0.9 ppm error); Found: 274.1227; $C_{19}H_{16}NO$ (M−OMe) Requires 274.1226 (0.4 ppm error).

3-(1*H*-Indol-3-yl)-N,N-dimethyl-3-phenylacrylamide (44)

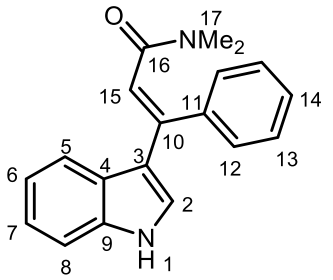

44

Synthesised using general procedure A from indole (23 mg, 0.20 mmol), *N*,*N*-dimethyl-3-phenylpropiolamide (52 mg, 0.30 mmol) and [bis(trifluoromethanesulfonyl)imidate]-(triphenylphosphine)gold(I) (2:1) toluene adduct (16 mg, 0.01 mmol) in toluene (2 mL) for 24 hours at 40 °C. Purification by flash column chromatography (7:3 diethyl ether/hexane to ethyl acetate) afforded the *title product* as a pale, brown solid, as a mixture of *E* and *Z* stereoisomers (33 mg, 56%, *E*:*Z* = 26:74).

R_f (hexane/ethyl acetate 3:7) 0.33;

mp 124–129 °C;

ν_{max} (thin film)/cm^{-1} 3218 (br), 2925, 1613, 1494, 1399, 1199, 736;

δ_H (600 MHz; $CDCl_3$) 2.78 (3 H, s, H-17a, *Z*), 2.85 (3 H, s, H-17a, *E*), 2.86 (3 H, s, H-17b, *E*), 2.87 (3 H, s, H-17b, *Z*), 6.28 (1 H, s, H-15, *Z*), 6.55 (1 H, s, H-15, *E*), 6.88 (1 H, d, *J* = 8.0, H-5, *Z*), 6.93 (1 H, dd, *J* = 8.0, 8.0, H-6, *Z*), 6.98 (1 H, d, *J* = 2.5, H-2, *E*), 7.14 (2 H, m, H-Ar, *E* and *Z*), 7.23 (1 H, dd, *J* = 7.0, 7.0, H-7, *E*), 7.29 – 7.42 (12 H, m, H-Ar, *E* and *Z*), 7.43 (1 H, d, *J* = 2.5, H-2, *Z*), 7.61 (1 H, d, *J* = 8.0, H-5, *E*), 8.52 (1 H, br s, H-1, *E*), 8.72 (1 H, br s, H-1, *Z*);

δ_C (150 MHz; $CDCl_3$) 34.7 (C-17a, *E*), 34.7 (C-17a, *Z*), 38.1 (C-17b, *E*), 38.1 (C-17b, *Z*), 111.5 (CH, C-Ar), 111.8 (CH, C-Ar), 114.1 (C-3, *Z*), 117.7 (C-15, *E*), 118.4 (C-3, *E*), 119.9 (C-15, *Z*), 120.0 (C-6, *Z*), 120.6 (C-5, *E*), 120.7 (C-5, *Z*), 120.8 (C-7, *E*), 122.2 (C-6, *Z*), 122.8 (C-7, *E*), 125.8 (C, C-Ar), 126.2 (C-2, *Z*), 126.4 (C-2, *E*), 126.6 (C, C-Ar), 128.1 (2 x CH, C-Ar), 128.3 (CH, C-Ar), 128.4 (CH, C-Ar), 128.6 (CH, C-Ar), 129.3 (CH, C-Ar), 136.3 (C-9, *Z*), 137.0 (C-9, *E*), 140.2 (C, C-Ar, *E*), 141.2 (C, C-Ar, *Z*), 141.4 (C, C-Ar, *Z*), 142.8 (C, C-Ar, *E*), 169.2 (C-16, *E*), 169.6 (C-16, *Z*);

HRMS (ESI^+) Found: 291.1491; $C_{19}H_{19}N_2O$ ($M+H^+$) Requires 291.1492 (0.2 ppm error); Found: 313.1310; $C_{19}H_{18}N_2NaO$ (M+Na) Requires 313.1311 (0.5 ppm error).

3-(1*H*-Indol-3-yl)-3-(4-methoxyphenyl)-*N*,*N*-dimethylacrylamide (45)

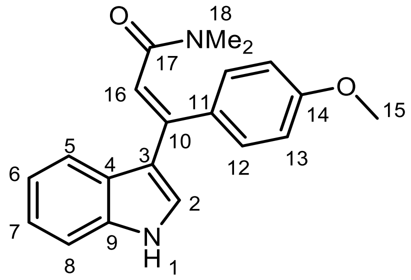

45

Synthesised using general procedure A from indole (23 mg, 0.20 mmol), 3-(4-methoxyphenyl)-N,N-dimethylpropiolamide (61 mg, 0.30 mmol) and [bis(trifluoromethanesulfonyl)imidate]-(triphenylphosphine)gold(I) (2:1) toluene adduct (16 mg, 0.01 mmol) in toluene (2 mL) for 2 hours at 40 °C. Purification by flash column chromatography (1:1 to 2:8 hexane/ethyl acetate) afforded the *title product* as a pale, brown solid, as a mixture of *E* and *Z* stereoisomers (64 mg, 100%, *E*:*Z* = 45:55).

R_f (hexane/ethyl acetate 3:7) 0.23;

mp 78–85 °C;

ν_{max} (thin film)/cm^{-1} 3205 (br), 2927, 1604, 1509, 1457, 1439, 1397, 1247, 742;

δ_H (600 MHz; $CDCl_3$) 2.76 (3 H, s, H-18a, *Z*), 2.85 (3 H, s, H-18b, *Z*), 2.86 (3 H, s, H-18a, *E*), 2.91 (3 H, s, H-18b, *E*), 3.78 (3 H, s, H-15, *E*), 3.82 (3 H, s, H-15, *Z*), 6.21 (1 H, s, H-16, *Z*), 6.40 (1 H, s, H-16, *E*), 6.78 – 6.82 (2 H, m ,H-13, *E*), 6.82 – 6.85 (2 H, m, H-13, *Z*), 6.90 – 6.94 (3 H, m, H-Ar, *Z* and *E*), 7.06 – 7.10 (2 H, m, H-Ar, *Z* and *E*), 7.16 (1 H, t, *J* = 7.5, H-Ar, *E*), 7.23 – 7.27 (2 H, m, H-12, *E*), 7.30 – 7.33 (3 H, m, H-Ar, *Z*), 7.34 (1 H, d, *J* = 8.5, H-Ar, *Z*), 7.37 (1 H, d, *J* = 8.5, H-Ar, *E*), 7.53 (1 H, d, *J* = 8.0, H-5, *E*), 9.39 (1 H, br s, H-1, *E*), 9.44 (1 H, br s, H-1, *Z*);

δ_C (150 MHz; $CDCl_3$) 34.7 (C-18a, *E*), 34.8 (C-18a, *Z*), 38.1 (C-18b, *E*), 38.1 (C-18b, *Z*),55.3 (C-15, *E*), 55.4 (C-15, *Z*), 111.7 (CH, C-Ar), 112.0 (CH, C-Ar), 113.5 (C-13, *E*), 113.7 (C-13, *Z*), 113.9 (C-3, *Z*), 116.4 (C-16, *E*), 117.8 (C-16, *Z*), 118.0 (C-3, *E*), 119.8 (CH, C-Ar), 120.3 (CH, C-Ar), 120.5 (CH, C-Ar), 121.9 (CH, C-Ar), 122.3 (CH, C-Ar), 125.8 (C-4, *E*), 126.4 (C-2, *E*), 126.6 (C-4, *Z*), 126.8 (C-2, *Z*), 129.4 (C-12, *Z*), 130.6 (C-12, *E*), 132.5 (C, C-Ar), 133.7 (C, C-Ar), 136.5 (C-9, *Z*), 137.1 (C-9, *E*), 141.5 (C, C-Ar), 142.9 (C, C-Ar), 159.6 (C-14, *E*), 160.0 (C-14, *Z*), 169.8 (C-17, *E*), 170.0 (C-17, *Z*);

HRMS (ESI$^+$) Found: 321.1590; $C_{20}H_{21}N_2O_2$ (M+H$^+$) Requires 321.1598 (2.4 ppm error); Found: 343.1411; $C_{20}H_{20}N_2NaO_2$ (M+Na) Requires 343.1417 (1.8 ppm error).

3-(1,3-Dimethyl-indol-2-yl)-*N*,*N*-dimethyl-3-phenylacrylamide (46)

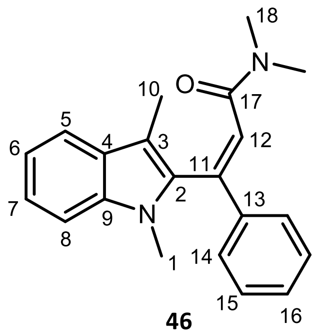

46

Synthesised using general procedure A from 1,3-dimethylindole (29 mg, 0.20 mmol), *N*,*N*-dimethyl-3-phenylpropiolamide (52 mg, 0.30 mmol) and [bis(trifluoromethanesulfonyl)imidate]-(triphenylphosphine)gold(I) (2:1) toluene adduct (16 mg, 0.01 mmol) in toluene (2 mL) for 48 hours at 40 °C. Purification by flash column chromatography (8:2 to 2:1 hexane/diethyl ether) afforded the *title product* as a yellow oil, as a mixture of *E* and *Z* stereoisomers (38 mg, 59%, *E*:*Z* = 7:93).

^{1}H and ^{13}C NMR data reported for the major *Z*-isomer only.

R_f (hexane/ethyl acetate 2:1) 0.20;

ν_{max} (thin film)/cm^{-1} 3053, 2924, 1629, 1493,1469, 1139, 732, 696;

δ_H (400 MHz; CDCl$_3$) 2.20 (3 H, s, H-10), 2.93 (6 H, br s, H-18), 3.44 (3 H, s, H-1), 6.89 (1 H, s, H-12), 7.11 – 7.16 (1 H, m, H-6), 7.21 – 7.30 (4 H, m, H-Ar), 7.30 – 7.36 (3 H, m, H-Ar), 7.60 (1 H, d, *J* = 8.0, H-5);

δ_C (100 MHz; CDCl$_3$) 9.5 (C-10), 31.0 (C-1), 35.1 (br, C-18a), 37.9 (br, C-18b), 109.4 (CH, C-Ar), 110.4 (C-3), 118.9 (C-6), 119.2 (C-5), 121.9 (CH, C-Ar), 125.8 (C-12), 127.0 (CH, C-Ar), 128.4 (C-4), 128.9 (CH, C-Ar), 129.1 (CH, C-Ar), 133.9 (C-2), 137.6 (C-9), 139.4 (C, C-Ar), 139.4 (C, C-Ar), 167.3 (C-17);

HRMS (ESI$^+$) Found: 319.1804; $C_{21}H_{23}N_2O$ (M+H$^+$) Requires 319.1805 (0.2 ppm error); Found: 341.1624; $C_{21}H_{22}N_2NaO$ (M+Na) Requires 341.1624 (−0.0 ppm error); Found: 278.1228; $C_{19}H_{16}NO$ (M−NMe$_2$) Requires 278.1226 (0.7 ppm error).

Characteristic NMR resonances for the minor *E*-isomer can be found at: δ_H 2.44 (3 H, s, H-10, *E*), 3.30 (3 H, s, H-1, *E*), 6.24 (1 H, s, H-12, *E*).

Gold-pyrylium Complex

3-Acetyl-2,4-bis(4-(dimethylamino)phenyl)-6-methylpyryl-5-yl(triphenylphosphine)gold(I) bis[(trifluoromethane)sulfonyl]azanide (12)

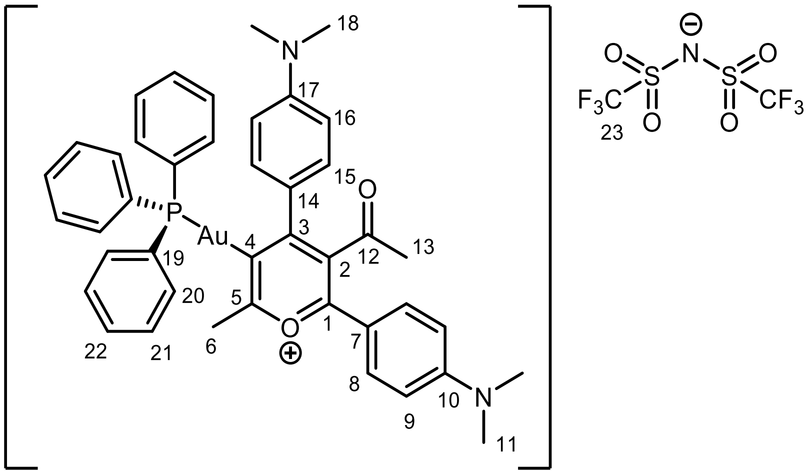

12

For the ^{1}H, ^{19}F and ^{31}P NMR spectra, the clearest data for the *title product* was observed when a more dilute sample was prepared, using 4 equivalents (a 2-fold excess) of the starting ynone. As such, to a sample vial, 4-(4-(dimethylamino)phenyl)but-3-yn-2-one (15 mg, 0.08 mmol) was dissolved in *d*2-DCM (0.6 mL), and [bis(trifluoromethanesulfonyl)imidate](triphenylphosphine)gold(I) (2:1) toluene adduct (16 mg, 0.01 mmol) was added, which resulted in an immediate colour change to dark red. The sample was transferred to an NMR tube and ^{1}H, ^{19}F and ^{31}P data were recorded immediately.

For the ^{13}C NMR spectra, a more concentrated sample was prepared using the same method, with 4-(4-(dimethylamino)phenyl)but-3-yn-2-one (78 mg, 0.4 mmol) and [bis(trifluoromethanesulfonyl)-imidate](triphenylphosphine)gold(I) (2:1) toluene adduct (163 mg, 0.1 mmol). After transferring the sample to an NMR tube, ^{13}C NMR data was recorded immediately.

ν_{max} (thin film)/cm^{-1} 2910, 1705, 1597, 1423, 1348, 1137, 1056;

δ_H (400 MHz; CD_2Cl_2) 2.05 (3 H, s, H-13), 2.97 (6 H, s, H-11 or H-18), 3.03 (3 H, s, H-6), 3.13 (6 H, s, H-11 or H-18), 6.54 – 6.59 (2 H, m, H-9 or H-16), 6.76 – 6.80 (2 H, m, H-9 or H-16), 7.35 – 7.50 (15 H, m, PPh_3), 7.57 – 7.61 (2 H, m, H-8 or H-15), 7.69 – 7.73 (2 H, m, H-8 or H-15);

δ_C (100 MHz; CD_2Cl_2) 25.9 (C-13), 32.1 (C-6), 40.1 (C-11 or C-18), 40.2 (C-11 or C-18), 111.5 (C-9 or C-16), 111.2 (C-9 or C-16), 115.7 (C-7 or C-14), 120.3 (q, $^{1}J_{C\text{-}F}$ = 322.0, C-23), 127.2 (C-7 or C-14), 129.6 (d, $^{3}J_{C\text{-}P}$ = 11.5, C-21), 129.8 (d, $^{1}J_{C\text{-}P}$ = 55.0, C-19), 131.0 (d, $^{4}J_{C\text{-}P}$ = 4.0, C-2), 131.7 (C-8 or C-15), 131.8 (C8 or C-15), 132.2 (d, $^{4}J_{C\text{-}P}$ = 2.0, C-22), 134.4 (d, $^{2}J_{C\text{-}P}$ = 13.5, C-20), 152.4 (C-10 or C-17), 154.1 (C-10 or C-17), 161.4 (d, $^{2}J_{C\text{-}P}$ = 111.0, C-4), 165.5 (C-3), 172.1 (C-1), 175.7 (d, $^{3}J_{C\text{-}P}$ = 5.0, C-5), 202.3 (C-12);

δ_P (162 MHz; CD_2Cl_2) 41.9;

δ_F (376 MHz; CD_2Cl_2) −79.4;

HRMS (ESI$^+$) Found: 833.2579; $C_{42}H_{41}AuN_2O_2P$ (M^+) Requires 833.2566 (−1.6 ppm error).

Note that toluene is present in the ^{1}H and ^{13}C NMR spectra due to its inclusion in the crystal structure of gold triflimide.

NMR Spectra

47

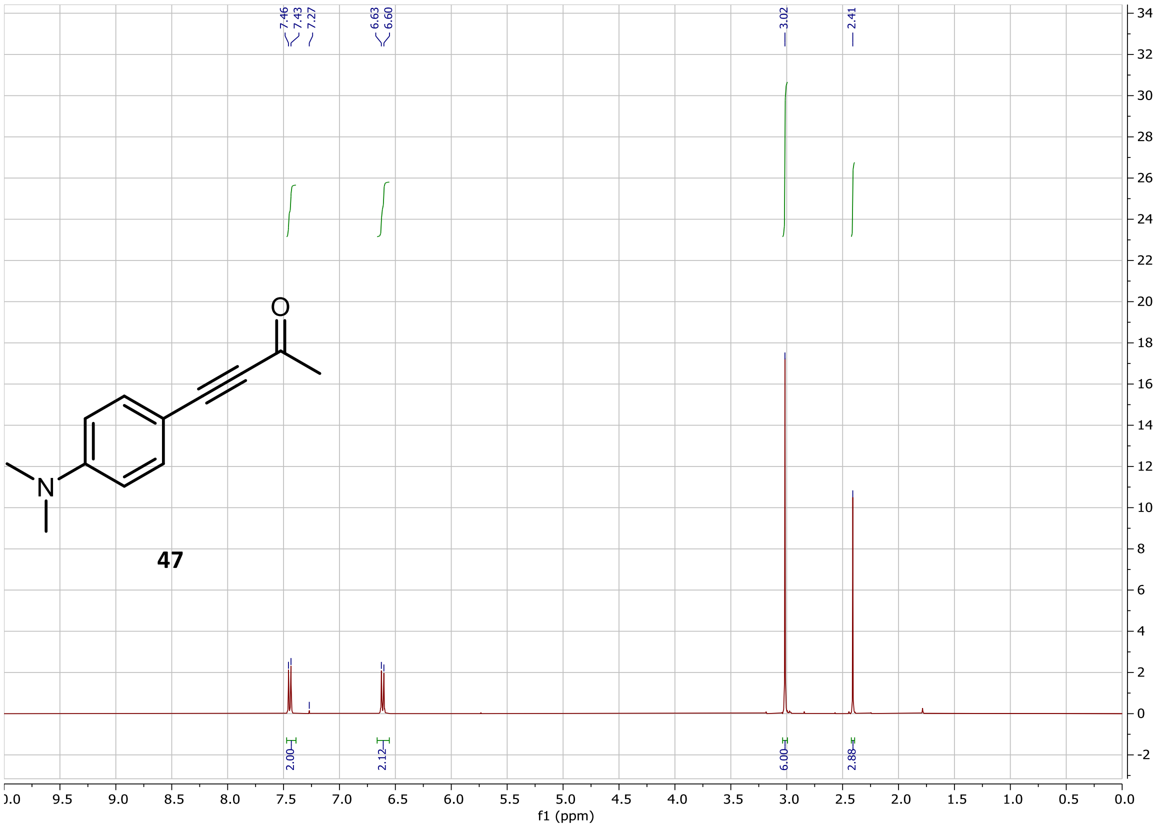

Figure S5 - ^{1}H NMR spectrum of **47** recorded in $CDCl_3$ solution.

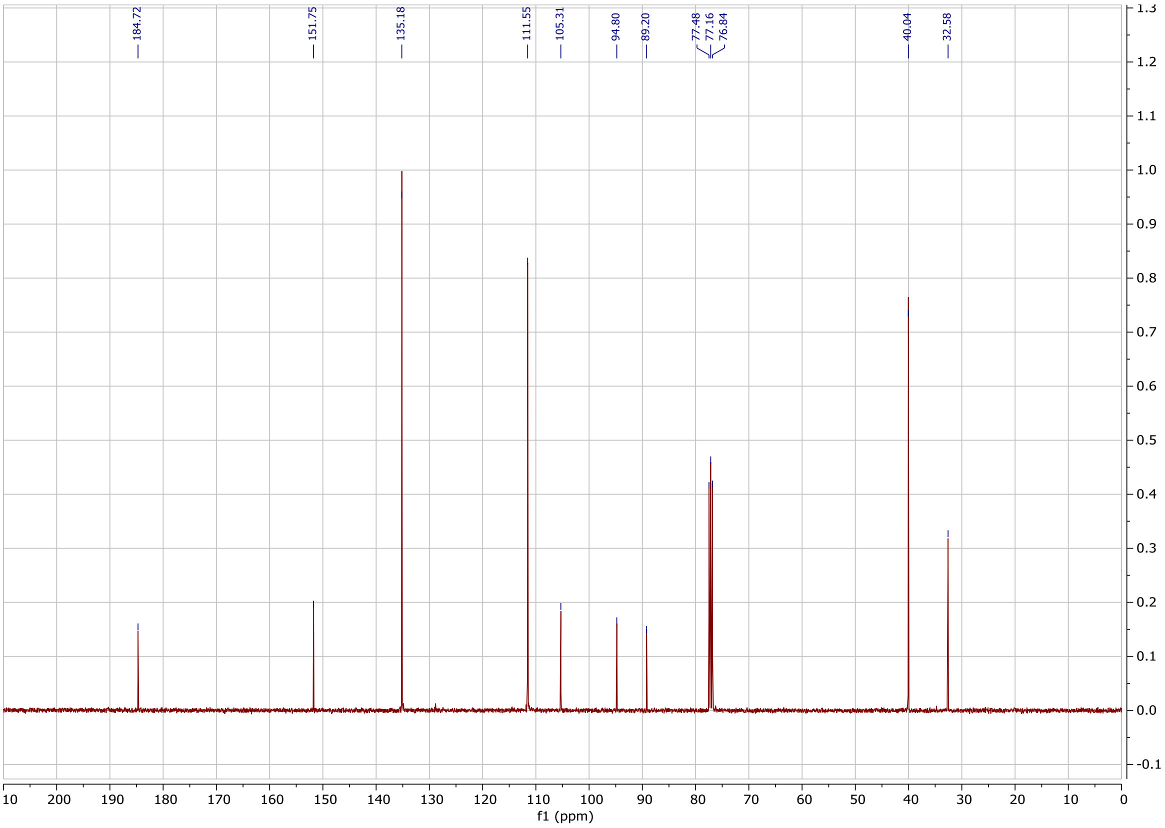

Figure S6 - $^{13}C\{^{1}H\}$ NMR spectrum of 47 recorded in $CDCl_3$ solution.

49

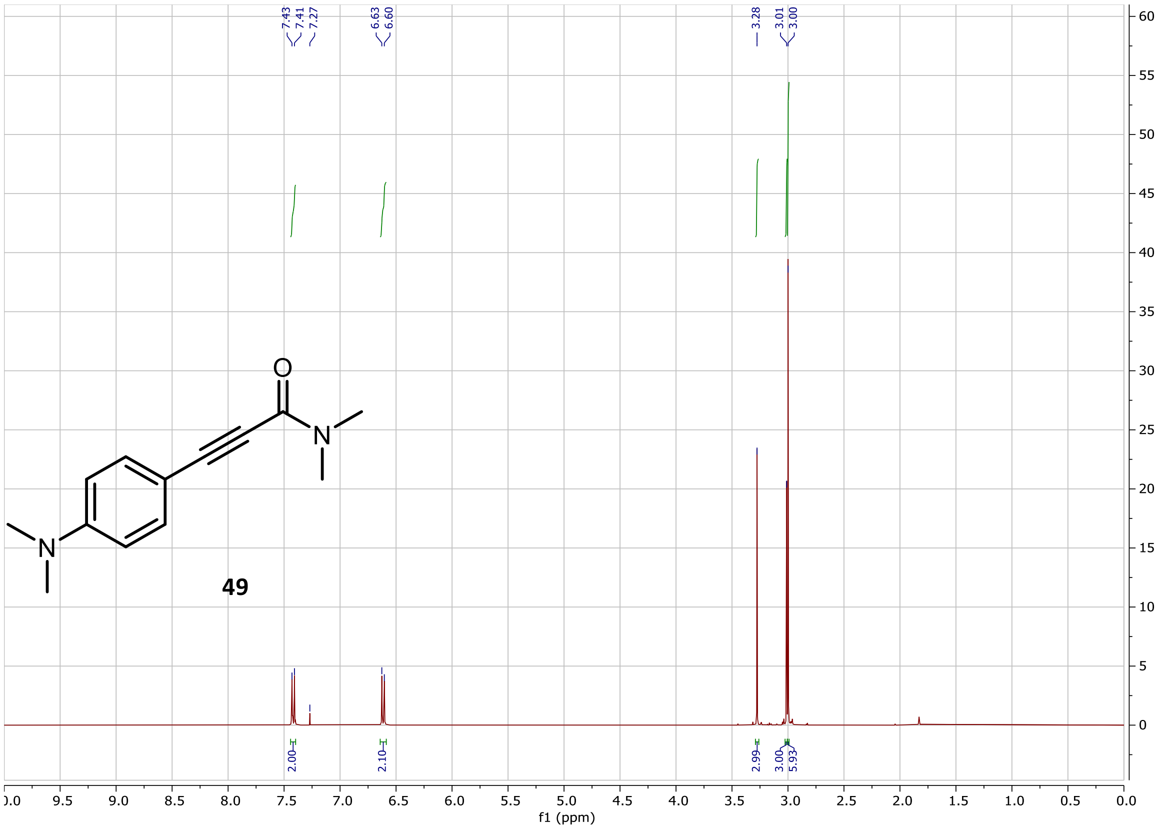

Figure S7 - ^{1}H NMR spectrum of **49** recorded in $CDCl_3$ solution.

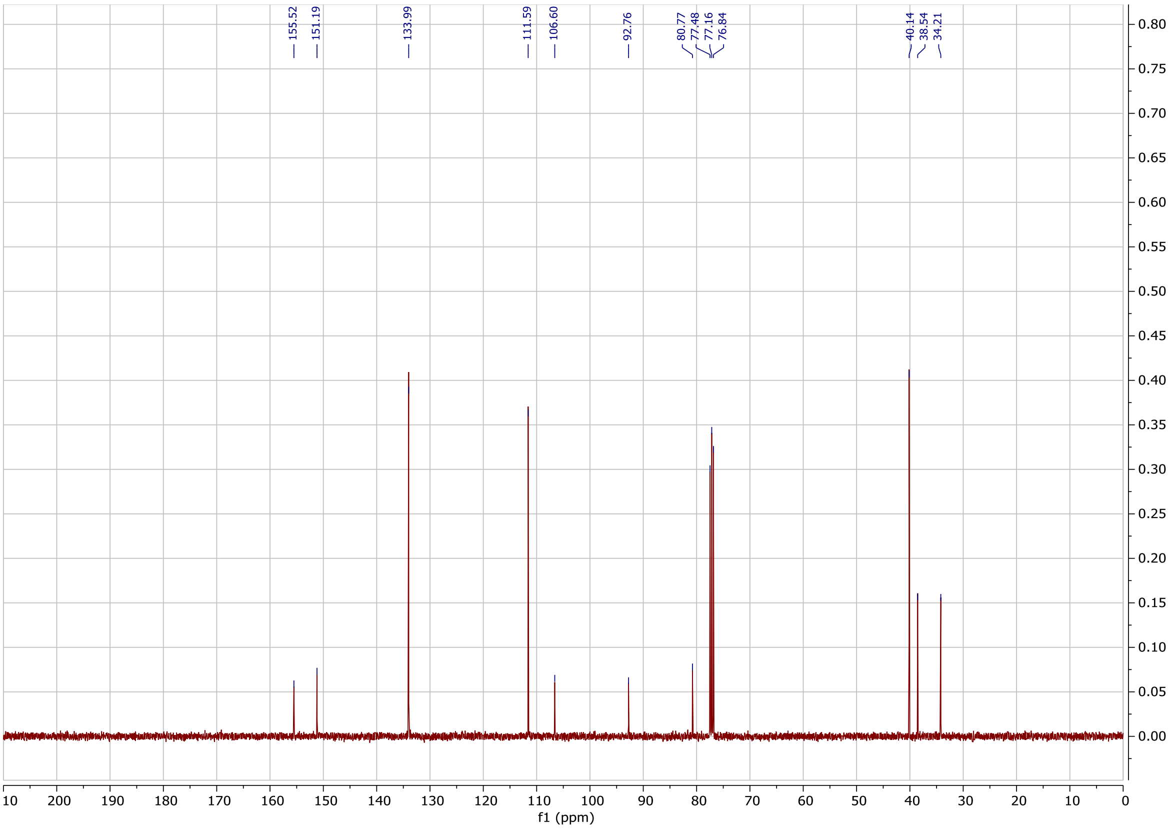

Figure S8 - $^{13}C\{^{1}H\}$ NMR spectrum of **49** recorded in $CDCl_3$ solution.

14a

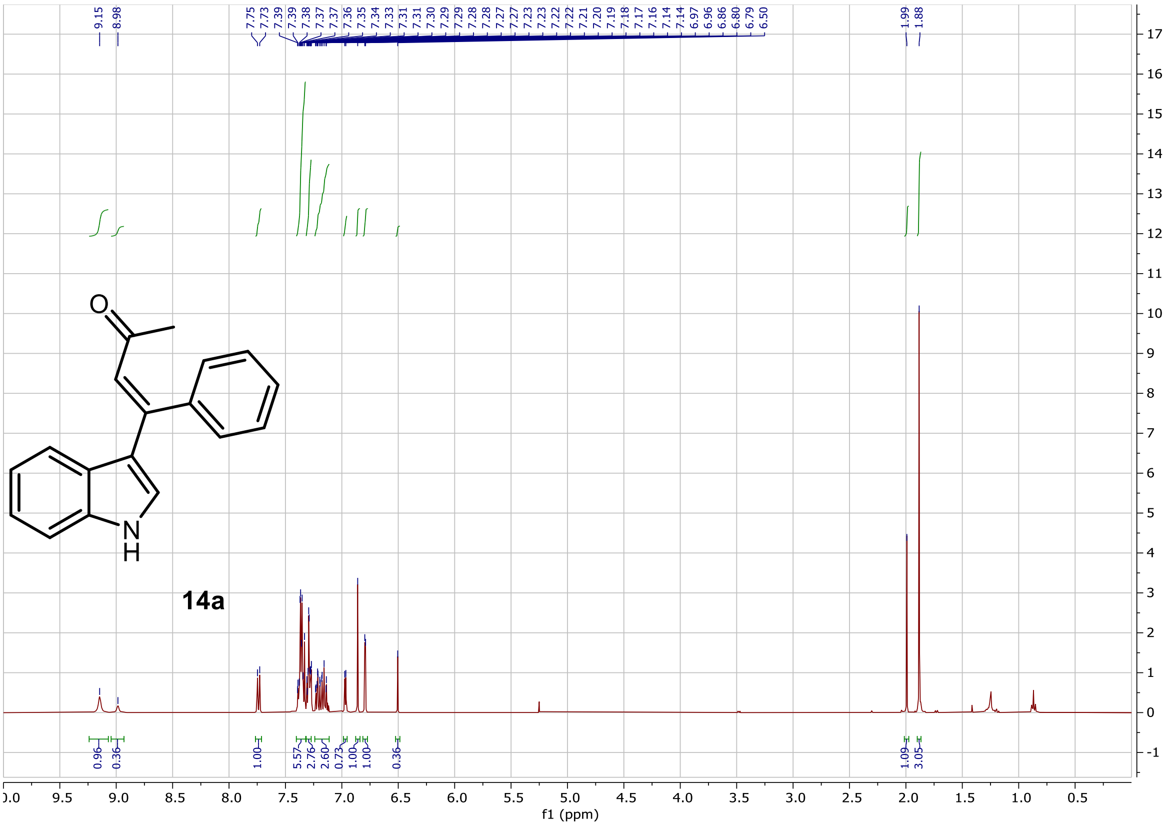

Figure S9 - ^{1}H NMR spectrum of **14a** recorded in $CDCl_3$ solution.

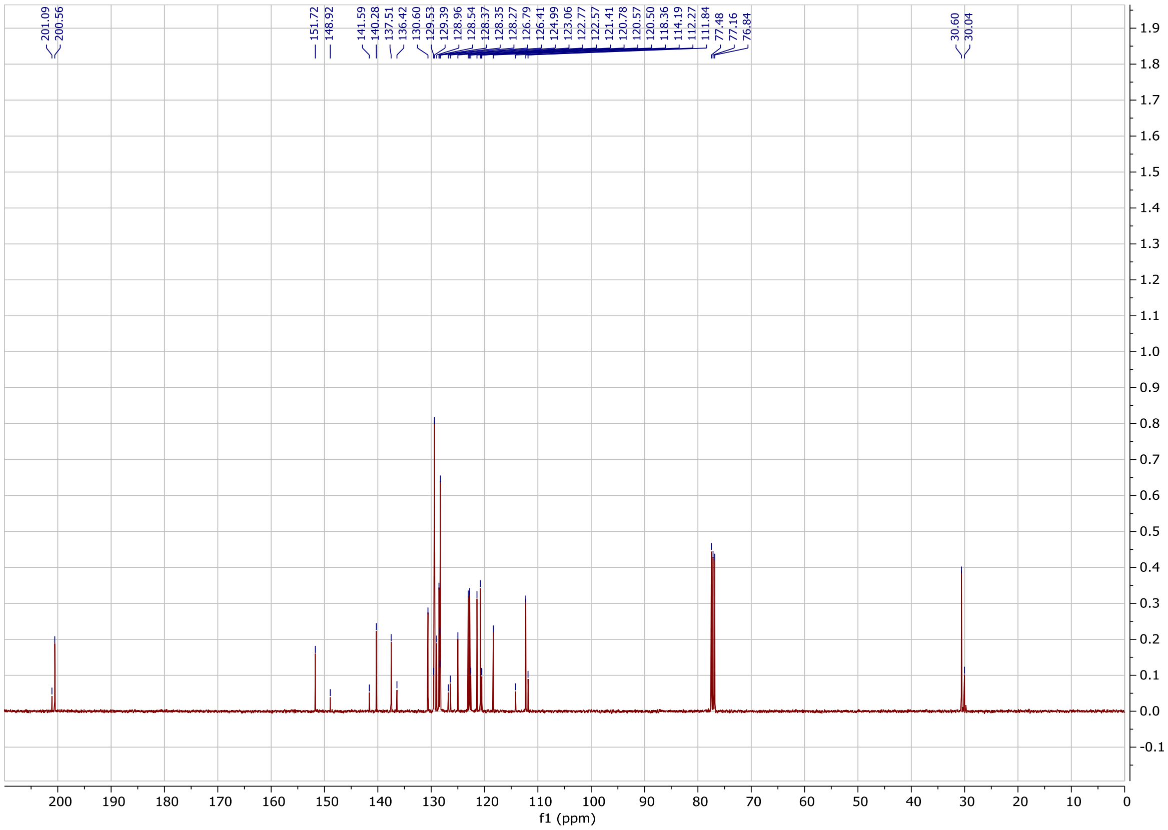

Figure S10 - $^{13}C\{^{1}H\}$ NMR spectrum of **14a** recorded in $CDCl_3$ solution.

16

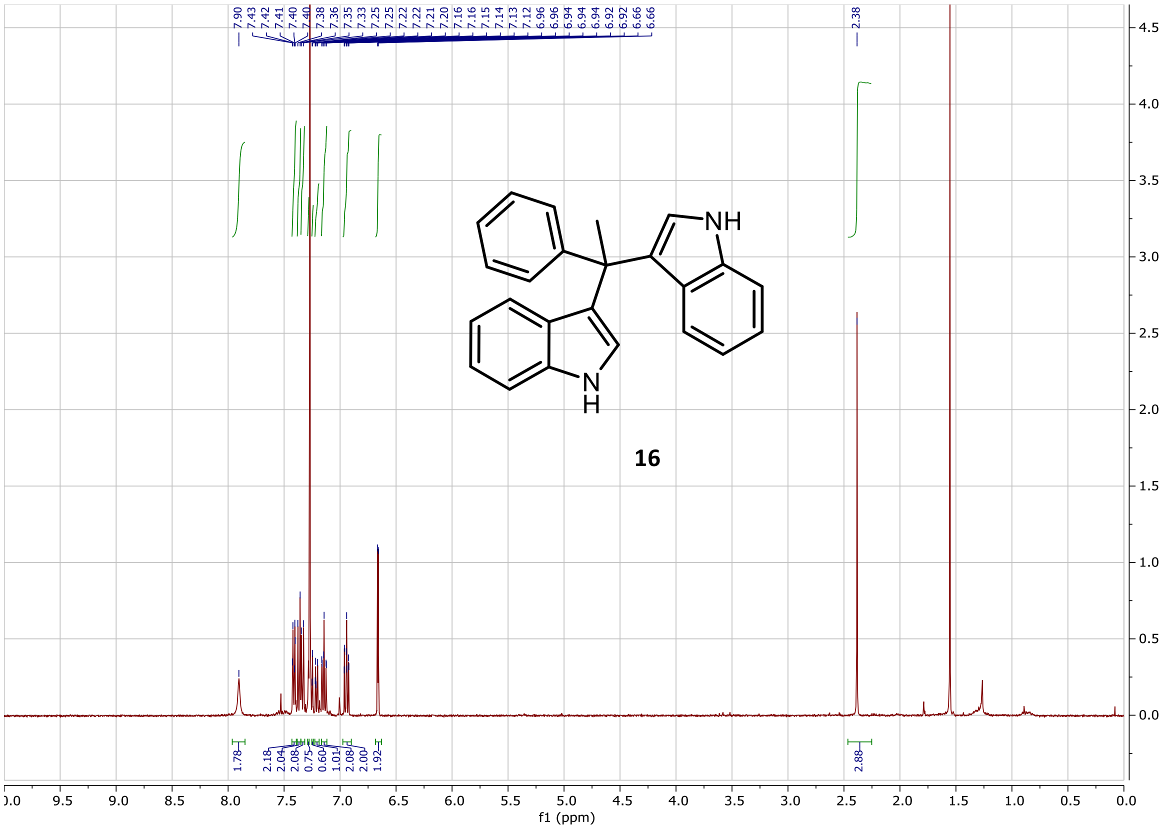

Figure S11 - ^{1}H NMR spectrum of **16** recorded in $CDCl_3$ solution.

17

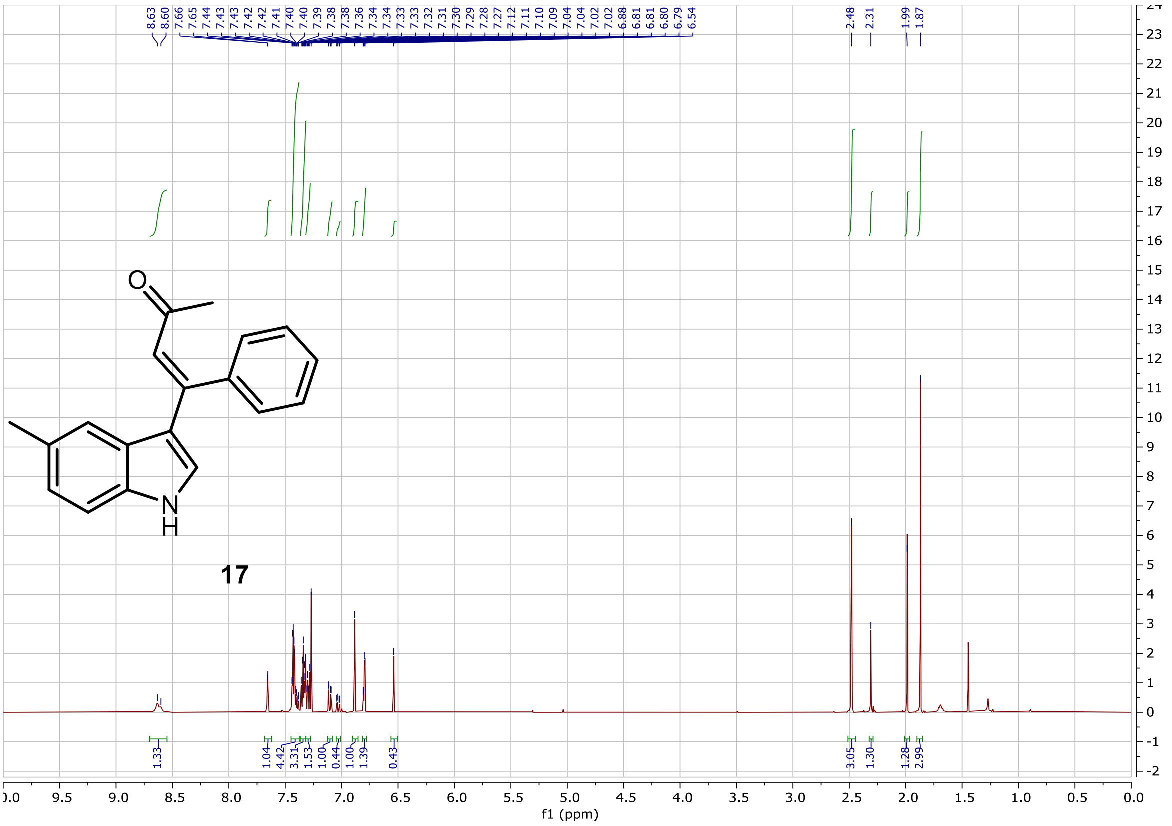

Figure S12 - ^{1}H NMR spectrum of **17** recorded in $CDCl_3$ solution.

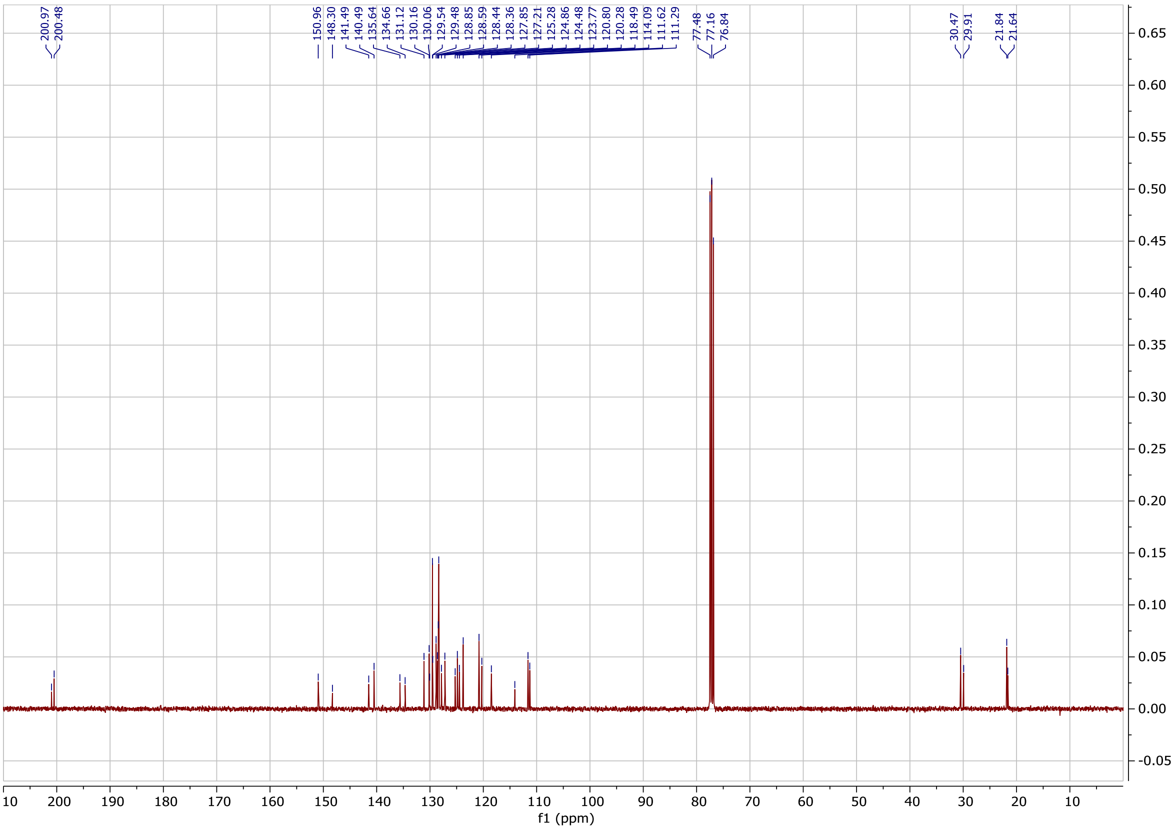

Figure S13 - $^{13}C\{^{1}H\}$ NMR spectrum of **17** recorded in $CDCl_3$ solution.

18

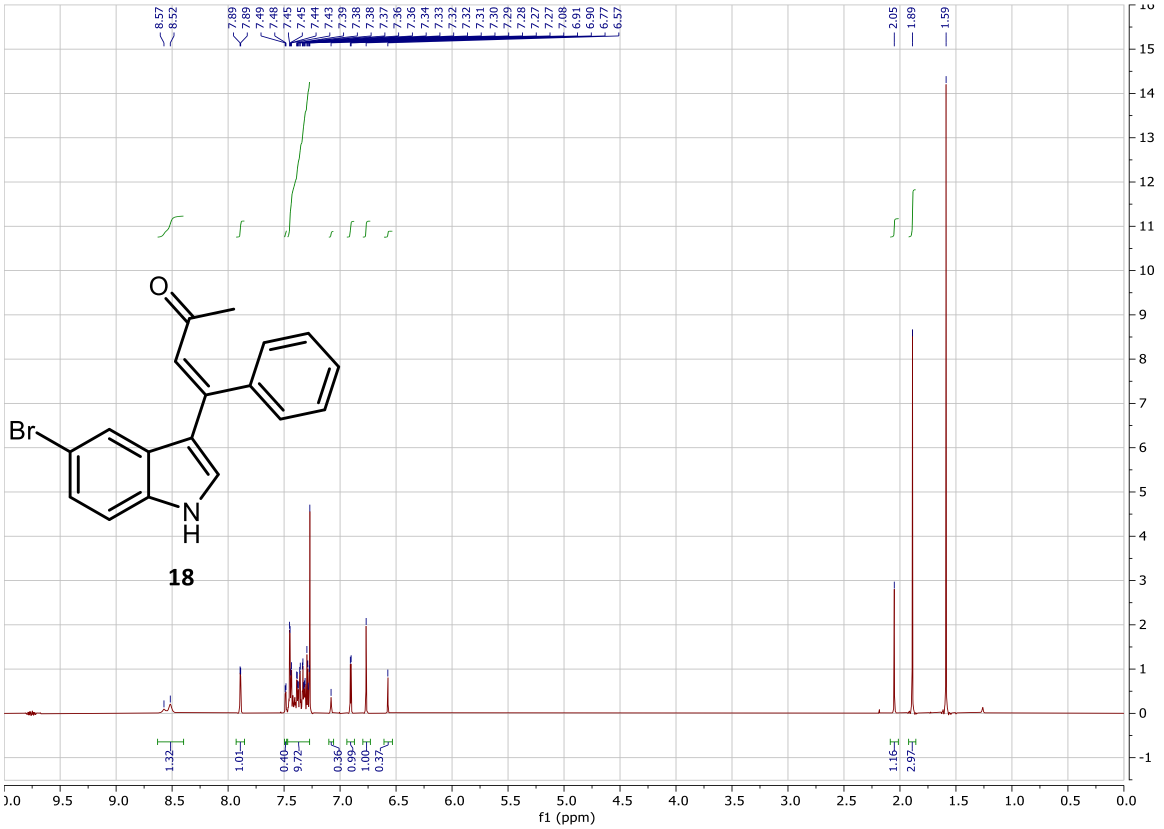

Figure S14 - ^{1}H NMR spectrum of **18** recorded in $CDCl_3$ solution.

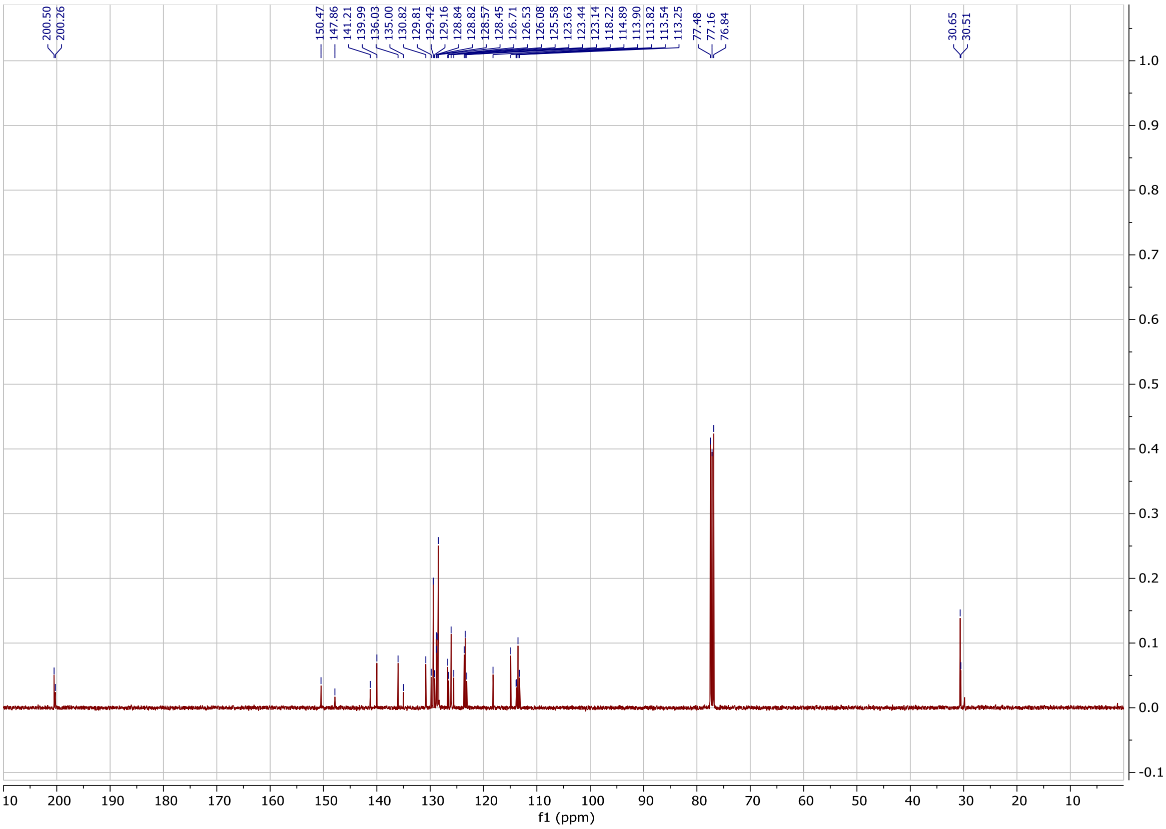

Figure S15 - $^{13}C\{^{1}H\}$ NMR spectrum of **18** recorded in $CDCl_3$ solution.

19

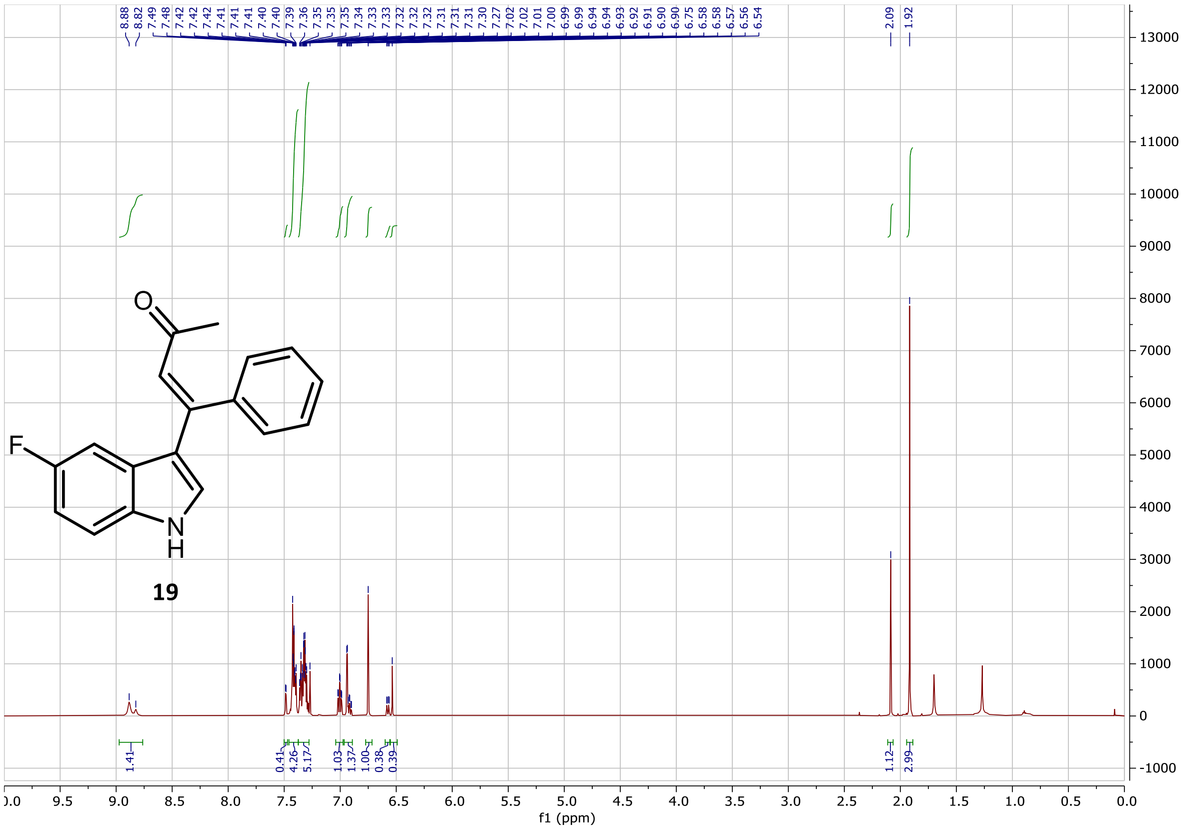

Figure S16 - ^{1}H NMR spectrum of **19** recorded in $CDCl_3$ solution.

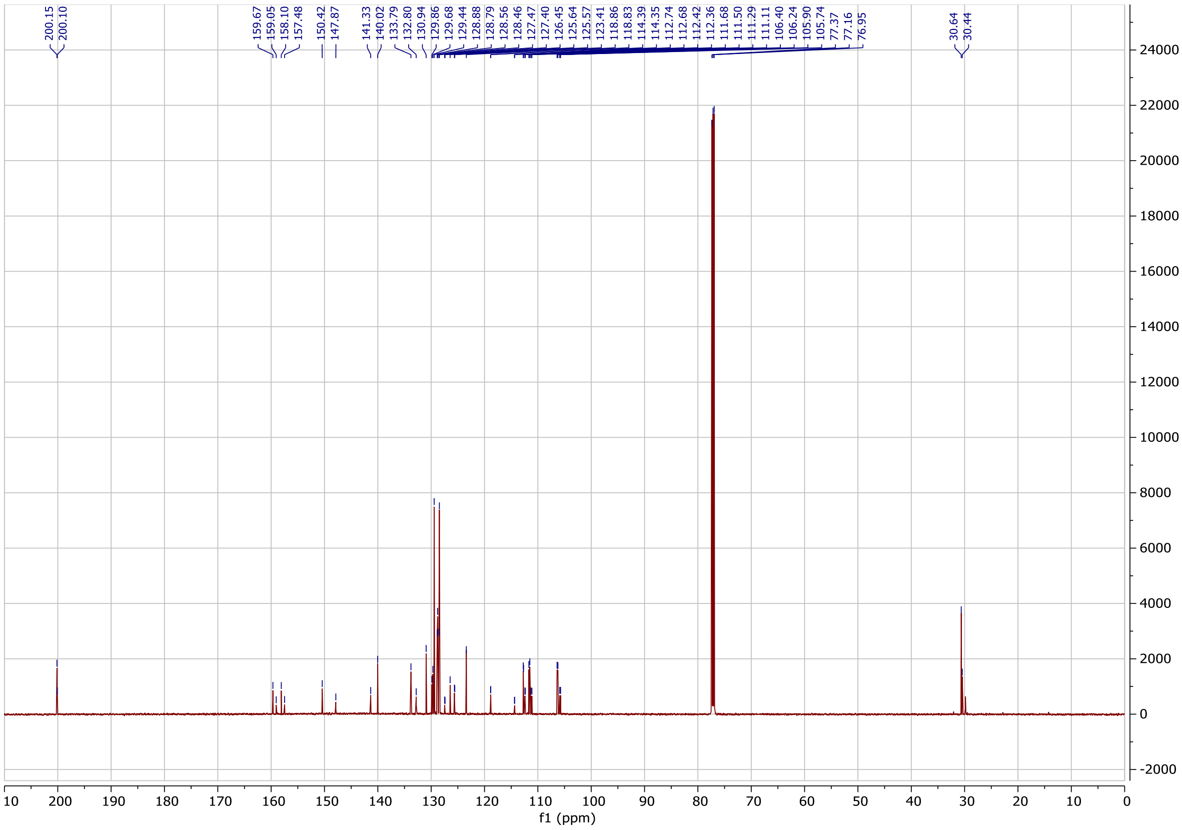

Figure S17 – $^{13}C\{^{1}H\}$ NMR spectrum of **19** recorded in $CDCl_3$ solution.

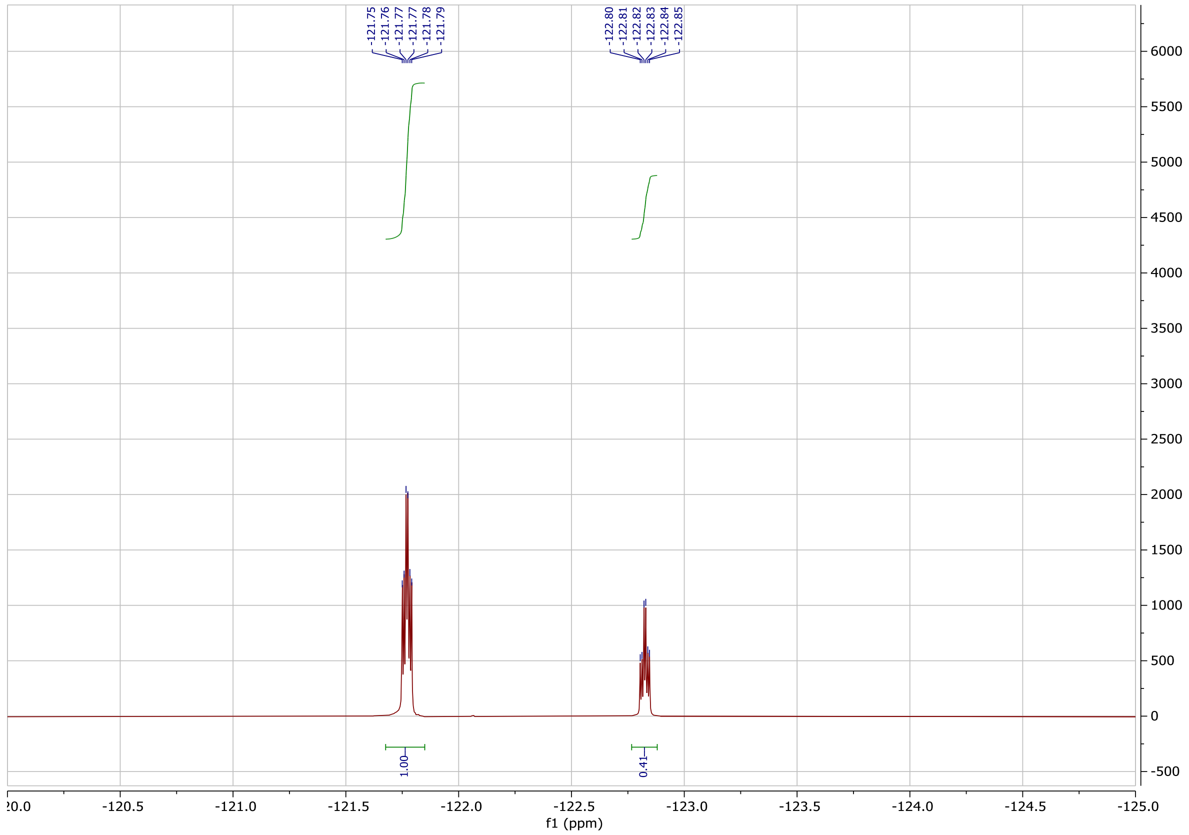

Figure S18 - ^{19}F NMR spectrum of **19** recorded in $CDCl_3$ solution.

20

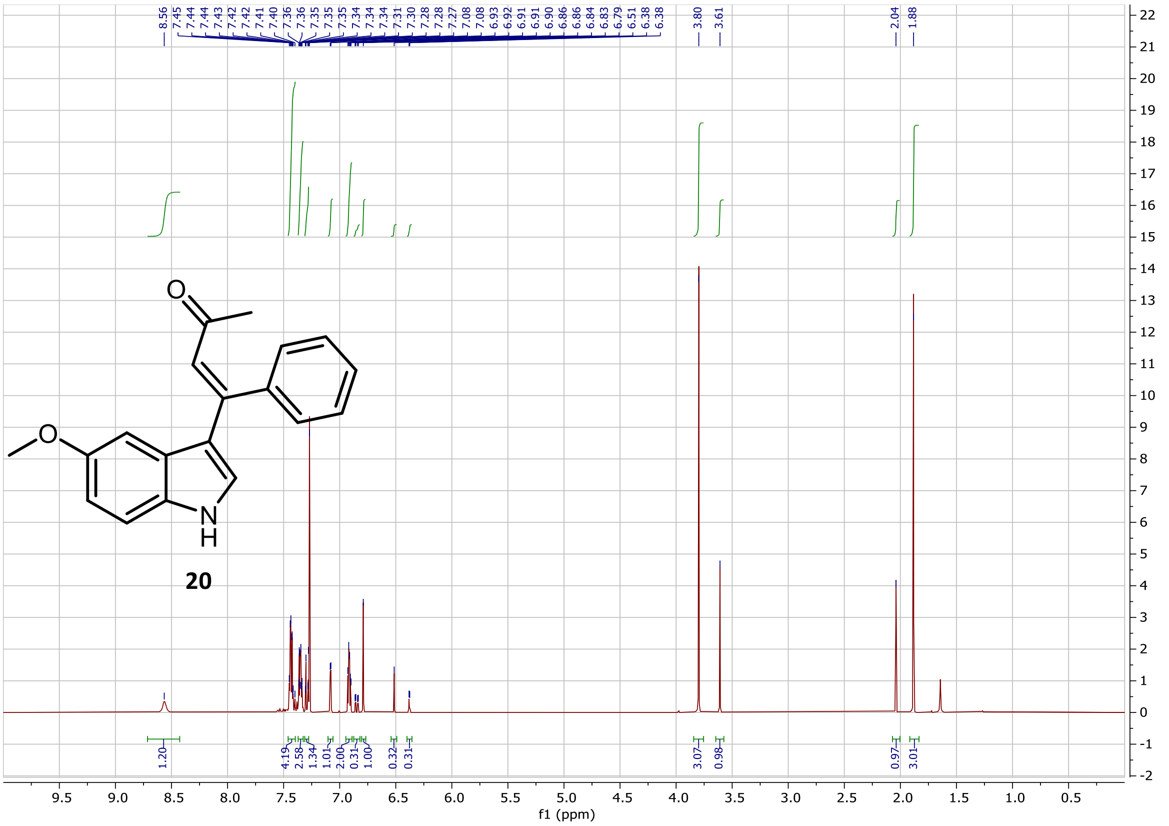

Figure S19 - ^{1}H NMR spectrum of **20** recorded in $CDCl_3$ solution.

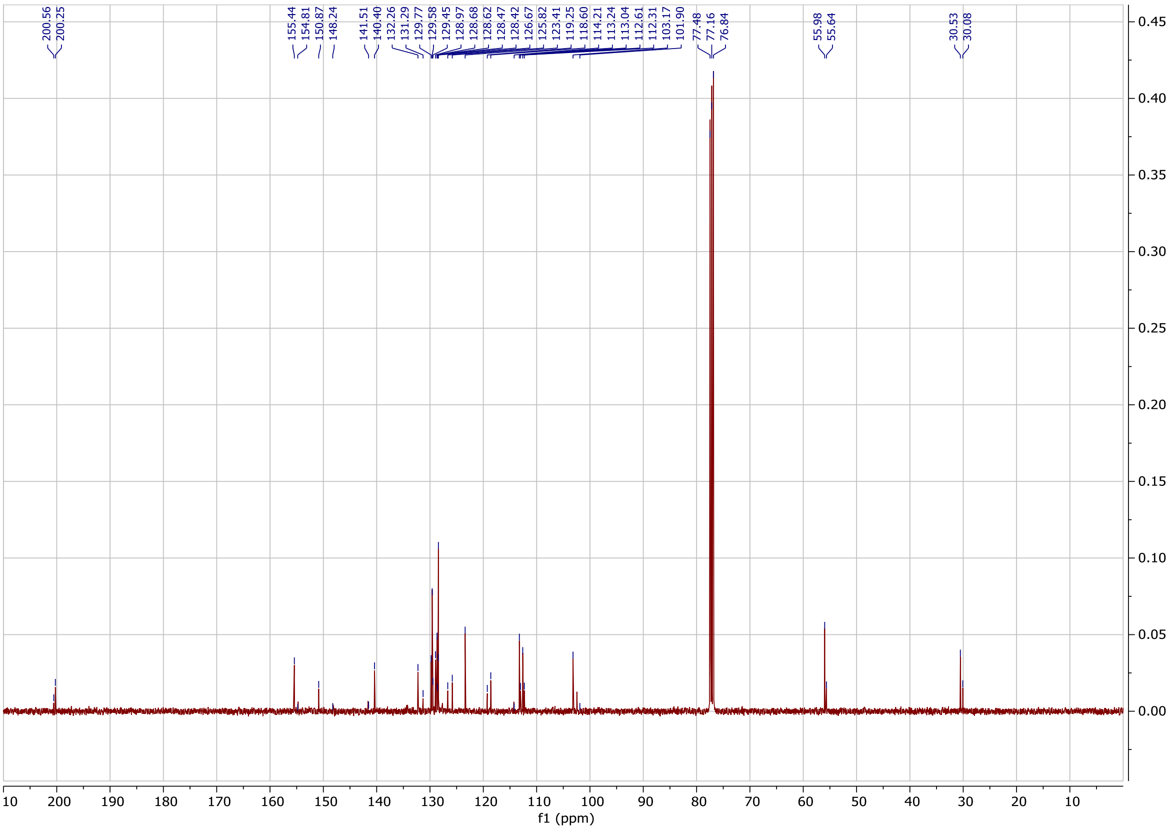

Figure S20 - $^{13}C\{^{1}H\}$ NMR spectrum of **20** recorded in $CDCl_3$ solution.

21

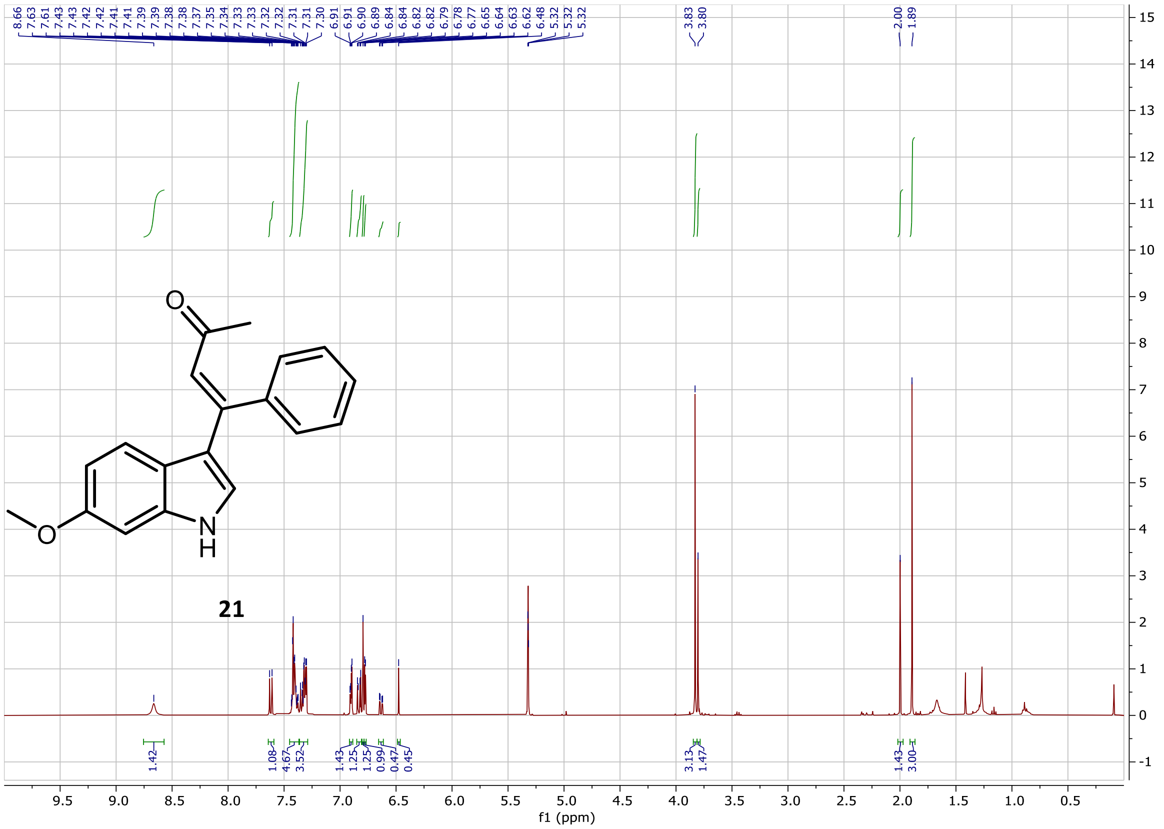

Figure S21 - ^{1}H NMR spectrum of **21** recorded in CD_2Cl_2 solution.

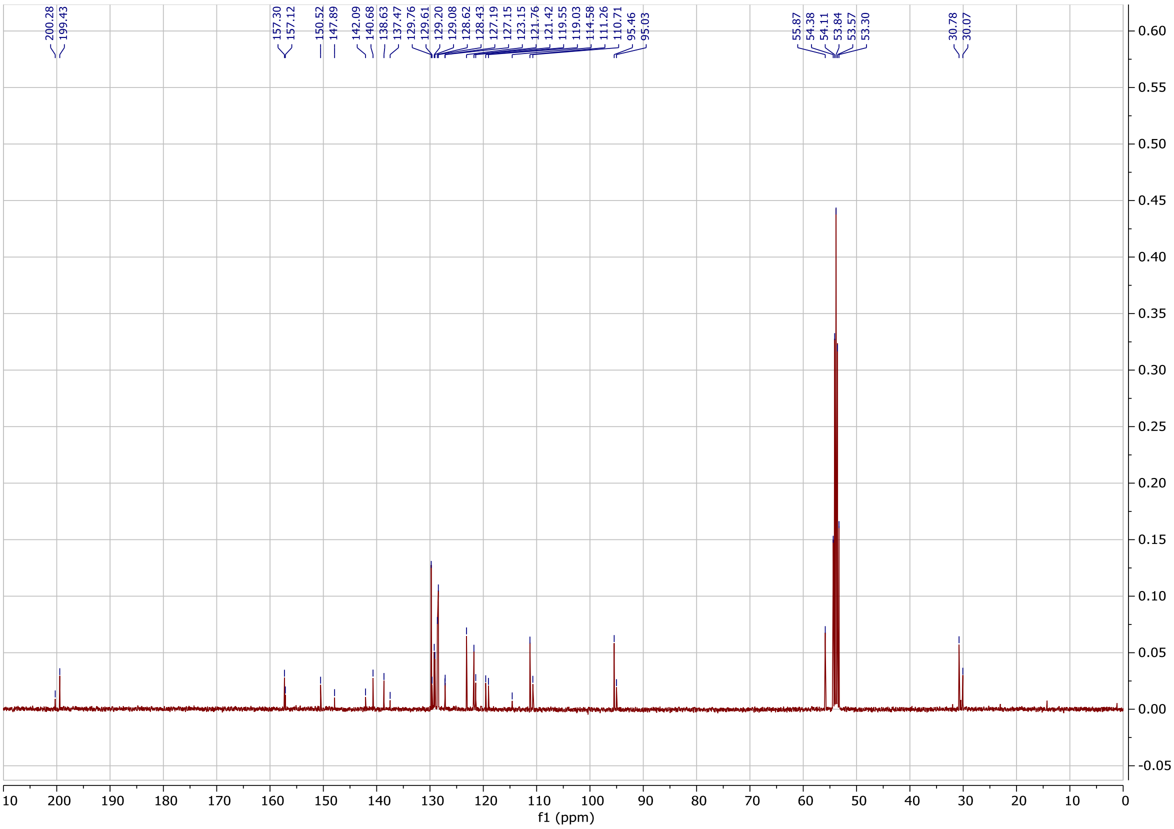

Figure S22 - $^{13}C\{^{1}H\}$ NMR spectrum of **21** recorded in CD_2Cl_2 solution.

22

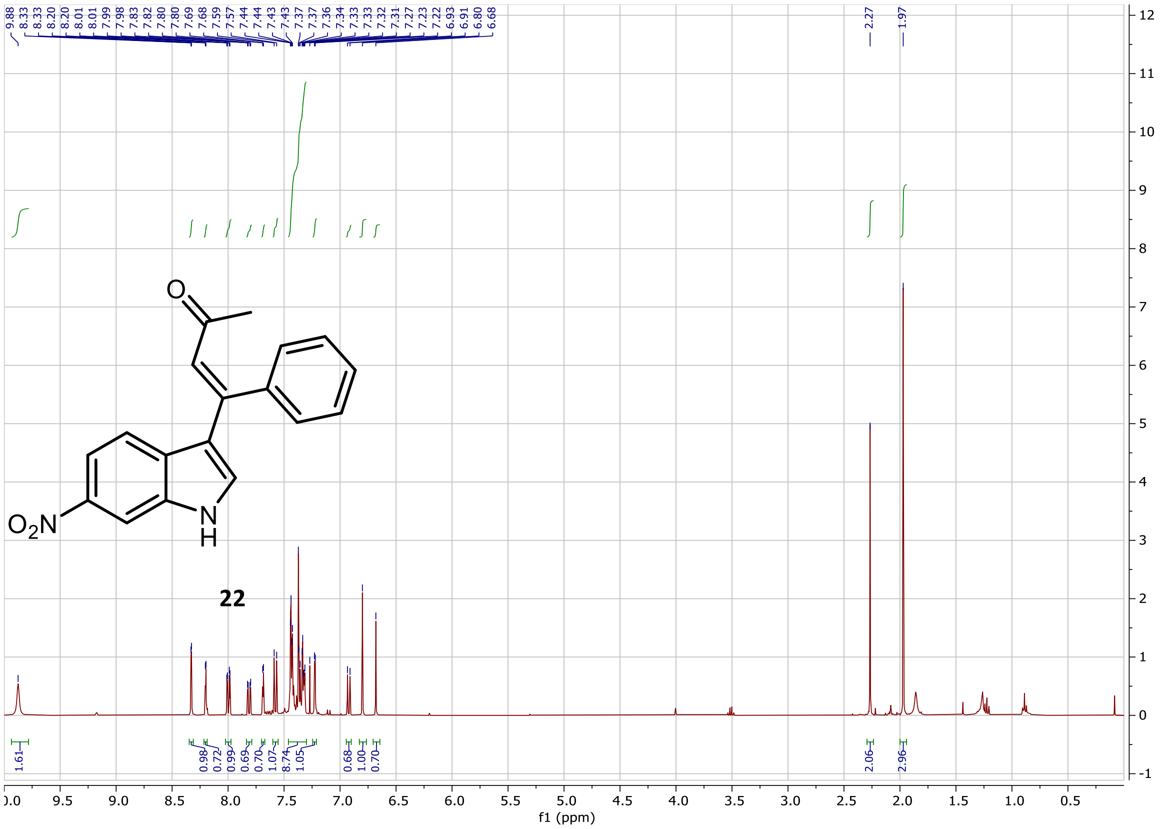

Figure S23 - ^{1}H NMR spectrum of **22** recorded in $CDCl_3$ solution.

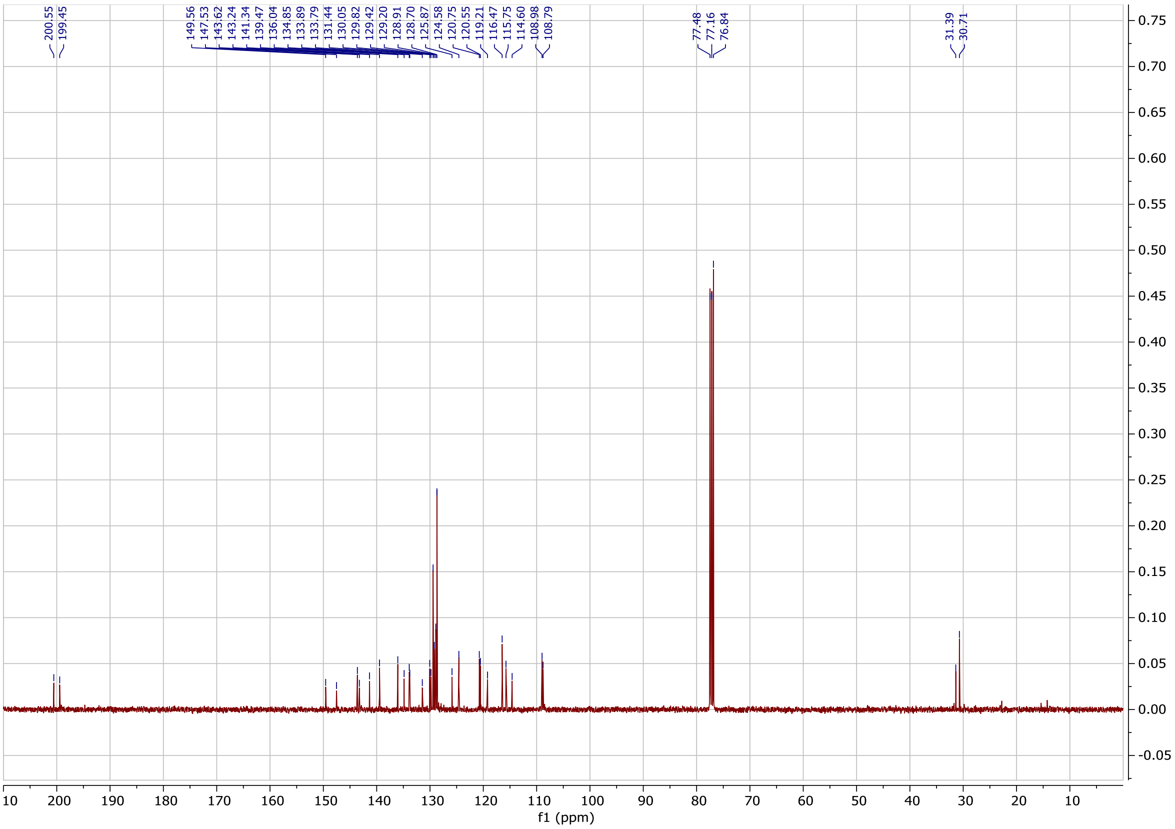

Figure S24 - $^{13}C\{^{1}H\}$ NMR spectrum of **22** recorded in $CDCl_3$ solution.

23

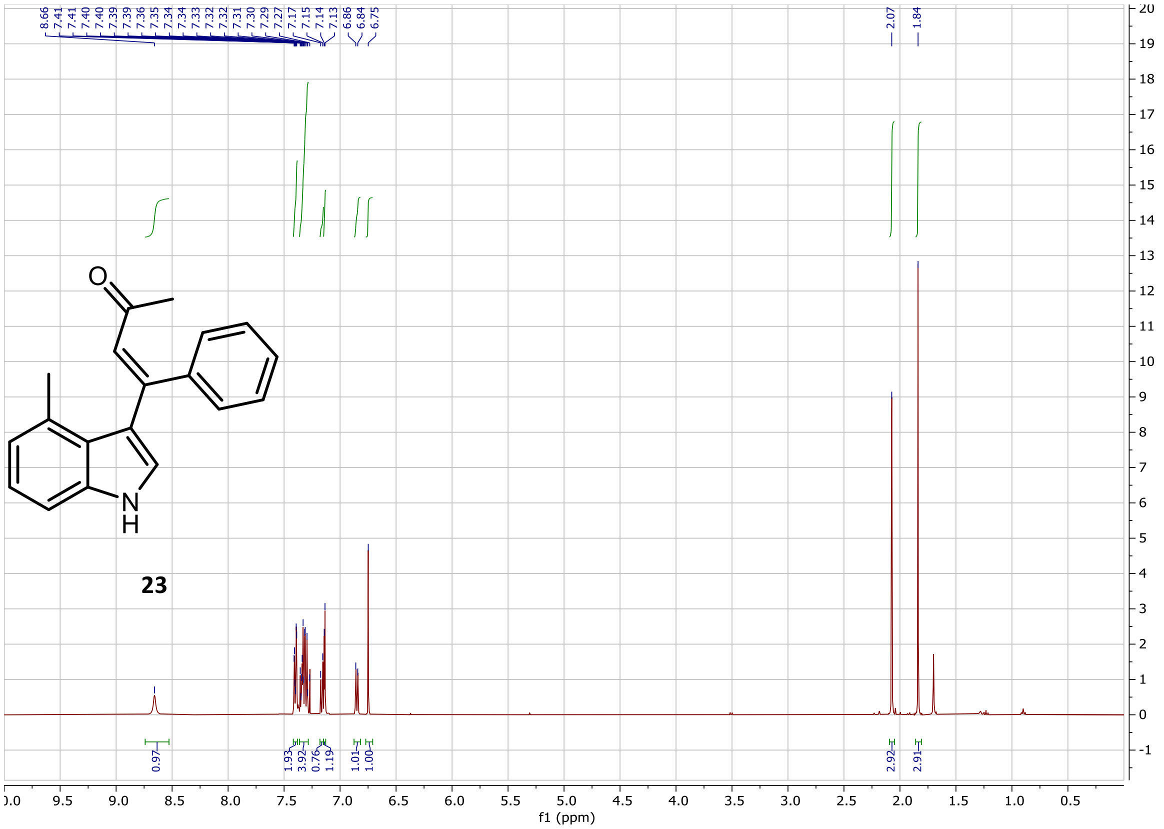

Figure S25 - ^{1}H NMR spectrum of **23** recorded in $CDCl_3$ solution.

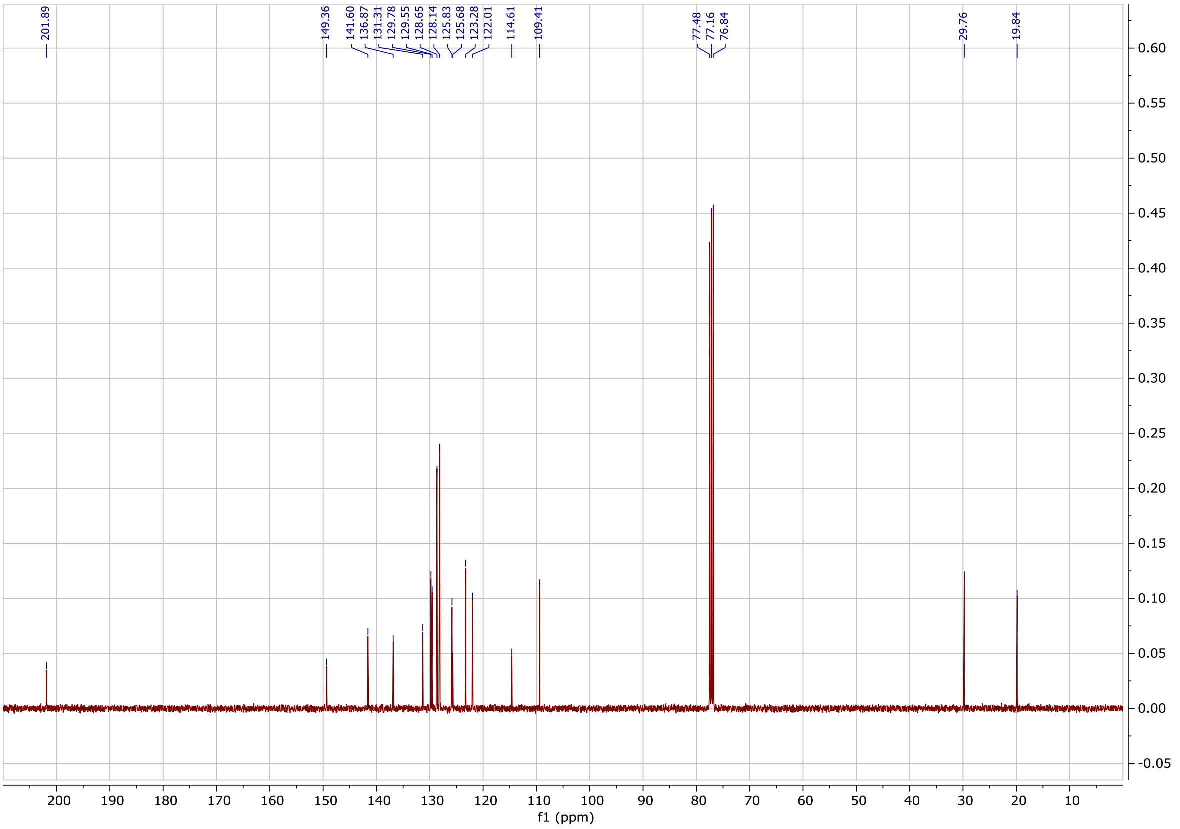

Figure S26 - ^{13}C{^{1}H} NMR spectrum of **23** recorded in $CDCl_3$ solution.

24

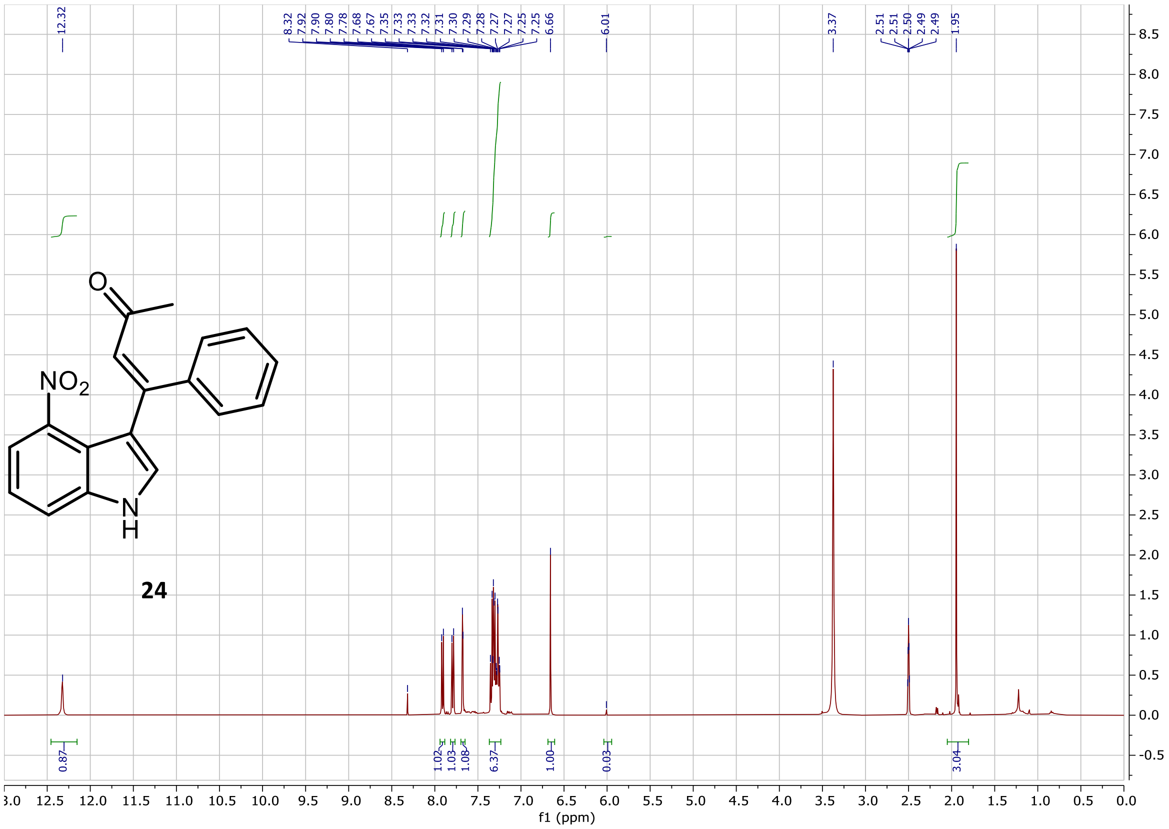

Figure S27 - ^{1}H NMR spectrum of **24** recorded in *d*6-DMSO solution.

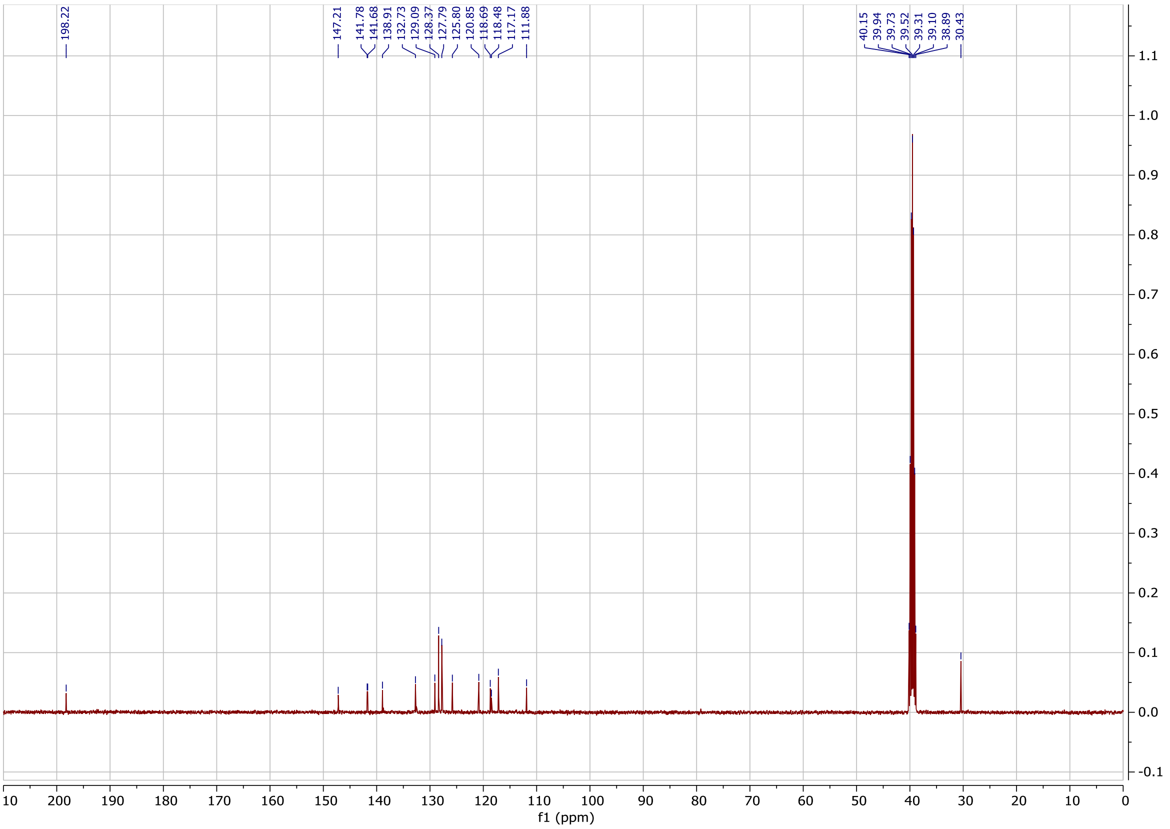

Figure S28 - $^{13}C\{^{1}H\}$ NMR spectrum of **24** recorded in *d*6-DMSO solution.

25

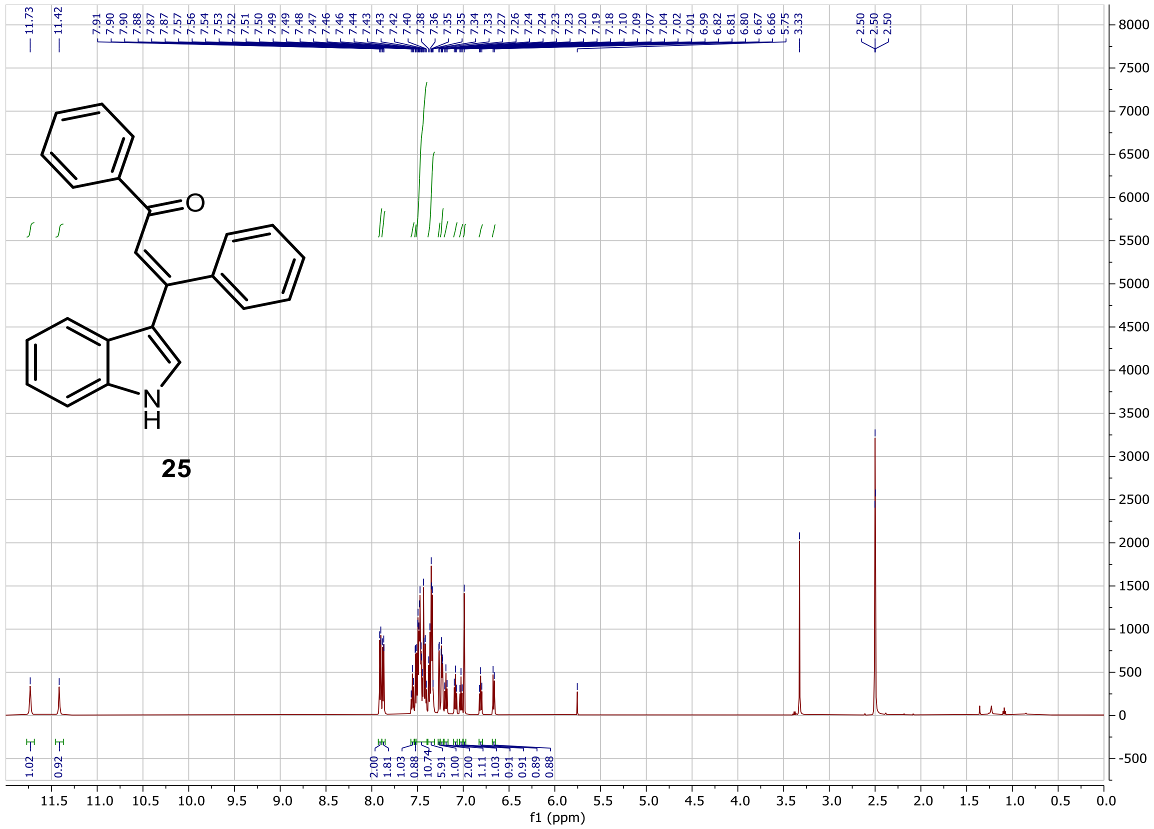

Figure S29 - ^{1}H NMR spectrum of **25** recorded in *d*6-DMSO solution.

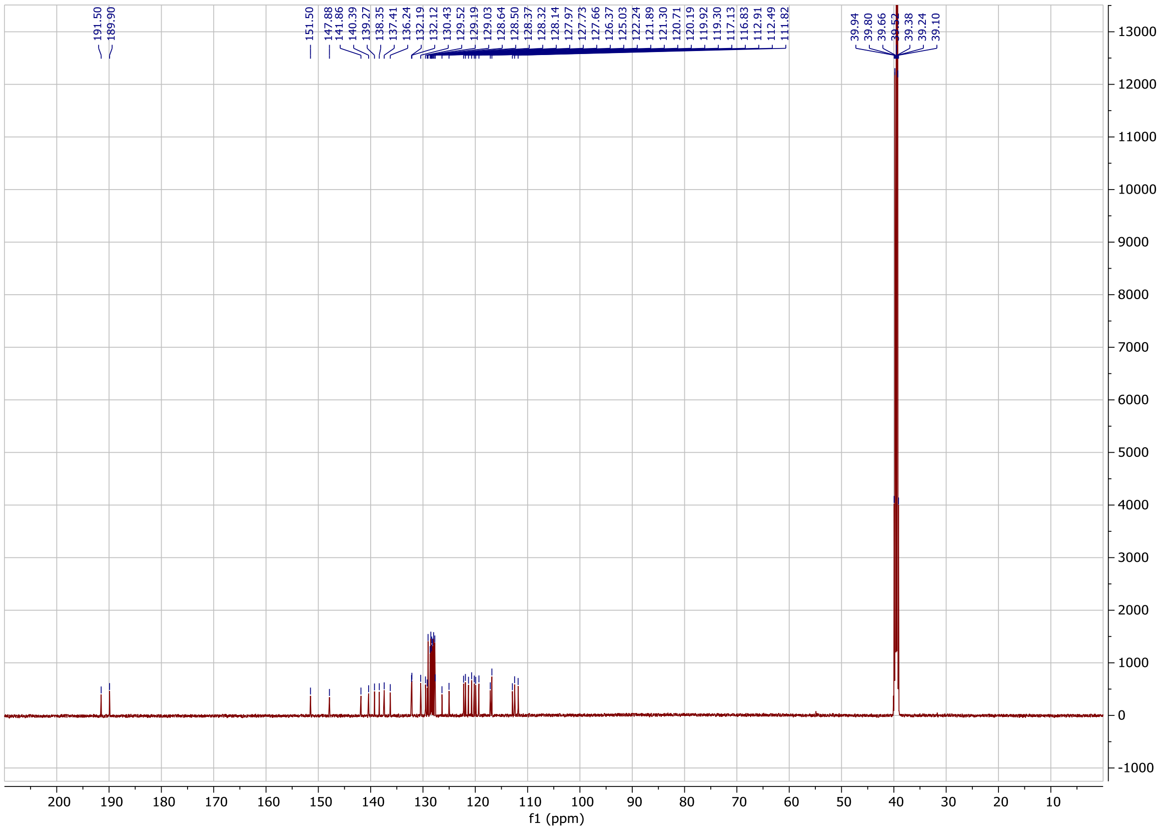

Figure S30 - ^{13}C{^{1}H} NMR spectrum of **25** recorded in *d*6-DMSO solution.

26a

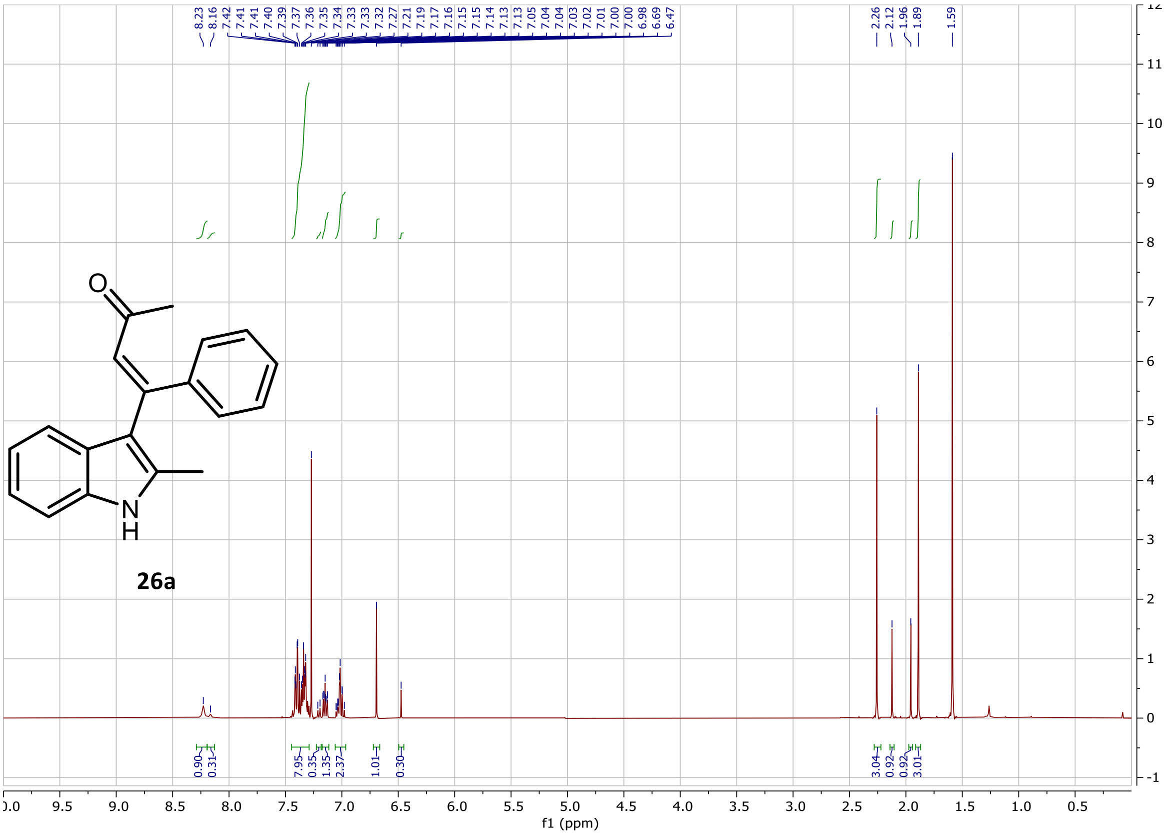

Figure S31 - ^{1}H NMR spectrum of **26a** recorded in $CDCl_3$ solution.

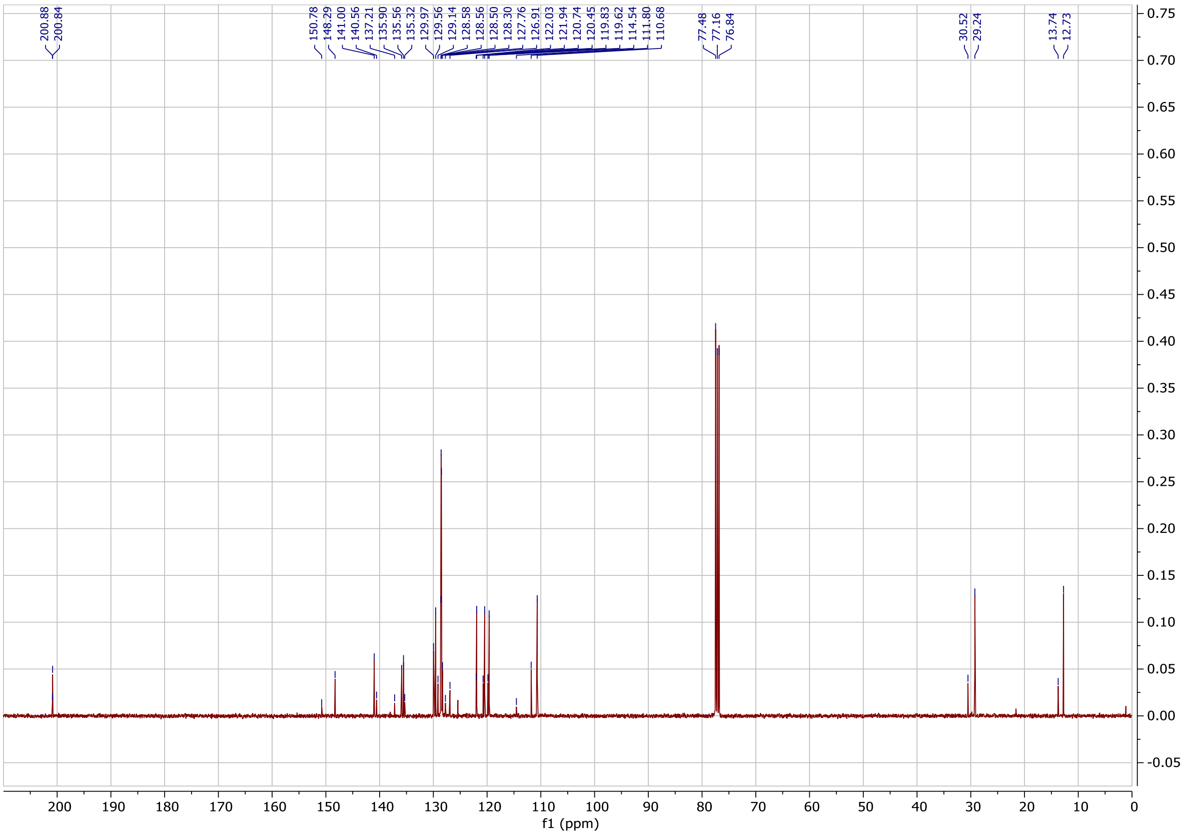

Figure S32 - $^{13}C\{^{1}H\}$ NMR spectrum of **26a** recorded in $CDCl_3$ solution.

26b

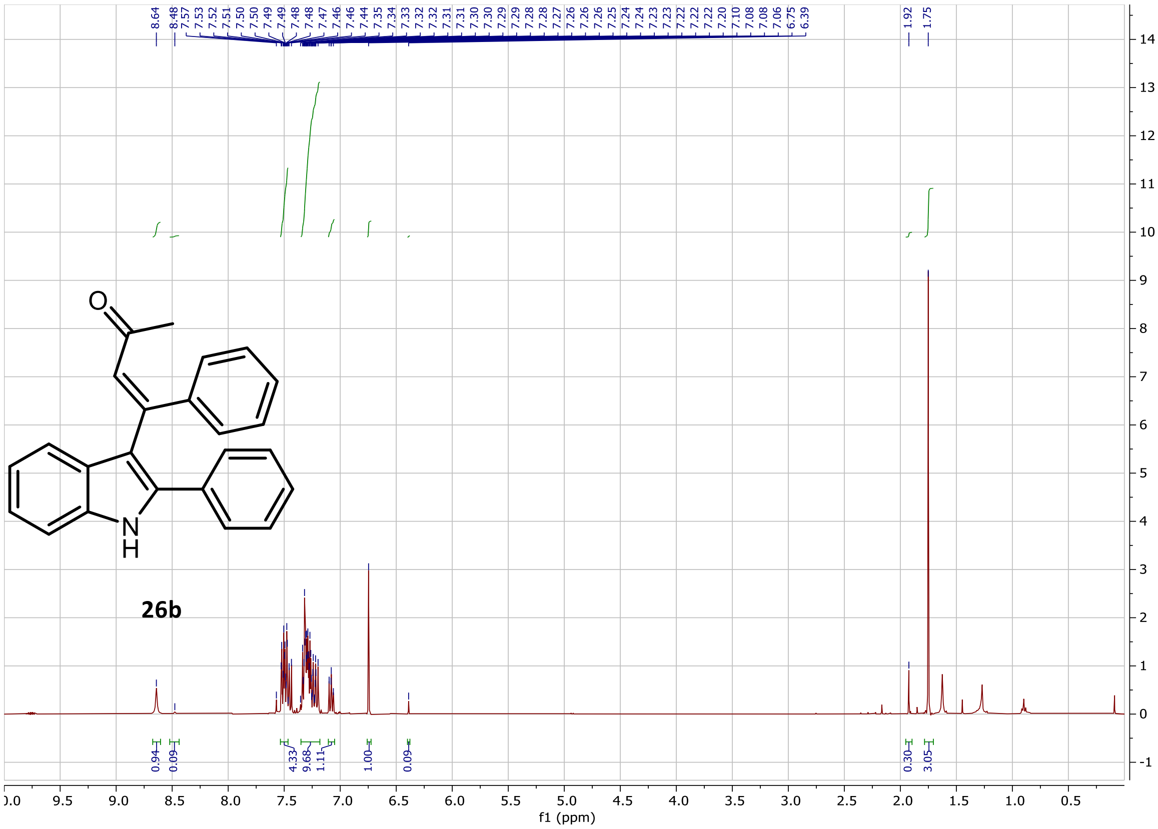

Figure S33 - ^{1}H NMR spectrum of **26b** recorded in $CDCl_3$ solution.

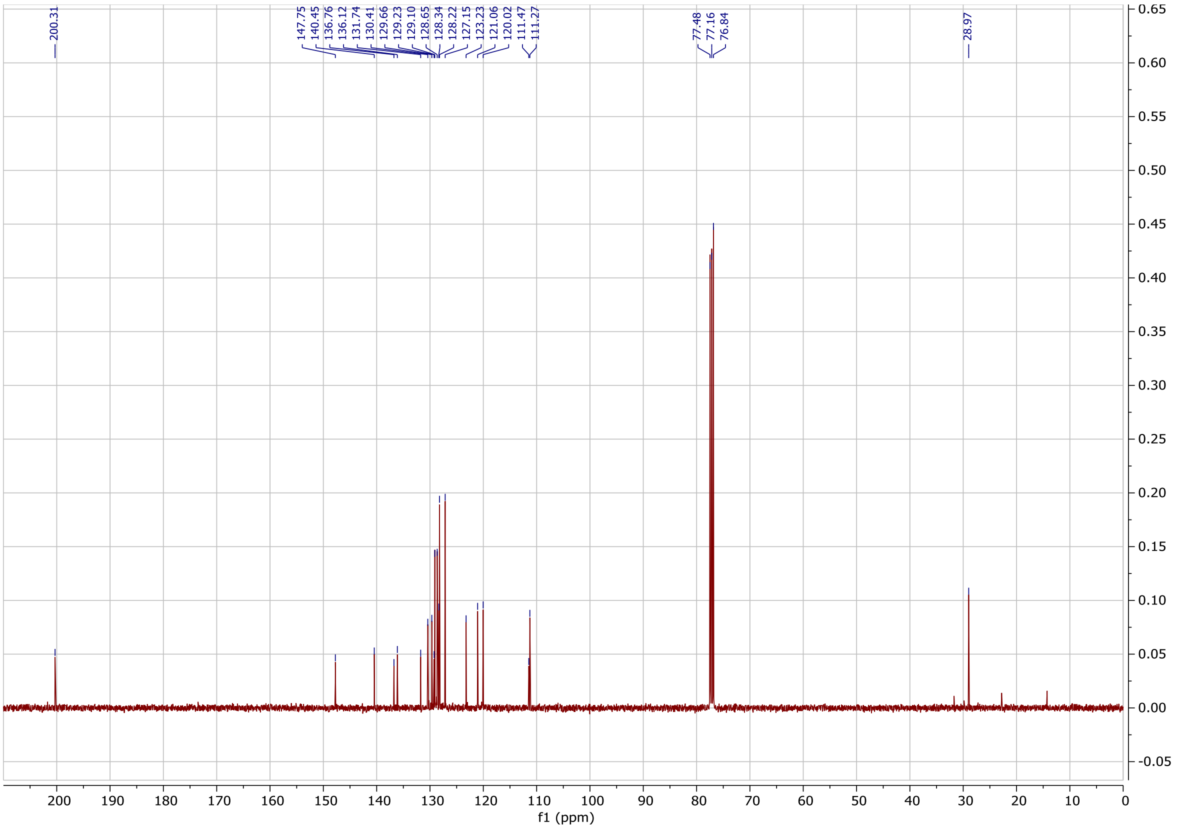

Figure S34 - $^{13}C\{^{1}H\}$ NMR spectrum of **26b** recorded in $CDCl_3$ solution.

27

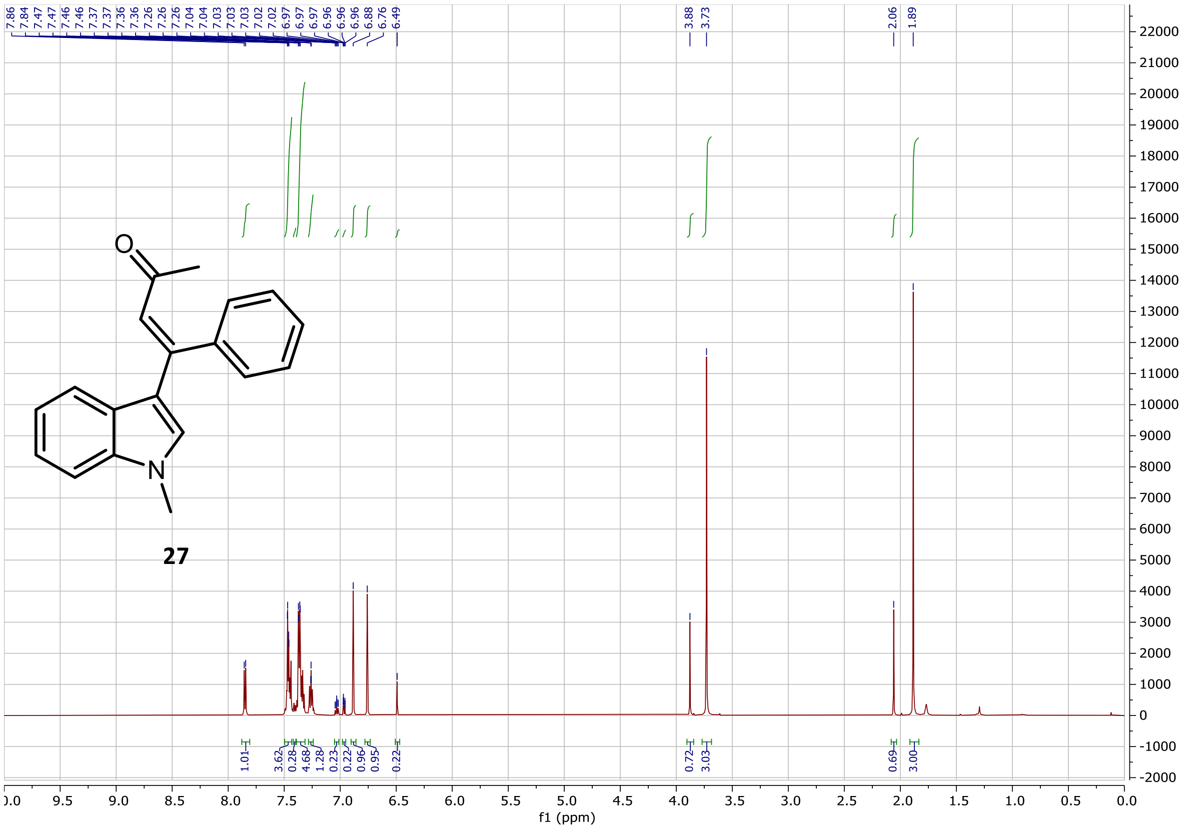

Figure S35 - ^{1}H NMR spectrum of **27** recorded in $CDCl_3$ solution.

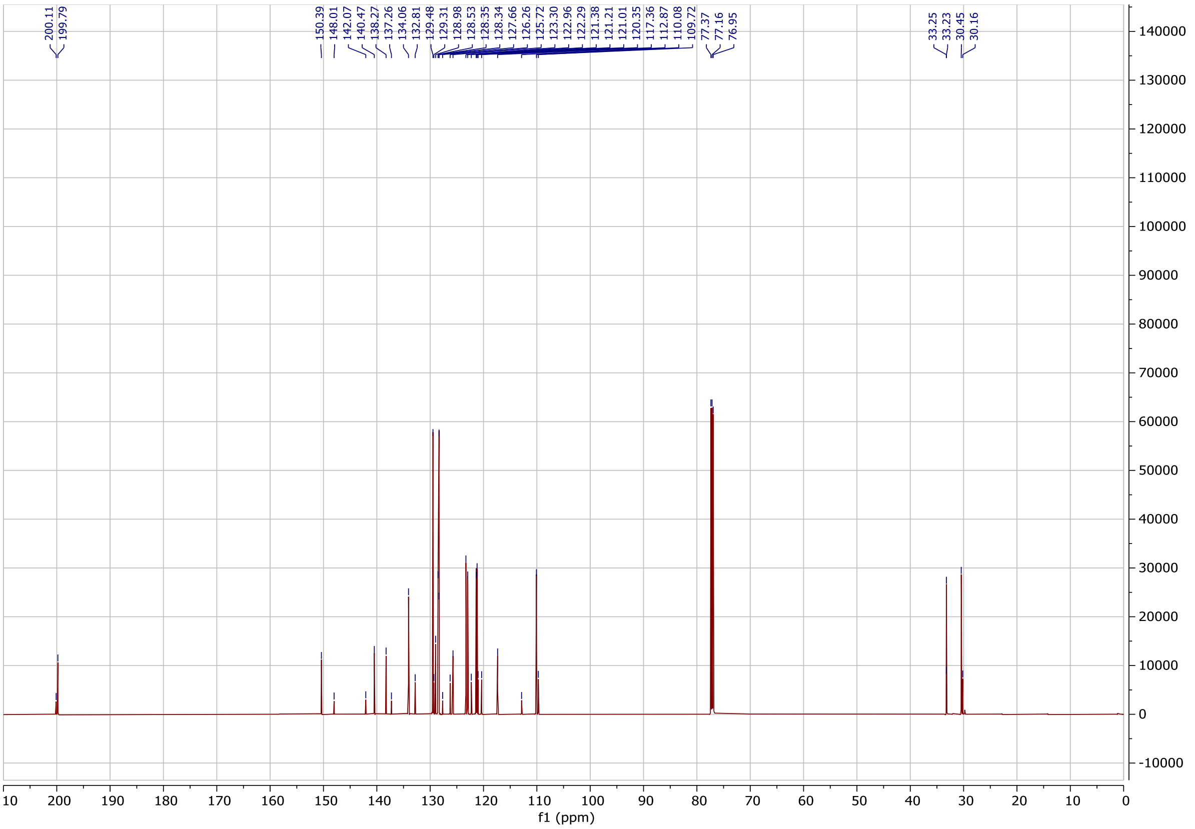

Figure S36 - $^{13}C\{^{1}H\}$ NMR spectrum of **27** recorded in $CDCl_3$ solution.

28

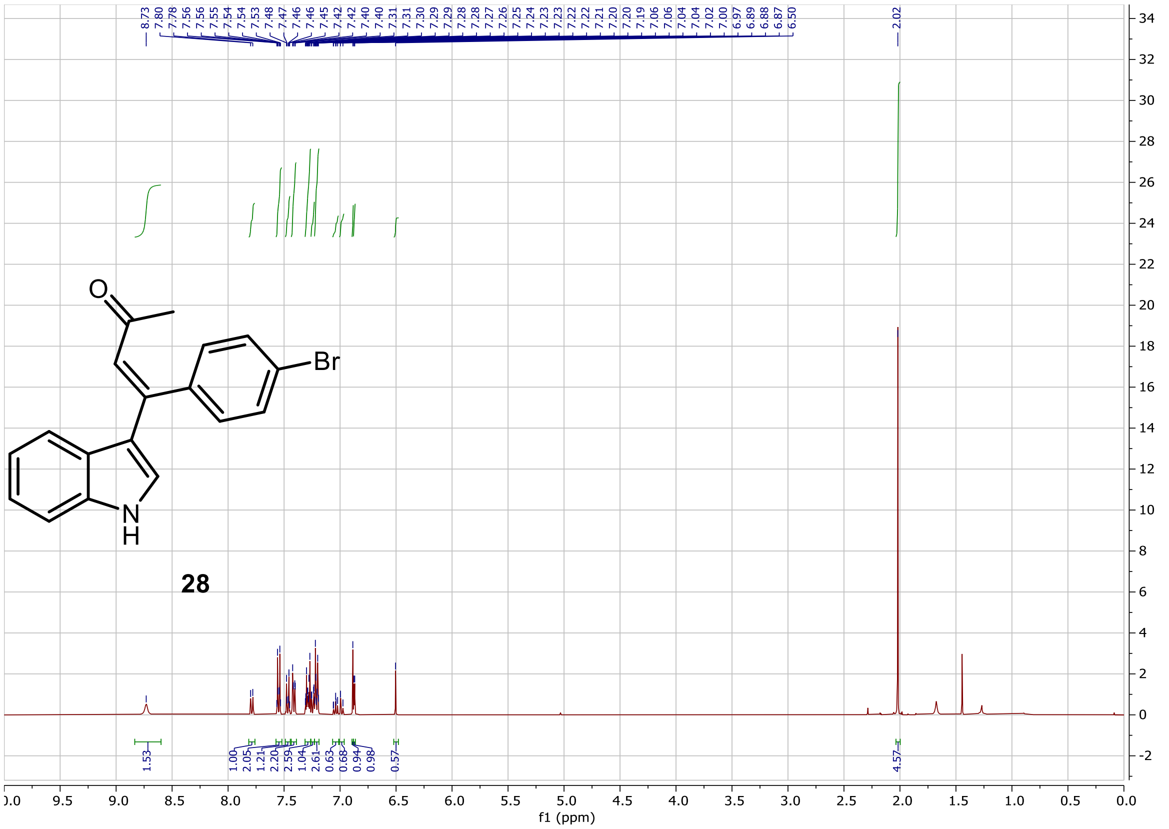

Figure S37 - ^{1}H NMR spectrum of **28** recorded in $CDCl_3$ solution.

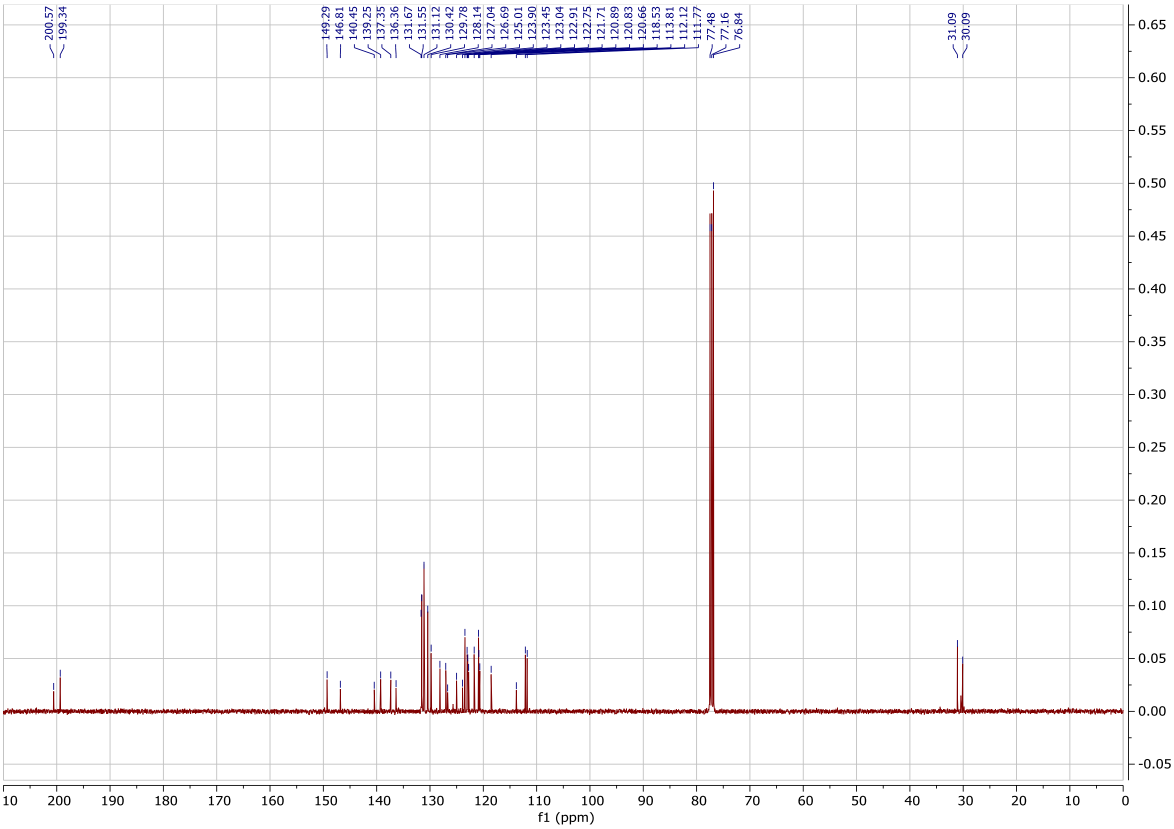

Figure S38 - $^{13}C\{^{1}H\}$ NMR spectrum of **28** recorded in $CDCl_3$ solution.

29

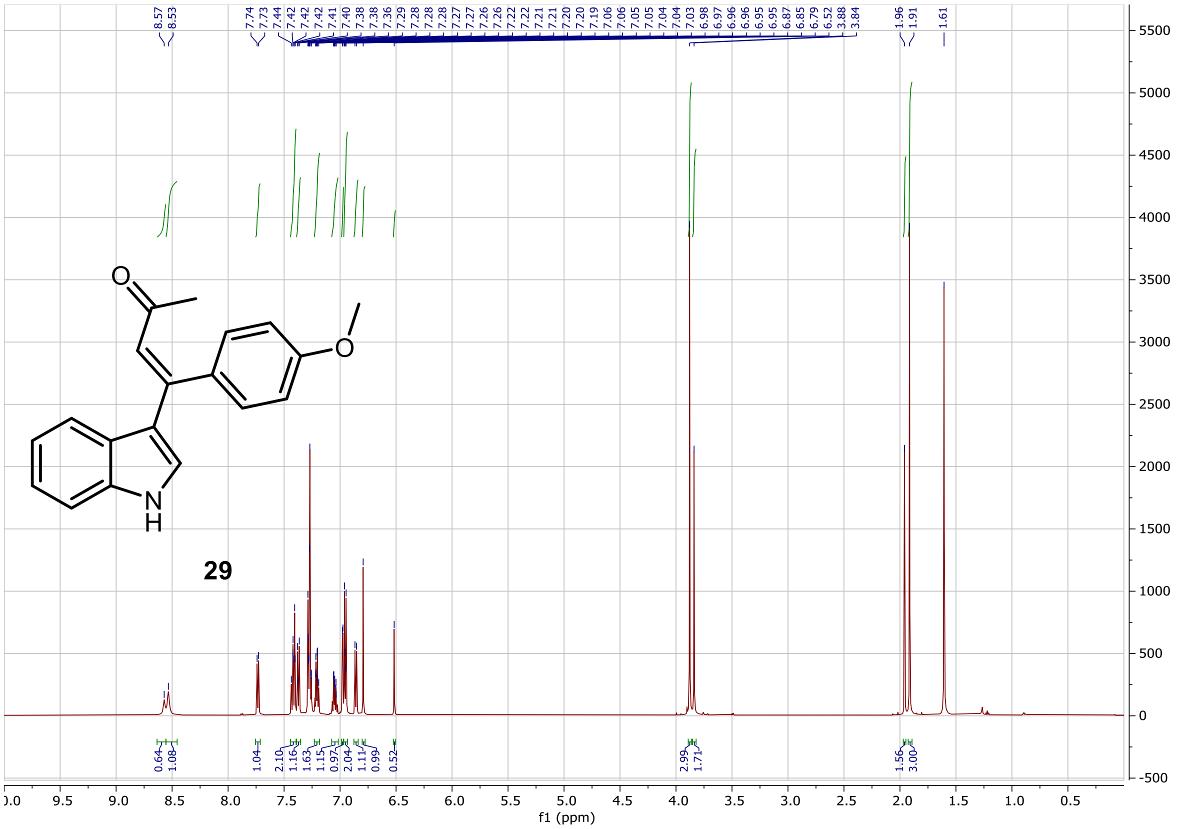

Figure S39 - ^{1}H NMR spectrum of **29** recorded in $CDCl_3$ solution.

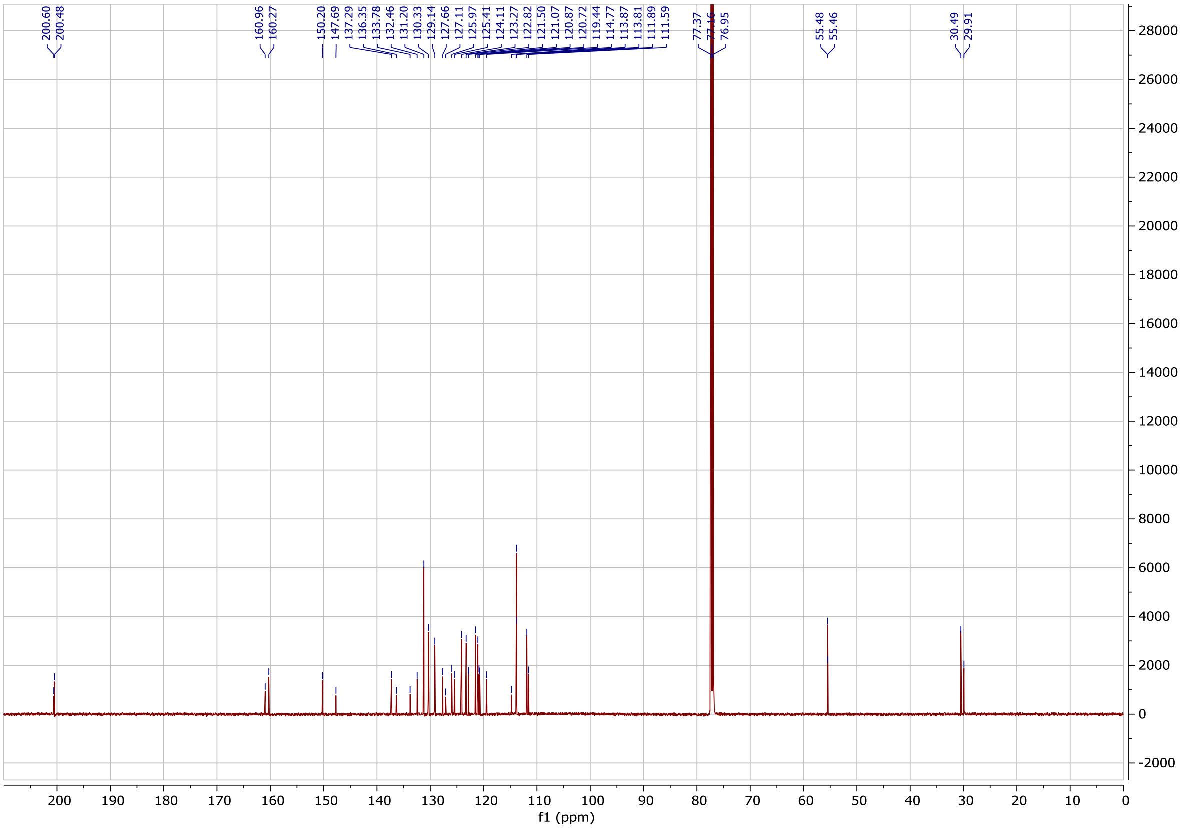

Figure S40 - $^{13}C\{^{1}H\}$ NMR spectrum of **29** recorded in $CDCl_3$ solution.

30

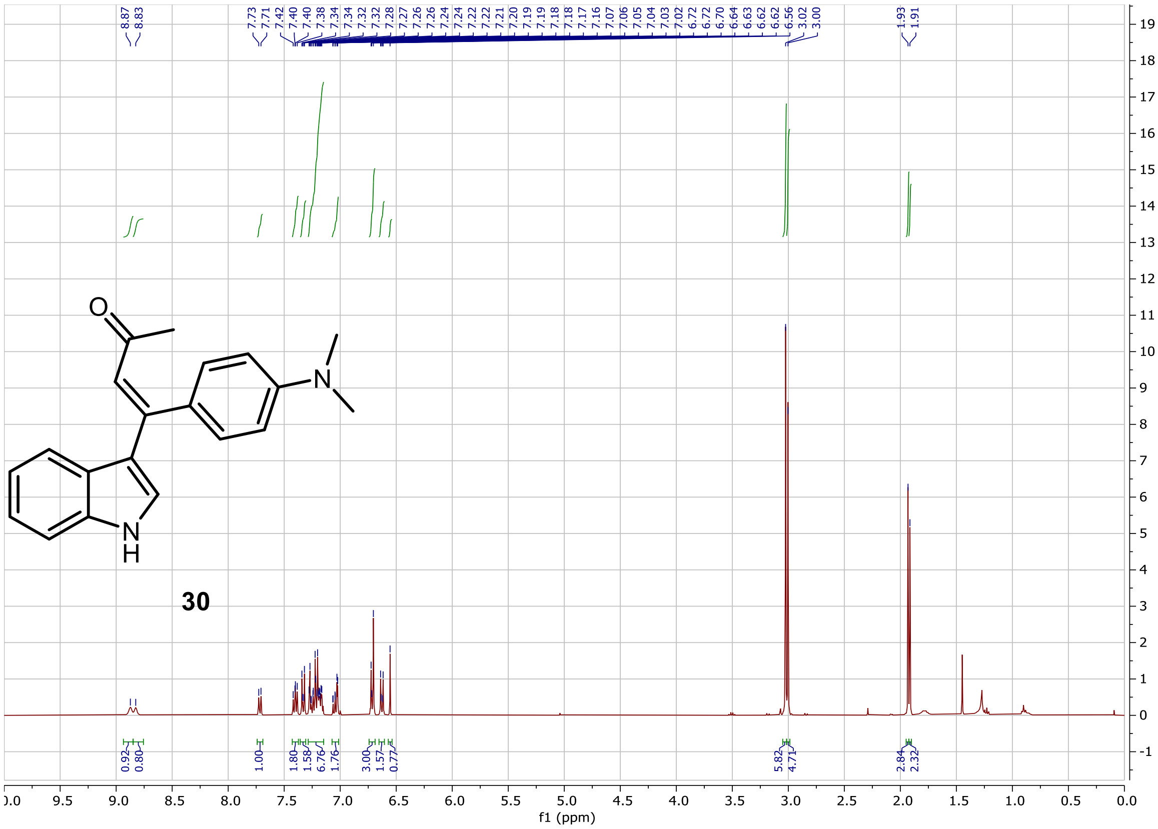

Figure S41 - ^{1}H NMR spectrum of **30** recorded in $CDCl_3$ solution.

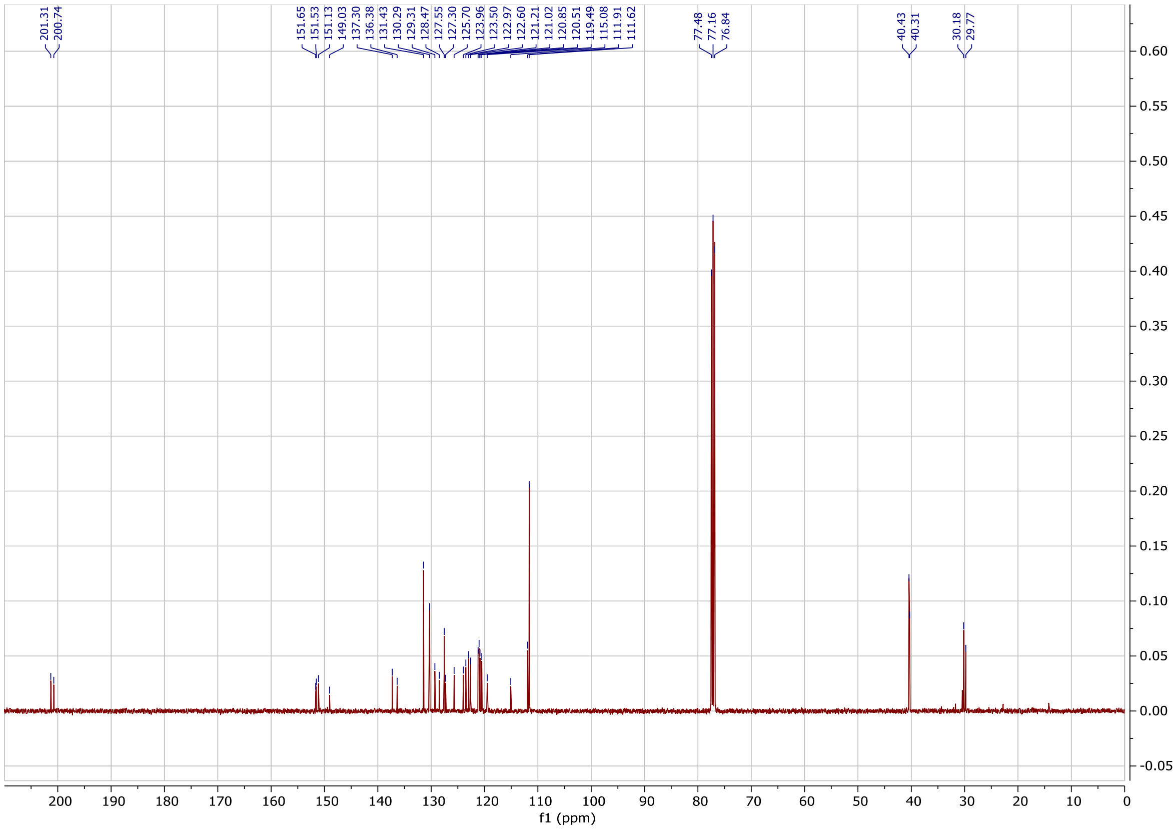

Figure S42 - $^{13}C\{^{1}H\}$ NMR spectrum of **30** recorded in $CDCl_3$ solution.

32

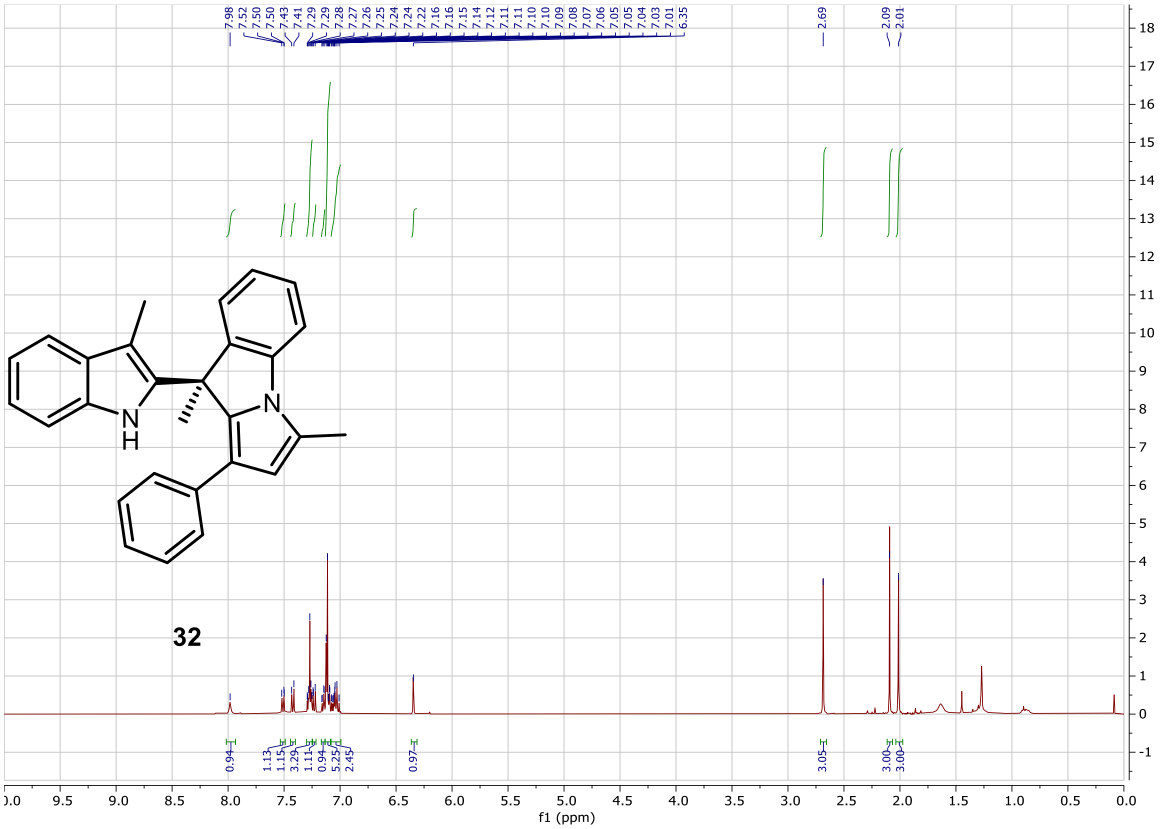

Figure S43 - ^{1}H NMR spectrum of **32** recorded in $CDCl_3$ solution.

36

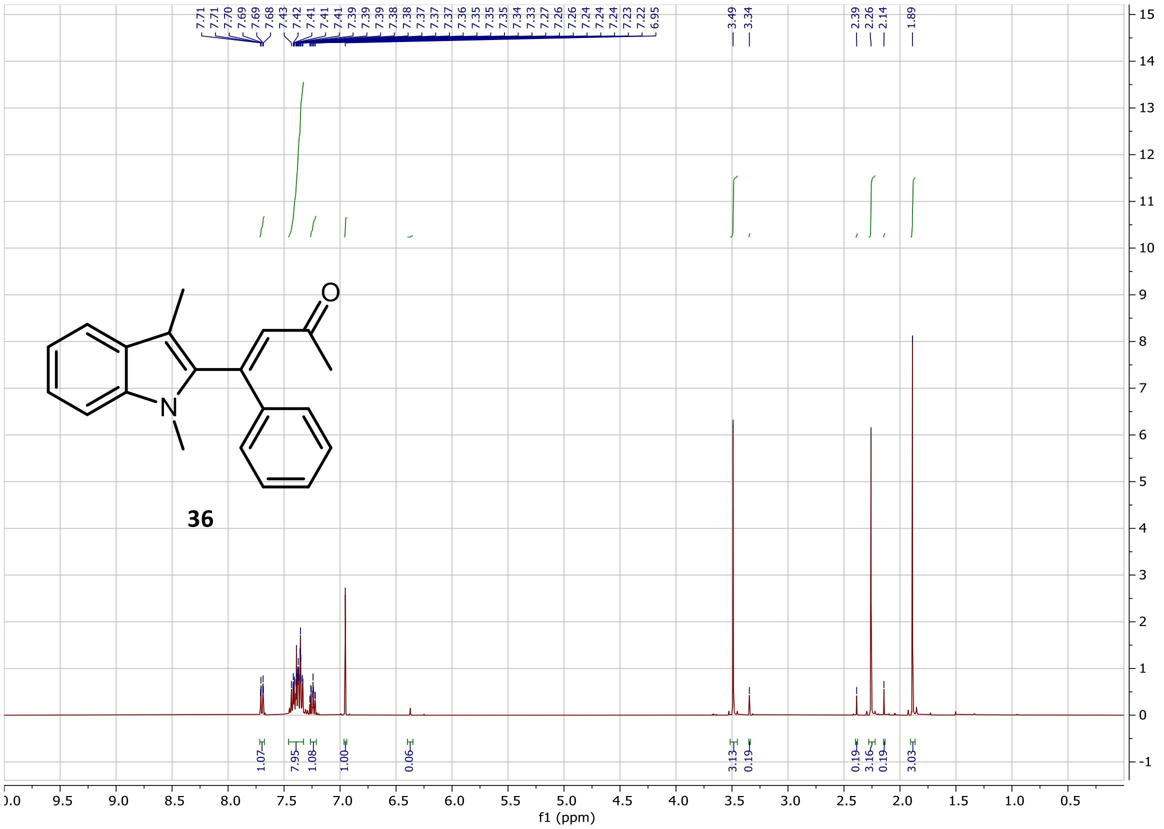

Figure S44 - ^{1}H NMR spectrum of **36** recorded in $CDCl_3$ solution.

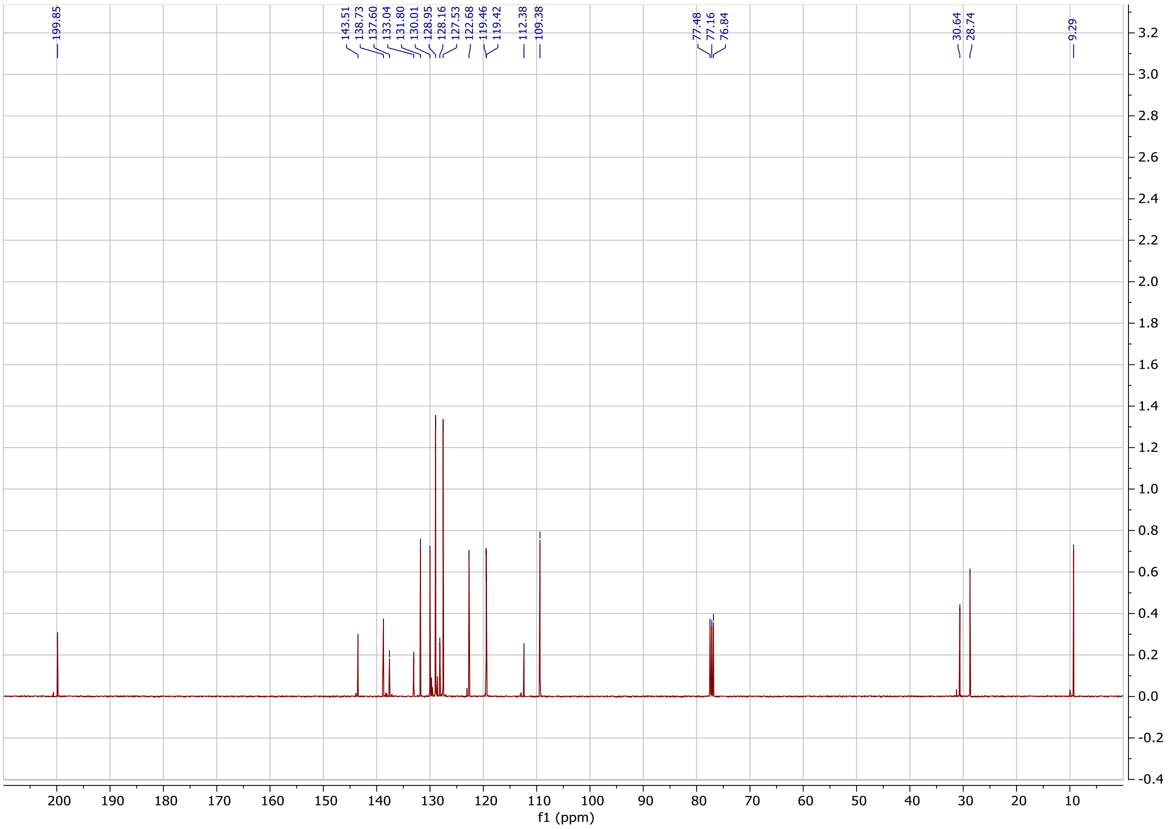

Figure S45 - $^{13}C\{^{1}H\}$ NMR spectrum of **36** recorded in $CDCl_3$ solution.

37

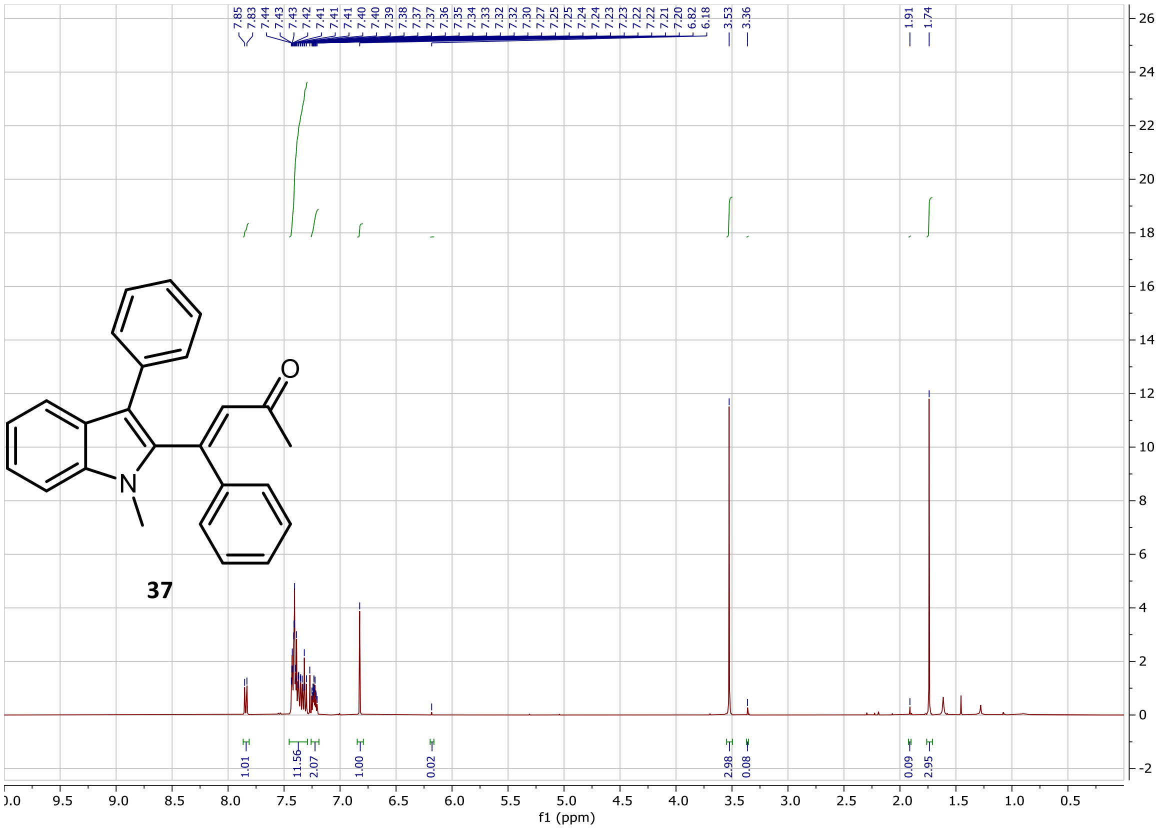

Figure S46 - ^{1}H NMR spectrum of **37** recorded in $CDCl_3$ solution.

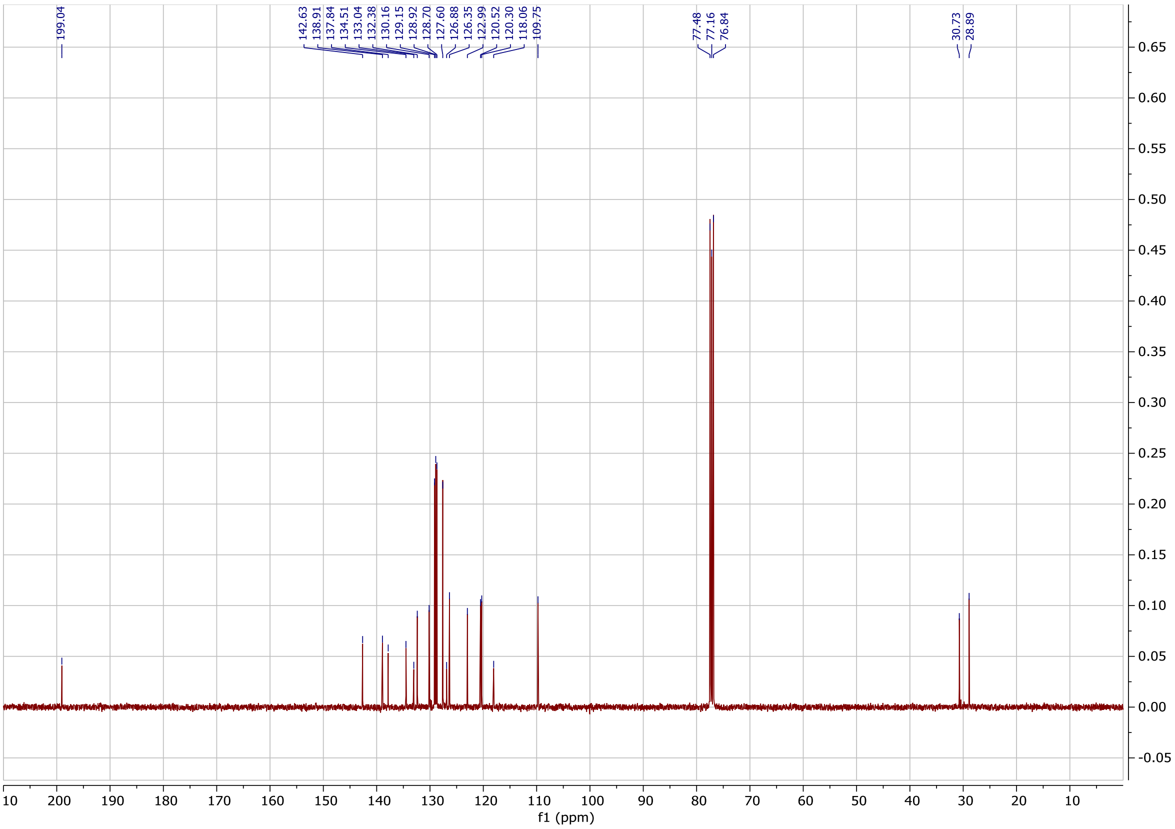

Figure S47 - $^{13}C\{^{1}H\}$ NMR spectrum of **37** recorded in $CDCl_3$ solution.

38

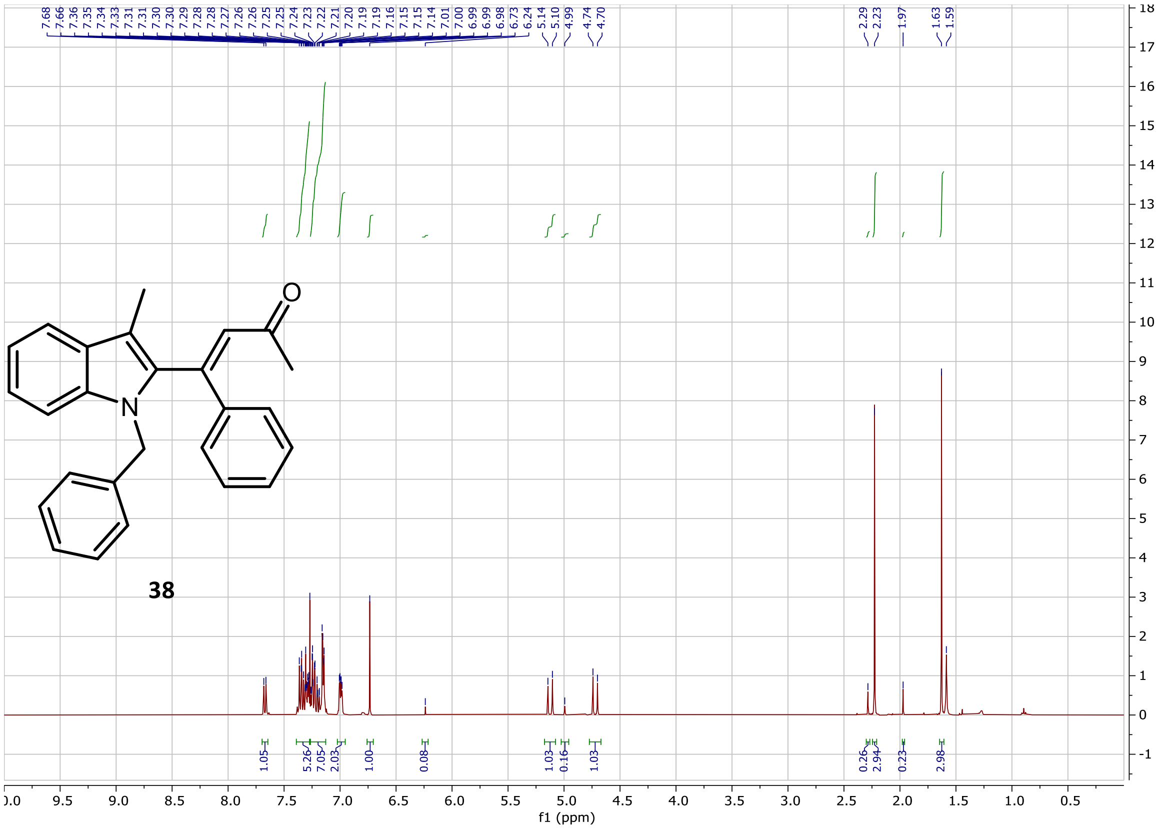

Figure S48 - ^{1}H NMR spectrum of **38** recorded in $CDCl_3$ solution.

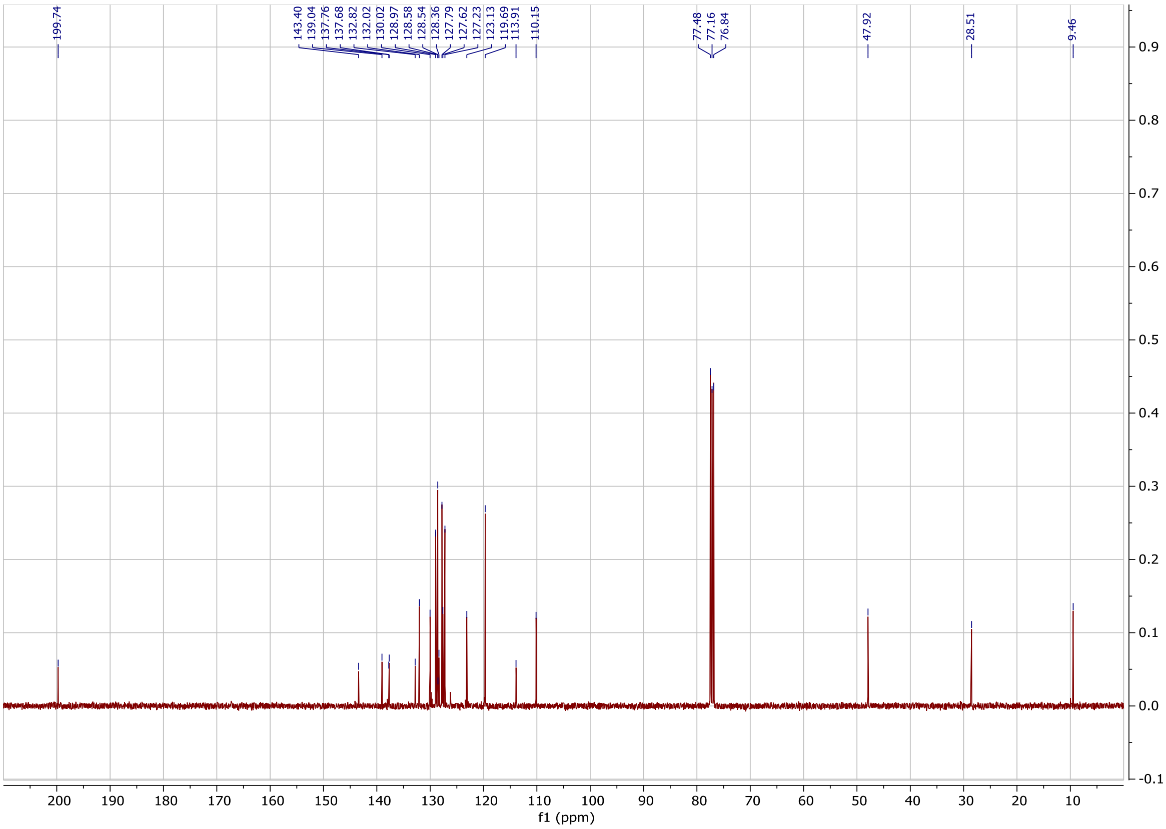

Figure S49 - $^{13}C\{^{1}H\}$ NMR spectrum of **38** recorded in $CDCl_3$ solution.

40

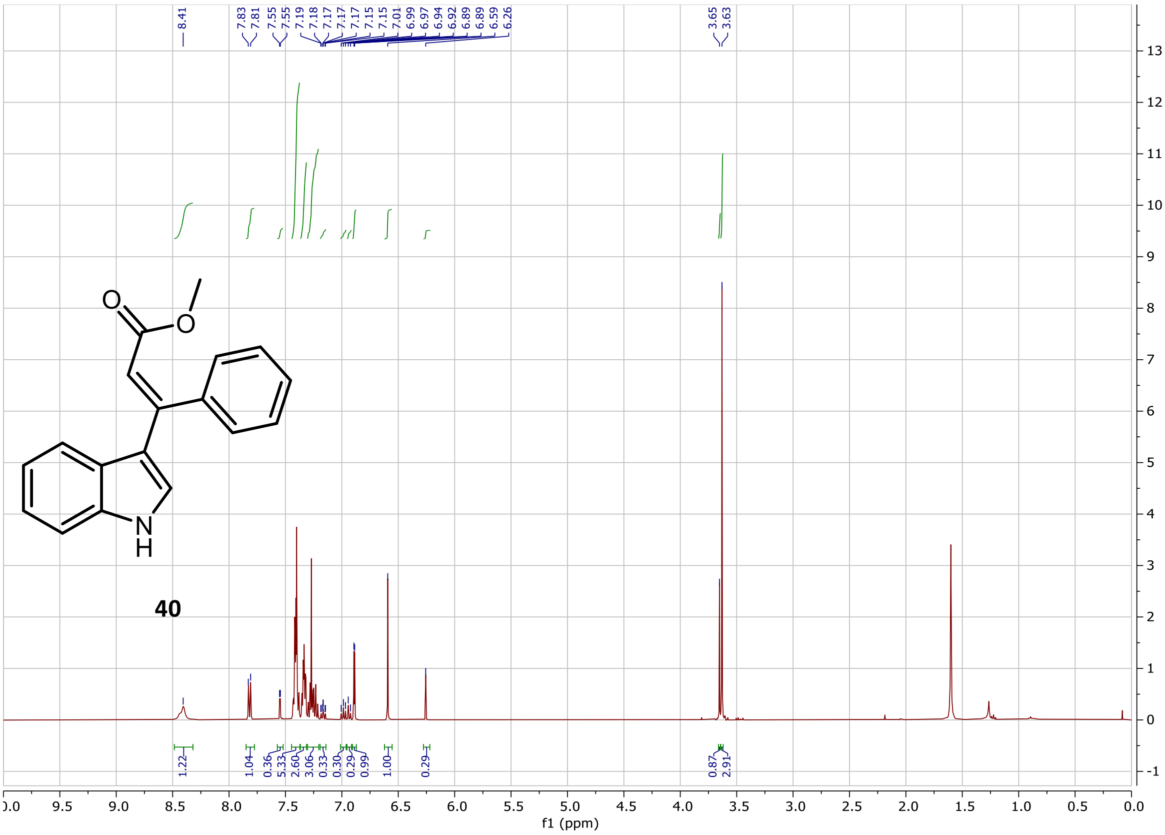

Figure S50 - ^{1}H NMR spectrum of **40** recorded in $CDCl_3$ solution.

41

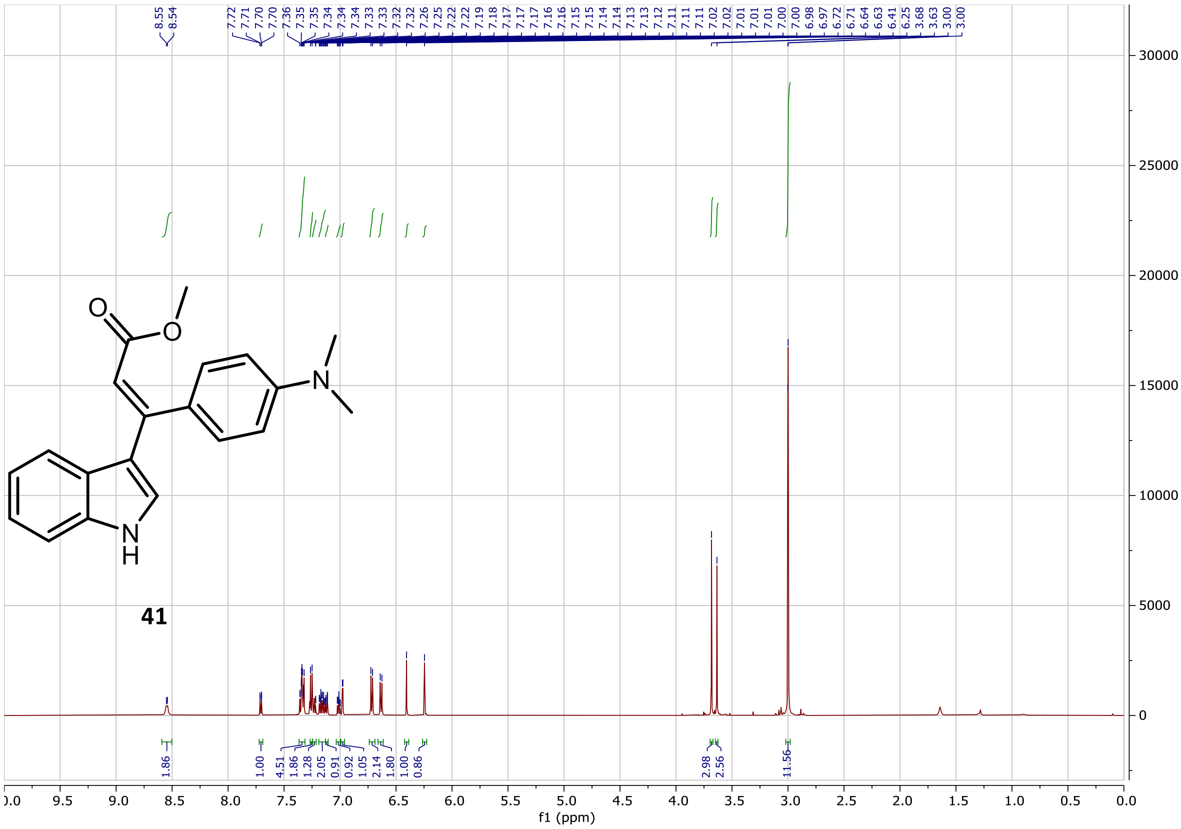

Figure S51 - ^{1}H NMR spectrum of **41** recorded in $CDCl_3$ solution.

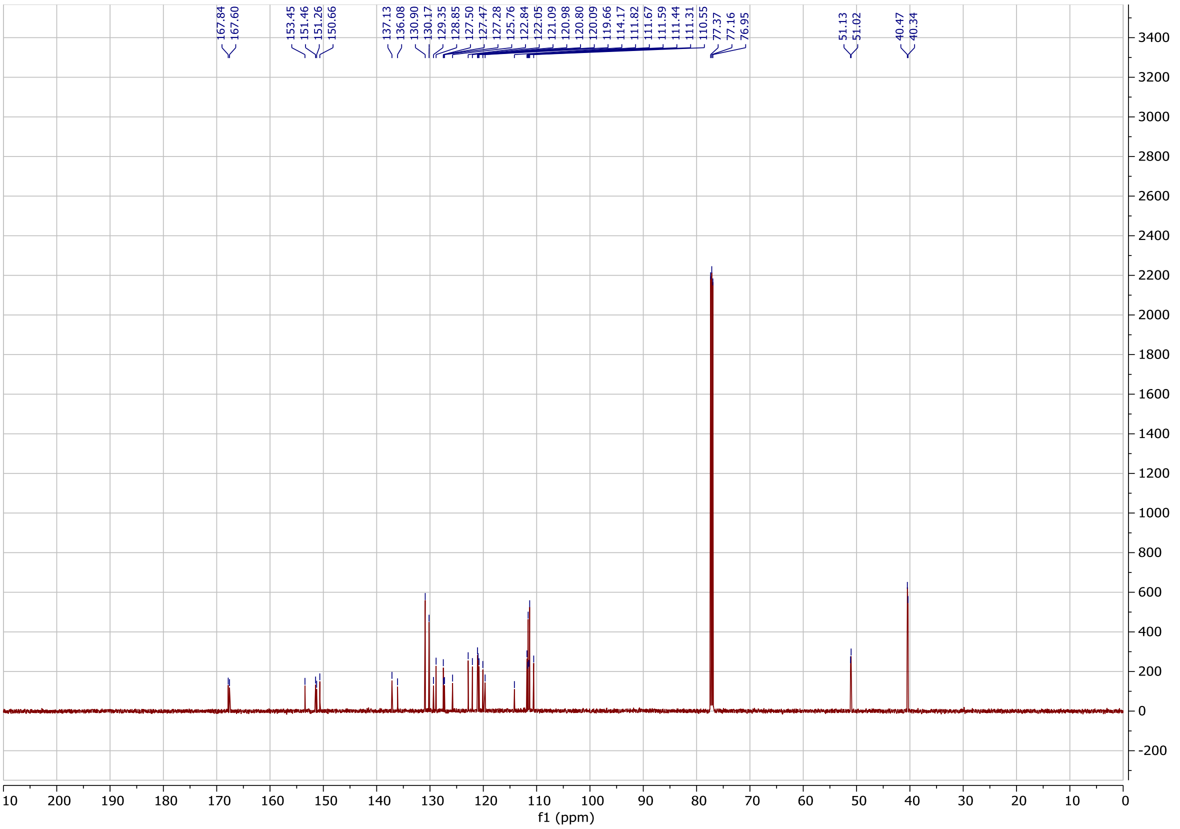

Figure S52 - $^{13}C\{^{1}H\}$ NMR spectrum of **41** recorded in $CDCl_3$ solution.

42

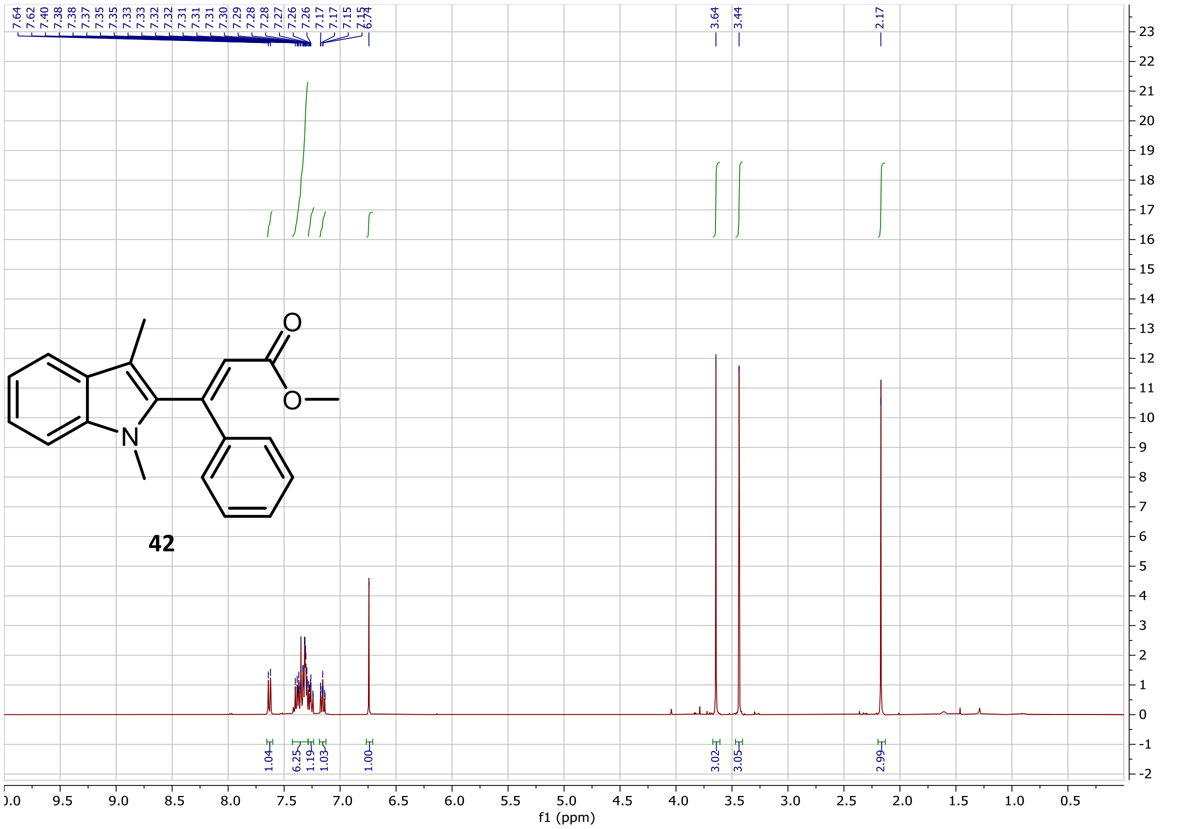

Figure S53 - ^{1}H NMR spectrum of **42** recorded in $CDCl_3$ solution.

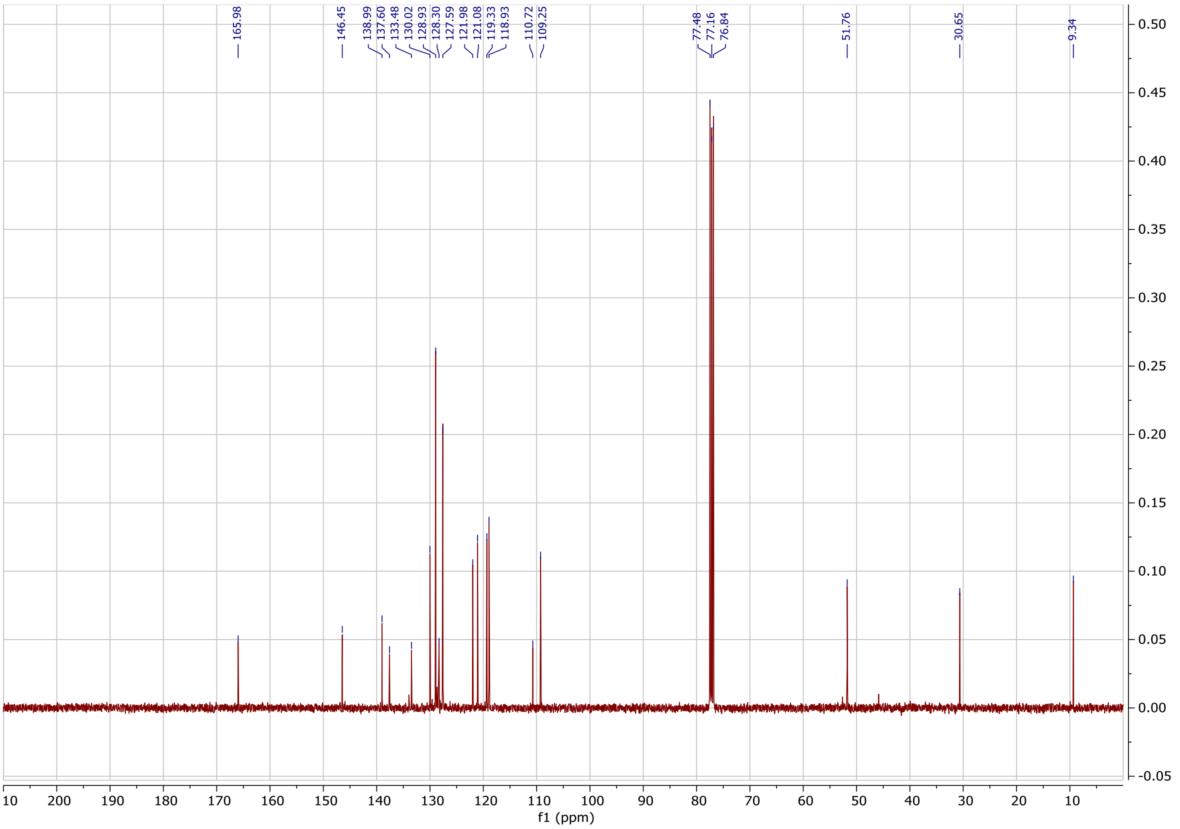

Figure S54 - $^{13}C\{^1H\}$ NMR spectrum of **42** recorded in $CDCl_3$ solution.

44

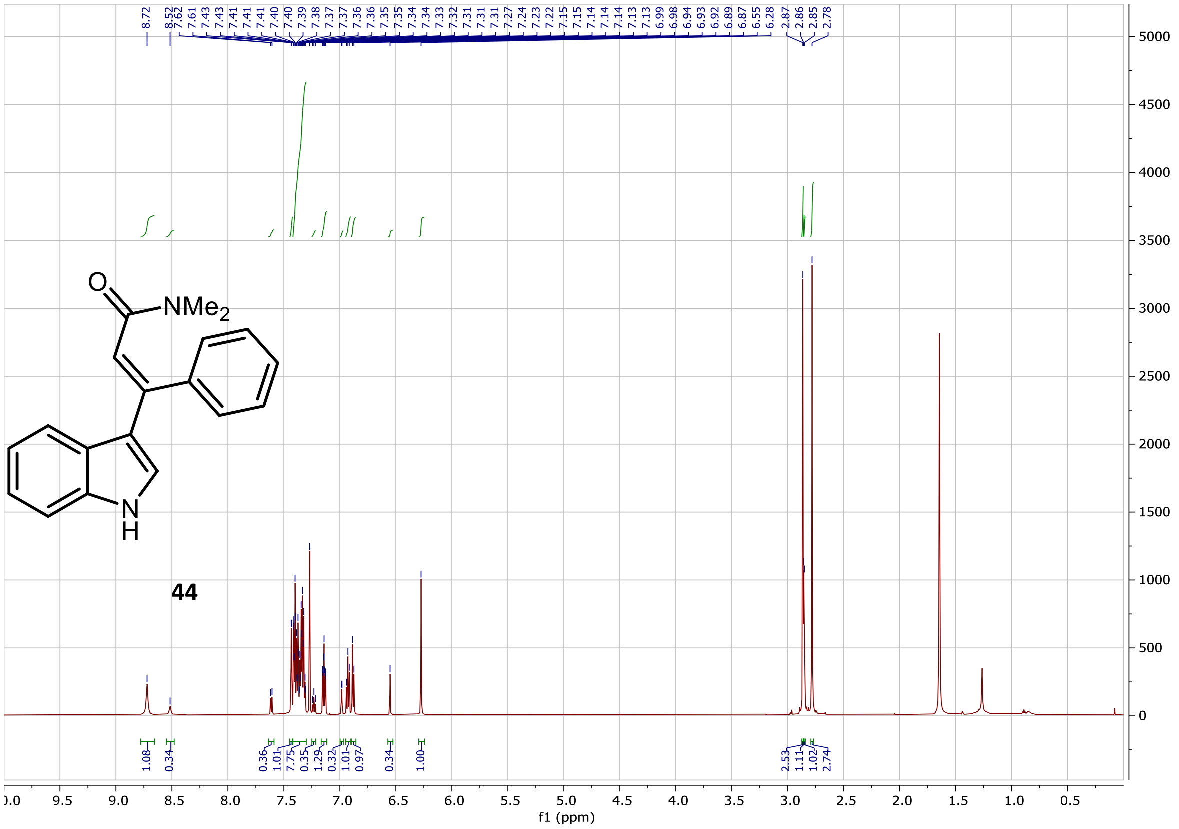

Figure S55 - ^{1}H NMR spectrum of **44** recorded in $CDCl_3$ solution.

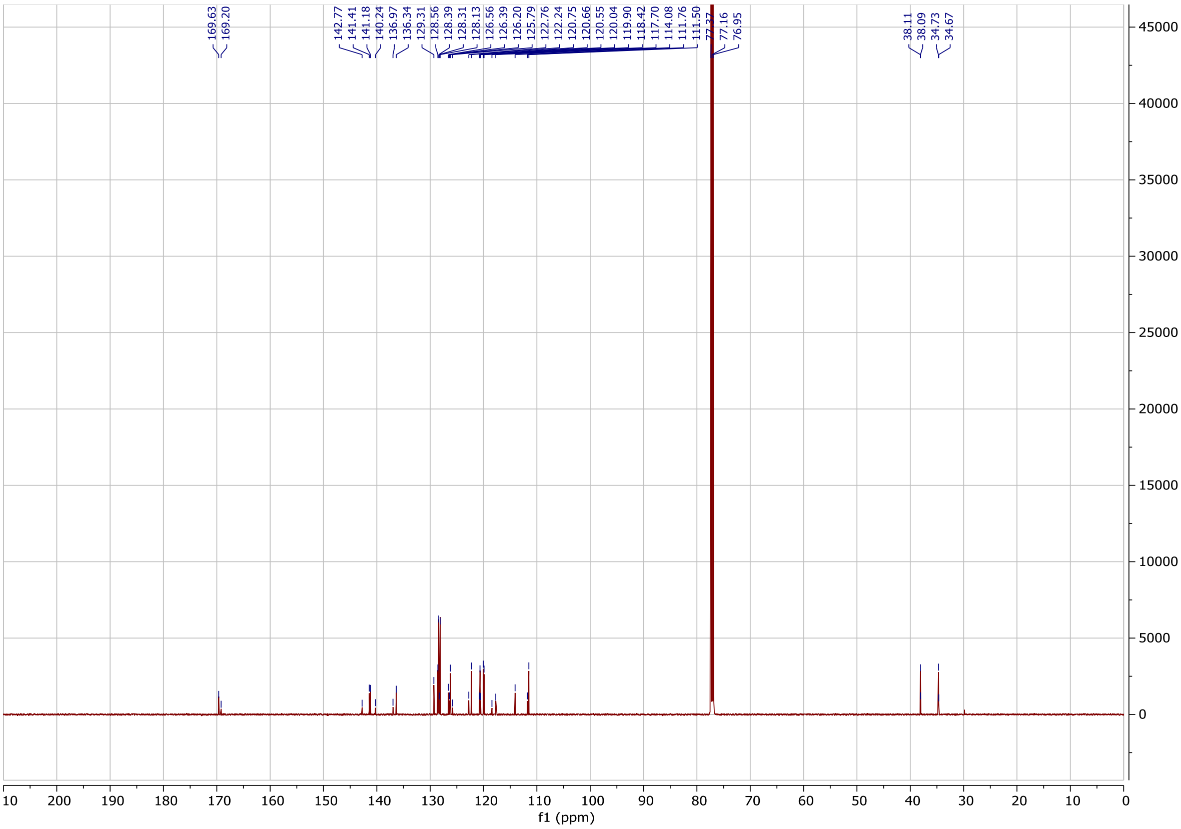

Figure S56 - $^{13}C\{^{1}H\}$ NMR spectrum of **44** recorded in $CDCl_3$ solution.

45

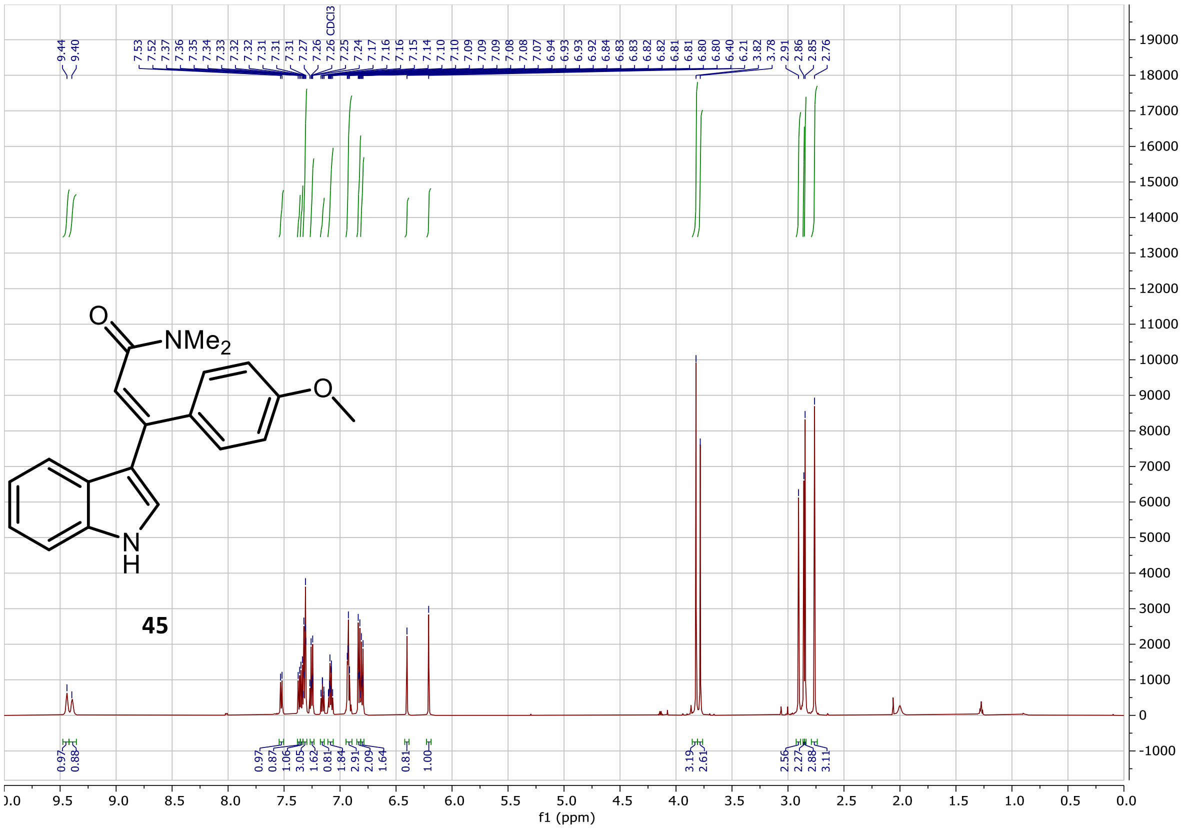

Figure S57 - ^{1}H NMR spectrum of **45** recorded in $CDCl_3$ solution.

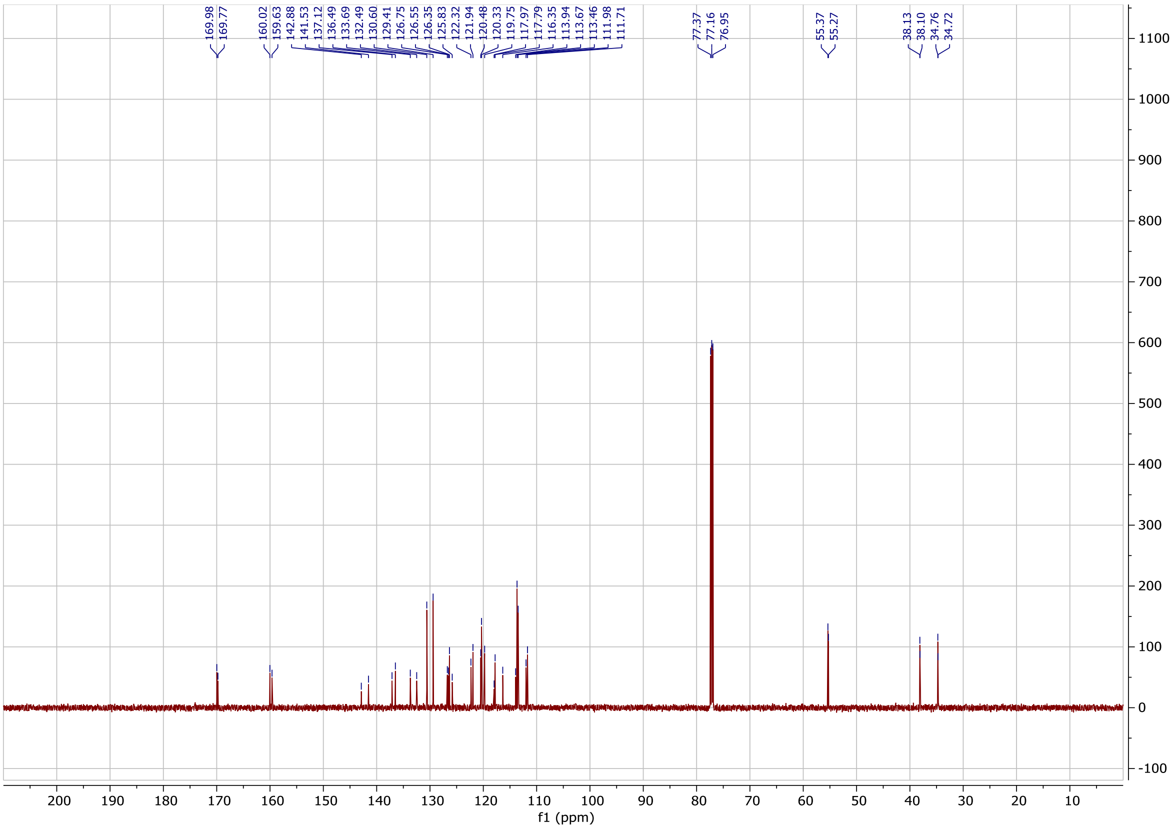

Figure S58 - $^{13}C\{^{1}H\}$ NMR spectrum of **45** recorded in $CDCl_3$ solution.

46

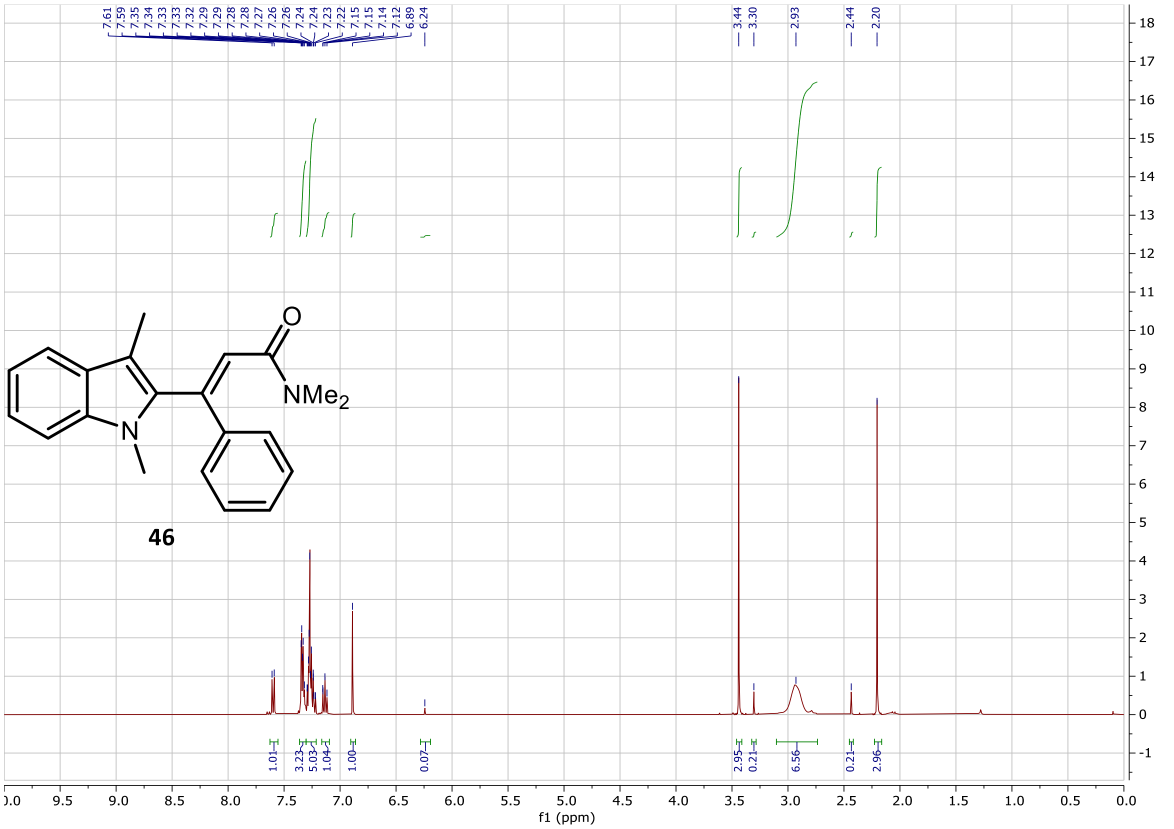

Figure S59 - ^{1}H NMR spectrum of **46** recorded in $CDCl_3$ solution.

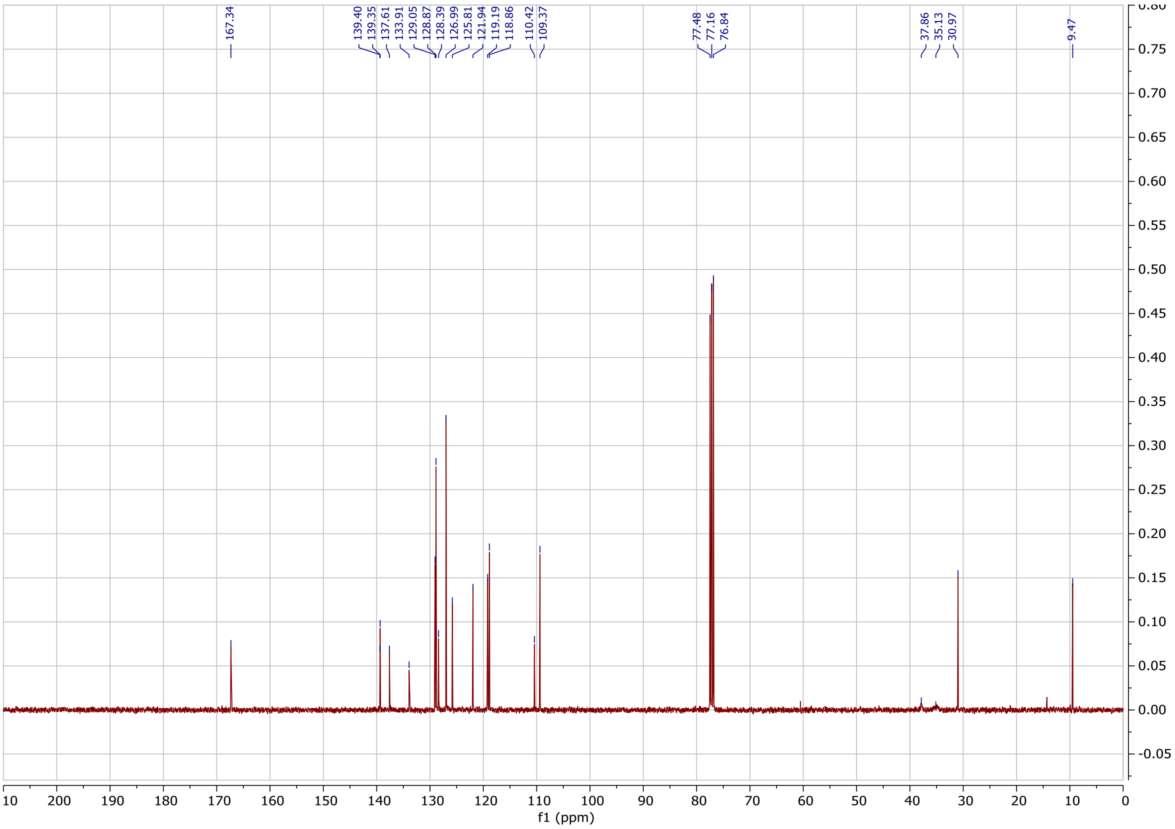

Figure S60 - $^{13}C\{^1H\}$ NMR spectrum of **46** recorded in $CDCl_3$ solution.

12

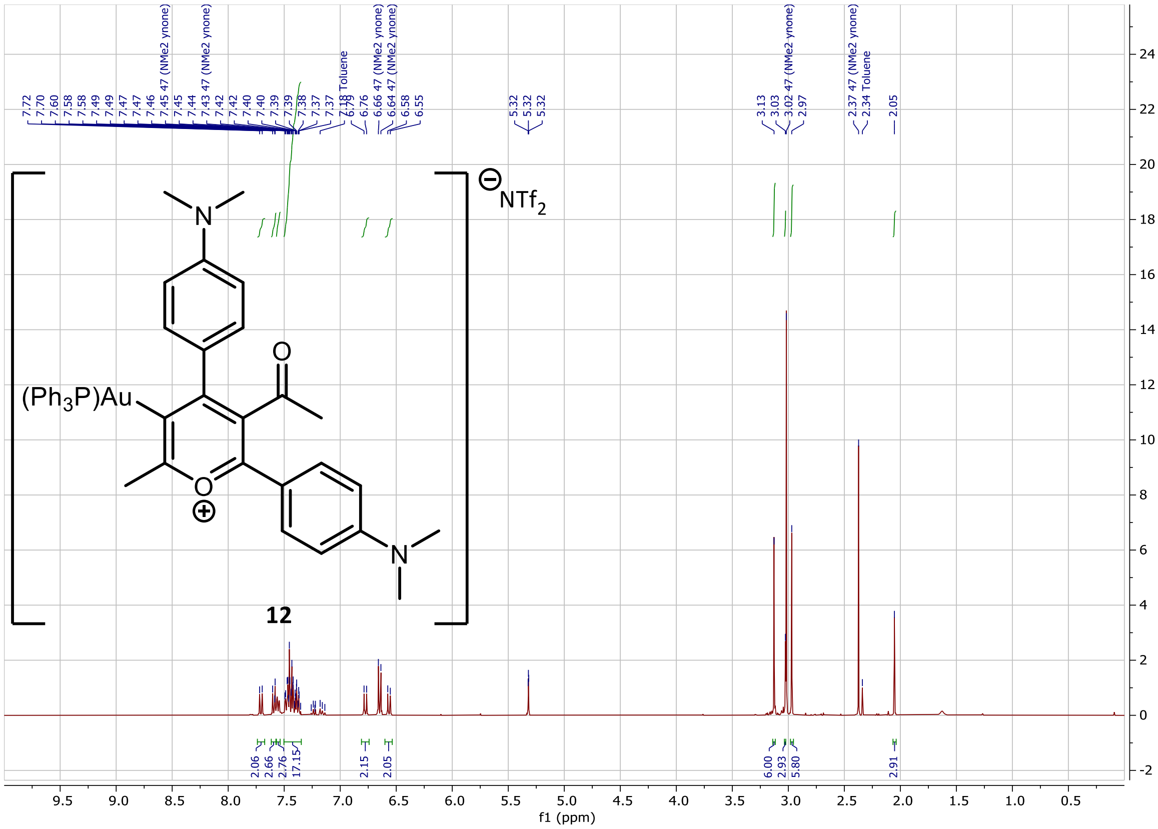

Figure S61 - ^{1}H NMR spectrum of **12** recorded in CD_2Cl_2 solution.

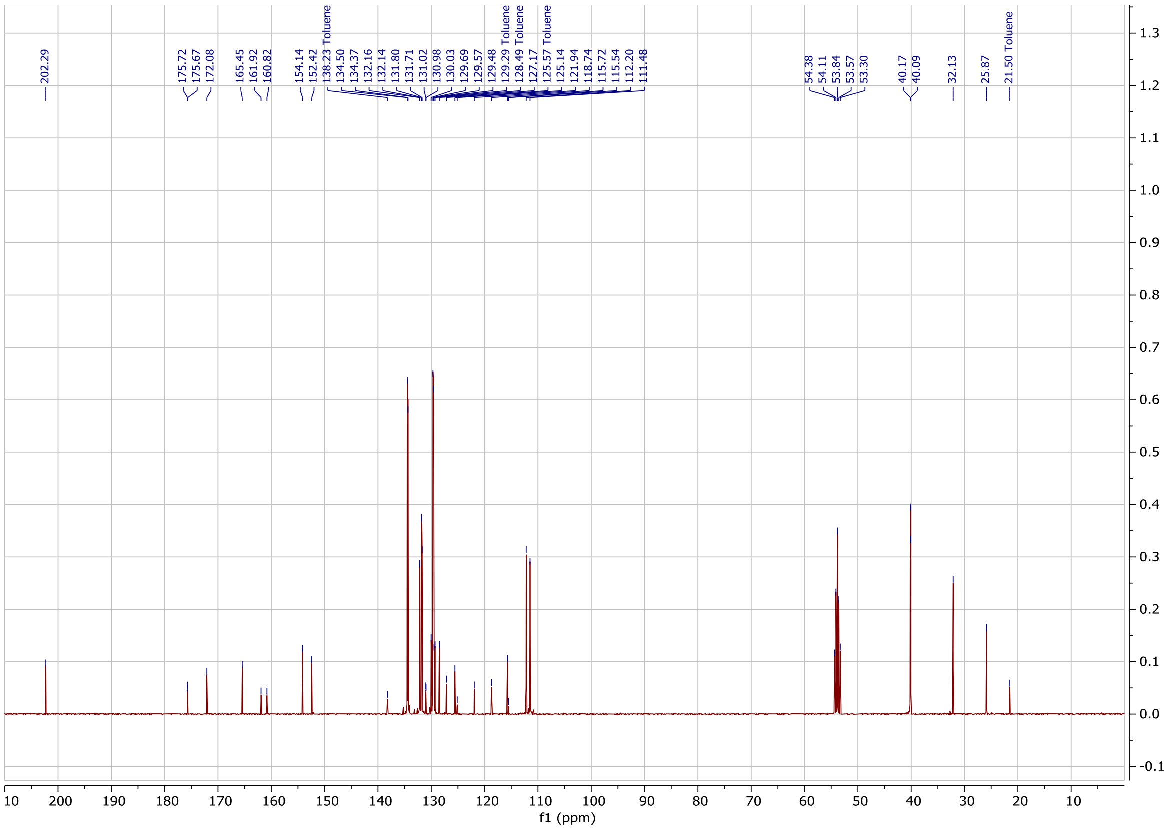

Figure S62 - $^{13}C\{^{1}H\}$ NMR spectrum of **12** recorded in CD_2Cl_2 solution.

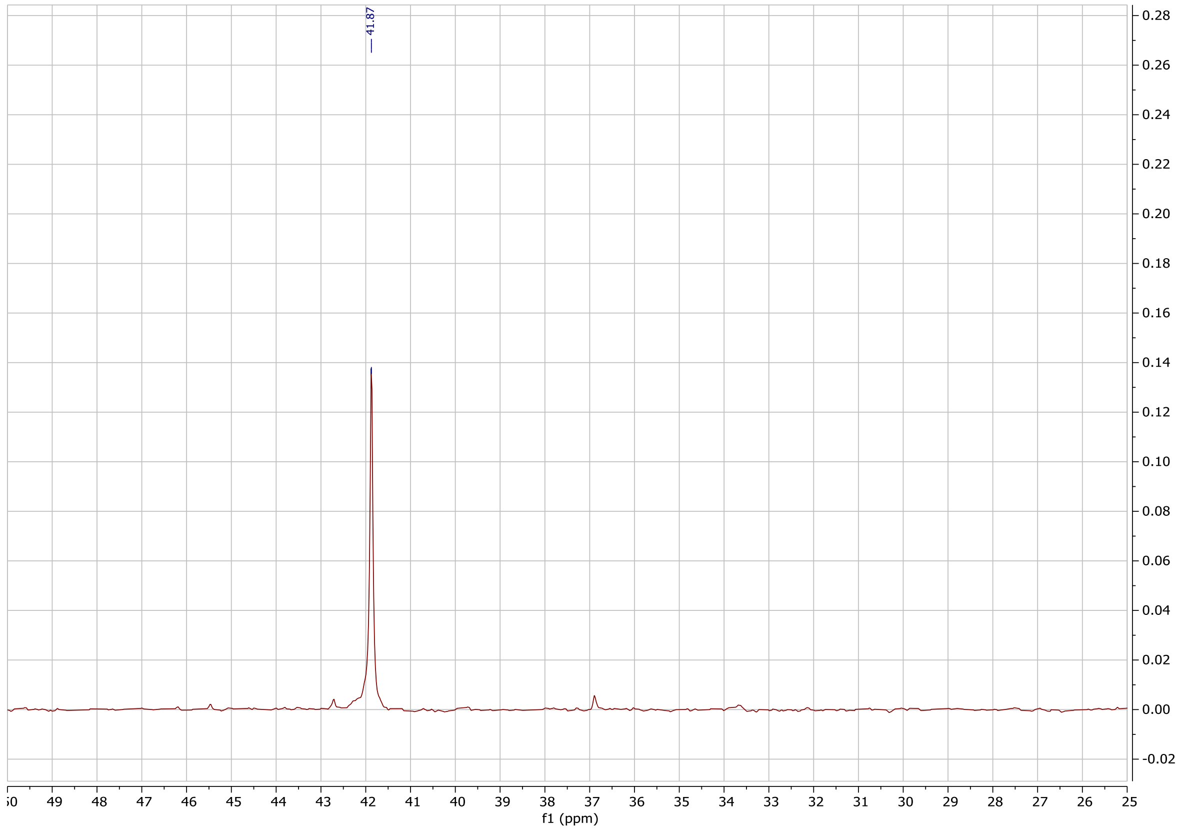

Figure S63 - $^{31}P\{^1H\}$ NMR spectrum of **12** recorded in CD_2Cl_2 solution.

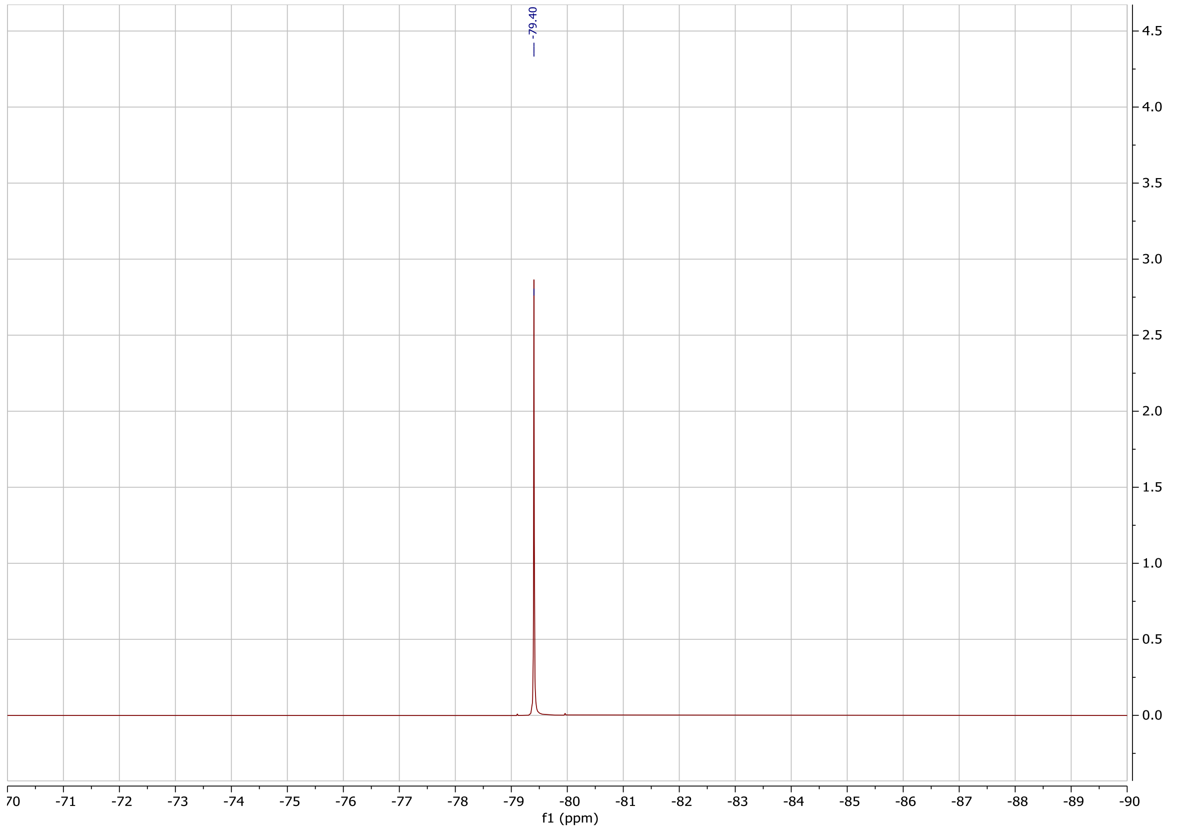

Figure S64 - ^{19}F NMR spectrum of **12** recorded in CD_2Cl_2 solution.

DFT Calculations

Computational Method

All calculations were performed using the TURBOMOLE V6.4 package using the resolution of identity (RI) approximation.[13-24]

Initial optimisations were performed at the (RI-)BP86/SV(P) level, followed by frequency calculations at the same level. Transition states were located by initially performing a constrained minimisation (by freezing internal coordinates that change most during the reaction) of a structure close to the anticipated transition state. This was followed by a frequency calculation to identify the transition vector to follow during a subsequent transition state optimisation. A final frequency calculation was then performed on the optimised transition-state structure. All minima were confirmed as such by the absence of imaginary frequencies and all transition states were identified by the presence of only one imaginary frequency. Dynamic Reaction Coordinate (DRC) analysis confirmed that transition states were connected to the appropriate minima. Single-point calculations on the (RI-)BP86/SV(P) optimised geometries were performed using the hybrid PBE0 functional and the flexible def2-TZVPP basis set. The (RI-)PBE0/def2-TZVPP SCF energies were corrected for their zero-point energies, thermal energies and entropies (obtained from the (RI-)BP86/SV(P)-level frequency calculations). A 60 electron quasi-relativistic ECP replaced the core electrons of Au. No symmetry constraints were applied during optimisations. Solvent corrections were applied with the COSMO dielectric continuum model,[25] and dispersion effects modelled with Grimme's D3 method.[26,27]

Energies and the imaginary frequency of transition states are presented. xyz coordinates of each structure are presented in a separate file available to download.

Energies of C2 and C3 Indole Addition at Different Levels of Theory

Due to the similar energy of **TS_{BD-H}** and **TS_{BF-H}** at the D3-PBE0/def2-TZVPP//BP86/SV(P) level of theory (see main text, Figure 5), different levels of theory were employed to compare the energies of these structures. At each level of theory (Table S3), the energy of C-3 attack (**TS_{BD-H}**) was indeed lower in energy than C-2 attack (**TS_{BF-H}**).

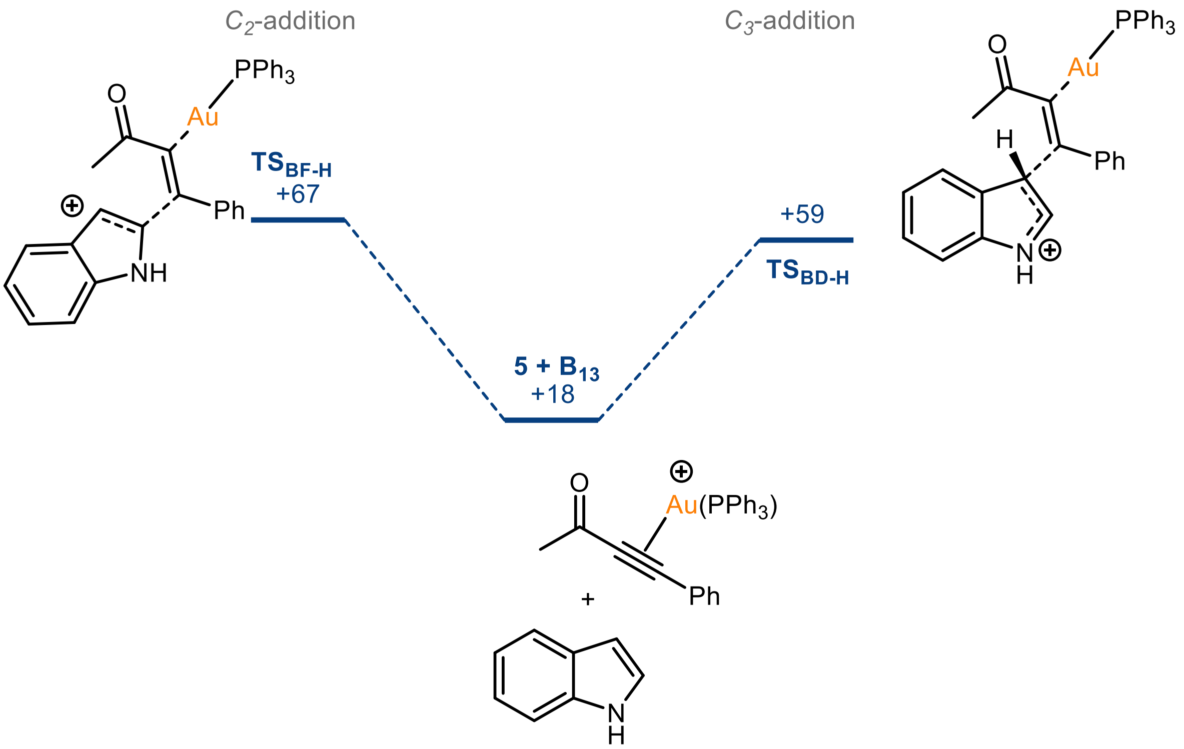

Figure S65 - C2 and C3 transition states for the addition of indole **5** into gold-alkyne coordinated ynone **B_{13}**.

Table S3 - DFT-calculated energies of TS_{BD-H} and TS_{BF-H} at different levels of theory. Energies are Gibbs energies at 298.15 K. Solvation refers to a COSMO solvent correction in toluene.

Level of Theory	Solvation	TS_{BD-H}/ kJ mol^{-1}	TS_{BF-H} / kJ mol^{-1}
BP86/SV(P)	N	+71	+76
BP86/SV(P)	Y	+84	+91
PBE0/def2-TZVPP//BP86/SV(P)	N	+85	+92
D3-PBE0/def2-TZVPP//BP86/SV(P)	N	+44	+49
PBE0/def2-TZVPP//BP86/SV(P)	Y	+100	+109
D3-PBE0/def2-TZVPP//BP86/SV(P)	Y	+59	+67

Interaction of the Triflimide Anion with Cationic Gold Complexes.

The thermodynamics of forming the gold-coordinated ynone complex with the triflimide counterion included is shown below (Scheme S1), for both the unsubstituted ynone **13** and electron-rich ynone **47**. The coordination mode of the gold catalyst and position of the triflimide anion were varied, with the scheme below showing the lowest energy isomer. 3D images produced by CYLview[28] are included with the collated energies to accurately show the position of the triflimide anion.

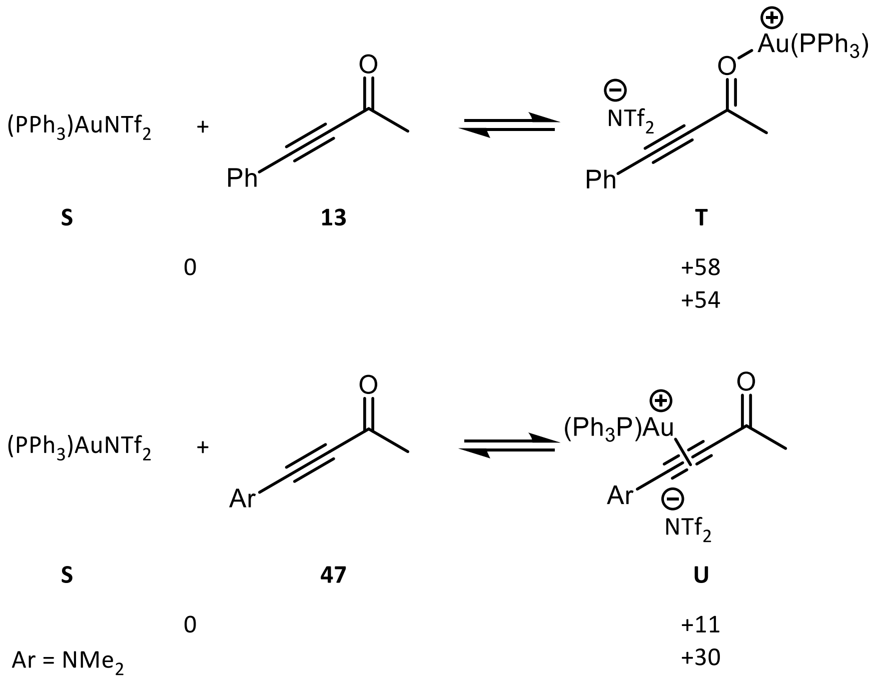

Scheme S1 - DFT-calculated energies for the formation of the gold-coordinated ynone complexes. All energies are Gibbs energies at 298.15 K at the D3(BJ)-PBE0/def2-TZVPP//BP86/SV(P) level of theory with COSMO solvent correction in CH_2Cl_2 (top value) and toluene (bottom value).

The relative change in free energy upon coordination of the gold catalyst to indole **5** and ynone **13** (see main paper, Scheme 3) was reconsidered with the triflimide anion included in the structures (Scheme S2), with solvent corrections applied in CH_2Cl_2 (top value) and toluene (bottom value). Gold-coordinated indole complex with the triflimide anion coordinated to the indole NH (**V**) was calculated as the lowest energy isomer and was taken as the reference state for the rest of the energies. Gold-coordinated ynone species with the triflimide anion included (**T**, **Y** and **Z**) were highly endergonic (+37 and +39 kJ mol^{-1} in CH_2Cl_2), independent of the specific coordination mode. Higher energies were calculated for structures in which the gold cation and triflimide anion were separated – particularly so when the solvent correction was applied with toluene, likely caused by the polarity differences between CH_2Cl_2 and toluene.

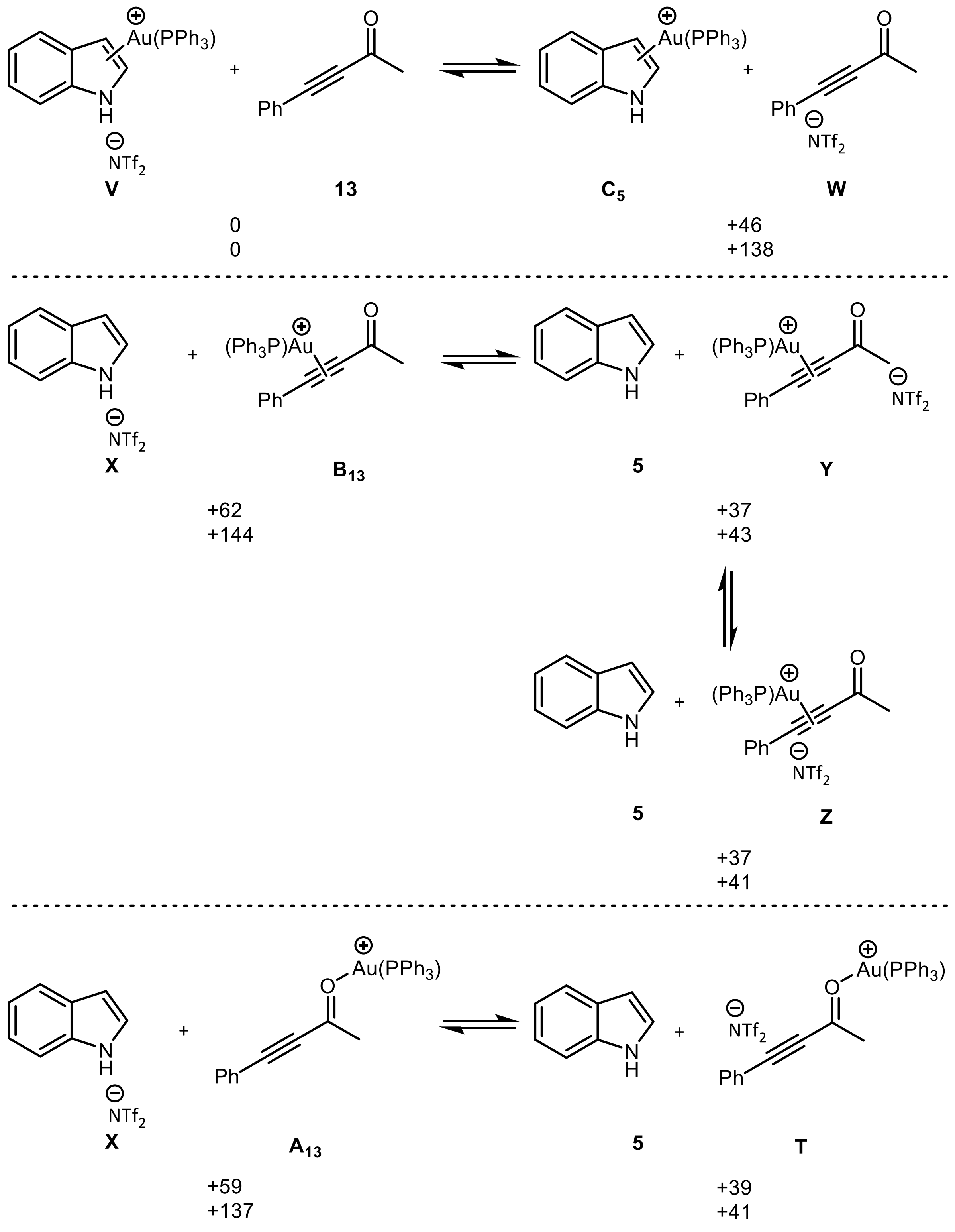

Scheme S2 - DFT-calculated energies comparing species with varied $[Au(PPh_3)]^+$ and triflimide anion coordination. All energies are Gibbs energies at 298.15 K at the D3(BJ)-PBE0/def2-TZVPP//BP86/SV(P) level of theory with COSMO solvent correction in CH_2Cl_2 (top value) and toluene (bottom value).

These data support the fundamental finding from the work that indole coordination to the gold is more thermodynamically favourable than with η^1(O) or $\eta 2(\pi)$ binding to the alkyne and plays a role in inhibiting the bimolecular reaction.

Energies

Indoles

5

SCF Energy (au) (RI)BP86/SV(P) = -363.5654002
SCF Energy (au) (RI)BP86/SV(P) = -363.5704067674 (toluene correction)
SCF Energy (au) (RI)PBE0/def2-TZVPP = -363.5194187
SCF Energy (au) (RI)PBE0/def2-TZVPP = -363.5299837672 (DCM correction)
SCF Energy (au) (RI)PBE0/def2-TZVPP = -363.5249793543 (toluene correction)
Zero Point Energy (au) = 0.1262955
Chemical potential (kJ mol^{-1}) = 251.72
Dispersion correction (au) (RI)PBE0/def2-TZVPP = -0.03618137

31

SCF Energy (au) (RI)BP86/SV(P) = -402.8507792043
SCF Energy (au) (RI)PBE0/def2-TZVPP = -402.8507792043
SCF Energy (au) (RI)PBE0/def2-TZVPP = -402.8071151343 (toluene correction)
Zero Point Energy (au) = 0.1530157
Chemical potential (kJ mol^{-1}) = 316.05
Dispersion correction (au) (RI)PBE0/def2-TZVPP = -0.01801967

Alkynes

13

SCF Energy (au) (RI)BP86/SV(P) = -460.7142835347
SCF Energy (au) (RI)BP86/SV(P) = -460.718894525 (toluene correction)
SCF Energy (au) (RI)PBE0/def2-TZVPP = -460.6595881932
SCF Energy (au) (RI)PBE0/def2-TZVPP = -460.6706447768 (DCM correction)
SCF Energy (au) (RI)PBE0/def2-TZVPP = -460.6653732945 (toluene correction)
Zero Point Energy (au) = 0.1431076
Chemical potential (kJ mol^{-1}) = 276.36
Dispersion correction (au) (RI)PBE0/def2-TZVPP = -0.01650162

47

SCF Energy (au) (RI)BP86/SV(P) = -594.5871358119
SCF Energy (au) (RI)PBE0/def2-TZVPP = -594.5211849986
SCF Energy (au) (RI)PBE0/def2-TZVPP = -594.5361399802 (DCM correction)
SCF Energy (au) (RI)PBE0/def2-TZVPP = -594.5289095977 (toluene correction)
Zero Point Energy (au) = 0.2139250
Chemical potential (kJ mol^{-1}) = 447.07
Dispersion correction (au) (RI)PBE0/def2-TZVPP = -0.02470142

52

SCF Energy (au) (RI)BP86/SV(P) = -575.1596578421
SCF Energy (au) (RI)PBE0/def2-TZVPP = -575.1059996162
SCF Energy (au) (RI)PBE0/def2-TZVPP = -575.1195292587 (DCM correction)
Zero Point Energy (au) = 0.1747071
Chemical potential (kJ mol^{-1}) = 350.73
Dispersion correction (au) (RI)PBE0/def2-TZVPP = -0.02009000

53

SCF Energy (au) (RI)BP86/SV(P) = -3034.2508579450
SCF Energy (au) (RI)PBE0/def2-TZVPP = -3033.920207880
SCF Energy (au) (RI)PBE0/def2-TZVPP = -3033.9312607153 (DCM correction)
Zero Point Energy (au) = 0.1330876
Chemical potential (kJ mol^{-1}) = 241.27
Dispersion correction (au) (RI)PBE0/def2-TZVPP = -0.01884500

54

SCF Energy (au) (RI)BP86/SV(P) = -665.1013700856
SCF Energy (au) (RI)PBE0/def2-TZVPP = -665.0409699881
SCF Energy (au) (RI)PBE0/def2-TZVPP = -665.0557023287 (DCM correction)
Zero Point Energy (au) = 0.1453704
Chemical potential (kJ mol^{-1}) = 270.22
Dispersion correction (au) (RI)PBE0/def2-TZVPP = -0.01963230

Vinylated Indole Products

14a

SCF Energy (au) (RI)BP86/SV(P) = -824.3377725069

SCF Energy (au) (RI)PBE0/def2-TZVPP = -824.2387991595

SCF Energy (au) (RI)PBE0/def2-TZVPP = -824.2490760965 (toluene correction)

Zero Point Energy (au) = 0.2734539

Chemical potential (kJ mol^{-1}) = 597.30

Dispersion correction (au) (RI)PBE0/def2-TZVPP = -0.04158047

55

SCF Energy (au) (RI)BP86/SV(P) = -824.3431808595

SCF Energy (au) (RI)PBE0/def2-TZVPP = -824.242096096

SCF Energy (au) (RI)PBE0/def2-TZVPP = -824.2498565033 (toluene correction)

Zero Point Energy (au) = 0.2735973

Chemical potential (kJ mol^{-1}) = 597.09

Dispersion correction (au) (RI)PBE0/def2-TZVPP = -0.04026478

56

SCF Energy (au) (RI)BP86/SV(P) = -863.6237725056

SCF Energy (au) PBE0/def2-TZVPP = -863.5234480024

SCF Energy (au) PBE0/def2-TZVPP = -863.5323684151 (toluene correction)

Zero Point Energy (au) = 0.3002982

Chemical potential (kJ mol^{-1}) = 662.81

Dispersion correction (au) PBE0/def2-TZVPP = -0.04610171

57

SCF Energy (au) (RI)BP86/SV(P) = -863.5821170571

SCF Energy (au) PBE0/def2-TZVPP = -863.4805647943

SCF Energy (au) PBE0/def2-TZVPP = -863.4882884565 (toluene correction)

Zero Point Energy (au) = 0.2994459

Chemical potential (kJ mol^{-1}) = 662.24

Dispersion correction (au) PBE0/def2-TZVPP = -0.0476954

***O*-coordinated Gold Complexes**

A_{13}

SCF Energy (au) (RI)BP86/SV(P) = -1632.2089323840
SCF Energy (au) (RI)PBE0/def2-TZVPP = -1631.858945451
SCF Energy (au) (RI)PBE0/def2-TZVPP = -1631.9067811976 (DCM correction)
SCF Energy (au) (RI)PBE0/def2-TZVPP = -1631.8858638047 (toluene correction)
Zero Point Energy (au) = 0.4127436
Chemical potential (kJ mol^{-1}) = 897.04
Dispersion correction (au) (RI)PBE0/def2-TZVPP = -0.07326290

A_{47}

SCF Energy (au) (RI)BP86/SV(P) = -1766.0937496670
SCF Energy (au) (RI)PBE0/def2-TZVPP = -1765.731173119
SCF Energy (au) (RI)PBE0/def2-TZVPP = -1765.7781302147 (DCM correction)
Zero Point Energy (au) = 0.4839063
Chemical potential (kJ mol^{-1}) = 1069.18
Dispersion correction (au) (RI)PBE0/def2-TZVPP = -0.08146736

A_{48}

SCF Energy (au) (RI)BP86/SV(P) = -1726.8259482840
SCF Energy (au) (RI)PBE0/def2-TZVPP = -1726.469844621
SCF Energy (au) (RI)PBE0/def2-TZVPP = -1726.5171161237 (DCM correction)
Zero Point Energy (au) = 0.4575545
Chemical potential (kJ mol^{-1}) = 1004.42
Dispersion correction (au) (RI)PBE0/def2-TZVPP = -0.07981239

A_{49}

SCF Energy (au) (RI)BP86/SV(P) = -1860.7060209980
SCF Energy (au) (RI)PBE0/def2-TZVPP = -1860.337959735
SCF Energy (au) (RI)PBE0/def2-TZVPP = -1860.3843645118 (DCM correction)
Zero Point Energy (au) = 0.5284427
Chemical potential (kJ mol^{-1}) = 1176.94
Dispersion correction (au) (RI)PBE0/def2-TZVPP = -0.08789920

A_{50}

SCF Energy (au) (RI)BP86/SV(P) = -1707.3867364320
SCF Energy (au) (RI)PBE0/def2-TZVPP = -1707.041270314
SCF Energy (au) (RI)PBE0/def2-TZVPP = -1707.0889912694 (DCM correction)
Zero Point Energy (au) = 0.4184572
Chemical potential (kJ mol^{-1}) = 908.53
Dispersion correction (au) (RI)PBE0/def2-TZVPP = -0.07403348

A_{51}

SCF Energy (au) (RI)BP86/SV(P) = -1841.2690603310
SCF Energy (au) (RI)PBE0/def2-TZVPP = -1840.911485096
SCF Energy (au) (RI)PBE0/def2-TZVPP = -1840.9584674554 (DCM correction)
Zero Point Energy (au) = 0.4894453
Chemical potential (kJ mol^{-1}) = 1080.06
Dispersion correction (au) (RI)PBE0/def2-TZVPP = -0.08217718

Alkyne-coordinated Gold Complexes

B_{13}

SCF Energy (au) (RI)BP86/SV(P) = -1632.1982463430
SCF Energy (au) (RI)BP86/SV(P) = -1632.2253162549 (toluene correction)
SCF Energy (au) (RI)PBE0/def2-TZVPP = -1631.851307023
SCF Energy (au) (RI)PBE0/def2-TZVPP = -1631.9022640297 (DCM correction)
SCF Energy (au) (RI)PBE0/def2-TZVPP = -1631.8798233785 (toluene correction)
Zero Point Energy (au) = 0.4116606
Chemical potential (kJ mol^{-1}) = 893.21
Dispersion correction (au) (RI)PBE0/def2-TZVPP = -0.07548566

B_{47}

SCF Energy (au) (RI)BP86/SV(P) = -1766.0879636720
SCF Energy (au) (RI)PBE0/def2-TZVPP = -1765.726769871
SCF Energy (au) (RI)PBE0/def2-TZVPP = -1765.7781570815 (DCM correction)
Zero Point Energy (au) = 0.4832314
Chemical potential (kJ mol^{-1}) = 1066.40
Dispersion correction (au) (RI)PBE0/def2-TZVPP = -0.08308189

B_{48}

SCF Energy (au) (RI)BP86/SV(P) = -1726.8102612640
SCF Energy (au) (RI)PBE0/def2-TZVPP = -1726.456901267
SCF Energy (au) (RI)PBE0/def2-TZVPP = -1726.5067724167 (DCM correction)
Zero Point Energy (au) = 0.4563079
Chemical potential (kJ mol^{-1}) = 998.63
Dispersion correction (au) (RI)PBE0/def2-TZVPP = -0.08158914

B_{49}

SCF Energy (au) (RI)BP86/SV(P) = -1860.6978515570
SCF Energy (au) (RI)PBE0/def2-TZVPP = -1860.329225798
SCF Energy (au) (RI)PBE0/def2-TZVPP = -1860.3823300319 (DCM correction)
Zero Point Energy (au) = 0.5279019
Chemical potential (kJ mol^{-1}) = 1174.60
Dispersion correction (au) (RI)PBE0/def2-TZVPP = -0.08883878

B_{50}

SCF Energy (au) (RI)BP86/SV(P) = -1707.3802325870
SCF Energy (au) (RI)PBE0/def2-TZVPP = -1707.035772876
SCF Energy (au) (RI)PBE0/def2-TZVPP = -1707.0877577646 (DCM correction)
Zero Point Energy (au) = 0.4174705
Chemical potential (kJ mol^{-1}) = 903.46
Dispersion correction (au) (RI)PBE0/def2-TZVPP = -0.07583652

B_{51}

SCF Energy (au) (RI)BP86/SV(P) = -1841.2699542220
SCF Energy (au) (RI)PBE0/def2-TZVPP = -1840.912534476
SCF Energy (au) (RI)PBE0/def2-TZVPP = -1840.9649053525 (DCM correction)
Zero Point Energy (au) = 0.4889985
Chemical potential (kJ mol^{-1}) = 1077.56
Dispersion correction (au) (RI)PBE0/def2-TZVPP = -0.08344635

B_{52}

SCF Energy (au) (RI)BP86/SV(P) = -1746.6511497560
SCF Energy (au) (RI)PBE0/def2-TZVPP = -1746.302411760
SCF Energy (au) (RI)PBE0/def2-TZVPP = -1746.3538908352 (DCM correction)
Zero Point Energy (au) = 0.4435577
Chemical potential (kJ mol^{-1}) = 968.16
Dispersion correction (au) (RI)PBE0/def2-TZVPP = -0.07859117

B_{53}

SCF Energy (au) (RI)BP86/SV(P) = -4205.7334295080
SCF Energy (au) (RI)PBE0/def2-TZVPP = -4205.108834950
SCF Energy (au) (RI)PBE0/def2-TZVPP = -4205.1606598237 (DCM correction)
Zero Point Energy (au) = 0.4016213
Chemical potential (kJ mol^{-1}) = 858.09
Dispersion correction (au) (RI)PBE0/def2-TZVPP = -0.07766887

B_{54}

SCF Energy (au) (RI)BP86/SV(P) = -1836.5759819500
SCF Energy (au) (RI)PBE0/def2-TZVPP = -1836.223673775
SCF Energy (au) (RI)PBE0/def2-TZVPP = -1836.2831946636 (DCM correction)
Zero Point Energy (au) = 0.4135558
Chemical potential (kJ mol^{-1}) = 886.01
Dispersion correction (au) (RI)PBE0/def2-TZVPP = -0.07875338

Indole-coordinated Gold Complexes

C_5

SCF Energy (au) (RI)BP86/SV(P) = -1535.055043902
SCF Energy (au) (RI)BP86/SV(P) = -1535.084691442 (toluene correction)
SCF Energy (au) (RI)PBE0/def2-TZVPP = -1534.717538227
SCF Energy (au) (RI)PBE0/def2-TZVPP = -1534.7730150426 (DCM correction)
SCF Energy (au) (RI)PBE0/def2-TZVPP = -1534.7484225556 (toluene correction)
Zero Point Energy (au) = 0.3957794
Chemical potential (kJ mol^{-1}) = 871.74
Dispersion correction (au) (RI)PBE0/def2-TZVPP = -0.07320066

C_{31}

SCF Energy (au) (RI)BP86/SV(P) = -1573.338042079
SCF Energy (au) (RI)PBE0/def2-TZVPP = -1573.998204752
SCF Energy (au) (RI)PBE0/def2-TZVPP = -1574.0282197694 (toluene correction)
Zero Point Energy (au) = 0.4225127
Chemical potential (kJ mol^{-1}) = 937.47
Dispersion correction (au) (RI)PBE0/def2-TZVPP = -0.07748812

Indole-vinylation Pathway

TS_{BD-H}

SCF Energy (au) (RI)BP86/SV(P) = -1995.764633294

SCF Energy (au) (RI)BP86/SV(P) = -1995.7938552419 (toluene correction)

SCF Energy (au) (RI)PBE0/def2-TZVPP = -1995.367311968

SCF Energy (au) (RI)PBE0/def2-TZVPP = -1995.3983017006 (toluene correction)

Zero Point Energy (au) = 0.5394534

Chemical potential (kJ mol^{-1}) = 1206.93

Dispersion correction (au) (RI)PBE0/def2-TZVPP = -0.10519127

Imaginary Frequency = -135.59

E_H

SCF Energy (au) (RI)BP86/SV(P) = -1995.813904821

SCF Energy (au) (RI)PBE0/def2-TZVPP = -1995.426654619

SCF Energy (au) (RI)PBE0/def2-TZVPP = -1995.4570096897 (toluene correction)

Zero Point Energy (au) = 0.5432136

Chemical potential (kJ mol^{-1}) = 1223.37

Dispersion correction (au) (RI)PBE0/def2-TZVPP = -0.10486142

TS_{BF-H}

SCF Energy (au) (RI)BP86/SV(P) = -1995.762065392

SCF Energy (au) (RI)BP86/SV(P) = -1995.7905337575 (toluene correction)

SCF Energy (au) (RI)PBE0/def2-TZVPP = -1995.363849682

SCF Energy (au) (RI)PBE0/def2-TZVPP = -1995.3939529445 (toluene correction)

Zero Point Energy (au) = 0.539309

Chemical potential (kJ mol^{-1}) = 1204.95

Dispersion correction (au) (RI)PBE0/def2-TZVPP = -0.1058511

Imaginary Frequency = -168.53

G_H

SCF Energy (au) (RI)BP86/SV(P) = -1995.810823895

SCF Energy (au) (RI)PBE0/def2-TZVPP = -1995.422839534

SCF Energy (au) (RI)PBE0/def2-TZVPP = -1995.4524078215 (toluene correction)

Zero Point Energy (au) = 0.5426446

Chemical potential (kJ mol^{-1}) = 1219.80

Dispersion correction (au) (RI)PBE0/def2-TZVPP = -0.1031982

Indole-vinylation via *O*-coordinated alkyne

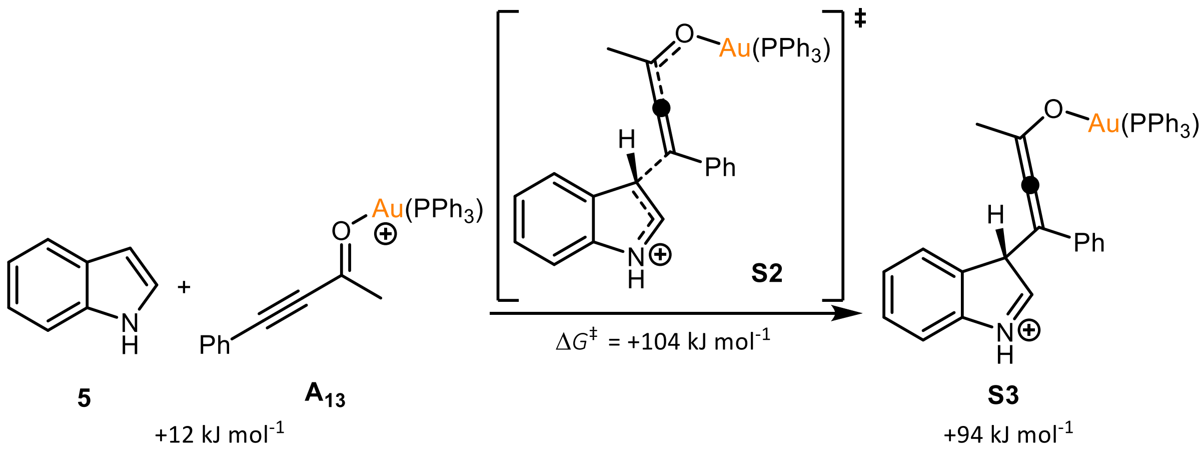

Scheme S3 - DFT-calculated energies for indole-vinylation via *O*-coordinated alkyne. All energies are Gibbs energies at 298.15 K at the D3(BJ)-PBE0/def2-TZVPP//BP86/SV(P) level of theory with COSMO solvent correction in toluene.

S2

SCF Energy (au) (RI)BP86/SV(P) = -1995.7524776720

SCF Energy (au) (RI)PBE0/def2-TZVPP = -1995.352933898

SCF Energy (au) (RI)PBE0/def2-TZVPP = -1995.3844713602 (toluene correction)

Zero Point Energy (au) = 0.5402090

Chemical potential (kJ mol^{-1}) = 1206.01

Dispersion correction (au) (RI)PBE0/def2-TZVPP = -0.10128635

Imaginary Frequency = -240.14

S3

SCF Energy (au) (RI)BP86/SV(P) = -1995.7533449390

SCF Energy (au) (RI)PBE0/def2-TZVPP = -1995.355520278

SCF Energy (au) (RI)PBE0/def2-TZVPP = -1995.3893407286 (toluene correction)

Zero Point Energy (au) = 0.5411315

Chemical potential (kJ mol^{-1}) = 1207.96

Dispersion correction (au) (RI)PBE0/def2-TZVPP = -0.10125116

Skatole-vinylation Pathway

$TS_{BD\text{-}Me}$

SCF Energy (au) (RI)BP86/SV(P) = -2035.03777929

SCF Energy (au) (RI)PBE0/def2-TZVPP = -2034.637710125

SCF Energy (au) (RI)PBE0/def2-TZVPP = -2034.6684668027 (toluene correction)

Zero Point Energy (au) = 0.5661915

Chemical potential (kJ mol^{-1}) = 1272.93

Dispersion correction (au) (RI)PBE0/def2-TZVPP = -0.11095758

Imaginary Frequency = -206.48

D_{Me}

SCF Energy (au) (RI)BP86/SV(P) = -2035.055831041

SCF Energy (au) (RI)PBE0/def2-TZVPP = -2034.662445266

SCF Energy (au) (RI)PBE0/def2-TZVPP = -2034.6970986464 (toluene correction)

Zero Point Energy (au) = 0.5684548

Chemical potential (kJ mol^{-1}) = 1278.17

Dispersion correction (au) (RI)PBE0/def2-TZVPP = -0.11105826

$TS_{DF\text{-}Me}$

SCF Energy (au) (RI)BP86/SV(P) = -2035.044687762

SCF Energy (au) (RI)PBE0/def2-TZVPP = -2034.649056256

SCF Energy (au) (RI)PBE0/def2-TZVPP = -2034.680970544 (toluene correction)

Zero Point Energy (au) = 0.566723

Chemical potential (kJ mol^{-1}) = 1280.28

Dispersion correction (au) (RI)PBE0/def2-TZVPP = -0.1112578

Imaginary Frequency = -205.29

G_{Me}

SCF Energy (au) (RI)BP86/SV(P) = -2035.096965902

SCF Energy (au) (RI)PBE0/def2-TZVPP = -2034.706156787

SCF Energy (au) (RI)PBE0/def2-TZVPP = -2034.735094009 (toluene correction)

Zero Point Energy (au) = 0.5695263

Chemical potential (kJ mol^{-1}) = 1286.98

Dispersion correction (au) (RI)PBE0/def2-TZVPP = -0.10868397

$TS_{BF\text{-}Me}$

SCF Energy (au) (RI)BP86/SV(P) = -2035.047906714

SCF Energy (au) (RI)PBE0/def2-TZVPP = -2034.645623605

SCF Energy (au) (RI)PBE0/def2-TZVPP = -2034.675198528 (toluene correction)

Zero Point Energy (au) = 0.5658431

Chemical potential (kJ mol^{-1}) = 1267.88

Dispersion correction (au) (RI)PBE0/def2-TZVPP = -0.11025582

Imaginary Frequency = -132.47

S4 – Methyl migration of D_{Me}

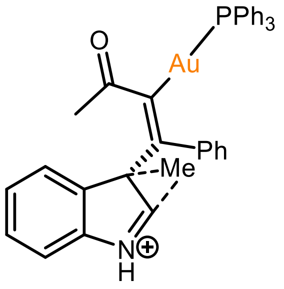

S4

SCF Energy (au) (RI)BP86/SV(P) = -2035.0271142550

SCF Energy (au) (RI)PBE0/def2-TZVPP = -2034.632425630

SCF Energy (au) (RI)PBE0/def2-TZVPP = -2034.6649797868 (toluene correction)

Zero Point Energy (au) = 0.5670328

Chemical potential (kJ mol^{-1}) = 1282.50

Dispersion correction (au) (RI)PBE0/def2-TZVPP = -0.10984228

Imaginary Frequency = -454.22

Indole Addition to Vinylated-indoles

16a

SCF Energy (au) (RI)BP86/SV(P) = -1187.9121417650
SCF Energy (au) (RI)PBE0/def2-TZVPP = -1187.774354790
SCF Energy (au) (RI)PBE0/def2-TZVPP = -1187.7862157405 (toluene correction)
Zero Point Energy (au) = 0.4033980
Chemical potential (kJ mol^{-1}) = 918.06
Dispersion correction (au) (RI)PBE0/def2-TZVPP = -0.07188470

14b

SCF Energy (au) (RI)BP86/SV(P) = -671.7997295493
SCF Energy (au) (RI)PBE0/def2-TZVPP = -671.7083153898
SCF Energy (au) (RI)PBE0/def2-TZVPP = -671.7154059794 (toluene correction)
Zero Point Energy (au) = 0.2373245
Chemical potential (kJ mol^{-1}) = 516.21
Dispersion correction (au) (RI)PBE0/def2-TZVPP = -0.03473201

16b

SCF Energy (au) (RI)BP86/SV(P) = -1035.3838472620
SCF Energy (au) (RI)PBE0/def2-TZVPP = -1035.251560445
SCF Energy (au) (RI)PBE0/def2-TZVPP = -1035.2618590222 (toluene correction)
Zero Point Energy (au) = 0.3668890
Chemical potential (kJ mol^{-1}) = 832.64
Dispersion correction (au) (RI)PBE0/def2-TZVPP = -0.06229923

H

SCF Energy (au) (RI)BP86/SV(P) = -824.7182729867
SCF Energy (au) (RI)PBE0/def2-TZVPP = -824.6283019641
SCF Energy (au) (RI)PBE0/def2-TZVPP = -824.6631364945 (toluene correction)
Zero Point Energy (au) = 0.2870169
Chemical potential (kJ mol^{-1}) = 635.19
Dispersion correction (au) (RI)PBE0/def2-TZVPP = -0.04310701

I

SCF Energy (au) (RI)BP86/SV(P) = -824.7181874903
SCF Energy (au) (RI)PBE0/def2-TZVPP = -824.6188330010
SCF Energy (au) (RI)PBE0/def2-TZVPP = -824.6542640271 (toluene correction)
Zero Point Energy (au) = 0.2862225
Chemical potential (kJ mol^{-1}) = 632.37
Dispersion correction (au) (RI)PBE0/def2-TZVPP = -0.04317682

TS_{HJ}

SCF Energy (au) (RI)BP86/SV(P) = -1188.2451706030
SCF Energy (au) (RI)PBE0/def2-TZVPP = -1188.112855975
SCF Energy (au) (RI)PBE0/def2-TZVPP = -1188.1505155213 (toluene correction)
Zero Point Energy (au) = 0.4138978
Chemical potential (kJ mol^{-1}) = 946.57
Dispersion correction (au) (RI)PBE0/def2-TZVPP = -0.07507751

Imaginary Frequency = -189.71

J

SCF Energy (au) (RI)BP86/SV(P) = -1188.2461178520
SCF Energy (au) (RI)PBE0/def2-TZVPP = -1188.117012228
SCF Energy (au) (RI)PBE0/def2-TZVPP = -1188.1554070038 (toluene correction)
Zero Point Energy (au) = 0.4145531
Chemical potential (kJ mol^{-1}) = 946.70
Dispersion correction (au) (RI)PBE0/def2-TZVPP = -0.07516109

TS_{IK}

SCF Energy (au) (RI)BP86/SV(P) = -1188.2677398670
SCF Energy (au) (RI)PBE0/def2-TZVPP = -1188.124890968
SCF Energy (au) (RI)PBE0/def2-TZVPP = -1188.1619196915 (toluene correction)
Zero Point Energy (au) = 0.4143763
Chemical potential (kJ mol^{-1}) = 946.58
Dispersion correction (au) (RI)PBE0/def2-TZVPP = -0.07386064

Imaginary Frequency = -208.58

K

SCF Energy (au) (RI)BP86/SV(P) = -1188.2691421060
SCF Energy (au) (RI)PBE0/def2-TZVPP = -1188.130463447
SCF Energy (au) (RI)PBE0/def2-TZVPP = -1188.1694344729 (toluene correction)
Zero Point Energy (au) = 0.4150524
Chemical potential (kJ mol^{-1}) = 947.41
Dispersion correction (au) (RI)PBE0/def2-TZVPP = -0.07516109

L

SCF Energy (au) (RI)BP86/SV(P) = -672.1840148495

SCF Energy (au) (RI)PBE0/def2-TZVPP = -672.0925518944

SCF Energy (au) (RI)PBE0/def2-TZVPP = -672.1269344973 (toluene correction)

Zero Point Energy (au) = 0.2498422

Chemical potential (kJ mol^{-1}) = 549.64

Dispersion correction (au) (RI)PBE0/def2-TZVPP = -0.03560389

TS_{LM}

SCF Energy (au) (RI)BP86/SV(P) = -1035.7399856820

SCF Energy (au) (RI)PBE0/def2-TZVPP = -1035.601786004

SCF Energy (au) (RI)PBE0/def2-TZVPP = -1035.6361779450 (toluene correction)

Zero Point Energy (au) = 0.3781098

Chemical potential (kJ mol^{-1}) = 863.95

Dispersion correction (au) (RI)PBE0/def2-TZVPP = -0.06470281

Imaginary Frequency = -248.82

M

SCF Energy (au) (RI)BP86/SV(P) = -1035.7447503700

SCF Energy (au) (RI)PBE0/def2-TZVPP = -1035.612121170

SCF Energy (au) (RI)PBE0/def2-TZVPP = -1035.6486314432 (toluene correction)

Zero Point Energy (au) = 0.3791144

Chemical potential (kJ mol^{-1}) = 865.60

Dispersion correction (au) (RI)PBE0/def2-TZVPP = -0.06425161

Pyrylium Complex Formation

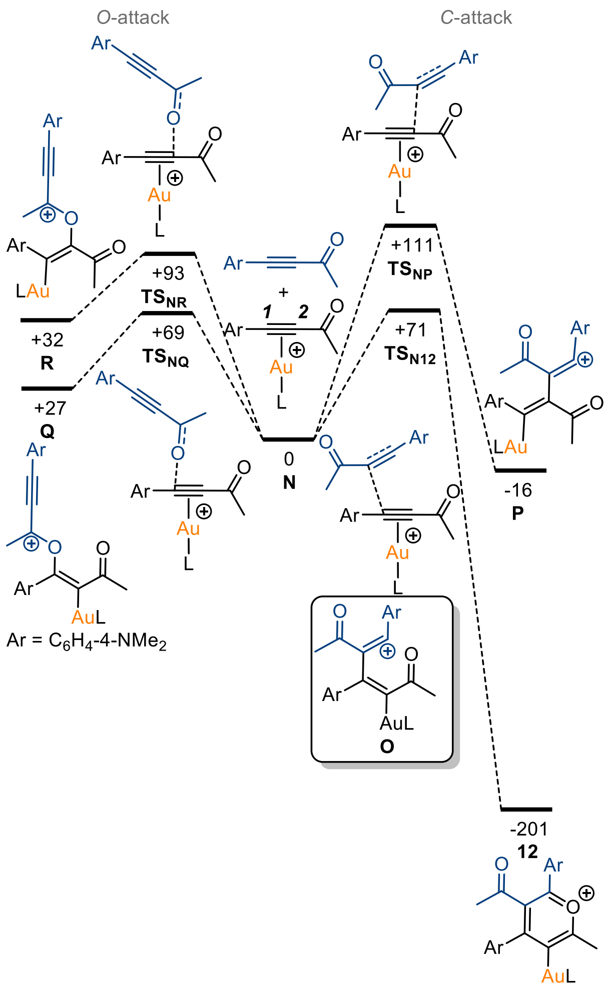

Scheme 4 - DFT-calculated pathways for the gold-mediated dimerization of alkynes. All energies are Gibbs energies at 298.15 K at the D3(BJ)-PBE0/def2-TZVPP//BP86/SV(P) level of theory with COSMO solvent correction in CH_2Cl_2.

Table S4 - DFT-calculated pathways for the gold-mediated dimerization of alkynes. All energies are Gibbs energies at 298.15 K at the D3(BJ)-PBE0/def2-TZVPP//BP86/SV(P) level of theory with COSMO solvent correction in CH_2Cl_2.

Entry	SI Page Numbers	Ar	L	TS_{N12}	TS_{NQ}	Q	12
1	S96 – S97	C_6H_4-4-NMe_2	PPh_3	+71	+69	+27	−201
2	S98	C_6H_5	PPh_3	+75	+63	+23	−224
3	S99	C_6H_4-4-OMe	PPh_3	+69	+61	+20	−220
4	S100 – S101	C_6H_4-4-NMe_2	PMe_3	+61	+70	+33	−197
5	S102 – S103	C_6H_4-4-NMe_2	IPr	+77	+77	+33	−198
6	S104 – S105	C_6H_4-4-NMe_2	JohnPhos	+70	+75	+37	−199

Pyrylium Complex Formation, Entry 1 (Table S4)

TS_{N12}

SCF Energy (au) (RI)BP86/SV(P) = -2360.6749694420
SCF Energy (au) (RI)PBE0/def2-TZVPP = -2360.244236366
SCF Energy (au) (RI)PBE0/def2-TZVPP = -2360.2963469339 (DCM correction)
Zero Point Energy (au) = 0.6976376
Chemical potential (kJ mol^{-1}) = 1572.75
Dispersion correction (au) (RI)PBE0/def2-TZVPP = -0.12121997

Imaginary Frequency = -265.58

12

SCF Energy (au) (RI)BP86/SV(P) = -2360.7846411010
SCF Energy (au) (RI)PBE0/def2-TZVPP = -2360.361968336
SCF Energy (au) (RI)PBE0/def2-TZVPP = -2360.4101137061 (DCM correction)
Zero Point Energy (au) = 0.7040260
Chemical potential (kJ mol^{-1}) = 1605.93
Dispersion correction (au) (RI)PBE0/def2-TZVPP = -0.12561242

TS_{NP}

SCF Energy (au) (RI)BP86/SV(P) = -2360.6598133890
SCF Energy (au) (RI)PBE0/def2-TZVPP = -2360.226830510
SCF Energy (au) (RI)PBE0/def2-TZVPP = -2360.2767538449 (DCM correction)
Zero Point Energy (au) = 0.6969677
Chemical potential (kJ mol^{-1}) = 1573.82
Dispersion correction (au) (RI)PBE0/def2-TZVPP = -0.12387095

Imaginary Frequency = -302.67

P

SCF Energy (au) (RI)BP86/SV(P) = -2360.7039842940
SCF Energy (au) (RI)PBE0/def2-TZVPP = -2360.269703374
SCF Energy (au) (RI)PBE0/def2-TZVPP = -2360.3319787962 (DCM correction)
Zero Point Energy (au) = 0.6999262
Chemical potential (kJ mol^{-1}) = 1586.43
Dispersion correction (au) (RI)PBE0/def2-TZVPP = -0.12385385

TS_{NQ}

SCF Energy (au) (RI)BP86/SV(P) = -2360.6823336050
SCF Energy (au) (RI)PBE0/def2-TZVPP = -2360.249682351
SCF Energy (au) (RI)PBE0/def2-TZVPP = -2360.2997101041 (DCM correction)
Zero Point Energy (au) = 0.6980823
Chemical potential (kJ mol^{-1}) = 1572.54
Dispersion correction (au) (RI)PBE0/def2-TZVPP = -0.11857721

Imaginary Frequency = -193.70

Q

SCF Energy (au) (RI)BP86/SV(P) = -2360.6924042150
SCF Energy (au) (RI)PBE0/def2-TZVPP = -2360.263499090
SCF Energy (au) (RI)PBE0/def2-TZVPP = -2360.3163213398 (DCM correction)
Zero Point Energy (au) = 0.6999778
Chemical potential (kJ mol^{-1}) = 1579.60
Dispersion correction (au) (RI)PBE0/def2-TZVPP = -0.12054704

TS_{NR}

SCF Energy (au) (RI)BP86/SV(P) = -2360.6757455720
SCF Energy (au) (RI)PBE0/def2-TZVPP = -2360.241487197
SCF Energy (au) (RI)PBE0/def2-TZVPP = -2360.2895174469 (DCM correction)
Zero Point Energy (au) = 0.6980338
Chemical potential (kJ mol^{-1}) = 1572.93
Dispersion correction (au) (RI)PBE0/def2-TZVPP = -0.11980710

Imaginary Frequency = -153.09

R

SCF Energy (au) (RI)BP86/SV(P) = -2360.6970697230
SCF Energy (au) (RI)PBE0/def2-TZVPP = -2360.263571665
SCF Energy (au) (RI)PBE0/def2-TZVPP = -2360.2895174469 (DCM correction)
Zero Point Energy (au) = 0.7005929
Chemical potential (kJ mol^{-1}) = 1585.98
Dispersion correction (au) (RI)PBE0/def2-TZVPP = -0.12042466

Pyrylium Complex Formation, Entry 2 (Table S4)

TS_{N12}

SCF Energy (au) (RI)BP86/SV(P) = -2092.9104606630
SCF Energy (au) (RI)PBE0/def2-TZVPP = -2092.503430210
SCF Energy (au) (RI)PBE0/def2-TZVPP = -2092.5540674371 (DCM correction)
Zero Point Energy (au) = 0.5554871
Chemical potential (kJ mol^{-1}) = 1228.81
Dispersion correction (au) (RI)PBE0/def2-TZVPP = -0.10486719

Imaginary Frequency = -275.34

12

SCF Energy (au) (RI)BP86/SV(P) = -2093.0238439160
SCF Energy (au) (RI)PBE0/def2-TZVPP = -2092.625982597
SCF Energy (au) (RI)PBE0/def2-TZVPP = -2092.6766973562 (DCM correction)
Zero Point Energy (au) = 0.5620013
Chemical potential (kJ mol^{-1}) = 1260.96
Dispersion correction (au) (RI)PBE0/def2-TZVPP = -0.10853329

TS_{NQ}

SCF Energy (au) (RI)BP86/SV(P) = -2092.9209589260
SCF Energy (au) (RI)PBE0/def2-TZVPP = -2092.512301144
SCF Energy (au) (RI)PBE0/def2-TZVPP = -2092.5619629181 (DCM correction)
Zero Point Energy (au) = 0.5560835
Chemical potential (kJ mol^{-1}) = 1229.61
Dispersion correction (au) (RI)PBE0/def2-TZVPP = -0.10181690

Imaginary Frequency = -149.78

Q

SCF Energy (au) (RI)BP86/SV(P) = -2092.524632710
SCF Energy (au) (RI)PBE0/def2-TZVPP = -2092.512301144
SCF Energy (au) (RI)PBE0/def2-TZVPP = -2092.5773119580 (DCM correction)
Zero Point Energy (au) = 0.5578354
Chemical potential (kJ mol^{-1}) = 1234.09
Dispersion correction (au) (RI)PBE0/def2-TZVPP = -0.10355302

Pyrylium Complex Formation, Entry 3 (Table S4)

$\mathbf{TS_{N12}}$

SCF Energy (au) (RI)BP86/SV(P) = -2321.8097492340
SCF Energy (au) (RI)PBE0/def2-TZVPP = -2321.403755638
SCF Energy (au) (RI)PBE0/def2-TZVPP = -2321.4563970844 (DCM correction)
Zero Point Energy (au) = 0.6187721
Chemical potential (kJ mol^{-1}) = 1378.38
Dispersion correction (au) (RI)PBE0/def2-TZVPP = -0.11200256

Imaginary Frequency = -271.25

12

SCF Energy (au) (RI)BP86/SV(P) = -2321.9171969240
SCF Energy (au) (RI)PBE0/def2-TZVPP = -2321.520786012
SCF Energy (au) (RI)PBE0/def2-TZVPP = -2321.5738181876 (DCM correction)
Zero Point Energy (au) = 0.6249092
Chemical potential (kJ mol^{-1}) = 1408.58
Dispersion correction (au) (RI)PBE0/def2-TZVPP = -0.11632687

$\mathbf{TS_{NQ}}$

SCF Energy (au) (RI)BP86/SV(P) = -2321.8193095010
SCF Energy (au) (RI)PBE0/def2-TZVPP = -2321.411582247
SCF Energy (au) (RI)PBE0/def2-TZVPP = -2321.4625677723 (DCM correction)
Zero Point Energy (au) = 0.6192996
Chemical potential (kJ mol^{-1}) = 1378.68
Dispersion correction (au) (RI)PBE0/def2-TZVPP = -0.10922311

Imaginary Frequency = -174.36

Q

SCF Energy (au) (RI)BP86/SV(P) = -2321.8281280680
SCF Energy (au) (RI)PBE0/def2-TZVPP = -2321.424195614
SCF Energy (au) (RI)PBE0/def2-TZVPP = -2321.4783323347 (DCM correction)
Zero Point Energy (au) = 0.6211509
Chemical potential (kJ mol^{-1}) = 1384.66
Dispersion correction (au) (RI)PBE0/def2-TZVPP = -0.11104747

Pyrylium Complex Formation, Entry 4 (Table S4)

B_{47} (PMe_3)

SCF Energy (au) (RI)BP86/SV(P) = -1191.2785455390
SCF Energy (au) (RI)PBE0/def2-TZVPP = -1191.014118887
SCF Energy (au) (RI)PBE0/def2-TZVPP = -1191.0679994467 (DCM correction)
Zero Point Energy (au) = 0.3267839
Chemical potential (kJ mol^{-1}) = 696.82
Dispersion correction (au) (RI)PBE0/def2-TZVPP = -0.04363209

TS_{N12}

SCF Energy (au) (RI)BP86/SV(P) = -1785.8669061570
SCF Energy (au) (RI)PBE0/def2-TZVPP = -1785.532565921
SCF Energy (au) (RI)PBE0/def2-TZVPP = -1785.5856284503 (DCM correction)
Zero Point Energy (au) = 0.5412390
Chemical potential (kJ mol^{-1}) = 1190.72
Dispersion correction (au) (RI)PBE0/def2-TZVPP = -0.08146727

Imaginary Frequency = -267.12

12

SCF Energy (au) (RI)BP86/SV(P) = -1785.9720487850
SCF Energy (au) (RI)PBE0/def2-TZVPP = -1785.646361631
SCF Energy (au) (RI)PBE0/def2-TZVPP = -1785.6968846080 (DCM correction)
Zero Point Energy (au) = 0.5472129
Chemical potential (kJ mol^{-1}) = 1234.00
Dispersion correction (au) (RI)PBE0/def2-TZVPP = -0.08507509

TS_{NQ}

SCF Energy (au) (RI)BP86/SV(P) = -1785.8747034310
SCF Energy (au) (RI)PBE0/def2-TZVPP = -1785.538315508
SCF Energy (au) (RI)PBE0/def2-TZVPP = -1785.5890198823 (DCM correction)
Zero Point Energy (au) = 0.5416343
Chemical potential (kJ mol^{-1}) = 1201.56
Dispersion correction (au) (RI)PBE0/def2-TZVPP = -0.07859298

Imaginary Frequency = -178.24

Q

SCF Energy (au) (RI)BP86/SV(P) = -1785.8853569020
SCF Energy (au) (RI)PBE0/def2-TZVPP = -1785.552554117
SCF Energy (au) (RI)PBE0/def2-TZVPP = -1785.6049736990 (DCM correction)
Zero Point Energy (au) = 0.5435797
Chemical potential (kJ mol^{-1}) = 1210.48
Dispersion correction (au) (RI)PBE0/def2-TZVPP = -0.08017555

Pyrylium Complex Formation, Entry 5 (Table S4)

B_{47} (IPr)

SCF Energy (au) (RI)BP86/SV(P) = -1889.5494993860
SCF Energy (au) (RI)PBE0/def2-TZVPP = -1889.208012637
SCF Energy (au) (RI)PBE0/def2-TZVPP = -1889.2593977330 (DCM correction)
Zero Point Energy (au) = 0.7698924
Chemical potential (kJ mol^{-1}) = 1774.88
Dispersion correction (au) (RI)PBE0/def2-TZVPP = -0.12341676

TS_{N12}

SCF Energy (au) (RI)BP86/SV(P) = -2484.1335431210
SCF Energy (au) (RI)PBE0/def2-TZVPP = -2483.722301057
SCF Energy (au) (RI)PBE0/def2-TZVPP = -2483.7746865910 (DCM correction)
Zero Point Energy (au) = 0.9844650
Chemical potential (kJ mol^{-1}) = 2284.50
Dispersion correction (au) (RI)PBE0/def2-TZVPP = -0.16348550

Imaginary Frequency = -289.10

12

SCF Energy (au) (RI)BP86/SV(P) = -2484.2353616210
SCF Energy (au) (RI)PBE0/def2-TZVPP = -2483.834778287
SCF Energy (au) (RI)PBE0/def2-TZVPP = -2483.8841689738 (DCM correction)
Zero Point Energy (au) = 0.9902886
Chemical potential (kJ mol^{-1}) = 2316.55
Dispersion correction (au) (RI)PBE0/def2-TZVPP = -0.17103717

TS_{NQ}

SCF Energy (au) (RI)BP86/SV(P) = -2484.1413084600
SCF Energy (au) (RI)PBE0/def2-TZVPP = -2483.728569956
SCF Energy (au) (RI)PBE0/def2-TZVPP = -2483.7771550399 (DCM correction)
Zero Point Energy (au) = 0.9849174
Chemical potential (kJ mol^{-1}) = 2286.87
Dispersion correction (au) (RI)PBE0/def2-TZVPP = -0.16180011

Imaginary Frequency = -199.49

Q

SCF Energy (au) (RI)BP86/SV(P) = -2484.1500162980
SCF Energy (au) (RI)PBE0/def2-TZVPP = -2483.740787210
SCF Energy (au) (RI)PBE0/def2-TZVPP = -2483.7926899168 (DCM correction)
Zero Point Energy (au) = 0.9866949
Chemical potential (kJ mol^{-1}) = 2293.60
Dispersion correction (au) (RI)PBE0/def2-TZVPP = -0.16556991

Pyrylium Complex Formation, Entry 6 (Table S4)

B_{47} (JohnPhos)

SCF Energy (au) (RI)BP86/SV(P) = -1849.4327822990
SCF Energy (au) (RI)PBE0/def2-TZVPP = -1849.086732006
SCF Energy (au) (RI)PBE0/def2-TZVPP = -1849.1365857858 (DCM correction)
Zero Point Energy (au) = 0.6213898
Chemical potential (kJ mol^{-1}) = 1421.55
Dispersion correction (au) (RI)PBE0/def2-TZVPP = -0.10966167

TS_{N12}

SCF Energy (au) (RI)BP86/SV(P) = -2444.0177721650
SCF Energy (au) (RI)PBE0/def2-TZVPP = -2443.602657239
SCF Energy (au) (RI)PBE0/def2-TZVPP = -2443.6524055639 (DCM correction)
Zero Point Energy (au) = 0.8357224
Chemical potential (kJ mol^{-1}) = 1926.98
Dispersion correction (au) (RI)PBE0/def2-TZVPP = -0.15026615

Imaginary Frequency = -281.15

12

SCF Energy (au) (RI)BP86/SV(P) = -2444.1191322730
SCF Energy (au) (RI)PBE0/def2-TZVPP = -2443.713147288
SCF Energy (au) (RI)PBE0/def2-TZVPP = -2443.7624477294 (DCM correction)
Zero Point Energy (au) = 0.8418029
Chemical potential (kJ mol^{-1}) = 1959.76
Dispersion correction (au) (RI)PBE0/def2-TZVPP = -0.15505522

TS_{NQ}

SCF Energy (au) (RI)BP86/SV(P) = -2444.0242257540
SCF Energy (au) (RI)PBE0/def2-TZVPP = -2443.606679200
SCF Energy (au) (RI)PBE0/def2-TZVPP = -2443.6549321098 (DCM correction)
Zero Point Energy (au) = 0.8362389
Chemical potential (kJ mol^{-1}) = 1928.81
Dispersion correction (au) (RI)PBE0/def2-TZVPP = -0.14638001

Imaginary Frequency = -193.59

Q

SCF Energy (au) (RI)BP86/SV(P) = -2444.0332850660
SCF Energy (au) (RI)PBE0/def2-TZVPP = -2443.620021433
SCF Energy (au) (RI)PBE0/def2-TZVPP = -2443.6703090336 (DCM correction)
Zero Point Energy (au) = 0.8383866
Chemical potential (kJ mol^{-1}) = 1940.27
Dispersion correction (au) (RI)PBE0/def2-TZVPP = -0.14988708

Triflimide-containing structures

S

SCF Energy (au) (RI)BP86/SV(P) = -2997.9838561660

SCF Energy (au) (RI)PBE0/def2-TZVPP = -2997.840518194

SCF Energy (au) (RI)PBE0/def2-TZVPP = -2997.8611765265 (DCM correction)

SCF Energy (au) (RI)PBE0/def2-TZVPP = -2997.8516084024 (toluene correction)

Zero Point Energy (au) = 0.3202133

Chemical potential (kJ mol^{-1}) = 638.20

Dispersion correction (au) (RI)PBE0/def2-TZVPP = -0.07724908

T

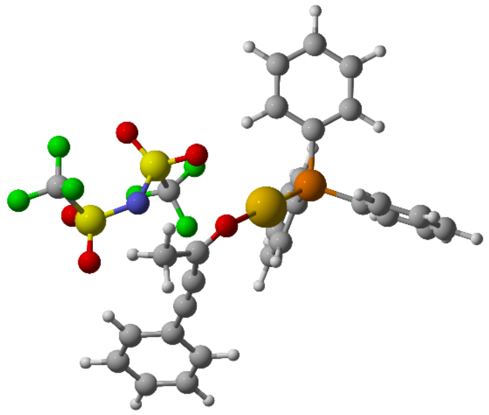

Figure S66 - 3D representation of **T**.

SCF Energy (au) (RI)BP86/SV(P) = -3458.6865462940

SCF Energy (au) (RI)PBE0/def2-TZVPP = -3458.490805646

SCF Energy (au) (RI)PBE0/def2-TZVPP = -3458.5194562951 (DCM correction)

SCF Energy (au) (RI)PBE0/def2-TZVPP = -3458.5060213051 (toluene correction)

Zero Point Energy (au) = 0.4649554

Chemical potential (kJ mol^{-1}) = 976.79

Dispersion correction (au) (RI)PBE0/def2-TZVPP = -0.10767517

U

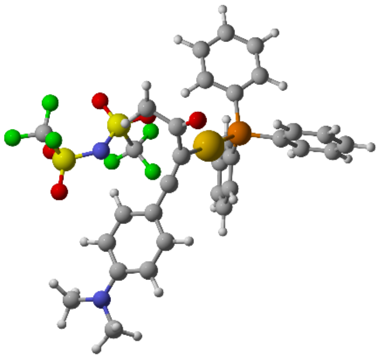

Figure S67 - 3D representation of **U**.

SCF Energy (au) (RI)BP86/SV(P) = -3592.5636693700

SCF Energy (au) (RI)PBE0/def2-TZVPP = -3592.359785784

SCF Energy (au) (RI)PBE0/def2-TZVPP = -3592.3951982271 (DCM correction)

SCF Energy (au) (RI)PBE0/def2-TZVPP = -3592.3785085889 (toluene correction)

Zero Point Energy (au) = 0.5353944

Chemical potential (kJ mol^{-1}) = 1144.44

Dispersion correction (au) (RI)PBE0/def2-TZVPP = -0.11515110

V

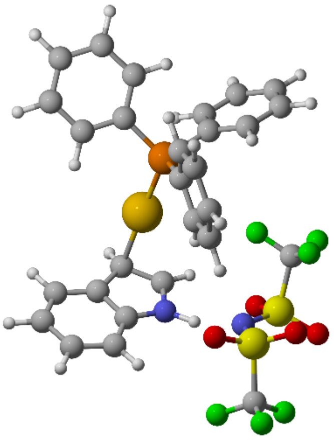

Figure S68 - 3D representation of **V**.

SCF Energy (au) (RI)BP86/SV(P) = -3361.5510180310

SCF Energy (au) (RI)PBE0/def2-TZVPP = -3361.366596675

SCF Energy (au) (RI)PBE0/def2-TZVPP = -3361.3931456927 (DCM correction)

SCF Energy (au) (RI)PBE0/def2-TZVPP = -3361.3807503525 (toluene correction)

Zero Point Energy (au) = 0.4473011

Chemical potential (kJ mol^{-1}) = 944.09

Dispersion correction (au) (RI)PBE0/def2-TZVPP = -0.10367452

W

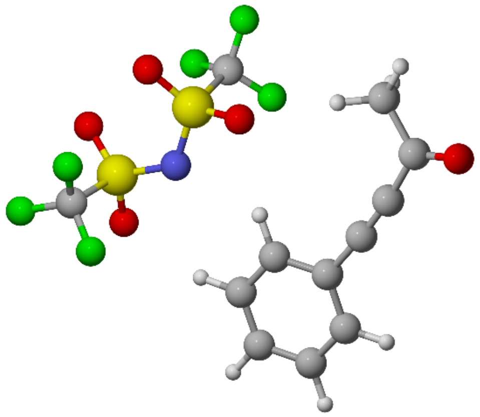

Figure S69 - 3D representation of **W**.

SCF Energy (au) (RI)BP86/SV(P) = -2287.1062729660
SCF Energy (au) (RI)PBE0/def2-TZVPP = -2287.213256625
SCF Energy (au) (RI)PBE0/def2-TZVPP = -2287.2761458412 (DCM correction)
SCF Energy (au) (RI)PBE0/def2-TZVPP = -2287.2482222793 (toluene correction)
Zero Point Energy (au) = 0.1950783
Chemical potential (kJ mol^{-1}) = 337.30
Dispersion correction (au) (RI)PBE0/def2-TZVPP = -0.03960358

X

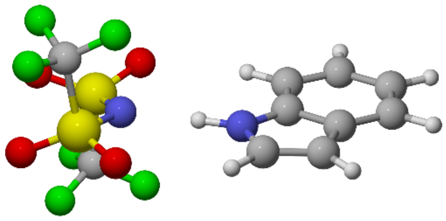

Figure S70 - 3D representation of **X**.

SCF Energy (au) (RI)BP86/SV(P) = -2189.9670518120
SCF Energy (au) (RI)PBE0/def2-TZVPP = -2190.080192108
SCF Energy (au) (RI)PBE0/def2-TZVPP = -2190.1401226088 (DCM correction)
SCF Energy (au) (RI)PBE0/def2-TZVPP = -2190.1137207103 (toluene correction)
Zero Point Energy (au) = 0.1783495
Chemical potential (kJ mol^{-1}) = 314.13
Dispersion correction (au) (RI)PBE0/def2-TZVPP = -0.03960358

Y

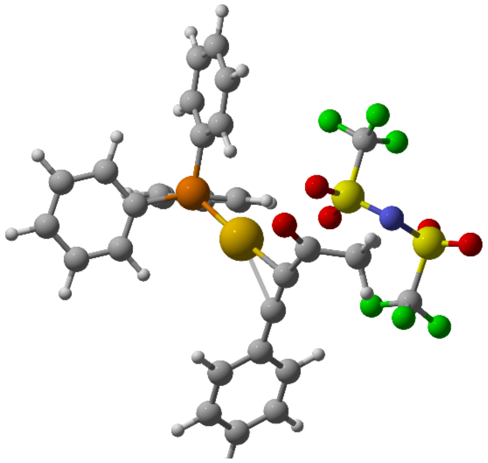

Figure S71 - 3D representation of **Y**.

SCF Energy (au) (RI)BP86/SV(P) = -3458.6773345890

SCF Energy (au) (RI)PBE0/def2-TZVPP = -3458.485785644

SCF Energy (au) (RI)PBE0/def2-TZVPP = -3458.5176452016 (DCM correction)

SCF Energy (au) (RI)PBE0/def2-TZVPP = -3458.5027082271 (toluene correction)

Zero Point Energy (au) = 0.4636723

Chemical potential (kJ mol^{-1}) = 969.10

Dispersion correction (au) (RI)PBE0/def2-TZVPP = -0.10734556

Z

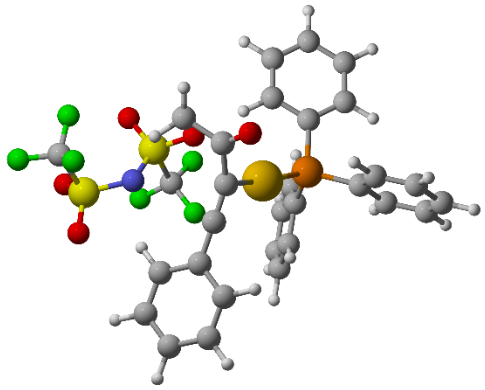

Figure S72 - 3D representation of **Z**.

SCF Energy (au) (RI)BP86/SV(P) = -3458.6809119500

SCF Energy (au) (RI)PBE0/def2-TZVPP = -3458.487624394

SCF Energy (au) (RI)PBE0/def2-TZVPP = -3458.5186259732 (DCM correction)

SCF Energy (au) (RI)PBE0/def2-TZVPP = -3458.5041272969 (toluene correction)

Zero Point Energy (au) = 0.4640795

Chemical potential (kJ mol^{-1}) = 971.77

Dispersion correction (au) (RI)PBE0/def2-TZVPP = -0.10756225

Gold-catalysed 1,3-*O*-transposition of Ynones

As mentioned in the main paper, one of the possible side products from intermediate **Q** is a cyclic acetal complex (**S5**), as reported in a detailed mechanistic study into the gold-catalysed 1,3-*O*-transposition reaction of ynones.[29]

The full proposed mechanism of this process is shown below (Scheme S5). Transposed product (**S7**) has been observed in ^{1}H NMR spectra of the crude reaction mixtures.

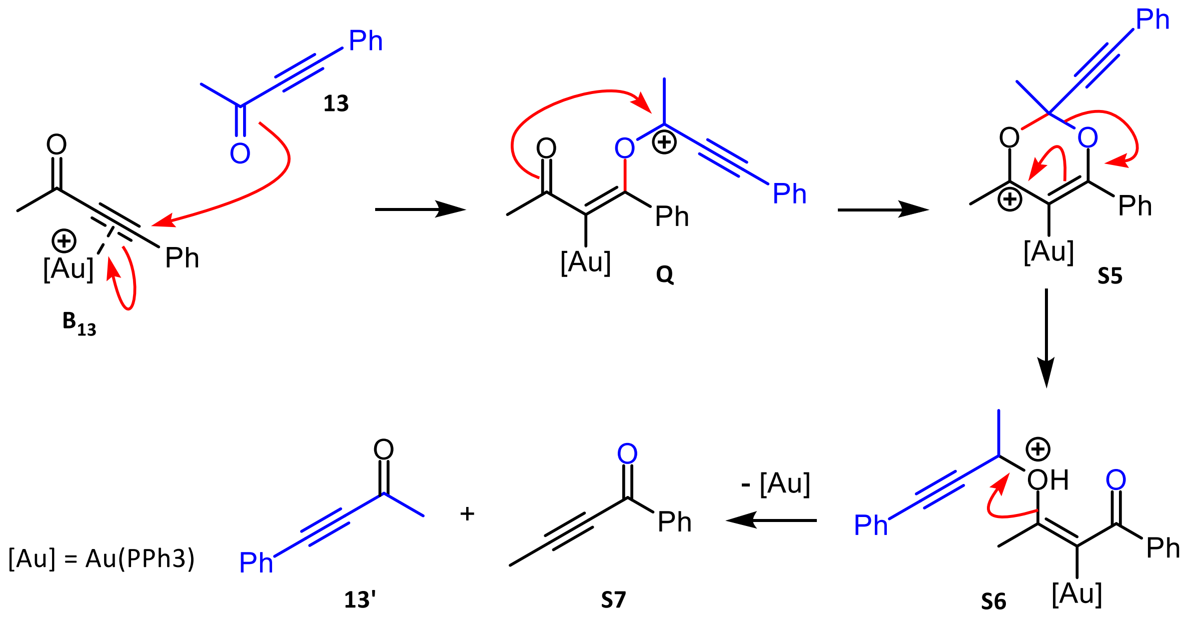

Scheme S5 - Proposed mechanism of the gold-catalysed 1,3-O-transposition of ynones.[28]

References

1. Godoi, M. N.; de Azambuja, F.; Martinez, P. D. G.; Morgon, N. H.; Santos, V. G.; Regiani, T.; Lesage, D.; Dossmann, H.; Cole, R. B.; Eberlin, M. N.; Correia, C. R. D. Revisiting the Intermolecular Fujiwara Hydroarylation of Alkynes *Eur. J. Org. Chem.* **2017**, *2017*, 1794–1803.
2. Dolomanov, O. V.; Bourhis, L. J.; Gildea, R. J.; Howard, J. A. K.; Puschmann, H. OLEX2: a complete structure solution, refinement and analysis program *J. Appl. Cryst.* **2009**, *42*, 339–341.
3. Sheldrick, G. M. SHELXT - Integrated space-group and crystal-structure determination *Acta Cryst.* **2015**, *A71*, 3–8.
4. Sheldrick, G. M. Crystal structure refinement with SHELXL *Acta Cryst.* **2015**, *C71*, 3–8.
5. Islam, S.; Larrosa, I.; “On Water”, Phosphine-Free Palladium-Catalyzed Room Temperature C–H Arylation of Indoles. *Chem. Eur. J.* **2013**, *19*, 15093–15096.
6. Bedford, R. B.; Fey, N.; Haddow, M. F.; Sankey, R. F. Remarkably reactive dihydroindoloindoles via palladium-catalysed dearomatisation. *Chem. Commun.* **2011**, *47*, 3649–3651.
7. Shao, C.; Shi, G.; Zhang, Y.; Pan, S.; Guan, X. Palladium-Catalyzed C–H Ethoxycarbonyldifluoromethylation of Electron-Rich Heteroarenes. *Org. Lett.* **2015**, *17*, 2652–2655.
8. Mo, F.; Lim, H. N.; Dong, G. Bifunctional Ligand-Assisted Catalytic Ketone α-Alkenylation with Internal Alkynes: Controlled Synthesis of Enones and Mechanistic Studies. *J. Am. Chem. Soc.* **2015**, *137*, 15518–15527.
9. Zi, Y.; Schömberg, F.; Seifert, F.; Görls, H.; Vilotijevic, I. *trans*-Hydroboration *vs*. 1,2-reduction: divergent reactivity of ynones and ynoates in Lewis-base-catalyzed reactions with pinacolborane. *Org. Biomol. Chem.* **2018**, *16*, 6341–6349.
10. Schubert, T.; Hummel, W.; Kula, M. R.; Müller, M.; Enantioselective Synthesis of Both Enantiomers of Various Propargylic Alcohols by Use of Two Oxidoreductases. *Eur. J. Org. Chem.* **2001**, *22*, 4181–4187.
11. More, A. A.; Szpilman, A. M.; Indium(III) Catalyzed Reactions of Vinyl Azides and Indoles. *Org. Lett.* **2020**, *22*, 3759–3764.
12. Xu, K.; Chen, W.; Lin, J.; Chen, G.; Wang, B.; Tian, X.; Facile synthesis of 9*H*-pyrrolo[1,2-a]indoles via Brønsted acid catalyzed cascade reactions. *Chem. Commun.* **2019**, *55*, 14613–14616.

13 Császár, P.; Pulay, P. Geometry optimization by direct inversion in the iterative subspace. *J. Mol. Struct.* **1984**, *114*, 31–34.

14 Ahlrichs, R.; Bär, M.; Häser, M.; Horn, H.; Kölmel, C. Electronic structure calculations on workstation computers: The program system turbomole. *Chem. Phys. Lett.* **1989**, *162*, 165–169.

15 Deglmann, P.; Furche, F.; Ahlrichs, R. An efficient implementation of second analytical derivatives for density functional methods. *Chem. Phys. Lett.* **2002**, *362*, 511–518.

16 Deglmann, P.; May, K.; Furche, F.; Ahlrichs, R. Nuclear second analytical derivative calculations using auxiliary basis set expansions. *Chem. Phys. Lett.* **2004**, *384*, 103–107.

17 Eichkorn, K.; Treutler, O.; Öhm, H.; Häser, M; Ahlrichs, R. Auxiliary basis sets to approximate Coulomb potentials. *Chem. Phys. Lett.* **1995**, *240*, 283–290.

18 Eichkorn, K.; Weigend, F.; Treutler, O.; Ahlrichs, R. Auxiliary basis sets for main row atoms and transition metals and their use to approximate Coulomb potentials. *Theor. Chem. Acc.* **1997**, *97*, 119–124.

19 Treutler, O.; Ahlrichs, R. Efficient molecular numerical integration schemes. *J. Chem. Phys.* **1995**, *102*, 346–354.

20 von Arnim, M.; Ahlrichs, R. Geometry optimization in generalized natural internal coordinates. *J. Chem. Phys.* **1999**, *111*, 9183–9190.

21 Schäfer, A.; Horn, H.; Ahlrichs, R. Fully optimized contracted Gaussian basis sets for atoms Li to Kr. *J. Chem. Phys.* **1992**, *97*, 2571–2577.

22 Weigend, F.; Häser, M.; Patzelt, H.; Ahlrichs, R. RI-MP2: optimized auxiliary basis sets and demonstration of efficiency. Chem. Phys. Letters **1998**, *294*, 143–152.

23 Weigend, F.; Ahlrichs, R. Balanced basis sets of split valence, triple zeta valence and quadruple zeta valence quality for H to Rn: Design and assessment of accuracy. *Phys. Chem. Chem. Phys*. **2005**, *7*, 3297–3305.

24 Weigend, F. Accurate Coulomb-fitting basis sets for H to Rn. *Phys. Chem. Chem. Phys.* **2006**, *8*, 1057–1065.

25 Klamt, A.; Schuurmann, G. COSMO: a new approach to dielectric screening in solvents with explicit expressions for the screening energy and its gradient. *J. Chem. Soc., Perk. Trans. 2* **1993**, *5*, 799–805.

26 Grimme, S.; Antony, J.; Ehrlich, S.; Krieg, H. A consistent and accurate ab initio parametrization of density functional dispersion correction (DFT-D) for the 94 elements H-Pu. *J. Chem. Phys.* **2010**, *132*, 154104–154123.

27 Grimme, S.; Ehrlich, S.; Goerigk, L. Effect of the damping function in dispersion corrected density functional theory. *J. Comput. Chem.* **2011**, *32*, 1456–1465.

28 CYLview, 1.0b; Legault, C. Y., Université de Sherbrooke, 2009 (http://www.cylview.org)

29 Aikonen, S.; Muuronen, M.; Wirtanen, T.; Heikkinen, S.; Musgreave, J.; Burés, J.; Helaja, J. Gold(I)-Catalyzed 1,3-O-Transposition of Ynones: Mechanism and Catalytic Acceleration with Electron-Rich Aldehydes. *ACS Catal.* **2018**, *8*, 960–967.
